# Regulatory role of resveratrol, a microRNA-controlling compound, in *HNRNPA1* expression, which is associated with poor prognosis in breast cancer

**DOI:** 10.18632/oncotarget.25339

**Published:** 2018-05-15

**Authors:** Kurataka Otsuka, Yusuke Yamamoto, Takahiro Ochiya

**Affiliations:** ^1^ Division of Molecular and Cellular Medicine, National Cancer Center Research Institute, Chuo-ku, Tokyo 104-0045, Japan; ^2^ R&D Division, Kewpie Corporation Sengawa Kewport, Chofu-shi, Tokyo 182-0002, Japan

**Keywords:** resveratrol, microRNA, HNRNPA1, breast cancer, diet

## Abstract

Certain lifestyles, such as unhealthy eating habits, are associated with an increased risk for several diseases, including cancer. Recently, some naturally occurring compounds, such as resveratrol, have been shown to regulate microRNA (miRNA) expression in a positive manner; this regulatory activity is likely to be advantageous for cancer prevention and treatment. Resveratrol, a multi-functional polyphenolic phytoalexin, has been known to exert anti-tumorigenic and anti-inflammatory effects and to regulate miRNA expression. However, our understanding of the underlying molecular mechanisms whereby resveratrol controls cancer cell growth via the regulation of miRNA and oncogenic target gene expression to inhibit disease progression remains incomplete. Here we show that resveratrol controls breast cancer cell proliferation by inducing tumor-suppressive miRNAs (*miR-34a*, *miR-424*, and *miR-503*) via the p53 pathway and then by suppressing heterogeneous nuclear ribonucleoprotein A1 (*HNRNPA1*), which is associated with tumorigenesis and tumor progression. Notably, *HNRNPA1* was directly regulated by *miR-424* and *miR-503*, the expression of which were mediated by resveratrol. Moreover, we found that resveratrol exerts broad effects on the *HNRNPA1*-related pre-mRNA splicing pathway. Our data provide novel insights into the regulatory roles of resveratrol for preventing and treating of diseases.

## INTRODUCTION

Historically, natural products have been considered invaluable as a source of therapeutic agents. Resveratrol is a polyphenolic phytoalexin present in many plants, including grapevines, pines, peanuts, berries, and grapes [1, 2]. Red wine is made from grapes (most commonly *Vitis vinifera*) that are rich in resveratrol; this has garnered much attention regarding the “French paradox,” which refers to the association between the consumption of red wine and the relatively low incidence of coronary heart disease in the French population, despite their high intake of dietary fat [3]. Numerous studies have examined the effects of resveratrol in detail, including work on sirtuin genes and longevity [4–6]. In addition, it has been suggested that resveratrol possesses potential anti-cancer properties, such as the inhibition of tumor initiation and progression, by inducing cell cycle arrest and apoptosis [7].

microRNAs (miRNAs), a family of small non-coding RNAs, have been identified in various organisms, such as worms, flies, mice, humans, and plants. Several miRNAs are conserved among different species, indicating that these miRNAs may serve important functions such as the modulation of gene expression. It has become realized that miRNAs contribute to many human diseases, including cancers, and are generally dysregulated in cancers compared with their expression in normal tissues. Accordingly, miRNA expression profiles can be used to classify poorly differentiated tumors, and some miRNAs are connected to well-studied tumor-suppressive or oncogenic networks [8, 9]. In addition, many studies have demonstrated that miRNAs are contained in exosomes/extracellular vesicles (EVs); these vesicles, which are secreted from all types of cells, act as mediators of cell-to-cell communication [10]. Furthermore, miRNAs are present in body fluids, such as blood, serum/plasma, saliva, and urine [11]. Researchers are currently investigating the potential utility of miRNA-expression patterns as novel specific and sensitive diagnostic markers, as well as that of miRNA profiles in body fluids as indicators of health conditions [12]. For instance, early-stage breast cancer could be detected and diagnosed by examining the expression profiles of serum miRNAs [13]. Thus, if we could maintain the expression levels and profiles of miRNAs in the body, this strategy would likely support the prevention and treatment of diseases. Recently, resveratrol was shown to regulate miRNAs in some cancer cell lines, which shed new light on cancer biology [14–20]. However, the molecular mechanisms through which resveratrol, a miRNA-regulating natural compound, can contribute to cancer treatment are not completely understood.

A previous study reported that resveratrol induces some tumor-suppressive miRNAs and Argonaute 2 (AGO2) expression, which can result in long-term gene silencing [9]. Here we examined the functional mechanisms underlying the regulatory role of resveratrol by analyzing both mRNA and miRNA profiles in breast cancer cells. Resveratrol up-regulated mainly three miRNAs (*miR-34a*, *miR-424*, and *miR-503*) that are under the control of p53. This was found to lead to the suppression of heterogeneous nuclear ribonucleoprotein A1 (*HNRNPA1*), which is up-regulated in a wide variety of cancers, and to the inhibition of cell proliferation. Our results provide novel information about the regulatory effects of resveratrol regarding the maintenance of good health.

## RESULTS

### Identification of resveratrol-regulated miRNAs in breast cancer

**Figure 1:**
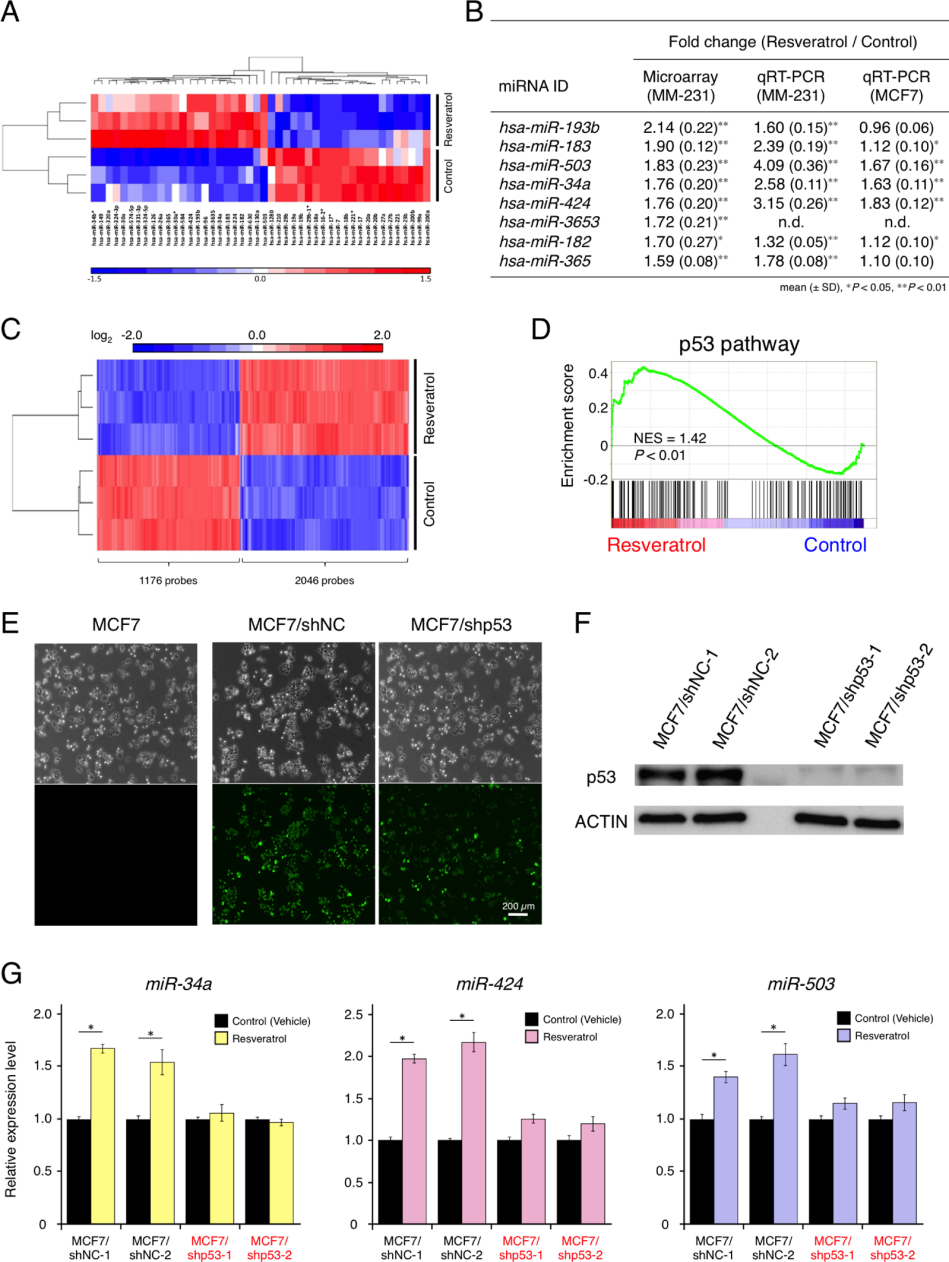
Effects of resveratrol on miRNA and mRNA profiles and resveratrol-regulated miRNAs in breast cancer (**A**) Heat map showing miRNAs whose expression levels differed by >1.2-fold in MDA-MB-231-luc-D3H2LN cells, with or without resveratrol treatment (*P* < 0.1). Twenty-four miRNAs were up-regulated and 22 miRNAs were down-regulated by resveratrol treatment. (**B**) The eight most highly up-regulated miRNAs following resveratrol treatment, as determined by microarray analysis. miRNA expression was validated by qRT-PCR in MDA-MB-231-luc-D3H2LN and MCF7 cells (mean ± SD, ^*^P < 0.05, ^**^P < 0.01). n.d.: not determined (**C**) Heat map showing mRNAs that differed by >1.5-fold in MDA-MB-231-luc-D3H2LN cells, with or without resveratrol treatment (*P* < 0.01). A total of 2,046 mRNAs were up-regulated and 1,176 mRNAs were down-regulated by resveratrol treatment. (**D**) Gene set-enrichment analysis of the p53 pathway signature in MDA-MB-231-luc-D3H2LN cells treated with resveratrol, as compared with control cells (treated with vehicle). NES: normalized enrichment score (**E**) GFP signals in transgenic MCF7 cells expressing control non-target shRNA and TP53-knockdown shRNA. MCF7/shNC: MCF7/H1::non-target shRNA/CMV::copGFP and MCF7/shp53: MCF7/H1::TP53 shRNA/CMV::copGFP. (**F**) Immunoblot analysis of p53 expression in MCF7 cells expressing a control non-target shRNA and p53-knockdown shRNA. (**G**) The expression of resveratrol-regulated miRNAs in p53-knockdown MCF7 cells and control cells, with or without resveratrol treatment (mean ± SEM, ^*^P < 0.05). Two independent clones were prepared for each cell line. The number of data points shown for each sample is four.

A previous study reported that AGO2 up-regulation by resveratrol enhanced the expression of some tumor-suppressive miRNAs and increased its RNA interference activity [15]. However, the understanding of the complete molecular mechanisms by which resveratrol contributes to cancer prevention and treatment is inadequate. For example, it is unclear which miRNAs are mainly regulated by resveratrol and how resveratrol-regulated miRNAs control cancer cell proliferation by modulating target gene expression. To obtain insight into the functional mechanisms of resveratrol, as resveratrol inhibited the proliferation of MDA-MB-231-luc-D3H2LN breast cancer cells (Supplementary Figure 1), we comprehensively analyzed miRNA and mRNA profiles of cancer cells treated with or without resveratrol. miRNA analysis revealed transcriptome changes between resveratrol-treated cells and untreated cells, and that resveratrol induced 24 miRNAs and suppressed 22 miRNAs (Figure 1A and Supplementary Figure 2A). Of these miRNAs, we selected the eight most highly up-regulated miRNAs and validated their expression levels by quantitative reverse transcription-PCR (qRT-PCR; Figure 1B). Additionally, to identify resveratrol-regulated miRNAs that are commonly expressed in different types of cancer cell lines, we examined the eight miRNAs in MCF7 cancer cells. We observed that only three miRNAs (*miR-34a*, *miR-503*, and *miR-424*) were significantly increased by >1.5-fold in both cell lines (Figure 1B); thus, we decided to focus on these miRNAs for further studies.

mRNA analysis suggested that resveratrol regulates a wide variety of genes in treated cells versus untreated cells and affects many biological pathways, such as the p53 pathway and apoptosis. We observed changes in p53-regulated gene-expression levels, which indicated that p53 activation by resveratrol contributed to apoptosis (Figure 1C, 1D, Supplementary Figure 2B–2E, and Supplementary Table 1). Intriguingly, *miR-34a* can be directly transactivated by p53, and *miR-424* and *miR-503* (which are transcribed together as the *miR-424/503* cluster) are presumably under the control of p53 [21–24]. To investigate whether these miRNAs are regulated in the resveratrol/p53 pathway, we established two independent p53-knockdown MCF7 cell lines (Figure 1E and 1F), because MCF7 cells have wild-type p53 [25]. However, MDA-MB-231 cells contain mild mutations in p53 [26]. Resveratrol induced *miR-34a*, *miR-503*, and *miR-424* expression in p53-expressing cells but not in p53-knockdown cells, suggesting that p53 is important for the regulatory function of resveratrol in miRNA induction (Figure 1G). These results showed that, in parallel with inhibiting breast cancer cell proliferation, resveratrol could mainly regulate *miR-34a*, *miR-503*, and *miR-424* via the p53 pathway.

### Identification of the target gene of resveratrol-regulated miRNAs and its clinical significance

**Figure 2:**
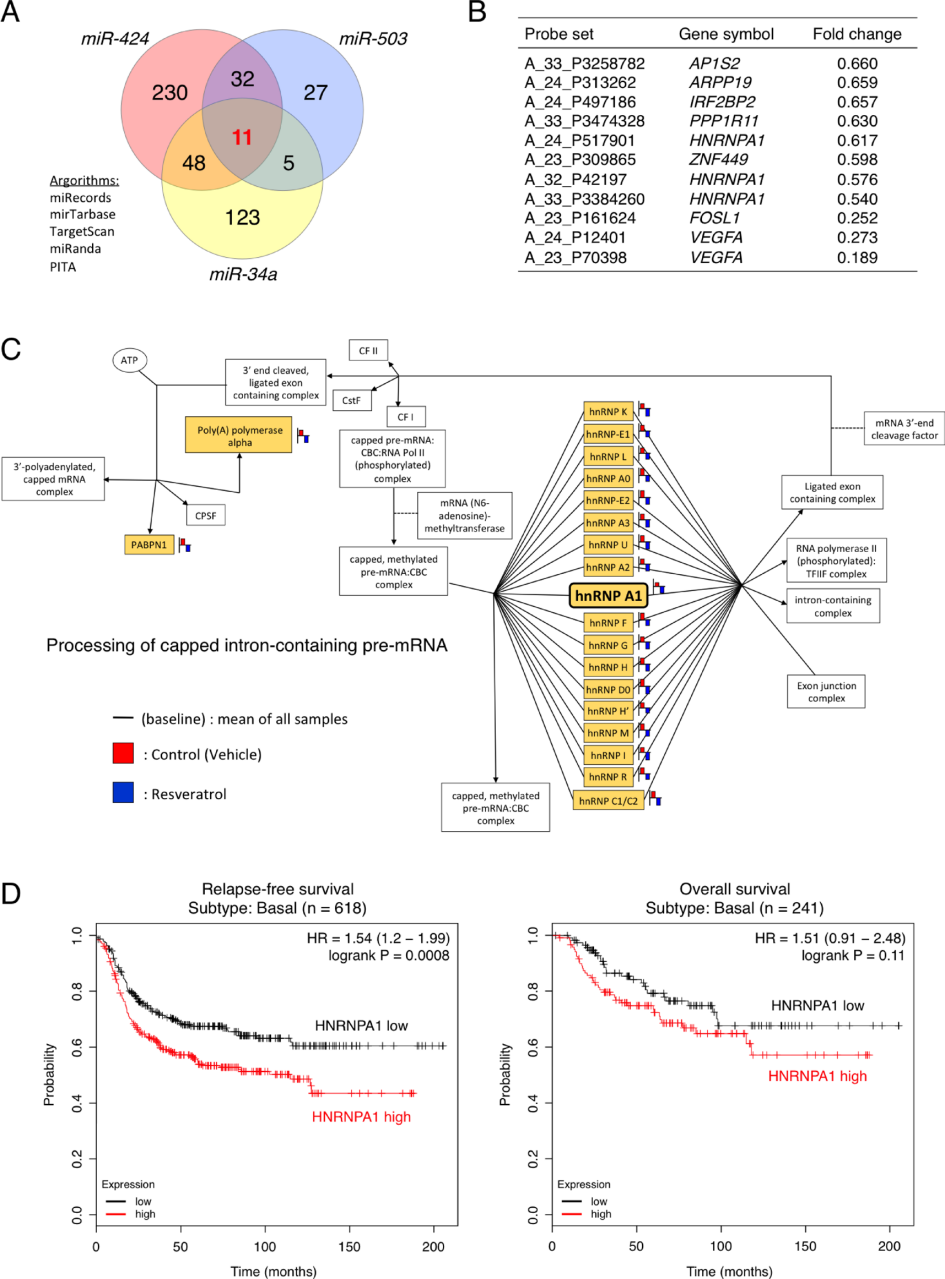
Clinical significance of *HNRNPA1*, a potential target gene of resveratrol-regulated miRNAs, in basal-like breast cancer (**A**) Venn diagram depicting the overlap between predicted *miR-34a*, *miR-424*, and *miR-503* targets. The target prediction algorithms miRecords, miRTarBase, TargetScan, miRanda, and PITA were chosen for whole-transcriptome analysis. (**B**) Common candidates for the putative direct target genes of *miR-34a*, *miR-424*, and *miR-503* (**C**) KEGG pathway analysis of differentially expressed genes in the *HNRNPA1*-related pathway involved in processing capped intron-containing pre-mRNAs. The probe (gene symbol) on the arrays is represented as a yellow color box. (**D**) Left: Kaplan–Meier analysis of correlations between relapse-free survival in patients with basal-like breast cancer exhibiting high (above median value, *n* = 309) and low (below median value, *n* = 309) *HNRNPA1* expression. Right: Kaplan–Meier analysis of correlations between overall survival of patients with basal-like breast cancer exhibiting high (above median value, *n* = 121) and low (below median value, *n* = 120) *HNRNPA1* expression. The *P* value was determined by log-rank test. HR: hazard ratio.

To elucidate the mechanisms through which resveratrol-regulated miRNAs inhibit cancer cell proliferation, instead of searching target genes for each miRNAs, we investigated common targets of all three miRNAs. To this end, we integrated the results of *in silico* analysis using five target-prediction algorithms (miRecords, miRTarBase, TargetScan, miRanda, and PITA) with the transcriptome profile for genes that were down-regulated by >1.5-fold following resveratrol treatment (Figure 2A) [27–32]. Of the 1,176 down-regulated genes, we identified 187, 321, and 75 genes as putative direct target genes of *miR-34a*, *miR-424*, and *miR-503*, respectively. Venn diagram analysis revealed that 11 probe sets, consisting of eight gene symbols, were common candidates for the putative direct target genes of the three miRNAs (*miR-34a*, *miR-424*, and *miR-503*; Figure 2A and 2B). To narrow down the list of potential target genes, we performed gene ontology analysis and KEGG pathway analysis based on the mRNA microarray data. The findings showed that resveratrol treatment affected the *HNRNPA1*-related pathway, which alters the processing of capped intron-containing pre-mRNAs (Figure 2C) [33–35]. *HNRNPA1* is up-regulated in a wide spectrum of cancers, such as breast cancer, colorectal cancer, lung cancer, and glioma [36–39]. These results led us to focus on *HNRNPA1*.

Next, to evaluate the prognostic value of *HNRNPA1* expression in basal-like breast cancer, we investigated the association between *HNRNPA1* expression in breast cancer samples and disease progression (for other types of breast cancer, see Supplementary Figure 3) [40]. Intriguingly, Kaplan–Meier survival analysis demonstrated that patients with high *HNRNPA1* expression had a significantly shorter relapse-free survival than patients with low *HNRNPA1* expression (*P* = 0.0008 by log-rank test) and that patients with high *HNRNPA1* expression had a shorter overall survival than patients with low *HNRNPA1* expression (*P* = 0.11 by log-rank test; Figure 2D). In contrast, among potential target genes of *miR-34a*, *miR-424*, and *miR-503*, vascular endothelial growth factor A (*VEGFA*) and FOS-like 1 (*FOSL1*) expression did not exhibit clear correlations with poor prognosis in patients with basal-like cancer (Supplementary Figure 4). Therefore, the data above showed that, in basal-like breast cancer, *HNRNPA1* was associated with poor clinical outcomes and could serve as a viable target for resveratrol/miRNAs in cancer treatment.

### Resveratrol and resveratrol-regulated miRNAs suppress *HNRNPA1* to mediate cell proliferation

**Figure 3:**
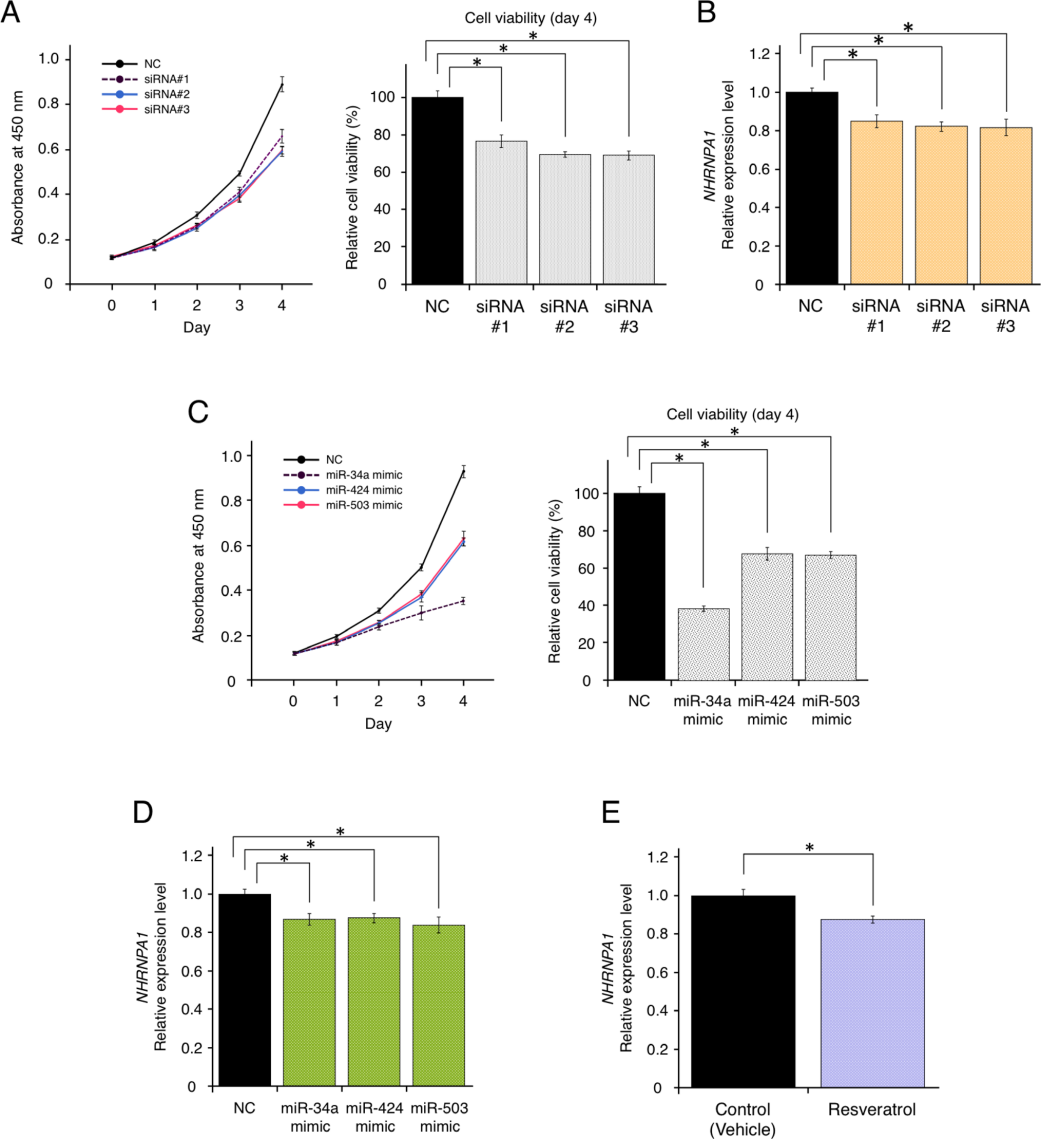
Resveratrol-mediated down-regulation of *HNRNPA1* expression inhibited cancer cell proliferation (**A**) Effects of *HNRNPA1* siRNAs on the proliferation of MDA-MB-231-luc-D3H2LN cells (mean ± SEM). Left: cell viability at 0, 1, 2, 3, and 4 days after siRNA transfection. Right: cell proliferation normalized to that of the negative control (NC) at 4 days (^*^P < 0.05). The number of data points shown for each sample is six. (**B**) Effects of *HNRNPA1* siRNAs on *HNRNPA1* expression (mean ± SEM, ^*^P < 0.05). The number of data points shown for each sample is four. (**C**) Effects of *miR-34a*, *miR-424*, or *miR-503* mimics on the proliferation of MDA-MB-231-luc-D3H2LN cells (mean ± SEM). Left: cell viability at 0, 1, 2, 3, and 4 days after siRNA transfection. Right: cell proliferation normalized to that of the negative control (NC) at 4 days (^*^P < 0.05). The number of data points shown for each sample is six. (**D**) Effects of resveratrol-regulating miRNA (*miR-34a*, *miR-424*, and *miR-503*) mimics on *HNRNPA1* expression (mean ± SEM, ^*^P < 0.05). The number of data points shown for each sample is four. (**E**) Effects of resveratrol on *HNRNPA1* expression (mean ± SEM, ^*^P < 0.05). The number of data points shown for each sample is four.

As demonstrated above, *HNRNPA1* could be highly relevant to cancer progression. However, it remained unclear whether *HNRNPA1* expression is correlated positively or negatively with cell proliferation, miRNA expression, and resveratrol treatment. To determine the functional effects of *HNRNPA1* on cell proliferation of basal-like breast cancer cells (MDA-MB-231-luc-D3H2LN cells), we knocked down *HNRNPA1* using three independent siRNAs. siRNA transfection significantly reduced *HNRNPA1* expression and cell proliferation (Figure 3A and 3B). This result suggested that *HNRNPA1* promoted cancer cell proliferation and that suppressing *HNRNPA1* expression inhibited cell proliferation.

Furthermore, we investigated whether resveratrol-regulated miRNAs (*miR-34a*, *miR-424*, and *miR-503*) and resveratrol could affect *HNRNPA1* expression. After transfection with each miRNA mimic or non-target negative control miRNA mimic, we confirmed that overexpression of these miRNAs inhibited cell proliferation, demonstrating their effects as tumor-suppressive miRNAs (Figure 3C). We also observed that transfection of each miRNA significantly reduced *HNRNPA1* expression (Figure 3D). Importantly, resveratrol treatment itself could down-regulate *HNRNPA1* expression (Figure 3E). Taken together, these data suggested that the axis of resveratrol and resveratrol-regulated miRNAs functionally control the expression of *HNRNPA1*, which is involved in cancer cell proliferation.

### Direct or indirect regulation of *HNRNPA1* by resveratrol-regulated miRNAs

**Figure 4:**
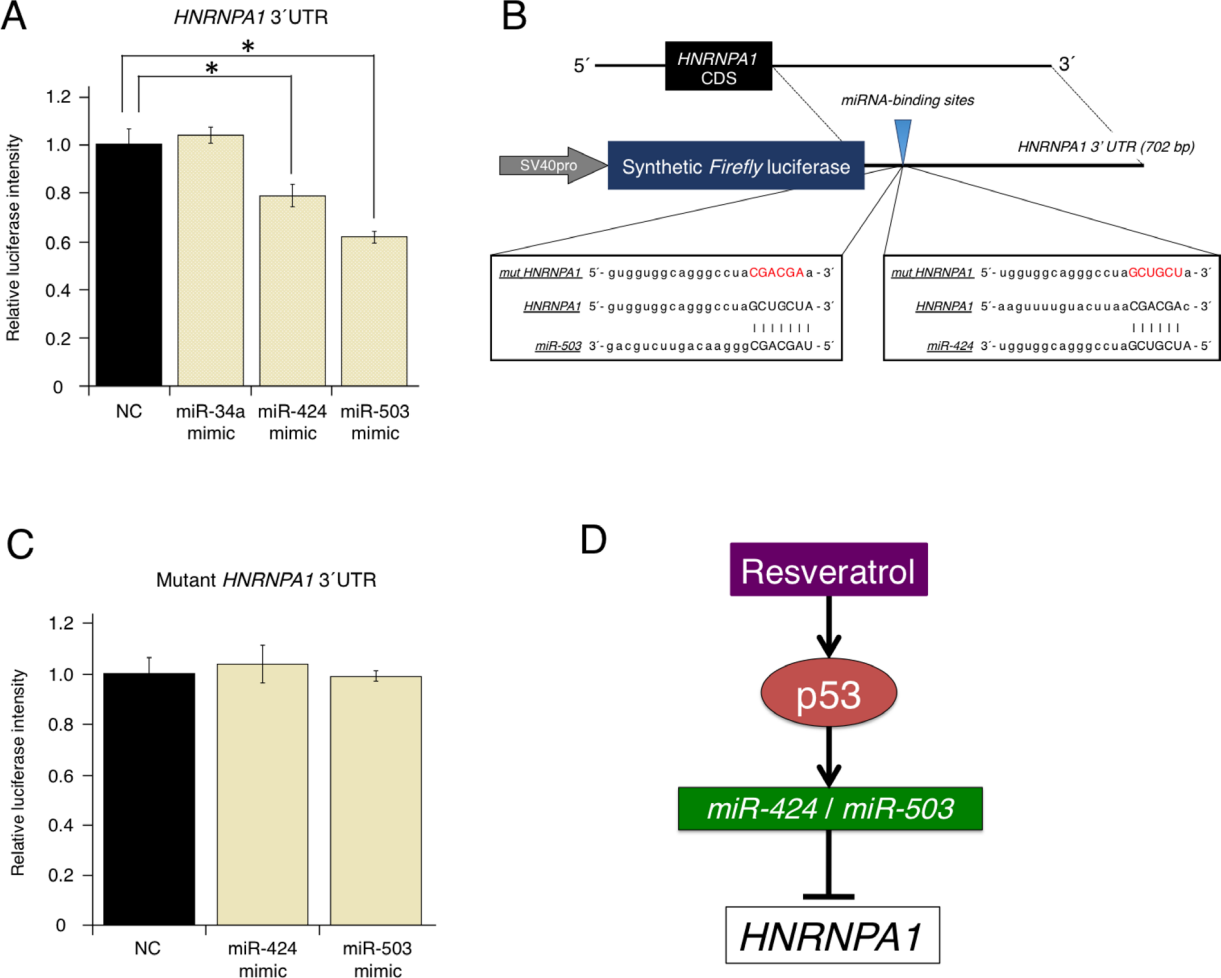
Direct regulation of oncogenic *HNRNPA1* by the tumor-suppressive miRNAs, *miR-424* and *miR-503* (**A**) Effects of overexpressing resveratrol-regulated miRNA (*miR-34a*, *miR-424*, and *miR-503*) on WT-*HNRNPA1* 3′-UTR luciferase activity. *Firefly* luciferase activities were normalized to *Renilla* luciferase activities (mean ± SEM, ^*^P < 0.05). Number of data points per sample was four. (**B**) Diagram of the luciferase reporter containing the WT and mutant *HNRNPA1* 3′-UTR regions. Sequences of miRNAs (*miR-424* or *miR-503*), the miRNA-binding site, and the mutated site in the 3′-UTR of *HNRNPA1* are shown. (**C**) Effects of overexpressing resveratrol-regulated miRNA (*miR-424* and *miR-503*) on mutant *HNRNPA1* 3′-UTR luciferase activity. *Firefly* luciferase activities were normalized to *Renilla* luciferase activities (mean ± SEM, ^*^P < 0.05). The number of data points shown for each sample is four. (**D**) Schematic representation of the molecular mechanisms through which resveratrol inhibits cancer cell proliferation via the p53/miRNA/HNRNPA1 axis.

As shown in Figure 2A, using the target-prediction algorithms, *HNRNPA1* was selected as a putative direct target of resveratrol-regulated miRNAs (*miR-34a*, *miR-424*, and *miR-503*). To elucidate the mechanism through which *miR-34a*, *miR-424*, and *miR-503* regulate *HNRNPA1*, we prepared a luciferase reporter vector in which the 3′-untranslated region (UTR) of *HNRNPA1* was cloned downstream of a luciferase gene (wild-type [WT]-*HNRNPA1* 3′-UTR vector). When the luciferase reporter WT-*HNRNPA1* 3′-UTR vector was co-transfected with *miR-34a*, *miR-424*, or *miR-503* mimics, luciferase activity was significantly reduced by *miR-424* and *miR-503* mimics but not by the *miR-34a* mimic (Figure 4A). This result indicated that *miR-34a* could regulate *HNRNPA1* without directly binding to the *HNRNPA1* 3′-UTR.

We next prepared a mutant luciferase reporter (mut-*HNRNPA1* 3′-UTR vector) in which the putative binding site of *miR-424* and *miR-503* was changed (Figure 4B). In contrast to the WT-*HNRNPA1* 3′-UTR vector, when the mut-*HNRNPA1* 3′-UTR vector was used, mutations in the binding site abolished inhibition by *miR-424* and *miR-503* mimics (Figure 4C). These results strongly suggested that *HNRNPA1* expression could be regulated directly by *miR-424* and *miR-503*, but indirectly by *miR-34a* (Figure 4D).

## DISCUSSION

It has long been considered that the consumption of healthy foods prevents or cures illness, whereas it has been shown that unhealthy eating habits are associated with increased risks for developing certain diseases, including cancer [41–44]. The therapeutic potential of naturally occurring products has attracted much attention in the past decade. Resveratrol, a multi-functional polyphenolic phytoalexin, has been known to exert anti-tumorigenic and anti-inflammatory effects, since it was demonstrated to inhibit carcinogenesis in mice [45]. Recently, it has been unveiled that some naturally occurring compounds, including resveratrol, regulate the expression of miRNAs in a favorable manner, which opened new avenues for cancer prevention and treatment. In breast cancer, some data have shown that resveratrol induces the expression of *miR-663* and *miR-744* in MCF7 cells and *miR-200c* and *miR-141* in MDA-MB-231-luc-D3H2LN cells [14, 15]. However, it is still unclear how resveratrol controls cancer cell growth by regulating miRNA expression for the prevention of diseases. Here we showed that resveratrol controlled breast cancer cell proliferation by inducing tumor-suppressive miRNAs (*miR-34a*, *miR-424*, and *miR-503*) via the p53 pathway, which in turn suppressed *HNRNPA1*–the expression of which is associated with tumorigenesis and tumor progression.

*miR-34a* is a direct target of p53 and is known to promote cell cycle arrest in the G_1_ phase, induce senescence, and enhance apoptosis by directly repressing CDK4, CDK6, cyclin E2, E2F transcription factor 3 (E2F3), c-Myc, and BCL-2 [21–23, 46, 47]. Moreover, it was observed that resveratrol controls *miR-34a* expression in colon cancer and glioma cells [20, 48]. In breast cancer cells, consistent with previous studies, we showed that resveratrol induced *miR-34a*, suggesting that resveratrol could exert anticancer effects. *miR-424* and *miR-503*, which are co-transcribed as the *miR-424/503* cluster, are thought to belong to the extended *miR-16* family based on the presence of the AGCAGC sequence in the 5′-end of the miRNA [49]. *miR-16* has been identified as a tumor-suppressive miRNA and is deleted and/or downregulated in approximately 68% of cases of B-cell chronic lymphocytic leukemia, similar to results in many other cancers, strongly suggesting its important functions in tumor formation [50–54]. The seed sequences of *miR-424* and *miR-503* are similar to that of *miR-16*, indicating that they share target genes with *miR-16* and act as tumor suppressors. Intriguingly, recent work in knockout mice has demonstrated that the *miR-424*/*503* cluster is commonly lost in a subset of aggressive breast cancers, which promotes breast tumorigenesis *in vivo* [55]. These findings have provided evidence demonstrating that *miR-424* and *miR-503* act as tumor suppressors in breast cancer.

In this study, we found that resveratrol regulated the tumor-suppressive miRNAs *miR-424* and *miR-503* through the p53 pathway, in combination with *miR-34a*. These results support the therapeutic potential of resveratrol. Previous computational predictions suggested that various transcription factors, including runt-related transcription factor 1 (RUNX1), E2F3, SP3, yin yang 1 (YY1), nuclear factor erythroid 2-like 2 (NFE2L2), cAMP responsive element binding protein 1 (CREB1), activating transcription factor 2 (ATF2), upstream transcription factor 2 (USF2), ETS domain-containing protein (ELK1), CCAAT/enhancer binding protein beta (CEBPB), and homeobox A4 (HOXA4), can interact with the promoter region of *miR-424/503* cluster genes [56]. Our data indicated that *miR-424* and *miR-503* expression was regulated by p53 in breast cancer; however, we have yet to elucidate whether these miRNAs were direct or indirect targets of p53.

Although we mainly focused on resveratrol-induced miRNAs in this research, it is also important to cast a spotlight on miRNAs that were decreased by resveratrol treatment. For example, some oncogenic miRNAs were down-regulated by resveratrol, such as *miR-17*, miR-17*, *miR-18a*, *miR-19a*, *miR-19b*, and *miR-20a* (*miR-17-92* cluster). These miRNAs are transcriptionally repressed to varying degrees by p53 under normal conditions but, for instance, are up-regulated in human B-cell lymphoma and are amplified in malignant lymphoma [47, 57, 58]. Consistent with results showing that resveratrol controls tumor-suppressive miRNAs (*miR-34a*, *miR-424*, and *miR-503*) via the p53 pathway, p53-regulated oncogenic miRNAs were down-regulated by resveratrol, which suggests that such down-regulation could contribute to the inhibition of cancer cell growth and proliferation. It is reasonable to expect that balancing the expression of tumor-suppressive and oncogenic miRNAs is critical for its efficacy. Our results suggested that resveratrol could control tumor-suppressive and oncogenic miRNAs in a positive manner, but more studies are needed to fully understand these mechanisms.

*HNRNPA1* is linked to tumorigenesis and tumor progression and has been shown to be up-regulated in a wide spectrum of cancers, such as breast cancer, colorectal cancer, lung cancer, and glioma [36–39]. It has been known that HNRNPA1 plays an important role in the modulation of gene expression by properly controlling the splicing of a wide variety of gene transcripts. Because numerous transcripts are targeted by HNRNPA1, it is currently difficult to determine which known or unknown target transcripts mainly mediate downstream cell growth and proliferation that is inhibited by resveratrol.

The mRNA transcripts of several genes, such as breast cancer 1 (*BRCA1*) and pyruvate kinase M (*PKM*), have been experimentally demonstrated to be alternatively spliced by HNRNPA1 [60]. As a tumor-suppressor gene, *BRCA1* plays a pivotal role in cell-cycle regulation and DNA damage repair, and its defects result in defective cell-cycle checkpoints and genome instability [61]. Dysregulations of HNRNPA1 can lead to aberrant exon-skipping of *BRCA1* transcripts and impair its function in cell-cycle regulation [62, 63]. Regarding *PKM*, the oncogenic transcription factor c-Myc promotes the expression of three HNRNP proteins (hnRNPI, hnRNPA1, and hnRNPA2), resulting in maintaining a high level of the embryonic isoform of PKM containing exon 10 (PKM2), as compared with the adult isoform of PKM containing exon 9 (PKM1) [39]. Because PKM2 plays an important role as a promoter of the Warburg effect, its overexpression is likely to be universal in many tumors and it is expected to be critical for cancer cell proliferation [59]. Although, after the treatment of resveratrol, it remains uncovered how the downstream genes of HNRNPA1 contribute to the phenotype observed in this research, aberrant expression of *HNRNP* genes, including *HNRNPA1*, has an impact on the regulation of pre-mRNA alternative splicing in cancer, suggesting that the maintenance of *HNRNP* levels is important for cancer prevention and treatment.

In this study, we focused on *HNRNPA1* as a common target of resveratrol-regulated miRNAs (*miR-34a*, *miR-424*, and *miR-503*). As described above, *HNRNPA1* could serve as a therapeutic target in breast cancer, consistent with the Kaplan–Meier analysis showing that high *HNRNPA1* levels were associated with poor clinical outcomes in basal-like breast cancer. We also showed that *HNRNPA1* expression was inhibited by resveratrol and its regulated miRNAs, and that *HNRNPA1* is a new direct target of *miR-424* and *miR-503*. These data shed new light on the molecular mechanisms through which resveratrol contributes to the prevention and treatment of cancer. Although *miR-34a* indirectly suppressed *HNRNPA1*, it directly represses key genes required for promoting cell-cycle arrest in the G_1_ phase, senescence, and apoptosis [64]. Because of the existence of many potential *miR-34a* target genes, we could not elucidate the relationship between the downstream genes and HNRNPA1, although this will be an important subject of future studies.

Notably, KEGG pathway analysis showed that resveratrol could exert broad effects on the *HNRNPA1*-related pre-mRNA splicing pathway. However, it is currently difficult to determine how resveratrol affects splicing regulation and sequential changes in gene expression. It will be of interest to examine whether resveratrol can regulate its target miRNAs (*miR-34a*, *miR-424*, and *miR-503*) and *HNRNPA1* expression in other types of cancer. For therapeutic applications, future investigations will provide important information regarding the regulatory mechanisms of resveratrol.

For many years, a variety of studies have identified relationships between food and health, demonstrating the significance of a well-balanced diet for good health. Moreover, there is the possibility to prevent diseases and maintain good health if the expression levels and profiles of miRNAs in the body are appropriately maintained, as supported by the observation that changes in miRNA profiles in the bloodstream can be detected during the early stages of disease [20, 65]. It is particularly intriguing how foods and natural compounds substantially impact the expression of various miRNAs in the body, leading to the prevention of diseases. Thus, we believe that research and development will maintain or improve human health worldwide.

## MATERIALS AND METHODS

### Cell culture

MDA-MB-231-luc-D3H2LN cells (Xenogen) and MCF7 cells (American Type Culture Collection) were cultured in RPMI 1640 medium supplemented with 10% heat-inactivated fetal bovine serum and 1% antibiotic-antimycotic (Thermo Fisher Scientific) at 37° C in 5% CO_2_. Trans-resveratrol (Cayman Chemical) was used at concentrations ranging from 12.5–50 mM.

### Cell proliferation assay (MTS assay)

Cells were seeded in 96-well plates at a density of 5,000 cells/well. The following day, the cells were treated with resveratrol. After 3 days in culture, cell viability was measured using the Cell Counting Kit-8 (Dojindo Laboratories) according to the manufacturer’s instructions. The absorbance at 450 nm was measured with a Synergy H4 microplate reader (BioTek Instruments). Experiments were performed in at least triplicate.

### Expressing short-hairpin RNAs (shRNAs) via lentiviral transduction

Cell lines stably expressing a p53-specific shRNA (shp53) or a non-targeting control shRNA (shNC) were established by transducing cells with pLKO.1 lentiviral vectors encoding either shRNA, both of which were purchased from Sigma. The human *p53* shRNA target sequence was as follows: 5′-GACUCCAGUGGUAAUCUAC-3′. The control target shRNA sequence was as follows: 5′-GAAAUGUACUGCGCGUGGAGAC-3′. In knockdown experiments, 293T cells were transduced with constructs expressing shp53 or shNC. MCF7 cells were infected with recombinant lentiviruses expressing shp53 or shNC.

### Immunoblot analysis

Two days after resveratrol treatment, whole cell lysates were obtained using the M-PER mammalian protein-extraction reagent (Thermo Fisher Scientific). The proteins were resolved in a Mini-PROTEAN TGX Gel (4–12%, Bio-Rad) and electrotransferred onto a polyvinylidene fluoride membrane (Millipore). After blocking in Blocking One solution (Nacalai Tesque), the membranes were incubated for 1 h at room temperature with primary antibodies against p53 (sc-126; diluted 1:5000; Santa Cruz Biotechnology) and actin (MAB1501, diluted 1:5000; Millipore). The secondary antibody (horseradish peroxidase-conjugated anti-mouse IgG, NA931; GE Healthcare) was used at a dilution of 1:5000. The membranes were then exposed to ImmunoStar LD (Wako). The bound antibodies were visualized by chemiluminescence using the ECL Plus Western blotting detection system (GE HealthCare), and the signals were detected with a LAS-3000 LuminoImager (Fuji Film).

### qRT-PCR analysis

Total RNA and miRNA were extracted from cells cultured with or without resveratrol treatment using the QIAzol and miRNeasy Mini Kit (Qiagen) according to the manufacturer’s protocol. cDNA was generated using the PrimeScript RT reagent Kit (TaKaRa) and the TaqMan^®^ MicroRNA Reverse Transcript Kit (Applied Biosystems). TaqMan^®^ probes (TaqMan^®^ Gene Expression Assays and TaqMan^®^ MicroRNA Assays) were purchased from Applied Biosystems. qRT-PCR reactions were performed with the TaqMan probes using the Premix Ex Taq™ (Probe qPCR) (TaKaRa) and TaqMan™ Universal PCR Master Mix, no AmpErase™ UNG (Thermo Fisher Scientific) on the StepOne^®^ Real-Time PCR system (Applied Biosystems). Experiments were performed in at least triplicate. Expression levels of genes of interest were normalized to the expression of *GAPDH* and *RNU44B*.

### Microarray analyses

For mRNA microarray analysis, total RNA (50 ng) was extracted using the QIAzol reagent (Qiagen) from MDA-MB-231-luc-D3H2LN cells, with or without exposure to 25 µM resveratrol for 2 days, and purified using the RNeasy Micro Kit (Qiagen). SurePrint G3 Human GE 8 × 60 K arrays (Agilent Technologies) were hybridized with cyanine 3 (Cy3)-labeled cRNA targets prepared from the RNA samples, according to the manufacturer’s instructions. Experiments were performed in biological triplicate.

For miRNA microarray analysis, total RNA (100 ng) was extracted using the QIAzol reagent (Qiagen) from MDA-MB-231-luc-D3H2LN cells, with or without exposure to 25 µM resveratrol for 2 days, and purified using the RNeasy Micro Kit (Qiagen). SurePrint G3 Human miRNA 8 × 60 K arrays (Agilent Technologies) were hybridized with 5′-cytidine bisphosphate-Cy3 (pCp-Cy3)-labeled cRNA targets prepared from the RNA samples according to the manufacturer’s instructions. Experiments were performed in biological triplicate. Each of the arrays was analyzed with the Microarray Scanner (Agilent Technologies). Gene-expression levels were calculated using Feature Extraction software, version 10.7.3.1 (Agilent Technologies). Normalized and log-transformed intensity values were analyzed using GeneSpring GX software, version 7.3.1 (Agilent Technologies). The intensity values were log_2_-transformed and imported into the Partek Genomics Suite 6.6 (Partek Incorporated, USA). One-way analysis of variance (ANOVA) was performed to identify differentially expressed genes. Unsupervised clustering and heat map generation were performed with sorted data sets by the Euclidean distance based on average linkage clustering, and principal component analysis mapping was conducted for all probe sets using Partek Genomics Suite 6.6. Gene set-enrichment analysis (http://software.broadinstitute.org/gsea/index.jsp) was performed to compare resveratrol-treated samples and control samples.

### Transient assays

For siRNA transfections, pre-designed siRNAs targeting *HNRNPA1* mRNA (IDs: s6710, s6712, and s223860) were purchased from Applied Biosystems. The *Silencer*^®^ Select Negative Control #1 siRNA (Applied Biosystems) was used as a negative control. For miRNA transfections, synthetic hsa-miR-34a-5p (ID: MC11030), hsa-miR-424-5p (ID: MC10306), and hsa-miR-503-3p (ID: MC10378) were purchased from Applied Biosystems. The mirVana™ miRNA Mimic, Negative Control #1 (Applied Biosystems) was used as a negative control. Transfections were performed using the DharmaFECT transfection reagent (Thermo Fisher Scientific) according to the manufacturer’s protocol. A final concentration 25 nM in the supernatant was used when transfecting siRNAs or miRNAs. Experiments were performed in at least triplicate.

### Luciferase reporter assays

For 3′-UTR luciferase reporter assays, pEZX-MT01 dual luciferase reporter plasmids containing the 3′-UTR of *HNRNPA1*, or the mutated 3′-UTR of *HNRNPA1*, as well as a control plasmid lacking the no 3′-UTR were purchased from GeneCopoeia. MCF7 cells were seeded into a 96-well plate at a density of 1.5 × 10^4^ cells/well and co-transfected with the 100 ng of a vector and the 50 nM of a mirVana™ miRNA mimic or the mirVana™ miRNA inhibitor Negative Control (Thermo Fisher Scientific), using the DharmaFECT Duo transfection reagent (Thermo Fisher Scientific) for 24 h. The luciferase assays were performed using the Dual-Glo Luciferase assay system (Promega) according to the manufacturer’s instructions. Experiments were performed in at least triplicates.

### Survival analysis

Overall survival in patients with basal-type, luminal A, luminal B, or HER2-positive breast cancer was stratified according to expression of the gene of interest and is presented as Kaplan–Meier plots (http://www.kmplot.com) [40].

### Statistical analysis

The data are presented as the means ± SEM of at least three independent experiments. Statistical analyses of data containing more than two groups were performed using one-way ANOVA followed by Tukey’s honest significant difference test to account for multiple comparisons. *P* < 0.05 was considered statistically significant.

## SUPPLEMENTARY MATERIALS FIGURES AND TABLE




