# Supplementary material for: Regulatory role of resveratrol, a microRNA-controlling compound, in HNRNPA1 expression, which is associated with poor prognosis in breast cancer
Source: Oncotarget. 2018 May 15;9(37):24718–30. doi: 10.18632/oncotarget.25339 (PMC5973863; doi:10.18632/oncotarget.25339)
Supplement: Supplementary file 1 [file oncotarget-09-24718-s001.pdf]

Regulatory role of resveratrol, a microRNA-controlling compound, in *HNRNPA1* expression, which is associated with poor prognosis in breast cancer

SUPPLEMENTARY MATERIALS

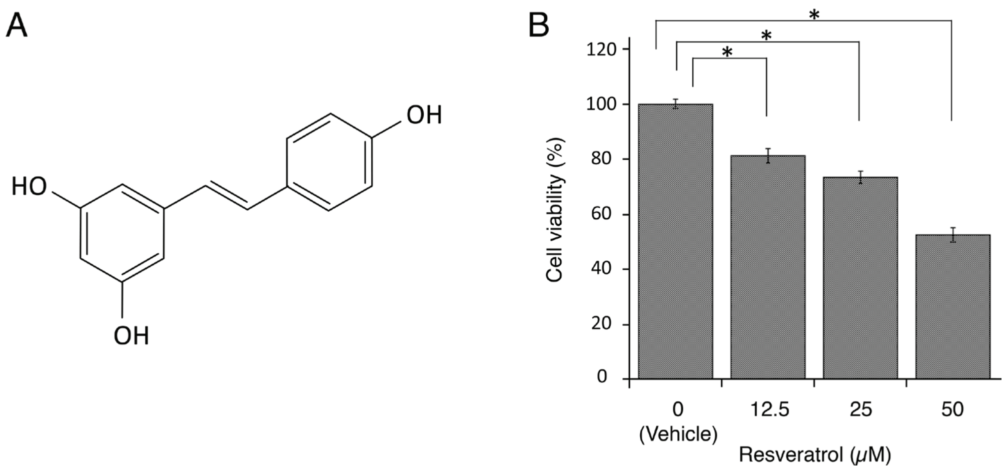

Supplementary Figure 1: Effect of resveratrol on breast cancer cell proliferation. (**A**) Structural formula of resveratrol. (**B**) Cell viability of MDA-MB-231-luc-D3H2LN cells treated with resveratrol ($^{*}P < 0.05$).

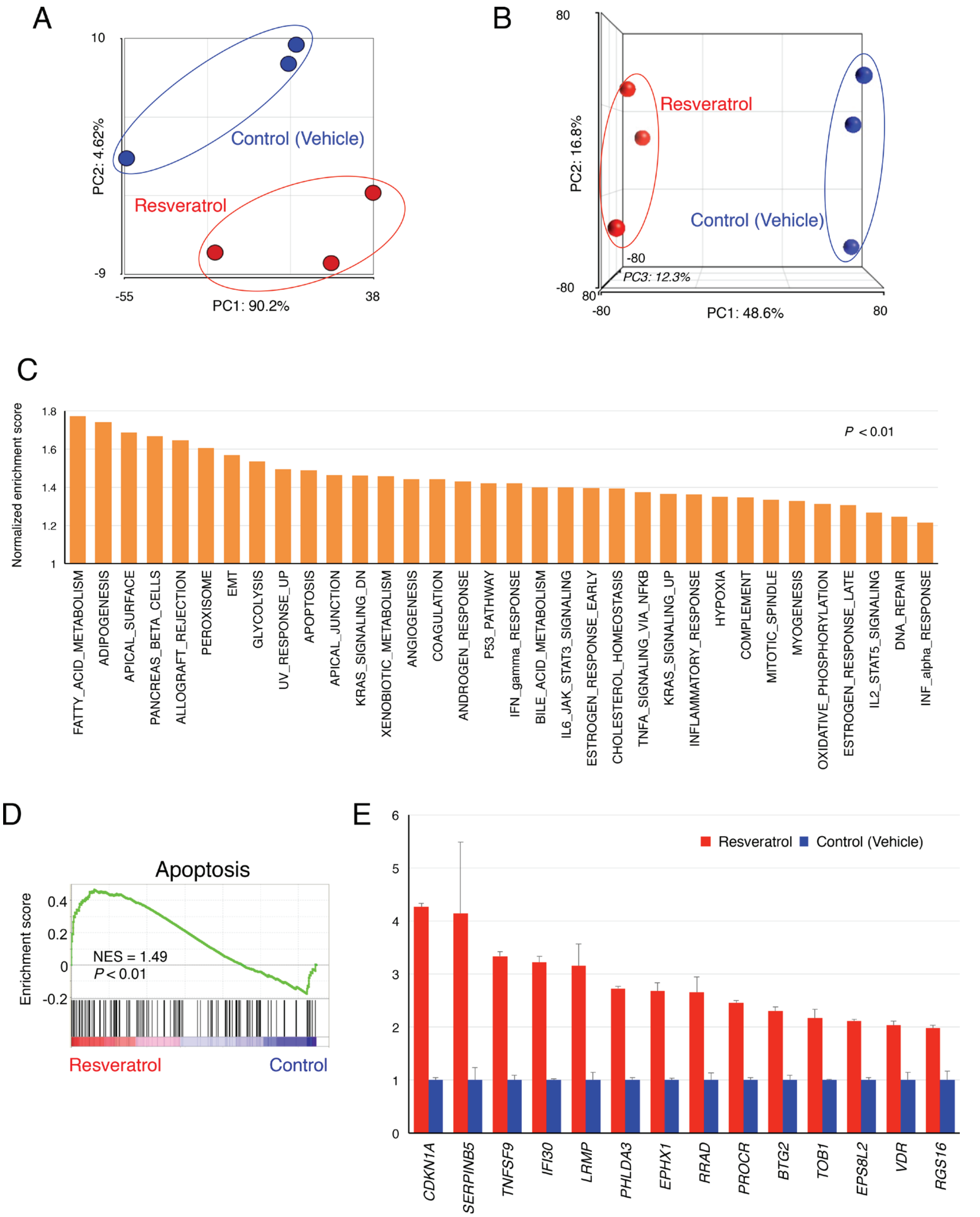

Supplementary Figure 2: Effect of resveratrol on mRNA and miRNA transcritomes. (**A**) Principle component analysis (PCA) of miRNA levels in MDA-MB-231-luc-D3H2LN cells treated with resveratrol (Resveratrol) versus without resveratrol (Control). Number of data per group was three. (**B**) PCA of mRNA levels in MDA-MB-231-luc-D3H2LN cells treated with resveratrol (Resveratrol) versus without resveratrol (Control). The number of data points shown for each sample is three. (**C**) Gene set-enrichment analysis (GSEA) of gene sets in MDA-MB-231-luc-D3H2LN cells with the treatment of resveratrol, as compared to control (vehicle). (**D**) GSEA of the apoptosis pathway signature in MDA-MB-231-luc-D3H2LN cells with the treatment of resveratrol, as compared to control (vehicle). NES: normalized enrichment score. (**E**) Changes in gene expression related to p53 and apoptosis pathways with resveratrol treatment (mean ± SEM). The number of data points shown for each sample is three.

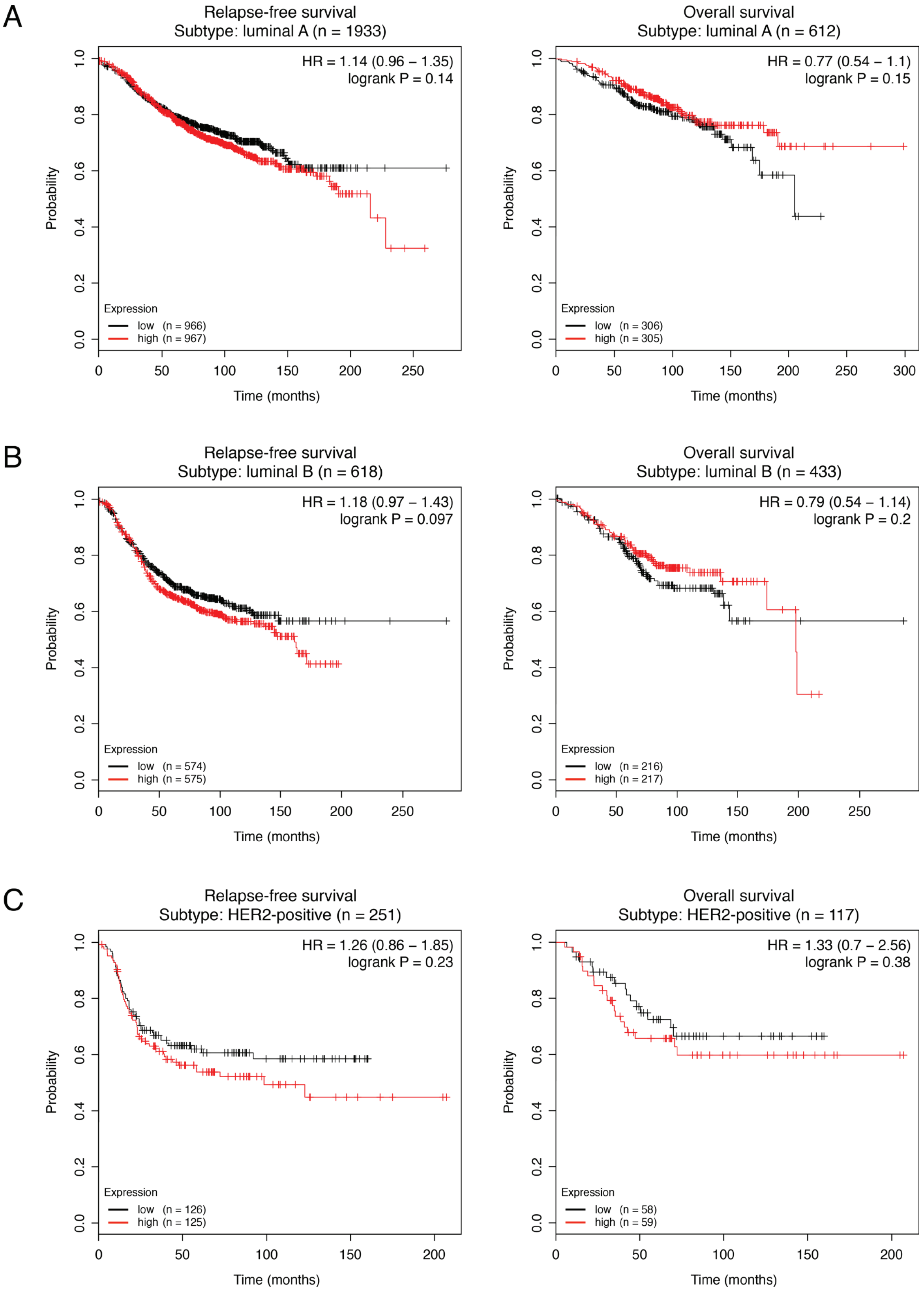

Supplementary Figure 3: Clinical significance of *HNRNPA1* in various intrinsic breast cancer subtypes (luminal A, luminal B, and HER2-positive). Left: Kaplan–Meier analysis of correlations between the relapse-free survival of luminal A (**A**), luminal B (**B**), or HER2-positive (**C**) breast cancer patients with high (above median value) and low (below median value) *HNRNPA1* levels. Right: Kaplan–Meier analysis of correlations between the overall survival of luminal A (A), luminal B (B), or HER2-positive (C) breast cancer patients with high (above median value) and low (below median value) *HNRNPA1* levels. *P* values were determined by log-rank test. HR: hazard ratio.

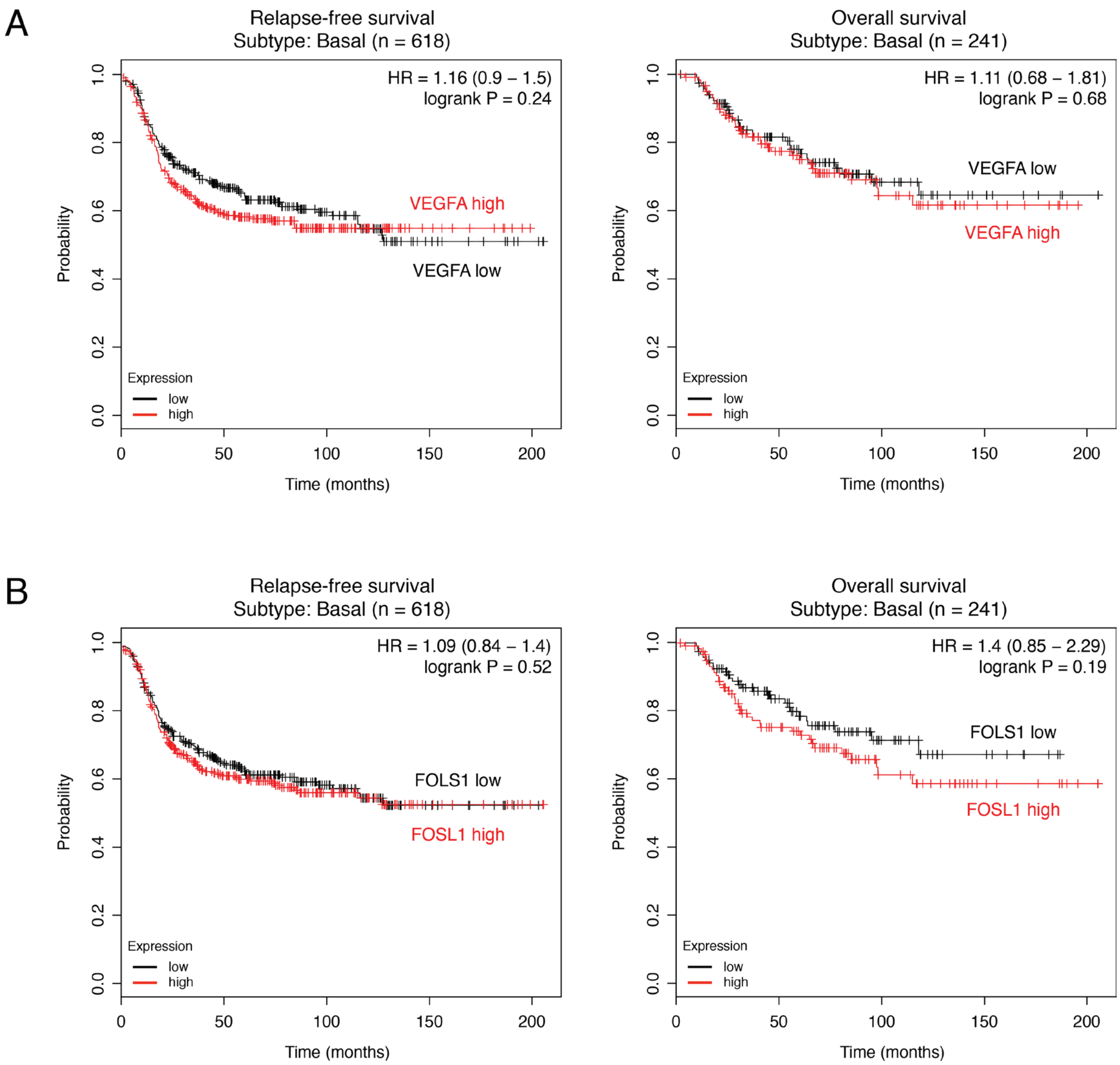

Supplementary Figure 4: Clinical significance of *VEGFA* and *FOSL1*, potential target genes of the resveratrol-regulating miRNAs, in basal-like breast cancer. (**A**) Left: Kaplan–Meier analysis of correlation between relapse-free survival of basal-like breast cancer patients with high (above median value, $n = 309$) and low (below median value, $n = 309$) *VEGFA* levels. Right: Kaplan–Meier analysis of correlation between overall survival of basal-like breast cancer patients with high (above median value, $n = 121$) and low (below median value, $n = 120$) *VEGFA* levels. (**B**) Left: Kaplan–Meier analysis of correlation between relapse-free survival of basal-like breast cancer patients with high (above median value, $n = 309$) and low (below median value, $n = 309$) *FOSL1* levels. Right: Kaplan–Meier analysis of correlation between overall survival of basal-like breast cancer patients with high (above median value, $n = 121$) and low (below median value, $n = 120$) *FOSL1* levels. *P* values were determined by log-rank test. HR: hazard ratio.

Supplementary Table 1: List of genes changed by >1.5-fold with resveratrol treatment. See Supplementary_Table_1
