# Supplementary material for: Regulatory role of resveratrol, a microRNA-controlling compound, in HNRNPA1 expression, which is associated with poor prognosis in breast cancer
Source: Oncotarget. 2018 May 15;9(37):24718–30. doi: 10.18632/oncotarget.25339 (PMC5973863; doi:10.18632/oncotarget.25339)
Supplement: Supplementary file 2 [file oncotarget-09-24718-s002.docx]

| **Supplementary Table 1: List of genes changed by >1.5-fold with resveratrol treatment.** | | | | |
| --- | --- | --- | --- | --- |
| Column ID | GeneName | SystematicName | Fold change (Resveratrol / Control) | *P* value |
| A_33_P3290343 | CYP1B1 | NM_000104 | -43.30 | 1.00E-04 |
| A_23_P209625 | CYP1B1 | NM_000104 | -35.41 | 9.89E-05 |
| A_23_P309381 | HIST2H2AA4 | NM_001040874 | -13.49 | 1.16E-05 |
| A_33_P3253214 | ENST00000369161 | ENST00000369161 | -12.25 | 4.25E-06 |
| A_23_P114626 | SERPINC1 | NM_000488 | -12.05 | 6.59E-06 |
| A_23_P5903 | SLCO4A1 | NM_016354 | -11.47 | 6.09E-05 |
| A_23_P59045 | HIST1H2AE | NM_021052 | -11.12 | 9.95E-06 |
| A_23_P428184 | HIST1H2AD | NM_021065 | -9.27 | 5.68E-06 |
| A_23_P205370 | ASB2 | NM_016150 | -8.89 | 4.60E-04 |
| A_24_P260639 | HIST1H1D | NM_005320 | -8.82 | 1.09E-05 |
| A_33_P3290780 | IL24 | NM_001185156 | -8.55 | 1.11E-04 |
| A_33_P3404989 | HIST1H3H | NM_003536 | -8.55 | 9.85E-07 |
| A_23_P114626 | SERPINC1 | NM_000488 | -8.25 | 1.70E-04 |
| A_23_P114626 | SERPINC1 | NM_000488 | -8.22 | 6.12E-04 |
| A_32_P24376 | LOC730755 | NM_001165252 | -7.91 | 5.30E-04 |
| A_23_P114626 | SERPINC1 | NM_000488 | -7.89 | 2.23E-05 |
| A_23_P114626 | SERPINC1 | NM_000488 | -7.61 | 7.33E-04 |
| A_24_P250922 | PTGS2 | NM_000963 | -7.57 | 8.25E-04 |
| A_24_P245379 | SERPINB2 | NM_002575 | -7.48 | 9.84E-04 |
| A_24_P250922 | PTGS2 | NM_000963 | -7.48 | 2.07E-04 |
| A_23_P167983 | HIST1H2AC | ENST00000314088 | -7.32 | 9.03E-05 |
| A_24_P250922 | PTGS2 | NM_000963 | -6.92 | 8.42E-05 |
| A_32_P55241 | SHISA2 | NM_001007538 | -6.91 | 1.29E-04 |
| A_23_P355439 | HIST1H2AA | NM_170745 | -6.85 | 1.14E-05 |
| A_24_P68631 | HIST2H2AB | NM_175065 | -6.82 | 3.04E-06 |
| A_24_P250922 | PTGS2 | NM_000963 | -6.82 | 2.17E-04 |
| A_23_P114626 | SERPINC1 | NM_000488 | -6.63 | 5.95E-04 |
| A_23_P104318 | DDIT4 | NM_019058 | -6.58 | 8.51E-06 |
| A_23_P122443 | HIST1H1C | NM_005319 | -6.54 | 2.55E-06 |
| A_23_P104318 | DDIT4 | NM_019058 | -6.50 | 5.73E-06 |
| A_24_P250922 | PTGS2 | NM_000963 | -6.49 | 5.73E-04 |
| A_23_P104318 | DDIT4 | NM_019058 | -6.46 | 8.94E-06 |
| A_24_P250922 | PTGS2 | NM_000963 | -6.46 | 3.40E-05 |
| A_23_P104318 | DDIT4 | NM_019058 | -6.45 | 1.95E-05 |
| A_23_P104318 | DDIT4 | NM_019058 | -6.43 | 7.59E-06 |
| A_23_P114626 | SERPINC1 | NM_000488 | -6.43 | 5.08E-04 |
| A_23_P104318 | DDIT4 | NM_019058 | -6.38 | 1.19E-06 |
| A_33_P3302632 | HIST1H2BE | NM_003523 | -6.37 | 5.98E-05 |
| A_23_P104318 | DDIT4 | NM_019058 | -6.35 | 5.78E-06 |
| A_23_P104318 | DDIT4 | NM_019058 | -6.28 | 6.18E-06 |
| A_23_P104318 | DDIT4 | NM_019058 | -6.27 | 8.31E-06 |
| A_24_P250922 | PTGS2 | NM_000963 | -6.25 | 1.39E-04 |
| A_23_P7976 | HIST1H1E | NM_005321 | -6.13 | 3.79E-06 |
| A_23_P145238 | HIST1H2BK | NM_080593 | -6.11 | 2.36E-05 |
| A_24_P3783 | HIST1H2BM | NM_003521 | -6.04 | 6.66E-06 |
| A_23_P104318 | DDIT4 | NM_019058 | -6.03 | 2.93E-06 |
| A_33_P3344127 | HIST1H2AC | NM_003512 | -6.03 | 5.23E-06 |
| A_24_P250922 | PTGS2 | NM_000963 | -5.97 | 2.65E-04 |
| A_23_P149545 | HIST2H2BE | NM_003528 | -5.94 | 3.02E-04 |
| A_24_P250922 | PTGS2 | NM_000963 | -5.87 | 3.49E-04 |
| A_33_P3232692 | IL24 | NM_001185156 | -5.86 | 2.02E-04 |
| A_33_P3360216 | HIST1H2AI | NM_003509 | -5.78 | 1.70E-06 |
| A_24_P105933 | VIPR1 | NM_004624 | -5.64 | 2.33E-04 |
| A_21_P0000633 | LOC255130 | NR_034081 | -5.61 | 5.67E-04 |
| A_23_P167997 | HIST1H2BG | NM_003518 | -5.58 | 1.17E-05 |
| A_23_P111054 | HIST1H2BB | NM_021062 | -5.57 | 1.00E-05 |
| A_21_P0010654 | HIST2H2BF | NM_001161334 | -5.37 | 1.20E-05 |
| A_23_P111041 | HIST1H2BI | NM_003525 | -5.35 | 3.97E-05 |
| A_33_P3229083 | HIST1H2BK | NM_080593 | -5.29 | 3.77E-05 |
| A_23_P70398 | VEGFA | NM_001025370 | -5.24 | 2.23E-05 |
| A_24_P223384 | HIST1H2AB | NM_003513 | -5.23 | 2.20E-06 |
| A_33_P3381235 | LOC100127888 | NR_024470 | -5.23 | 8.97E-05 |
| A_23_P93180 | HIST1H2BC | NM_003526 | -5.22 | 2.71E-05 |
| A_24_P250922 | PTGS2 | NM_000963 | -5.08 | 7.31E-06 |
| A_23_P59069 | HIST1H2BO | NM_003527 | -5.00 | 2.86E-05 |
| A_23_P366216 | HIST1H2BH | NM_003524 | -4.99 | 3.69E-05 |
| A_23_P251043 | SYNDIG1 | NM_024893 | -4.90 | 5.02E-04 |
| A_24_P86389 | HIST1H2AM | NM_003514 | -4.83 | 7.68E-06 |
| A_32_P75661 | THC2544198 | THC2544198 | -4.80 | 2.55E-06 |
| A_23_P163402 | CYP1A1 | NM_000499 | -4.77 | 6.93E-04 |
| A_33_P3222424 | CSF3 | NM_000759 | -4.77 | 2.37E-04 |
| A_23_P8013 | HIST1H2BL | NM_003519 | -4.72 | 4.20E-05 |
| A_24_P277673 | HIST1H4G | NM_003547 | -4.70 | 7.73E-04 |
| A_23_P135548 | DPYD | NM_000110 | -4.62 | 1.28E-05 |
| A_32_P221799 | HIST1H2AM | NM_003514 | -4.60 | 1.61E-06 |
| A_33_P3213377 | IMMP2L | NM_032549 | -4.59 | 1.83E-05 |
| A_21_P0014707 | LOC100508950 | XR_112293 | -4.57 | 6.10E-05 |
| A_33_P3236102 | IER5L | NM_203434 | -4.53 | 2.56E-05 |
| A_24_P146211 | HIST1H2BD | NM_021063 | -4.41 | 3.45E-05 |
| A_24_P217848 | HIST1H2AK | NM_003510 | -4.38 | 6.91E-06 |
| A_24_P915692 | PHLDA1 | NM_007350 | -4.38 | 9.81E-05 |
| A_23_P81859 | HIST1H2AH | NM_080596 | -4.28 | 7.70E-06 |
| A_33_P3299958 | ENST00000451368 | ENST00000451368 | -4.23 | 8.02E-05 |
| A_23_P30693 | PLG | NM_000301 | -4.22 | 4.44E-04 |
| A_21_P0000197 | HIST1H2AG | NM_021064 | -4.20 | 3.19E-05 |
| A_23_P29257 | H1F0 | NM_005318 | -4.00 | 1.19E-06 |
| A_23_P93258 | HIST1H3B | NM_003537 | -3.95 | 6.96E-06 |
| A_23_P372860 | HIST1H2AC | NM_003512 | -3.94 | 9.68E-06 |
| A_23_P161624 | FOSL1 | NM_005438 | -3.93 | 1.38E-06 |
| A_23_P324754 | KIAA1199 | NM_018689 | -3.84 | 1.07E-05 |
| A_23_P10206 | HAS2 | NM_005328 | -3.83 | 1.67E-04 |
| A_33_P3395651 | PCDHGA5 | NM_032054 | -3.83 | 6.73E-04 |
| A_23_P10206 | HAS2 | NM_005328 | -3.81 | 1.44E-04 |
| A_23_P214267 | GPR110 | NM_153840 | -3.77 | 6.78E-04 |
| A_23_P10206 | HAS2 | NM_005328 | -3.75 | 1.02E-04 |
| A_23_P10206 | HAS2 | NM_005328 | -3.74 | 1.51E-04 |
| A_24_P55148 | HIST1H2BJ | NM_021058 | -3.74 | 8.02E-05 |
| A_23_P41476 | SHISA3 | NM_001080505 | -3.72 | 6.19E-06 |
| A_23_P10206 | HAS2 | NM_005328 | -3.69 | 9.46E-05 |
| A_23_P10206 | HAS2 | NM_005328 | -3.68 | 4.56E-04 |
| A_21_P0010689 | XLOC_l2_000018 | ENST00000433695 | -3.66 | 8.05E-05 |
| A_33_P3253501 | HIST2H2BF | NM_001161334 | -3.65 | 1.94E-05 |
| A_23_P10206 | HAS2 | NM_005328 | -3.65 | 4.26E-04 |
| A_23_P154050 | CDK15 | NM_139158 | -3.64 | 5.63E-04 |
| A_24_P12401 | VEGFA | NM_001025366 | -3.63 | 1.77E-06 |
| A_33_P3344086 | HIST1H2AJ | NM_021066 | -3.61 | 3.33E-06 |
| A_33_P3368495 | AK057067 | AK057067 | -3.52 | 5.67E-04 |
| A_23_P10206 | HAS2 | NM_005328 | -3.50 | 7.68E-04 |
| A_33_P3287879 | HIST1H3H | NM_003536 | -3.49 | 1.67E-05 |
| A_23_P431179 | HIST1H4A | NM_003538 | -3.48 | 2.16E-04 |
| A_33_P3216601 | FHIT | NM_002012 | -3.42 | 8.81E-05 |
| A_23_P10206 | HAS2 | NM_005328 | -3.40 | 1.48E-04 |
| A_24_P931443 | GPR68 | NM_003485 | -3.32 | 2.31E-04 |
| A_33_P3303649 | MB | NM_203377 | -3.29 | 8.74E-04 |
| A_33_P3293164 | CHGA | NM_001275 | -3.29 | 2.20E-05 |
| A_32_P32254 | COL6A1 | NM_001848 | -3.28 | 6.17E-05 |
| A_23_P301247 | HIST2H2AC | NM_003517 | -3.27 | 1.05E-04 |
| A_23_P159255 | PTPRM | NM_002845 | -3.27 | 1.06E-05 |
| A_23_P153945 | GTDC1 | NM_001006636 | -3.26 | 1.55E-04 |
| A_23_P395374 | HIST1H4D | NM_003539 | -3.26 | 5.67E-06 |
| A_23_P323823 | HIST1H2BA | NM_170610 | -3.23 | 1.55E-06 |
| A_33_P3229672 | THC2651904 | THC2651904 | -3.22 | 1.09E-05 |
| A_24_P217834 | HIST1H3D | NM_003530 | -3.21 | 3.10E-06 |
| A_23_P44155 | CD96 | NM_198196 | -3.17 | 1.98E-04 |
| A_33_P3344229 | HIST1H4A | NM_003538 | -3.15 | 7.75E-06 |
| A_21_P0002492 | XLOC_002275 | CR749588 | -3.15 | 9.74E-05 |
| A_23_P44781 | CDKAL1 | NM_017774 | -3.13 | 1.74E-05 |
| A_33_P3378880 | ENST00000377803 | ENST00000377803 | -3.11 | 2.15E-05 |
| A_24_P111912 | FAM172A | NM_032042 | -3.11 | 2.16E-05 |
| A_21_P0014172 | LOC100507025 | ENST00000289352 | -3.09 | 1.48E-04 |
| A_23_P104972 | MS4A5 | NM_023945 | -3.07 | 9.41E-05 |
| A_32_P190303 | LONRF2 | NM_198461 | -3.07 | 3.45E-06 |
| A_24_P300777 | ADAM8 | NM_001109 | -3.07 | 2.88E-04 |
| A_33_P3543133 | LOC283624 | NR_038970 | -3.06 | 5.97E-04 |
| A_23_P128215 | SOCS2 | NM_003877 | -3.06 | 4.57E-04 |
| A_23_P422212 | SLC35F3 | NM_173508 | -3.05 | 1.37E-05 |
| A_24_P303193 | BG036557 | BG036557 | -3.05 | 3.55E-04 |
| A_23_P308150 | FAM123B | NM_152424 | -3.00 | 9.12E-05 |
| A_23_P355455 | TBC1D5 | NM_014744 | -2.94 | 1.88E-04 |
| A_32_P88310 | LOC730183 | XR_109284 | -2.93 | 1.01E-05 |
| A_33_P3302423 | TNRC6C | NM_001142640 | -2.93 | 1.69E-04 |
| A_23_P11685 | PLA2G4A | NM_024420 | -2.93 | 3.85E-04 |
| A_23_P7313 | SPP1 | NM_001040058 | -2.93 | 4.90E-04 |
| A_33_P3409139 | LOC143188 | NR_015409 | -2.93 | 2.50E-04 |
| A_24_P390668 | FMNL1 | NM_005892 | -2.91 | 7.29E-05 |
| A_23_P21363 | AHNAK | NM_024060 | -2.91 | 2.73E-05 |
| A_24_P166407 | HIST1H4B | NM_003544 | -2.90 | 4.93E-06 |
| A_23_P323685 | HIST1H4H | NM_003543 | -2.89 | 7.09E-05 |
| A_23_P416894 | PION | NM_017439 | -2.88 | 2.34E-04 |
| A_23_P218646 | TNFRSF6B | NM_003823 | -2.88 | 7.62E-05 |
| A_33_P3303121 | RAP2B | NM_002886 | -2.87 | 2.46E-04 |
| A_23_P112201 | KDM4C | NM_015061 | -2.86 | 2.14E-04 |
| A_33_P3346688 | HSPA8 | ENST00000527983 | -2.86 | 5.28E-04 |
| A_33_P3240507 | KCTD12 | NM_138444 | -2.86 | 7.15E-05 |
| A_24_P902052 | SNHG13 | NR_024031 | -2.86 | 4.54E-08 |
| A_24_P941268 | CA5B | NM_007220 | -2.84 | 2.43E-05 |
| A_33_P3240512 | KCTD12 | NM_138444 | -2.84 | 1.11E-04 |
| A_23_P112846 | MTHFD2L | NM_001144978 | -2.83 | 5.35E-05 |
| A_32_P17635 | SRSF8 | NM_032102 | -2.83 | 1.31E-04 |
| A_24_P827 | SUPT3H | NM_003599 | -2.83 | 7.57E-04 |
| A_23_P112846 | MTHFD2L | NM_001144978 | -2.82 | 2.25E-04 |
| A_32_P99100 | PTPRK | NM_002844 | -2.82 | 3.67E-06 |
| A_23_P112846 | MTHFD2L | NM_001144978 | -2.82 | 2.49E-04 |
| A_33_P3375002 | LOC144481 | NR_038263 | -2.81 | 7.57E-05 |
| A_33_P3772150 | ADARB2 | NM_018702 | -2.78 | 7.81E-04 |
| A_23_P78092 | EVI2A | NM_001003927 | -2.77 | 9.99E-05 |
| A_23_P7313 | SPP1 | NM_001040058 | -2.75 | 6.48E-04 |
| A_23_P30813 | HIST1H4K | NM_003541 | -2.75 | 4.48E-04 |
| A_33_P3382303 | FMNL1 | NM_005892 | -2.74 | 3.12E-05 |
| A_23_P105118 | OR51G1 | NM_001005237 | -2.74 | 6.42E-04 |
| A_33_P3221253 | LPP | NM_005578 | -2.74 | 5.33E-04 |
| A_21_P0001767 | FONG | NR_034096 | -2.73 | 1.91E-05 |
| A_23_P42198 | HIST1H3G | NM_003534 | -2.73 | 3.33E-06 |
| A_33_P3407636 | YWHAE | AK296555 | -2.73 | 2.53E-06 |
| A_33_P3245278 | PTPRG | NM_002841 | -2.73 | 6.05E-06 |
| A_24_P410610 | DPYD | NM_001160301 | -2.72 | 1.31E-04 |
| A_23_P1691 | MMP1 | NM_002421 | -2.71 | 5.93E-04 |
| A_33_P3379456 | SH3YL1 | NM_015677 | -2.70 | 7.75E-04 |
| A_32_P103291 | SMYD3 | NM_022743 | -2.70 | 1.59E-04 |
| A_33_P3256113 | GUSBP1 | NR_027026 | -2.69 | 1.42E-04 |
| A_23_P133236 | PCDHB14 | NM_018934 | -2.69 | 2.52E-04 |
| A_23_P217015 | SET | NM_003011 | -2.69 | 2.12E-04 |
| A_23_P112846 | MTHFD2L | NM_001144978 | -2.68 | 9.32E-04 |
| A_21_P0011022 | XLOC_l2_002271 | ENST00000513853 | -2.68 | 1.46E-06 |
| A_23_P112201 | KDM4C | NM_015061 | -2.68 | 7.02E-04 |
| A_23_P110276 | AFAP1-AS1 | NR_026892 | -2.68 | 1.56E-04 |
| A_23_P252403 | COMMD10 | NM_016144 | -2.68 | 6.24E-07 |
| A_23_P1691 | MMP1 | NM_002421 | -2.68 | 1.56E-04 |
| A_32_P174365 | SATB2 | NM_015265 | -2.67 | 2.95E-06 |
| A_21_P0007920 | XLOC_010287 | ENST00000422082 | -2.67 | 5.88E-04 |
| A_23_P11685 | PLA2G4A | NM_024420 | -2.66 | 4.34E-04 |
| A_32_P192376 | ENPP1 | NM_006208 | -2.66 | 2.78E-04 |
| A_23_P11685 | PLA2G4A | NM_024420 | -2.66 | 4.51E-04 |
| A_23_P156880 | ENPP1 | NM_006208 | -2.65 | 6.52E-05 |
| A_23_P70480 | HIST1H4L | NM_003546 | -2.65 | 1.32E-05 |
| A_21_P0011972 | XLOC_l2_008313 | THC2651904 | -2.64 | 6.48E-05 |
| A_19_P00322687 | ERVMER34-1 | NM_001242690 | -2.64 | 2.82E-06 |
| A_23_P112201 | KDM4C | NM_015061 | -2.64 | 9.85E-05 |
| A_33_P3272105 | FLJ38576 | XR_112683 | -2.63 | 2.27E-04 |
| A_21_P0009412 | LOC100499467 | NR_036488 | -2.62 | 6.91E-04 |
| A_23_P113393 | APLN | NM_017413 | -2.62 | 1.54E-04 |
| A_23_P127948 | ADM | NM_001124 | -2.62 | 3.93E-04 |
| A_33_P3257678 | HIST2H3A | NM_001005464 | -2.62 | 6.80E-06 |
| A_21_P0011023 | XLOC_l2_002271 | ENST00000511073 | -2.62 | 7.85E-04 |
| A_19_P00318261 | SNORA26 | NR_003016 | -2.61 | 1.98E-06 |
| A_33_P3314441 | FBXL17 | NM_001163315 | -2.61 | 9.43E-05 |
| A_33_P3299865 | HIST1H4K | NM_003541 | -2.61 | 6.01E-05 |
| A_23_P204879 | CAB39L | NM_030925 | -2.61 | 2.24E-05 |
| A_23_P1691 | MMP1 | NM_002421 | -2.60 | 5.48E-04 |
| A_33_P3391796 | NOG | NM_005450 | -2.60 | 1.00E-04 |
| A_24_P706340 | FAM155A | NM_001080396 | -2.59 | 1.11E-05 |
| A_24_P20873 | HIST1H4I | NM_003495 | -2.59 | 1.11E-04 |
| A_23_P113393 | APLN | NM_017413 | -2.59 | 7.41E-05 |
| A_33_P3313622 | MIR17HG | NR_027350 | -2.58 | 2.20E-05 |
| A_24_P9321 | HIST1H3I | NM_003533 | -2.58 | 4.54E-06 |
| A_23_P218597 | NPAS2 | NM_002518 | -2.58 | 1.16E-04 |
| A_23_P331235 | C5orf38 | NM_178569 | -2.57 | 4.82E-04 |
| A_33_P3290729 | POU3F2 | NM_005604 | -2.57 | 3.71E-04 |
| A_23_P127948 | ADM | NM_001124 | -2.57 | 8.20E-04 |
| A_23_P127948 | ADM | NM_001124 | -2.57 | 4.12E-04 |
| A_23_P127948 | ADM | NM_001124 | -2.57 | 6.41E-04 |
| A_23_P21092 | CALB2 | NM_001740 | -2.57 | 8.66E-04 |
| A_23_P127948 | ADM | NM_001124 | -2.56 | 7.89E-04 |
| A_23_P127948 | ADM | NM_001124 | -2.56 | 7.12E-04 |
| A_23_P32577 | DACH1 | NM_080759 | -2.56 | 9.12E-04 |
| A_33_P3699445 | BC025792 | BC025792 | -2.56 | 1.42E-04 |
| A_23_P10995 | RBMS3 | NM_014483 | -2.55 | 2.49E-04 |
| A_21_P0011518 | XLOC_l2_005690 | ENST00000437646 | -2.54 | 5.60E-04 |
| A_23_P11685 | PLA2G4A | NM_024420 | -2.53 | 3.50E-04 |
| A_33_P3238390 | SETP20 | XM_001717714 | -2.53 | 1.49E-04 |
| A_23_P359540 | HIST1H4F | NM_003540 | -2.53 | 9.38E-05 |
| A_23_P112846 | MTHFD2L | NM_001144978 | -2.53 | 6.91E-05 |
| A_23_P110276 | AFAP1-AS1 | NR_026892 | -2.52 | 2.90E-04 |
| A_23_P30805 | HIST1H4J | NM_021968 | -2.52 | 2.41E-04 |
| A_23_P136986 | APOOL | NM_198450 | -2.51 | 5.40E-04 |
| A_33_P3227990 | MBP | NM_001025101 | -2.51 | 8.87E-04 |
| A_33_P3280320 | LOC100288524 | NM_001195127 | -2.51 | 5.31E-04 |
| A_33_P3267160 | AK124658 | AK124658 | -2.50 | 2.24E-04 |
| A_33_P3781228 | FLJ26332 | AK129842 | -2.50 | 7.22E-04 |
| A_23_P110276 | AFAP1-AS1 | NR_026892 | -2.50 | 5.76E-04 |
| A_23_P66694 | EVI2B | NM_006495 | -2.50 | 2.48E-04 |
| A_23_P101905 | APC2 | NM_005883 | -2.49 | 7.26E-04 |
| A_33_P3373750 | BRD4 | NM_014299 | -2.49 | 2.14E-05 |
| A_23_P96087 | H1FX | NM_006026 | -2.49 | 1.97E-05 |
| A_23_P354805 | KLF12 | NM_007249 | -2.49 | 1.44E-04 |
| A_23_P112201 | KDM4C | NM_015061 | -2.49 | 5.18E-04 |
| A_33_P3314436 | FBXL17 | NM_001163315 | -2.48 | 3.63E-04 |
| A_23_P127948 | ADM | NM_001124 | -2.48 | 8.10E-04 |
| A_23_P112846 | MTHFD2L | NM_001144978 | -2.47 | 9.32E-05 |
| A_23_P110276 | AFAP1-AS1 | NR_026892 | -2.47 | 6.77E-04 |
| A_24_P137897 | IFRD1 | NM_001007245 | -2.46 | 1.71E-07 |
| A_23_P117424 | DCAF11 | NM_025230 | -2.45 | 3.15E-04 |
| A_23_P436281 | HIST2H4B | NM_001034077 | -2.44 | 1.61E-04 |
| A_23_P112201 | KDM4C | NM_015061 | -2.44 | 9.61E-04 |
| A_32_P524014 | UTRN | NM_007124 | -2.43 | 1.63E-04 |
| A_33_P3392192 | NRG1 | AF176921 | -2.43 | 2.95E-06 |
| A_24_P360078 | LRBA | NM_006726 | -2.43 | 8.76E-06 |
| A_23_P96853 | FAF1 | NM_007051 | -2.43 | 1.53E-05 |
| A_33_P3334313 | ACTR3B | NM_020445 | -2.43 | 3.09E-04 |
| A_23_P110276 | AFAP1-AS1 | NR_026892 | -2.43 | 7.17E-04 |
| A_23_P211110 | SIM2 | NM_005069 | -2.43 | 8.12E-04 |
| A_21_P0000003 | PRR4 | NM_007244 | -2.42 | 3.04E-04 |
| A_23_P30799 | HIST1H3F | NM_021018 | -2.42 | 1.79E-05 |
| A_21_P0000636 | TRAF3IP2-AS1 | NR_034110 | -2.42 | 4.81E-04 |
| A_33_P3259183 | FAM78B | NM_001017961 | -2.42 | 5.44E-04 |
| A_21_P0002505 | XLOC_002323 | TCONS_00004445 | -2.41 | 8.53E-05 |
| A_33_P3799692 | LOC338620 | BC043009 | -2.41 | 2.03E-04 |
| A_23_P110276 | AFAP1-AS1 | NR_026892 | -2.40 | 6.41E-04 |
| A_33_P3326984 | MOK | ENST00000520252 | -2.39 | 5.94E-07 |
| A_33_P3300312 | DMBT1 | NM_007329 | -2.39 | 2.53E-05 |
| A_23_P133814 | HIST1H3C | NM_003531 | -2.39 | 1.42E-05 |
| A_33_P3237517 | ZNF292 | ENST00000369578 | -2.39 | 1.54E-05 |
| A_23_P113393 | APLN | NM_017413 | -2.38 | 5.74E-04 |
| A_21_P0004722 | XLOC_005592 | AK126470 | -2.38 | 7.13E-04 |
| A_32_P209960 | CIITA | NM_000246 | -2.38 | 7.63E-04 |
| A_19_P00321110 | LOC100505519 | ENST00000449545 | -2.38 | 5.17E-04 |
| A_21_P0014380 | LOC100507486 | XR_109525 | -2.38 | 1.24E-04 |
| A_33_P3359856 | MZT2A | ENST00000445782 | -2.38 | 4.82E-05 |
| A_23_P117424 | DCAF11 | NM_025230 | -2.38 | 1.03E-05 |
| A_24_P354715 | NT5E | NM_002526 | -2.37 | 1.11E-04 |
| A_33_P3265872 | ENST00000419668 | ENST00000419668 | -2.37 | 8.98E-05 |
| A_23_P70445 | HIST1H3E | NM_003532 | -2.37 | 6.58E-07 |
| A_33_P3563369 | RPS2 | ENST00000526586 | -2.37 | 1.09E-06 |
| A_21_P0009798 | XLOC_013182 | THC2532393 | -2.35 | 2.32E-05 |
| A_23_P86599 | DMBT1 | NM_007329 | -2.35 | 4.63E-06 |
| A_23_P329152 | ILF3 | NM_012218 | -2.35 | 3.22E-04 |
| A_23_P420942 | ENST00000330439 | ENST00000330439 | -2.34 | 3.78E-04 |
| A_23_P162279 | CCDC91 | NM_018318 | -2.34 | 1.08E-04 |
| A_23_P434301 | PTMA | NM_002823 | -2.34 | 2.23E-08 |
| A_24_P414371 | PPP3CA | NM_000944 | -2.34 | 7.90E-06 |
| A_23_P95029 | SNTB1 | NM_021021 | -2.34 | 2.98E-04 |
| A_24_P98251 | VPS13B | NM_017890 | -2.34 | 3.80E-05 |
| A_23_P117424 | DCAF11 | NM_025230 | -2.33 | 8.06E-06 |
| A_23_P571 | SLC2A1 | NM_006516 | -2.33 | 3.02E-04 |
| A_23_P86838 | SLC36A4 | NM_152313 | -2.33 | 1.45E-06 |
| A_23_P110276 | AFAP1-AS1 | NR_026892 | -2.33 | 5.48E-04 |
| A_23_P36972 | ZIC2 | NM_007129 | -2.32 | 6.07E-05 |
| A_32_P187617 | TDRD3 | NM_030794 | -2.32 | 3.28E-04 |
| A_23_P385063 | DNAJB6 | NM_058246 | -2.32 | 1.97E-05 |
| A_23_P76969 | SIPA1L1 | NM_015556 | -2.32 | 1.42E-05 |
| A_23_P110276 | AFAP1-AS1 | NR_026892 | -2.32 | 5.75E-04 |
| A_23_P115375 | HIST2H3D | NM_001123375 | -2.32 | 7.74E-06 |
| A_23_P257423 | ALG14 | NM_144988 | -2.32 | 5.86E-05 |
| A_33_P3231156 | ENST00000379816 | ENST00000379816 | -2.32 | 4.95E-04 |
| A_33_P3410836 | HIST1H4D | NM_003539 | -2.32 | 7.72E-05 |
| A_33_P3346403 | PTMA | NM_001099285 | -2.32 | 2.00E-06 |
| A_33_P3326989 | MOK | NM_014226 | -2.31 | 1.13E-04 |
| A_23_P47991 | MED13L | NM_015335 | -2.31 | 1.18E-04 |
| A_21_P0014108 | PVRL3 | NM_001243288 | -2.31 | 1.62E-04 |
| A_21_P0012454 | PTPN11 | NM_002834 | -2.31 | 2.62E-04 |
| A_23_P92543 | SCFD2 | NM_152540 | -2.30 | 4.73E-05 |
| A_33_P3357949 | ETV1 | NM_004956 | -2.30 | 1.82E-04 |
| A_23_P117424 | DCAF11 | NM_025230 | -2.30 | 2.10E-05 |
| A_33_P3421490 | KIAA1024 | NM_015206 | -2.29 | 1.71E-04 |
| A_33_P3280945 | SNHG3 | NR_036473 | -2.29 | 8.10E-05 |
| A_33_P3373985 | LOC100130009 | XM_001718914 | -2.29 | 4.16E-05 |
| A_33_P3311076 | CYB5A | NM_001190807 | -2.29 | 4.97E-04 |
| A_23_P95930 | HMGA2 | NM_003483 | -2.29 | 4.83E-05 |
| A_23_P44964 | FAM171A1 | NM_001010924 | -2.29 | 5.30E-06 |
| A_23_P101208 | CYB5A | NM_001914 | -2.28 | 5.96E-04 |
| A_21_P0013910 | PTMA | NM_001099285 | -2.28 | 1.15E-05 |
| A_23_P1331 | COL13A1 | NM_080801 | -2.28 | 1.65E-06 |
| A_33_P3713357 | ALCAM | NM_001627 | -2.28 | 8.49E-06 |
| A_23_P1331 | COL13A1 | NM_080801 | -2.28 | 3.87E-05 |
| A_23_P344988 | ICK | NM_016513 | -2.27 | 2.00E-05 |
| A_23_P101208 | CYB5A | NM_001914 | -2.27 | 1.24E-04 |
| A_23_P69810 | AGPAT9 | NM_032717 | -2.27 | 5.74E-06 |
| A_23_P117424 | DCAF11 | NM_025230 | -2.27 | 8.22E-05 |
| A_23_P117424 | DCAF11 | NM_025230 | -2.27 | 9.66E-05 |
| A_23_P150693 | FJX1 | NM_014344 | -2.27 | 6.13E-04 |
| A_23_P1331 | COL13A1 | NM_080801 | -2.27 | 5.94E-05 |
| A_23_P117424 | DCAF11 | NM_025230 | -2.27 | 1.33E-05 |
| A_24_P542375 | PTMA | NM_002823 | -2.27 | 1.10E-06 |
| A_23_P101208 | CYB5A | NM_001914 | -2.27 | 3.98E-04 |
| A_33_P3326588 | TNFRSF10D | NM_003840 | -2.27 | 4.84E-05 |
| A_23_P115743 | FAM204A | NM_022063 | -2.26 | 9.91E-04 |
| A_23_P1331 | COL13A1 | NM_080801 | -2.26 | 7.47E-05 |
| A_23_P117424 | DCAF11 | NM_025230 | -2.26 | 2.67E-05 |
| A_33_P3277674 | FBXL22 | NM_203373 | -2.26 | 9.08E-04 |
| A_24_P49747 | XM_929965 | XM_929965 | -2.26 | 4.93E-04 |
| A_23_P1331 | COL13A1 | NM_080801 | -2.26 | 3.65E-05 |
| A_24_P264207 | PTMA | NM_002823 | -2.26 | 9.11E-07 |
| A_23_P214487 | HIST1H4C | NM_003542 | -2.26 | 1.59E-07 |
| A_23_P101208 | CYB5A | NM_001914 | -2.25 | 4.95E-05 |
| A_33_P3401156 | ETV1 | NM_004956 | -2.25 | 3.79E-05 |
| A_23_P426305 | AOC3 | NM_003734 | -2.25 | 9.23E-04 |
| A_23_P115743 | FAM204A | NM_022063 | -2.25 | 3.24E-05 |
| A_33_P3353051 | C6orf48 | NM_001040438 | -2.25 | 9.58E-05 |
| A_23_P45059 | DOCK1 | NM_001380 | -2.25 | 8.25E-05 |
| A_23_P321452 | PDZD8 | NM_173791 | -2.25 | 9.31E-05 |
| A_33_P3374293 | PYROXD1 | NM_024854 | -2.24 | 1.10E-04 |
| A_21_P0001240 | XLOC_001257 | ENST00000425412 | -2.24 | 9.74E-04 |
| A_32_P29118 | SEMA3D | NM_152754 | -2.24 | 4.21E-04 |
| A_33_P3315223 | HNRNPA0 | NM_006805 | -2.24 | 6.21E-05 |
| A_33_P3221868 | TMEM216 | NM_001173990 | -2.24 | 5.50E-04 |
| A_19_P00315668 | HIPK2 | NM_022740 | -2.24 | 1.26E-04 |
| A_24_P21056 | PHF14 | NM_014660 | -2.23 | 1.15E-04 |
| A_23_P101208 | CYB5A | NM_001914 | -2.23 | 4.41E-04 |
| A_23_P373119 | HMGB3P1 | NR_002165 | -2.23 | 6.48E-04 |
| A_23_P115743 | FAM204A | NM_022063 | -2.23 | 1.97E-05 |
| A_19_P00318425 | ERVMER34-1 | NM_001242690 | -2.23 | 2.64E-04 |
| A_33_P3358626 | TAF4B | NM_005640 | -2.23 | 6.51E-05 |
| A_23_P51410 | SMYD3 | NM_022743 | -2.23 | 4.02E-05 |
| A_23_P52298 | NPM3 | NM_006993 | -2.23 | 1.39E-04 |
| A_32_P1701 | POLA1 | NM_016937 | -2.23 | 2.01E-04 |
| A_23_P419795 | SBF2 | NM_030962 | -2.22 | 1.97E-04 |
| A_24_P917866 | SET | NM_003011 | -2.22 | 7.23E-06 |
| A_23_P117424 | DCAF11 | NM_025230 | -2.21 | 5.39E-05 |
| A_24_P368544 | SLC25A26 | NM_173471 | -2.21 | 1.87E-04 |
| A_32_P80597 | ELOVL6 | ENST00000394607 | -2.21 | 6.75E-04 |
| A_23_P7083 | C4orf42 | NR_033339 | -2.21 | 3.18E-04 |
| A_21_P0014236 | LOC100507319 | XR_108980 | -2.21 | 9.66E-04 |
| A_23_P101208 | CYB5A | NM_001914 | -2.21 | 1.37E-04 |
| A_23_P115743 | FAM204A | NM_022063 | -2.21 | 4.38E-04 |
| A_23_P89941 | CDKN2D | NM_001800 | -2.20 | 1.19E-04 |
| A_23_P1331 | COL13A1 | NM_080801 | -2.20 | 1.27E-05 |
| A_23_P66241 | MT1M | NM_176870 | -2.20 | 3.68E-05 |
| A_24_P303145 | ANKH | NM_054027 | -2.20 | 4.39E-04 |
| A_23_P115743 | FAM204A | NM_022063 | -2.20 | 8.72E-05 |
| A_23_P1331 | COL13A1 | NM_080801 | -2.20 | 6.85E-05 |
| A_33_P3619819 | LOC100289388 | AK124949 | -2.20 | 2.10E-05 |
| A_23_P101208 | CYB5A | NM_001914 | -2.20 | 2.68E-04 |
| A_32_P38623 | PPP1R9A | NM_017650 | -2.20 | 5.02E-04 |
| A_33_P3229241 | HIST2H2BF | NM_001024599 | -2.19 | 1.02E-04 |
| A_23_P1331 | COL13A1 | NM_080801 | -2.19 | 4.20E-06 |
| A_21_P0000671 | MT1E | NM_175617 | -2.19 | 7.88E-06 |
| A_23_P117424 | DCAF11 | NM_025230 | -2.19 | 9.95E-06 |
| A_32_P118847 | FLJ42709 | NR_021491 | -2.19 | 8.77E-05 |
| A_32_P34589 | RSRC1 | NM_016625 | -2.19 | 1.60E-06 |
| A_23_P101208 | CYB5A | NM_001914 | -2.18 | 1.54E-04 |
| A_33_P3594654 | TMEM185B | NM_024121 | -2.18 | 4.49E-05 |
| A_23_P1331 | COL13A1 | NM_080801 | -2.18 | 1.31E-05 |
| A_33_P3242748 | ARFRP1 | NM_001134758 | -2.18 | 2.08E-05 |
| A_23_P99027 | PTPN11 | NM_002834 | -2.18 | 2.90E-05 |
| A_21_P0011047 | XLOC_l2_003021 | BF789912 | -2.18 | 4.44E-05 |
| A_24_P916496 | PRKCA | NM_002737 | -2.17 | 3.66E-05 |
| A_24_P141688 | PCBP2 | NM_005016 | -2.17 | 4.84E-04 |
| A_23_P386888 | LPA | NM_005577 | -2.17 | 8.70E-04 |
| A_23_P42331 | HMGA1 | NM_145901 | -2.17 | 7.99E-05 |
| A_32_P27479 | NLRP11 | NM_145007 | -2.17 | 4.83E-04 |
| A_23_P17593 | CDH4 | NM_001794 | -2.17 | 7.01E-05 |
| A_24_P338187 | HNRNPH3 | NM_012207 | -2.17 | 4.97E-05 |
| A_23_P74229 | STK40 | NM_032017 | -2.17 | 4.10E-04 |
| A_24_P122137 | LIF | NM_002309 | -2.17 | 1.99E-04 |
| A_24_P290163 | ENST00000330588 | ENST00000330588 | -2.16 | 1.20E-06 |
| A_23_P401472 | CHRM3 | NM_000740 | -2.16 | 4.68E-04 |
| A_33_P3775007 | ASAP1 | NM_001247996 | -2.16 | 1.33E-04 |
| A_33_P3234864 | UTRN | NM_007124 | -2.15 | 1.71E-04 |
| A_23_P115743 | FAM204A | NM_022063 | -2.15 | 1.08E-04 |
| A_23_P76731 | MOK | NM_014226 | -2.15 | 1.88E-05 |
| A_33_P3298430 | FAM171A1 | NM_001010924 | -2.15 | 5.26E-05 |
| A_23_P115743 | FAM204A | NM_022063 | -2.15 | 1.19E-04 |
| A_21_P0012930 | LOC643201 | NR_036494 | -2.15 | 6.41E-05 |
| A_33_P3288110 | PKP4 | NM_001005476 | -2.15 | 6.94E-05 |
| A_23_P99027 | PTPN11 | NM_002834 | -2.14 | 2.28E-06 |
| A_23_P53039 | LDHC | NM_002301 | -2.14 | 2.99E-04 |
| A_23_P10785 | VTI1A | NM_145206 | -2.14 | 8.34E-05 |
| A_21_P0011876 | XLOC_l2_007783 | THC2707941 | -2.13 | 3.40E-04 |
| A_23_P210048 | HDAC4 | NM_006037 | -2.13 | 1.02E-04 |
| A_24_P305764 | SMS | NM_004595 | -2.13 | 1.53E-05 |
| A_23_P101208 | CYB5A | NM_001914 | -2.13 | 1.21E-04 |
| A_32_P64200 | GUCA1B | NM_002098 | -2.13 | 6.35E-04 |
| A_23_P131646 | RPIA | NM_144563 | -2.13 | 2.44E-06 |
| A_24_P135322 | NRP1 | NM_001024629 | -2.13 | 4.25E-04 |
| A_32_P523096 | MGC12982 | NR_026878 | -2.13 | 2.31E-04 |
| A_23_P5831 | HPCAL1 | NM_134421 | -2.12 | 6.99E-05 |
| A_23_P321034 | INADL | NM_176877 | -2.12 | 1.70E-04 |
| A_21_P0004996 | XLOC_005592 | TCONS_00012063 | -2.12 | 3.61E-04 |
| A_23_P1331 | COL13A1 | NM_080801 | -2.12 | 3.75E-05 |
| A_23_P101208 | CYB5A | NM_001914 | -2.12 | 4.05E-04 |
| A_23_P103631 | EBNA1BP2 | NM_006824 | -2.12 | 2.37E-05 |
| A_23_P76882 | CCNB1IP1 | NM_182851 | -2.12 | 3.86E-04 |
| A_21_P0000363 | SNORA25 | NR_003028 | -2.11 | 4.65E-06 |
| A_23_P121082 | GBE1 | NM_000158 | -2.11 | 6.19E-05 |
| A_23_P153037 | ZNF624 | NM_020787 | -2.11 | 3.31E-04 |
| A_24_P35478 | PARD3 | NM_019619 | -2.11 | 1.30E-04 |
| A_23_P103631 | EBNA1BP2 | NM_006824 | -2.11 | 5.72E-05 |
| A_23_P206724 | MT1E | NM_175617 | -2.11 | 1.88E-05 |
| A_33_P3650353 | SNORA33 | AI887274 | -2.11 | 2.01E-05 |
| A_23_P122531 | C6orf48 | NM_001040437 | -2.10 | 1.74E-05 |
| A_23_P99027 | PTPN11 | NM_002834 | -2.10 | 1.22E-05 |
| A_23_P122531 | C6orf48 | NM_001040437 | -2.10 | 1.02E-04 |
| A_33_P3403048 | AK294208 | AK294208 | -2.10 | 3.47E-04 |
| A_24_P288722 | CASK | NM_003688 | -2.09 | 9.10E-06 |
| A_23_P122531 | C6orf48 | NM_001040437 | -2.09 | 3.76E-05 |
| A_23_P122531 | C6orf48 | NM_001040437 | -2.09 | 2.68E-05 |
| A_23_P103631 | EBNA1BP2 | NM_006824 | -2.09 | 2.64E-05 |
| A_21_P0012918 | XLOC_l2_012150 | TCONS_l2_00023029 | -2.09 | 1.61E-05 |
| A_19_P00320259 | ERVMER34-1 | NM_001242690 | -2.09 | 8.19E-04 |
| A_23_P122531 | C6orf48 | NM_001040437 | -2.09 | 6.63E-05 |
| A_23_P121082 | GBE1 | NM_000158 | -2.09 | 1.49E-05 |
| A_23_P122531 | C6orf48 | NM_001040437 | -2.09 | 3.82E-06 |
| A_32_P155247 | FTL | NM_000146 | -2.09 | 9.23E-04 |
| A_33_P3219803 | PTMA | NM_001099285 | -2.09 | 5.65E-06 |
| A_23_P170733 | ANTXR2 | NM_058172 | -2.09 | 2.16E-04 |
| A_23_P121082 | GBE1 | NM_000158 | -2.08 | 3.71E-06 |
| A_23_P103631 | EBNA1BP2 | NM_006824 | -2.08 | 2.11E-05 |
| A_33_P3210521 | C2orf43 | ENST00000381090 | -2.08 | 4.10E-04 |
| A_23_P122531 | C6orf48 | NM_001040437 | -2.08 | 1.48E-04 |
| A_23_P115743 | FAM204A | NM_022063 | -2.08 | 2.34E-04 |
| A_32_P161762 | RUNX2 | NM_004348 | -2.08 | 7.81E-05 |
| A_21_P0005447 | LOC202781 | NR_028090 | -2.08 | 1.76E-04 |
| A_33_P3533001 | UBE2E4P | AL568675 | -2.08 | 9.76E-04 |
| A_23_P121082 | GBE1 | NM_000158 | -2.08 | 7.48E-05 |
| A_23_P115743 | FAM204A | NM_022063 | -2.08 | 4.55E-05 |
| A_33_P3279629 | UCN2 | NM_033199 | -2.08 | 7.98E-04 |
| A_21_P0000739 | LOC100288637 | NR_038253 | -2.08 | 3.50E-05 |
| A_23_P398637 | RG9MTD2 | NM_152292 | -2.08 | 2.60E-04 |
| A_21_P0000328 | SNORA40 | NR_002973 | -2.08 | 6.79E-04 |
| A_33_P3231140 | ANKRD50 | NM_001167882 | -2.07 | 2.31E-05 |
| A_33_P3632937 | LOC100131262 | XR_132952 | -2.07 | 6.27E-04 |
| A_23_P122531 | C6orf48 | NM_001040437 | -2.07 | 4.43E-05 |
| A_23_P62081 | SCG5 | NM_003020 | -2.07 | 1.94E-04 |
| A_23_P115636 | TRDMT1 | NM_004412 | -2.07 | 2.97E-04 |
| A_23_P337424 | SERBP1 | NM_001018067 | -2.07 | 1.25E-05 |
| A_23_P121082 | GBE1 | NM_000158 | -2.07 | 3.16E-05 |
| A_21_P0000500 | SNORD86 | NR_004399 | -2.07 | 1.94E-04 |
| A_23_P94159 | FBXO25 | NM_183421 | -2.07 | 1.15E-04 |
| A_23_P103631 | EBNA1BP2 | NM_006824 | -2.07 | 1.64E-05 |
| A_33_P3303372 | PARD3 | NM_001184792 | -2.07 | 3.34E-05 |
| A_32_P168464 | CASK | NM_003688 | -2.07 | 1.00E-05 |
| A_23_P121082 | GBE1 | NM_000158 | -2.06 | 6.50E-06 |
| A_23_P122531 | C6orf48 | NM_001040437 | -2.06 | 8.03E-05 |
| A_23_P103631 | EBNA1BP2 | NM_006824 | -2.06 | 6.87E-06 |
| A_23_P503200 | PHF10 | NM_018288 | -2.06 | 1.65E-04 |
| A_33_P3350056 | MT1X | NM_005952 | -2.06 | 3.49E-04 |
| A_23_P200325 | RABGAP1L | NM_014857 | -2.06 | 1.32E-05 |
| A_33_P3209356 | RFWD2 | NM_022457 | -2.06 | 2.36E-04 |
| A_33_P3419835 | FBXL19-AS1 | NR_024348 | -2.06 | 9.35E-04 |
| A_23_P103631 | EBNA1BP2 | NM_006824 | -2.05 | 1.16E-04 |
| A_23_P108404 | AGAP1 | NM_001037131 | -2.05 | 5.22E-05 |
| A_32_P108889 | DCLK1 | NM_004734 | -2.05 | 9.90E-05 |
| A_23_P121082 | GBE1 | NM_000158 | -2.05 | 2.91E-05 |
| A_21_P0009360 | LOC100499467 | NR_036488 | -2.05 | 1.92E-04 |
| A_23_P121082 | GBE1 | NM_000158 | -2.05 | 2.05E-04 |
| A_19_P00812340 | FTL | NM_000146 | -2.05 | 6.48E-04 |
| A_33_P3423551 | IER3 | NM_003897 | -2.05 | 8.73E-05 |
| A_23_P99027 | PTPN11 | NM_002834 | -2.05 | 3.08E-05 |
| A_33_P3308764 | RXRG | NR_033824 | -2.05 | 8.89E-04 |
| A_23_P50504 | FTL | NM_000146 | -2.04 | 3.97E-04 |
| A_23_P99027 | PTPN11 | NM_002834 | -2.04 | 5.97E-05 |
| A_23_P99027 | PTPN11 | NM_002834 | -2.04 | 2.45E-06 |
| A_23_P74609 | G0S2 | NM_015714 | -2.04 | 1.02E-04 |
| A_33_P3223713 | UBE2E3 | NM_006357 | -2.04 | 7.91E-04 |
| A_33_P3345414 | RPS4X | ENST00000373626 | -2.04 | 9.43E-04 |
| A_23_P42257 | IER3 | NM_003897 | -2.04 | 2.76E-04 |
| A_33_P3267665 | ZDHHC2 | NM_016353 | -2.04 | 3.69E-05 |
| A_23_P115636 | TRDMT1 | NM_004412 | -2.04 | 1.50E-04 |
| A_33_P3315314 | MT1H | NM_005951 | -2.04 | 2.53E-04 |
| A_23_P99027 | PTPN11 | NM_002834 | -2.03 | 1.40E-05 |
| A_21_P0000315 | SNORA18 | NR_002959 | -2.03 | 6.79E-05 |
| A_23_P48358 | PCCA | NM_000282 | -2.03 | 2.63E-04 |
| A_33_P3316508 | RUNX2 | NM_001015051 | -2.03 | 3.77E-04 |
| A_23_P146908 | STX8 | NM_004853 | -2.03 | 7.64E-05 |
| A_24_P506977 | C7orf40 | NR_003697 | -2.03 | 3.90E-04 |
| A_33_P3727762 | SET | NM_003011 | -2.03 | 6.43E-05 |
| A_24_P935986 | BCAT1 | NM_005504 | -2.03 | 1.43E-04 |
| A_33_P3299882 | UQCRB | NM_001199975 | -2.03 | 7.94E-06 |
| A_32_P150891 | DIAPH3 | NM_001042517 | -2.03 | 1.01E-04 |
| A_23_P401547 | PVRL3 | NM_015480 | -2.03 | 6.52E-05 |
| A_32_P79434 | PTPRN2 | NM_002847 | -2.02 | 8.37E-05 |
| A_23_P362759 | PRDM5 | NM_018699 | -2.02 | 9.13E-05 |
| A_23_P121082 | GBE1 | NM_000158 | -2.02 | 4.51E-05 |
| A_33_P3294986 | LIPE | NM_005357 | -2.02 | 1.86E-04 |
| A_33_P3422991 | SLC36A4 | NM_152313 | -2.02 | 5.00E-05 |
| A_23_P216094 | ASPH | NM_004318 | -2.02 | 3.69E-04 |
| A_23_P121082 | GBE1 | NM_000158 | -2.01 | 1.06E-04 |
| A_23_P99027 | PTPN11 | NM_002834 | -2.01 | 2.61E-05 |
| A_24_P148450 | UBE2E3 | NM_006357 | -2.01 | 4.86E-04 |
| A_23_P159027 | ZNF521 | NM_015461 | -2.01 | 2.78E-05 |
| A_23_P108404 | AGAP1 | NM_001037131 | -2.01 | 3.81E-05 |
| A_24_P159948 | ARFRP1 | NM_003224 | -2.01 | 3.10E-05 |
| A_23_P10785 | VTI1A | NM_145206 | -2.01 | 2.27E-04 |
| A_23_P165247 | DAZAP1 | NM_170711 | -2.01 | 2.08E-05 |
| A_23_P115743 | FAM204A | NM_022063 | -2.01 | 6.41E-04 |
| A_23_P20852 | AUH | NM_001698 | -2.01 | 2.90E-04 |
| A_23_P108404 | AGAP1 | NM_001037131 | -2.00 | 7.89E-06 |
| A_19_P00321521 | JPX | NR_024582 | -2.00 | 3.58E-05 |
| A_32_P119248 | FOXD4 | NM_207305 | -2.00 | 5.86E-04 |
| A_33_P3299319 | RPL28 | NM_001136137 | -2.00 | 3.86E-04 |
| A_23_P359854 | BEND3 | NM_001080450 | -2.00 | 9.24E-05 |
| A_23_P99027 | PTPN11 | NM_002834 | -2.00 | 3.81E-06 |
| A_24_P74064 | ZFP161 | NM_003409 | -2.00 | 5.35E-04 |
| A_23_P103631 | EBNA1BP2 | NM_006824 | -2.00 | 8.25E-05 |
| A_21_P0002782 | XLOC_002877 | ENST00000496084 | -2.00 | 3.62E-06 |
| A_24_P914513 | BCKDHB | NM_183050 | -2.00 | 4.87E-04 |
| A_23_P99027 | PTPN11 | NM_002834 | -2.00 | 5.16E-05 |
| A_21_P0000684 | LOC100506548 | NR_037665 | -2.00 | 1.85E-04 |
| A_23_P103631 | EBNA1BP2 | NM_006824 | -2.00 | 4.14E-05 |
| A_19_P00801634 | NOP56 | NM_006392 | -1.99 | 1.13E-04 |
| A_23_P122531 | C6orf48 | NM_001040437 | -1.99 | 1.15E-04 |
| A_23_P108404 | AGAP1 | NM_001037131 | -1.99 | 3.73E-04 |
| A_24_P681011 | HIPK2 | NM_022740 | -1.99 | 1.47E-05 |
| A_23_P201567 | PRPF38B | NM_018061 | -1.99 | 3.05E-05 |
| A_23_P427703 | MT1L | NR_001447 | -1.99 | 1.02E-04 |
| A_23_P251927 | CHCHD3 | NM_017812 | -1.99 | 2.53E-05 |
| A_23_P356677 | PDE10A | NM_006661 | -1.99 | 3.41E-04 |
| A_32_P74955 | ARID2 | NM_152641 | -1.99 | 1.93E-04 |
| A_21_P0000251 | SNORA65 | NR_002449 | -1.98 | 4.20E-04 |
| A_23_P145606 | CHRM2 | NM_001006630 | -1.98 | 2.46E-04 |
| A_23_P212617 | TFRC | NM_003234 | -1.98 | 7.81E-05 |
| A_23_P120472 | TFAP2C | NM_003222 | -1.98 | 2.02E-04 |
| A_24_P235400 | MMP16 | NM_005941 | -1.98 | 5.00E-04 |
| A_21_P0012979 | XLOC_l2_011987 | ENST00000514048 | -1.98 | 8.95E-05 |
| A_33_P3260605 | CTNNAL1 | NM_003798 | -1.98 | 7.59E-04 |
| A_23_P23443 | EFHD2 | NM_024329 | -1.98 | 7.74E-06 |
| A_23_P123424 | CHRNB3 | NM_000749 | -1.98 | 7.92E-04 |
| A_23_P28015 | ZNF558 | NM_144693 | -1.97 | 1.32E-04 |
| A_23_P134809 | NSMAF | NM_003580 | -1.97 | 7.23E-05 |
| A_32_P466514 | IRF2BPL | NM_024496 | -1.97 | 8.15E-04 |
| A_24_P84008 | AI198876 | AI198876 | -1.97 | 7.55E-05 |
| A_21_P0000660 | LOC100499489 | NR_036533 | -1.97 | 9.11E-05 |
| A_24_P56363 | CAB39L | NM_030925 | -1.97 | 5.16E-05 |
| A_21_P0000237 | SNORA10 | NR_002327 | -1.97 | 3.95E-05 |
| A_21_P0013088 | SMS | NM_004595 | -1.97 | 3.84E-05 |
| A_23_P121637 | PRSS12 | NM_003619 | -1.97 | 3.35E-04 |
| A_23_P120472 | TFAP2C | NM_003222 | -1.97 | 5.08E-04 |
| A_23_P132874 | C3orf26 | NM_032359 | -1.97 | 2.77E-05 |
| A_19_P00319503 | ENST00000451884 | ENST00000451884 | -1.97 | 8.84E-05 |
| A_23_P103631 | EBNA1BP2 | NM_006824 | -1.97 | 2.34E-05 |
| A_33_P3290124 | ASB10 | NM_001142459 | -1.97 | 8.72E-05 |
| A_23_P212617 | TFRC | NM_003234 | -1.96 | 6.20E-05 |
| A_23_P120472 | TFAP2C | NM_003222 | -1.96 | 3.85E-04 |
| A_24_P706314 | HNRNPA3 | NM_194247 | -1.96 | 5.50E-05 |
| A_23_P108404 | AGAP1 | NM_001037131 | -1.96 | 2.54E-04 |
| A_23_P9894 | PRMT3 | NM_005788 | -1.96 | 6.46E-04 |
| A_33_P3601163 | LOC147727 | NR_024333 | -1.96 | 3.05E-05 |
| A_19_P00320132 | JPX | NR_024582 | -1.96 | 8.86E-04 |
| A_23_P115636 | TRDMT1 | NM_004412 | -1.96 | 5.32E-06 |
| A_23_P65830 | HDDC3 | NM_198527 | -1.95 | 3.28E-04 |
| A_23_P37983 | MT1B | NM_005947 | -1.95 | 2.21E-04 |
| A_24_P166663 | CDK6 | NM_001259 | -1.95 | 8.85E-05 |
| A_23_P162719 | DIAPH3 | NM_030932 | -1.95 | 7.60E-04 |
| A_24_P79712 | FAM36A | NM_198076 | -1.95 | 9.19E-04 |
| A_24_P183128 | PLAC8 | NM_016619 | -1.95 | 8.90E-05 |
| A_24_P941051 | CSTF2T | NM_015235 | -1.95 | 5.46E-05 |
| A_24_P379512 | PIGK | NM_005482 | -1.95 | 7.28E-04 |
| A_33_P3387646 | LOC643201 | NR_036494 | -1.94 | 1.36E-04 |
| A_32_P44568 | LDHA | NM_005566 | -1.94 | 1.06E-05 |
| A_33_P3357620 | C16orf53 | NM_024516 | -1.94 | 1.43E-04 |
| A_23_P417113 | RBM33 | ENST00000287912 | -1.94 | 3.53E-04 |
| A_23_P108404 | AGAP1 | NM_001037131 | -1.94 | 1.90E-04 |
| A_23_P212617 | TFRC | NM_003234 | -1.94 | 4.50E-05 |
| A_24_P145911 | TRA2B | ENST00000342294 | -1.94 | 8.46E-05 |
| A_33_P3364869 | NAMPT | AK023341 | -1.94 | 5.89E-05 |
| A_21_P0010565 | SMS | NM_004595 | -1.94 | 3.21E-05 |
| A_23_P162525 | UTP20 | NM_014503 | -1.94 | 3.10E-04 |
| A_23_P73012 | C9orf3 | NM_032823 | -1.94 | 1.35E-04 |
| A_23_P212617 | TFRC | NM_003234 | -1.93 | 6.45E-05 |
| A_23_P108404 | AGAP1 | NM_001037131 | -1.93 | 6.96E-05 |
| A_23_P256641 | KCNE1L | NM_012282 | -1.93 | 1.15E-04 |
| A_24_P176255 | GABPB1 | NM_005254 | -1.93 | 3.31E-04 |
| A_21_P0013303 | SEPT7P2 | NR_024271 | -1.93 | 4.58E-05 |
| A_23_P354387 | MYOF | NM_013451 | -1.93 | 1.05E-04 |
| A_33_P3262515 | RASA3 | NM_007368 | -1.93 | 7.64E-05 |
| A_23_P48307 | PABPC3 | NM_030979 | -1.93 | 1.64E-04 |
| A_19_P00322220 | LOC388796 | NR_027241 | -1.93 | 4.21E-04 |
| A_19_P00322336 | JPX | NR_024582 | -1.93 | 3.18E-04 |
| A_33_P3216150 | PRB4 | NM_002723 | -1.93 | 2.28E-04 |
| A_21_P0010986 | U2AF1 | NM_001025204 | -1.92 | 2.25E-04 |
| A_23_P115636 | TRDMT1 | NM_004412 | -1.92 | 6.20E-05 |
| A_33_P3285987 | METTL15 | NM_152636 | -1.92 | 7.02E-04 |
| A_33_P3373765 | DRD4 | NM_000797 | -1.92 | 8.59E-04 |
| A_33_P3344733 | NUBPL | NM_025152 | -1.92 | 2.50E-04 |
| A_24_P224998 | ENST00000392994 | ENST00000392994 | -1.92 | 8.30E-04 |
| A_21_P0002483 | LOC100506473 | XR_109933 | -1.92 | 9.35E-05 |
| A_23_P115636 | TRDMT1 | NM_004412 | -1.92 | 9.08E-04 |
| A_23_P212617 | TFRC | NM_003234 | -1.92 | 3.03E-04 |
| A_23_P13524 | TMEM126A | NM_032273 | -1.92 | 2.37E-05 |
| A_23_P212617 | TFRC | NM_003234 | -1.91 | 3.71E-04 |
| A_19_P00317824 | P39193 | ENST00000415215 | -1.91 | 2.75E-04 |
| A_19_P00807411 | RNF180 | NM_001113561 | -1.91 | 9.27E-04 |
| A_23_P212617 | TFRC | NM_003234 | -1.91 | 6.12E-05 |
| A_23_P115636 | TRDMT1 | NM_004412 | -1.91 | 1.12E-05 |
| A_24_P316305 | AQR | ENST00000156471 | -1.91 | 3.69E-05 |
| A_23_P105436 | PTPN11 | NM_002834 | -1.91 | 1.99E-04 |
| A_23_P204782 | MDM1 | NM_020128 | -1.91 | 3.55E-04 |
| A_23_P54840 | MT1A | NM_005946 | -1.91 | 2.29E-04 |
| A_23_P143958 | RPL22L1 | NM_001099645 | -1.91 | 5.59E-04 |
| A_23_P115636 | TRDMT1 | NM_004412 | -1.91 | 1.03E-04 |
| A_21_P0000302 | SNORA8 | NR_002920 | -1.90 | 8.78E-04 |
| A_33_P3290924 | BAG1 | NM_001172415 | -1.90 | 1.09E-04 |
| A_23_P132644 | NCEH1 | NM_020792 | -1.90 | 7.89E-04 |
| A_24_P346855 | MKI67 | NM_002417 | -1.90 | 2.90E-04 |
| A_23_P115636 | TRDMT1 | NM_004412 | -1.90 | 1.39E-04 |
| A_21_P0014103 | LOC100287375 | XR_108450 | -1.90 | 4.33E-04 |
| A_23_P218068 | PLEKHA5 | NM_019012 | -1.90 | 4.02E-05 |
| A_23_P108404 | AGAP1 | NM_001037131 | -1.90 | 2.25E-04 |
| A_33_P3332081 | KHDRBS3 | NM_006558 | -1.90 | 3.22E-04 |
| A_23_P108404 | AGAP1 | NM_001037131 | -1.90 | 5.15E-04 |
| A_23_P114210 | POU3F4 | NM_000307 | -1.90 | 6.20E-04 |
| A_33_P3219090 | INSIG1 | NM_005542 | -1.90 | 3.08E-04 |
| A_33_P3378925 | RBM14 | NM_006328 | -1.90 | 2.33E-04 |
| A_21_P0000130 | LOC100289187 | NM_001195541 | -1.90 | 1.66E-04 |
| A_33_P3342126 | PRDM8 | NM_020226 | -1.89 | 4.79E-04 |
| A_23_P86660 | HNRNPA3 | NM_194247 | -1.89 | 1.38E-04 |
| A_33_P3366124 | THC2624074 | THC2624074 | -1.89 | 3.15E-04 |
| A_24_P46093 | SLC6A6 | NM_003043 | -1.89 | 7.06E-05 |
| A_19_P00321613 | JPX | NR_024582 | -1.89 | 4.83E-05 |
| A_33_P3886707 | PPA2 | NM_176869 | -1.89 | 2.05E-06 |
| A_21_P0013259 | LOC100289187 | NM_001195541 | -1.89 | 2.11E-04 |
| A_23_P356004 | KCNIP3 | NM_013434 | -1.89 | 1.16E-04 |
| A_19_P00321618 | LINC00277 | NR_026949 | -1.88 | 4.29E-04 |
| A_19_P00318304 | LOC388796 | NR_027241 | -1.88 | 5.85E-05 |
| A_23_P91657 | EWSR1 | NM_013986 | -1.88 | 8.11E-04 |
| A_23_P82693 | PABPC1 | NM_002568 | -1.88 | 2.44E-04 |
| A_24_P278747 | CCND2 | NM_001759 | -1.88 | 9.57E-04 |
| A_21_P0008807 | XLOC_011157 | THC2577654 | -1.88 | 5.06E-04 |
| A_23_P10785 | VTI1A | NM_145206 | -1.88 | 9.21E-04 |
| A_23_P155765 | HMGB2 | NM_002129 | -1.88 | 2.31E-05 |
| A_21_P0000181 | CAMTA1 | NM_001242701 | -1.88 | 2.46E-04 |
| A_21_P0000247 | SNORD18C | NR_002443 | -1.88 | 7.01E-04 |
| A_33_P3212615 | TFPI | NM_006287 | -1.88 | 6.20E-05 |
| A_19_P00319050 | ENST00000443467 | ENST00000443467 | -1.88 | 7.67E-06 |
| A_23_P54055 | AJUBA | NM_032876 | -1.88 | 2.18E-04 |
| A_23_P47565 | LDHA | NM_005566 | -1.88 | 1.13E-06 |
| A_23_P212617 | TFRC | NM_003234 | -1.88 | 2.42E-04 |
| A_24_P31235 | EIF5A | NM_001970 | -1.88 | 1.93E-04 |
| A_23_P115636 | TRDMT1 | NM_004412 | -1.88 | 2.83E-05 |
| A_32_P31182 | RPL7 | NM_000971 | -1.88 | 4.54E-06 |
| A_21_P0000135 | ANKHD1 | NM_024668 | -1.87 | 7.38E-04 |
| A_23_P216568 | FAM206A | NM_017832 | -1.87 | 2.59E-04 |
| A_19_P00320719 | MDM4 | NM_002393 | -1.87 | 2.29E-05 |
| A_23_P423864 | PHC2 | NM_198040 | -1.87 | 6.22E-06 |
| A_23_P212617 | TFRC | NM_003234 | -1.87 | 3.09E-04 |
| A_23_P212617 | TFRC | NM_003234 | -1.87 | 2.00E-04 |
| A_23_P397293 | LY6K | NM_017527 | -1.87 | 2.90E-05 |
| A_24_P309415 | TMEM123 | NM_052932 | -1.87 | 6.30E-05 |
| A_23_P96542 | VMA21 | NM_001017980 | -1.87 | 3.22E-05 |
| A_24_P91916 | NXT2 | NM_018698 | -1.87 | 3.87E-04 |
| A_33_P3361681 | VMA21 | NM_001017980 | -1.87 | 2.58E-04 |
| A_24_P336759 | MCL1 | NM_021960 | -1.87 | 5.03E-06 |
| A_23_P434944 | ILF3 | NM_004516 | -1.87 | 3.72E-04 |
| A_24_P37519 | LZTFL1 | NM_020347 | -1.87 | 2.67E-06 |
| A_23_P325075 | RNGTT | NM_003800 | -1.86 | 3.50E-05 |
| A_23_P168771 | CCDC146 | NM_020879 | -1.86 | 8.79E-04 |
| A_24_P44341 | UGGT2 | NM_020121 | -1.86 | 4.48E-04 |
| A_19_P00805840 | ZNF37BP | NR_026777 | -1.86 | 6.13E-05 |
| A_33_P3318292 | SFPQ | NM_005066 | -1.86 | 6.21E-04 |
| A_24_P345846 | ANTXR2 | NM_058172 | -1.86 | 9.16E-04 |
| A_23_P91590 | RANBP1 | NM_002882 | -1.86 | 1.60E-04 |
| A_24_P396720 | PPP1CB | NM_002709 | -1.86 | 3.65E-04 |
| A_23_P10785 | VTI1A | NM_145206 | -1.86 | 3.84E-05 |
| A_24_P82419 | H3F3B | NM_005324 | -1.86 | 3.06E-05 |
| A_21_P0013574 | MTHFD1L | NM_001242767 | -1.86 | 4.58E-05 |
| A_23_P137391 | ENO1 | NM_001428 | -1.86 | 3.11E-06 |
| A_19_P00317793 | LOC388796 | NR_027241 | -1.86 | 5.93E-04 |
| A_23_P10785 | VTI1A | NM_145206 | -1.85 | 4.01E-04 |
| A_23_P152218 | E2F4 | NM_001950 | -1.85 | 4.74E-05 |
| A_24_P925062 | MXRA7 | NM_001008528 | -1.85 | 2.59E-05 |
| A_23_P108404 | AGAP1 | NM_001037131 | -1.85 | 3.69E-05 |
| A_23_P10785 | VTI1A | NM_145206 | -1.85 | 8.54E-04 |
| A_24_P207150 | UBE3A | NM_130839 | -1.85 | 1.95E-04 |
| A_24_P47547 | RAN | NM_006325 | -1.85 | 2.01E-04 |
| A_23_P205046 | ANKRD10 | NM_017664 | -1.85 | 4.96E-04 |
| A_24_P465772 | RPSAP52 | NR_026825 | -1.85 | 4.08E-06 |
| A_23_P15864 | MBD2 | NM_003927 | -1.85 | 2.32E-04 |
| A_23_P321959 | SFT2D1 | NM_145169 | -1.85 | 4.52E-05 |
| A_21_P0013516 | XLOC_l2_014518 | TCONS_l2_00028204 | -1.85 | 2.14E-05 |
| A_21_P0002865 | XLOC_003176 | ENST00000479244 | -1.85 | 3.86E-04 |
| A_33_P3250953 | SLC35B4 | NM_032826 | -1.85 | 8.93E-05 |
| A_19_P00810043 | FLJ38717 | XR_108650 | -1.84 | 4.56E-05 |
| A_23_P120472 | TFAP2C | NM_003222 | -1.84 | 5.55E-04 |
| A_33_P3251462 | C20orf141 | NM_080739 | -1.84 | 7.58E-05 |
| A_33_P3319155 | ACP1 | NM_001040649 | -1.84 | 1.73E-05 |
| A_33_P3328609 | PKP4 | NM_003628 | -1.84 | 3.61E-05 |
| A_23_P59528 | ACN9 | NM_020186 | -1.84 | 2.11E-04 |
| A_23_P120472 | TFAP2C | NM_003222 | -1.84 | 5.75E-04 |
| A_23_P208389 | AXL | NM_021913 | -1.84 | 2.60E-04 |
| A_33_P3403117 | NR2F1 | NM_005654 | -1.84 | 1.72E-04 |
| A_19_P00323041 | P39193 | ENST00000415215 | -1.84 | 2.60E-04 |
| A_23_P119418 | C19orf2 | NM_003796 | -1.84 | 1.19E-04 |
| A_23_P339079 | ZNF573 | NM_152360 | -1.84 | 1.54E-04 |
| A_23_P121637 | PRSS12 | NM_003619 | -1.84 | 7.38E-04 |
| A_23_P17512 | DTD1 | NM_080820 | -1.84 | 8.15E-05 |
| A_23_P120472 | TFAP2C | NM_003222 | -1.84 | 7.25E-04 |
| A_23_P38167 | GPRC5C | NM_022036 | -1.84 | 3.19E-04 |
| A_24_P500891 | AK2 | NM_013411 | -1.84 | 3.94E-05 |
| A_33_P3296193 | PSIMCT-1 | NR_003677 | -1.84 | 5.20E-04 |
| A_21_P0014871 | LOC727803 | XR_132766 | -1.83 | 4.25E-05 |
| A_33_P3384260 | HNRNPA1 | ENST00000546500 | -1.83 | 2.90E-04 |
| A_32_P162250 | ARHGAP18 | NM_033515 | -1.83 | 9.21E-04 |
| A_33_P3407324 | GULP1 | NM_016315 | -1.83 | 2.73E-04 |
| A_23_P150255 | RBM14 | NM_006328 | -1.83 | 1.25E-04 |
| A_33_P3250750 | CU677518 | CU677518 | -1.83 | 7.53E-04 |
| A_19_P00805273 | PSMD14 | NM_005805 | -1.83 | 3.86E-04 |
| A_19_P00322967 | LOC388796 | NR_027241 | -1.83 | 8.28E-04 |
| A_24_P349743 | RPSA | NM_002295 | -1.83 | 1.76E-05 |
| A_32_P23125 | LINC00261 | NR_001558 | -1.83 | 3.21E-06 |
| A_24_P340976 | THC2530888 | THC2530888 | -1.83 | 5.67E-05 |
| A_23_P92629 | CWC27 | NM_005869 | -1.83 | 9.94E-05 |
| A_33_P3317198 | GTF2F2 | NM_004128 | -1.83 | 7.35E-05 |
| A_23_P51906 | PFDN2 | NM_012394 | -1.83 | 7.45E-05 |
| A_19_P00807615 | XLOC_l2_015397 | BC014023 | -1.83 | 6.31E-05 |
| A_21_P0000530 | C17orf76-AS1 | NR_045024 | -1.83 | 3.64E-04 |
| A_33_P3214879 | KPNA1 | NM_002264 | -1.83 | 3.86E-04 |
| A_24_P860703 | LOC388796 | NR_015366 | -1.83 | 5.82E-04 |
| A_33_P3325306 | HSPE1 | ENST00000463841 | -1.83 | 1.68E-04 |
| A_24_P235049 | MTHFD1L | NM_015440 | -1.83 | 1.95E-05 |
| A_24_P336705 | RABGGTB | NM_004582 | -1.83 | 2.14E-05 |
| A_33_P3262890 | THC2556858 | THC2556858 | -1.83 | 2.90E-06 |
| A_33_P3245922 | MAP3K4 | NM_006724 | -1.82 | 1.74E-05 |
| A_19_P00806320 | ARPC2 | NM_152862 | -1.82 | 6.63E-05 |
| A_21_P0010069 | XLOC_013835 | THC2751630 | -1.82 | 8.38E-04 |
| A_33_P3290919 | BAG1 | NM_001172415 | -1.82 | 1.17E-04 |
| A_24_P382026 | MIF4GD | NM_020679 | -1.82 | 5.86E-04 |
| A_24_P916378 | HNRPLL | ENST00000410076 | -1.82 | 8.92E-04 |
| A_24_P780052 | RPSA | NM_002295 | -1.82 | 9.05E-06 |
| A_32_P82475 | SNHG10 | NR_003138 | -1.82 | 1.83E-04 |
| A_23_P138125 | FAIM3 | NM_005449 | -1.82 | 6.42E-04 |
| A_23_P93282 | HIST1H3J | NM_003535 | -1.82 | 4.84E-05 |
| A_33_P3429575 | LOC643454 | BC157883 | -1.82 | 2.02E-05 |
| A_21_P0011135 | EIF4A1 | NM_001416 | -1.82 | 2.71E-05 |
| A_23_P56922 | HSPE1 | NM_002157 | -1.82 | 9.94E-05 |
| A_33_P3272563 | NMT2 | NM_004808 | -1.82 | 3.23E-05 |
| A_23_P121637 | PRSS12 | NM_003619 | -1.82 | 4.60E-04 |
| A_23_P149200 | CDC20 | NM_001255 | -1.82 | 2.90E-04 |
| A_23_P18939 | RASA1 | NM_002890 | -1.81 | 3.81E-05 |
| A_24_P54485 | CCDC115 | NM_032357 | -1.81 | 3.63E-05 |
| A_23_P160862 | HNRNPU | NM_031844 | -1.81 | 4.94E-05 |
| A_24_P291973 | Sep7 | NM_001011553 | -1.81 | 3.20E-04 |
| A_23_P500381 | HTR7 | NM_019859 | -1.81 | 1.62E-04 |
| A_32_P92505 | LCLAT1 | NM_182551 | -1.81 | 3.88E-04 |
| A_23_P120472 | TFAP2C | NM_003222 | -1.81 | 4.01E-04 |
| A_23_P79927 | NOP56 | NM_006392 | -1.80 | 1.73E-04 |
| A_24_P850428 | ENST00000494591 | ENST00000494591 | -1.80 | 3.01E-05 |
| A_24_P832426 | B3GALTL | NM_194318 | -1.80 | 9.80E-04 |
| A_32_P73821 | CSDE1 | NM_001007553 | -1.80 | 2.92E-04 |
| A_24_P320254 | HNRNPH1 | NM_005520 | -1.80 | 1.67E-04 |
| A_24_P101391 | YBX1 | NM_004559 | -1.80 | 7.15E-05 |
| A_24_P942030 | VAMP4 | NM_003762 | -1.80 | 1.68E-04 |
| A_33_P3414789 | FSD1 | NM_024333 | -1.80 | 6.96E-04 |
| A_23_P158725 | SLC16A3 | NM_001042422 | -1.80 | 4.45E-04 |
| A_33_P3375934 | NAMPT | NM_005746 | -1.80 | 3.76E-04 |
| A_23_P146654 | BAG1 | NM_004323 | -1.79 | 1.05E-05 |
| A_24_P101402 | NOP56 | NM_006392 | -1.79 | 4.80E-05 |
| A_24_P381029 | GLRX3 | NM_006541 | -1.79 | 5.05E-04 |
| A_23_P205098 | PDS5B | NM_015032 | -1.79 | 1.64E-04 |
| A_23_P128574 | ENOX1 | NM_017993 | -1.79 | 4.04E-06 |
| A_33_P3238177 | ZNF37A | NM_001007094 | -1.79 | 2.06E-04 |
| A_24_P169148 | HMGB1 | NM_002128 | -1.79 | 6.32E-05 |
| A_23_P390744 | FOPNL | NM_144600 | -1.79 | 7.86E-04 |
| A_21_P0012935 | XLOC_l2_012323 | THC2679226 | -1.79 | 7.05E-04 |
| A_23_P120557 | TASP1 | NM_017714 | -1.79 | 2.45E-04 |
| A_33_P3292332 | KIF5B | NM_004521 | -1.79 | 1.97E-04 |
| A_23_P10785 | VTI1A | NM_145206 | -1.79 | 1.02E-05 |
| A_23_P5568 | SFT2D3 | NM_032740 | -1.79 | 1.82E-04 |
| A_23_P307328 | WHSC1 | NM_007331 | -1.79 | 5.90E-04 |
| A_33_P3297517 | TRA2B | NM_001243879 | -1.79 | 6.43E-04 |
| A_24_P244410 | C11orf51 | NM_014042 | -1.79 | 9.58E-05 |
| A_21_P0006914 | XLOC_008617 | AL049387 | -1.79 | 2.77E-04 |
| A_19_P00812250 | SMAD4 | NM_005359 | -1.78 | 4.70E-04 |
| A_24_P11575 | CRIM1 | NM_016441 | -1.78 | 4.63E-04 |
| A_23_P160582 | HYI | NM_031207 | -1.78 | 2.00E-04 |
| A_24_P376707 | HDGF | NM_004494 | -1.78 | 8.09E-06 |
| A_33_P3866631 | DKFZP564C152 | AL049980 | -1.78 | 7.73E-04 |
| A_33_P3380056 | C9orf30 | NM_001198807 | -1.78 | 5.53E-04 |
| A_21_P0014664 | LOC100505874 | XR_110949 | -1.78 | 6.99E-05 |
| A_23_P332399 | GULP1 | NM_016315 | -1.78 | 2.38E-05 |
| A_24_P713668 | BZW1 | NM_014670 | -1.78 | 2.35E-05 |
| A_23_P121637 | PRSS12 | NM_003619 | -1.78 | 9.71E-04 |
| A_33_P3334548 | BZW1 | NM_001207068 | -1.78 | 3.85E-04 |
| A_23_P8482 | Sep7 | NM_001011553 | -1.78 | 1.40E-04 |
| A_33_P3405500 | SACS | NM_014363 | -1.78 | 1.68E-04 |
| A_24_P315921 | ENST00000331856 | ENST00000331856 | -1.78 | 3.72E-04 |
| A_33_P3219240 | LOC642980 | AK131413 | -1.78 | 1.04E-04 |
| A_33_P3329834 | PRPF38B | NM_018061 | -1.77 | 8.47E-05 |
| A_23_P115636 | TRDMT1 | NM_004412 | -1.77 | 4.43E-04 |
| A_32_P105083 | FONG | NR_034096 | -1.77 | 3.64E-04 |
| A_33_P3215113 | LDOC1 | NM_012317 | -1.77 | 2.94E-05 |
| A_24_P82880 | TPM4 | NM_003290 | -1.77 | 8.52E-06 |
| A_23_P214037 | NPM1 | NM_002520 | -1.77 | 3.18E-05 |
| A_33_P3257643 | C17orf104 | ENST00000409464 | -1.77 | 1.00E-03 |
| A_33_P3269678 | LOC541471 | NR_015395 | -1.77 | 2.94E-04 |
| A_19_P00319623 | JPX | NR_024582 | -1.77 | 3.77E-04 |
| A_23_P42096 | MAP3K4 | NM_005922 | -1.77 | 3.39E-04 |
| A_32_P218989 | YBX1 | NM_004559 | -1.77 | 3.01E-05 |
| A_21_P0000367 | SNORA32 | NR_003032 | -1.77 | 2.44E-04 |
| A_32_P133884 | TUSC1 | NM_001004125 | -1.77 | 9.51E-04 |
| A_32_P8402 | SYNCRIP | NM_006372 | -1.77 | 4.82E-04 |
| A_23_P141974 | TPM4 | NM_003290 | -1.77 | 2.06E-07 |
| A_23_P39955 | ACTG2 | NM_001615 | -1.77 | 6.35E-04 |
| A_33_P3305158 | ZNF621 | NM_198484 | -1.77 | 1.88E-04 |
| A_24_P358164 | RPSA | NM_002295 | -1.77 | 9.96E-07 |
| A_24_P142473 | CTDNEP1 | NM_015343 | -1.77 | 3.91E-05 |
| A_21_P0014637 | LOC100506778 | XR_110850 | -1.76 | 1.26E-04 |
| A_32_P226149 | YWHAZ | NM_145690 | -1.76 | 6.87E-05 |
| A_24_P188941 | NPM1 | NM_002520 | -1.76 | 8.21E-07 |
| A_23_P31903 | VPS28 | NM_183057 | -1.76 | 2.40E-05 |
| A_23_P102122 | ARPC2 | NM_152862 | -1.76 | 1.77E-05 |
| A_24_P412734 | PRSS36 | NM_173502 | -1.76 | 4.01E-04 |
| A_23_P23114 | PTP4A2 | NM_080391 | -1.76 | 7.30E-05 |
| A_23_P6303 | U2AF1 | NM_001025204 | -1.76 | 4.12E-04 |
| A_23_P165691 | PSMD14 | NM_005805 | -1.76 | 8.98E-04 |
| A_23_P141362 | FZD2 | NM_001466 | -1.76 | 1.41E-04 |
| A_32_P506600 | RAN | NM_006325 | -1.76 | 3.14E-04 |
| A_23_P51508 | DUSP12 | NM_007240 | -1.76 | 8.20E-04 |
| A_23_P209449 | FZD7 | NM_003507 | -1.76 | 8.08E-04 |
| A_23_P430201 | CEP128 | NM_152446 | -1.76 | 2.54E-04 |
| A_24_P365515 | FOXA2 | NM_021784 | -1.76 | 8.55E-04 |
| A_23_P136964 | RPGR | NM_000328 | -1.76 | 8.92E-05 |
| A_21_P0013192 | Sep7 | NM_001788 | -1.75 | 3.16E-05 |
| A_23_P52531 | FAM24B | NM_152644 | -1.75 | 1.13E-05 |
| A_33_P3414422 | GPHN | NM_020806 | -1.75 | 1.84E-04 |
| A_32_P235159 | MSL3P1 | NR_024322 | -1.75 | 8.07E-04 |
| A_19_P00800467 | CLEC2D | NM_001004419 | -1.75 | 2.21E-04 |
| A_33_P3423721 | JPH3 | ENST00000301008 | -1.75 | 7.58E-05 |
| A_23_P16992 | PKP4 | NM_003628 | -1.75 | 1.71E-04 |
| A_24_P305678 | PITPNB | NM_012399 | -1.75 | 2.06E-04 |
| A_23_P121637 | PRSS12 | NM_003619 | -1.75 | 5.36E-04 |
| A_32_P7204 | RAB28 | NM_004249 | -1.75 | 9.69E-04 |
| A_33_P3232828 | SRSF3 | NM_003017 | -1.75 | 6.53E-05 |
| A_23_P204052 | PCBP2 | NM_031989 | -1.75 | 1.55E-04 |
| A_23_P376239 | PAPOLA | NM_032632 | -1.75 | 9.91E-04 |
| A_33_P3361393 | EIF1AX | NM_001412 | -1.75 | 1.94E-04 |
| A_23_P204472 | RPLP0 | NM_053275 | -1.75 | 5.31E-06 |
| A_23_P87902 | DYRK4 | NM_003845 | -1.75 | 1.38E-04 |
| A_21_P0000910 | LOC284628 | AK094692 | -1.75 | 7.94E-05 |
| A_19_P00321364 | JPX | NR_024582 | -1.75 | 1.90E-04 |
| A_23_P58353 | HNRNPD | NM_031370 | -1.75 | 8.57E-05 |
| A_23_P56798 | ACP1 | NM_004300 | -1.75 | 6.83E-04 |
| A_23_P503233 | EDARADD | NM_080738 | -1.75 | 9.60E-05 |
| A_33_P3368049 | LOC643988 | NM_001242659 | -1.75 | 2.20E-04 |
| A_33_P3316026 | USF2 | NM_003367 | -1.74 | 1.07E-06 |
| A_23_P385938 | PAN3 | NM_175854 | -1.74 | 1.35E-04 |
| A_23_P167841 | KCNQ5 | NM_019842 | -1.74 | 6.85E-04 |
| A_33_P3229256 | NT5C | NM_014595 | -1.74 | 1.96E-04 |
| A_33_P3296479 | APP | NM_001204303 | -1.74 | 2.25E-04 |
| A_21_P0008884 | CRNDE | FJ466686 | -1.74 | 2.08E-04 |
| A_24_P25080 | CBWD5 | NM_001024916 | -1.74 | 4.74E-04 |
| A_32_P149432 | EIF4A1 | NM_001416 | -1.74 | 1.82E-04 |
| A_23_P105664 | CCDC59 | NM_014167 | -1.74 | 8.83E-05 |
| A_21_P0011010 | XLOC_l2_002651 | TCONS_l2_00005011 | -1.74 | 1.66E-04 |
| A_23_P30884 | CLIC1 | NM_001288 | -1.74 | 2.52E-04 |
| A_23_P121637 | PRSS12 | NM_003619 | -1.74 | 5.06E-04 |
| A_23_P17074 | CCDC115 | NM_032357 | -1.74 | 1.74E-04 |
| A_33_P3285444 | TERF1 | NM_017489 | -1.74 | 6.14E-04 |
| A_33_P3341239 | CDK16 | NM_006201 | -1.74 | 8.46E-06 |
| A_23_P128828 | PABPN1 | NM_004643 | -1.74 | 9.58E-06 |
| A_23_P139471 | RPS26 | NM_001029 | -1.74 | 1.59E-04 |
| A_24_P15765 | RPS7P5 | NR_036695 | -1.74 | 7.68E-05 |
| A_24_P69691 | ZNF25 | NM_145011 | -1.74 | 6.34E-04 |
| A_33_P3370226 | RPL7 | NM_000971 | -1.74 | 4.70E-05 |
| A_23_P126197 | SRSF4 | NM_005626 | -1.74 | 2.50E-04 |
| A_23_P341325 | RPL10L | NM_080746 | -1.74 | 2.89E-06 |
| A_23_P163161 | SDR39U1 | NM_020195 | -1.74 | 1.90E-05 |
| A_23_P121637 | PRSS12 | NM_003619 | -1.74 | 1.84E-04 |
| A_33_P3322654 | RNPEPL1 | NM_018226 | -1.74 | 3.67E-04 |
| A_23_P105368 | AK001057 | AK001057 | -1.73 | 2.69E-04 |
| A_19_P00320640 | ENST00000453317 | ENST00000453317 | -1.73 | 2.83E-04 |
| A_32_P26376 | POGLUT1 | NM_152305 | -1.73 | 1.70E-04 |
| A_21_P0011052 | XLOC_l2_003065 | TCONS_l2_00005785 | -1.73 | 9.18E-05 |
| A_19_P00317504 | LOC100129936 | AK124820 | -1.73 | 8.10E-04 |
| A_23_P377965 | C18orf18 | NR_026849 | -1.73 | 6.16E-04 |
| A_33_P3305472 | AK123993 | AK123993 | -1.73 | 2.42E-04 |
| A_19_P00320914 | JPX | NR_024582 | -1.73 | 8.38E-04 |
| A_23_P165676 | MZT2A | ENST00000491265 | -1.73 | 4.81E-04 |
| A_23_P97394 | BCAR3 | NM_003567 | -1.73 | 9.29E-06 |
| A_32_P183609 | ASB1 | NM_001040445 | -1.73 | 1.80E-05 |
| A_23_P205575 | GPR135 | NM_022571 | -1.73 | 6.85E-05 |
| A_23_P259098 | ZSCAN16 | NM_025231 | -1.73 | 9.18E-05 |
| A_33_P3839897 | RNU4ATAC | DW419002 | -1.73 | 4.91E-04 |
| A_23_P6786 | TRNT1 | ENST00000339437 | -1.73 | 1.50E-04 |
| A_33_P3408054 | HSP90AB2P | NR_003132 | -1.73 | 2.95E-04 |
| A_23_P119418 | C19orf2 | NM_003796 | -1.73 | 6.20E-04 |
| A_23_P105664 | CCDC59 | NM_014167 | -1.73 | 5.28E-06 |
| A_23_P8253 | RAET1E | NM_139165 | -1.73 | 8.00E-04 |
| A_23_P213085 | SLC10A7 | NM_001029998 | -1.73 | 7.37E-04 |
| A_32_P84605 | RPS23 | NM_001025 | -1.72 | 9.25E-04 |
| A_33_P3259615 | TTC36 | NM_001080441 | -1.72 | 6.22E-05 |
| A_33_P3311285 | LMNA | NM_170707 | -1.72 | 7.56E-04 |
| A_23_P107644 | SNRPD1 | NM_006938 | -1.72 | 2.40E-04 |
| A_23_P21436 | PHF19 | NM_015651 | -1.72 | 8.99E-04 |
| A_21_P0000261 | RPL17 | NM_000985 | -1.72 | 3.62E-05 |
| A_23_P252211 | MTRR | NM_024010 | -1.72 | 1.70E-04 |
| A_19_P00316401 | XLOC_007052 | ENST00000518765 | -1.72 | 1.98E-05 |
| A_32_P199252 | HSP90AA1 | NM_001017963 | -1.72 | 5.71E-04 |
| A_23_P46396 | PTBP2 | NM_021190 | -1.72 | 6.33E-05 |
| A_23_P256903 | XR_037268 | XR_037268 | -1.72 | 1.88E-04 |
| A_19_P00803360 | LOC730101 | NR_024403 | -1.72 | 8.17E-04 |
| A_32_P42197 | HNRNPA1 | NM_031157 | -1.72 | 3.34E-04 |
| A_23_P218158 | SMEK1 | NM_032560 | -1.72 | 4.27E-06 |
| A_33_P3364864 | NAMPT | NM_005746 | -1.72 | 4.69E-04 |
| A_33_P3280721 | WLS | NM_001002292 | -1.72 | 1.51E-04 |
| A_23_P105664 | CCDC59 | NM_014167 | -1.72 | 9.42E-04 |
| A_24_P3973 | HNRNPA2B1 | NM_002137 | -1.72 | 1.12E-04 |
| A_24_P362572 | BZW1 | NM_014670 | -1.72 | 4.82E-05 |
| A_23_P93750 | LSM5 | NM_012322 | -1.72 | 3.97E-05 |
| A_23_P200976 | HYI | NM_001190880 | -1.72 | 4.97E-05 |
| A_33_P3237359 | HMGB3 | NM_005342 | -1.72 | 4.04E-04 |
| A_24_P336853 | PNO1 | NM_020143 | -1.72 | 5.39E-04 |
| A_24_P225468 | ANP32E | NM_030920 | -1.71 | 7.65E-04 |
| A_23_P204472 | RPLP0 | NM_053275 | -1.71 | 3.21E-06 |
| A_33_P3311618 | RABGGTB | NM_004582 | -1.71 | 2.41E-04 |
| A_23_P217778 | MSL3 | NM_078629 | -1.71 | 2.01E-04 |
| A_23_P403955 | TARDBP | NM_007375 | -1.71 | 2.03E-04 |
| A_33_P3347168 | TBL1X | NM_005647 | -1.71 | 8.45E-04 |
| A_23_P105664 | CCDC59 | NM_014167 | -1.71 | 4.43E-04 |
| A_23_P254415 | RPSA | NM_002295 | -1.71 | 1.05E-05 |
| A_33_P3210139 | PCF11 | NM_015885 | -1.71 | 2.68E-04 |
| A_24_P389517 | HNRNPK | NM_031262 | -1.71 | 2.05E-04 |
| A_23_P52676 | CATSPER1 | NM_053054 | -1.71 | 8.27E-04 |
| A_33_P3400653 | BNIP2 | ENST00000267859 | -1.71 | 6.84E-05 |
| A_24_P324640 | ZNF544 | NM_014480 | -1.71 | 3.37E-04 |
| A_23_P204472 | RPLP0 | NM_053275 | -1.71 | 3.77E-05 |
| A_21_P0010890 | XLOC_l2_002033 | TCONS_l2_00003643 | -1.71 | 2.39E-04 |
| A_33_P3254695 | RNU105A | NR_004404 | -1.71 | 2.71E-04 |
| A_23_P204472 | RPLP0 | NM_053275 | -1.71 | 4.77E-06 |
| A_23_P137103 | EIF4A1 | NM_001416 | -1.71 | 8.41E-05 |
| A_33_P3320762 | ATXN3 | NM_004993 | -1.71 | 2.30E-04 |
| A_33_P3305571 | TNFRSF6B | NM_003823 | -1.71 | 3.34E-04 |
| A_23_P103433 | OSCP1 | NM_145047 | -1.71 | 3.59E-04 |
| A_23_P434890 | CARD10 | NM_014550 | -1.71 | 6.23E-04 |
| A_33_P3353672 | RAB28 | NM_004249 | -1.71 | 3.52E-05 |
| A_23_P74349 | NUF2 | NM_145697 | -1.71 | 3.06E-04 |
| A_23_P67725 | LMNB2 | NM_032737 | -1.70 | 3.64E-05 |
| A_23_P4679 | ERF | NM_006494 | -1.70 | 3.74E-04 |
| A_21_P0000219 | SNORD34 | NR_000019 | -1.70 | 1.34E-04 |
| A_33_P3766959 | TDP1 | NM_018319 | -1.70 | 6.00E-04 |
| A_23_P74663 | TAF1A | NM_005681 | -1.70 | 3.99E-04 |
| A_23_P204472 | RPLP0 | NM_053275 | -1.70 | 3.62E-05 |
| A_21_P0014443 | LOC100507473 | XR_109833 | -1.70 | 3.04E-04 |
| A_19_P00321795 | P39193 | ENST00000415215 | -1.70 | 5.86E-04 |
| A_23_P74799 | SLC25A24 | NM_213651 | -1.70 | 7.34E-04 |
| A_23_P313632 | FUT8 | NM_178155 | -1.70 | 7.00E-04 |
| A_23_P71530 | TNFRSF11B | NM_002546 | -1.70 | 5.68E-04 |
| A_23_P10785 | VTI1A | NM_145206 | -1.70 | 7.11E-04 |
| A_24_P20630 | LEF1 | NM_016269 | -1.70 | 3.31E-04 |
| A_23_P378526 | RTEL1 | NM_016434 | -1.70 | 5.81E-04 |
| A_23_P20615 | ANP32B | NM_006401 | -1.70 | 4.98E-05 |
| A_23_P121637 | PRSS12 | NM_003619 | -1.70 | 6.39E-04 |
| A_23_P401568 | MAT2A | NM_005911 | -1.70 | 5.77E-06 |
| A_32_P148672 | SNRPD1 | NM_006938 | -1.70 | 1.55E-04 |
| A_23_P102122 | ARPC2 | NM_152862 | -1.70 | 4.38E-05 |
| A_33_P3210904 | SEC13 | AK095629 | -1.70 | 3.83E-04 |
| A_33_P3222105 | EIF1AX | NM_001412 | -1.70 | 6.40E-04 |
| A_23_P204472 | RPLP0 | NM_053275 | -1.70 | 2.01E-05 |
| A_21_P0000707 | FAM24B | NM_152644 | -1.70 | 2.21E-05 |
| A_23_P69958 | AP3S1 | NM_001284 | -1.70 | 7.57E-05 |
| A_21_P0001525 | XLOC_000889 | THC2666840 | -1.69 | 8.90E-04 |
| A_23_P105664 | CCDC59 | NM_014167 | -1.69 | 6.68E-05 |
| A_21_P0000668 | ADAM1 | NR_036636 | -1.69 | 1.81E-04 |
| A_23_P31315 | CBX3 | NM_016587 | -1.69 | 8.82E-06 |
| A_23_P130040 | PHB | NM_002634 | -1.69 | 3.56E-05 |
| A_23_P131139 | DIRC1 | NM_052952 | -1.69 | 1.79E-05 |
| A_23_P148629 | EIF1AY | NM_004681 | -1.69 | 6.69E-04 |
| A_32_P49423 | NPM1 | NM_001037738 | -1.69 | 1.55E-04 |
| A_23_P102122 | ARPC2 | NM_152862 | -1.69 | 1.82E-05 |
| A_32_P80068 | CLEC2D | NM_001004419 | -1.69 | 2.60E-04 |
| A_24_P345993 | CANX | NM_001746 | -1.69 | 7.23E-04 |
| A_21_P0014023 | LOC100288602 | XM_003403504 | -1.69 | 1.38E-04 |
| A_33_P3326285 | GAS5 | NR_002578 | -1.69 | 6.20E-04 |
| A_23_P259451 | LSM6 | NM_007080 | -1.69 | 3.01E-05 |
| A_23_P21409 | CEP63 | NM_001042384 | -1.69 | 2.40E-04 |
| A_33_P3223121 | HIPK2 | NM_001113239 | -1.69 | 1.45E-05 |
| A_33_P3238685 | TET2 | NM_001127208 | -1.69 | 8.02E-04 |
| A_33_P3237784 | PORCN | NM_203473 | -1.69 | 4.66E-04 |
| A_33_P3223116 | HIPK2 | ENST00000342645 | -1.69 | 1.98E-04 |
| A_23_P207058 | SOCS3 | NM_003955 | -1.69 | 2.03E-04 |
| A_21_P0014051 | LOC100132273 | NR_034118 | -1.69 | 6.73E-06 |
| A_24_P29733 | CDK14 | NM_012395 | -1.69 | 5.47E-04 |
| A_24_P35169 | GATAD1 | NM_021167 | -1.69 | 1.80E-05 |
| A_23_P202071 | CELF2 | NM_001025077 | -1.69 | 4.48E-04 |
| A_23_P204472 | RPLP0 | NM_053275 | -1.69 | 1.08E-04 |
| A_24_P16340 | NP1207855 | NP1207855 | -1.69 | 4.41E-05 |
| A_23_P50137 | MEX3C | NM_016626 | -1.69 | 8.60E-05 |
| A_33_P3270636 | SHISA5 | NM_016479 | -1.69 | 1.37E-05 |
| A_23_P204472 | RPLP0 | NM_053275 | -1.69 | 4.94E-05 |
| A_33_P3612589 | ATRX | NM_000489 | -1.69 | 5.18E-04 |
| A_24_P289139 | SH3KBP1 | NM_001024666 | -1.69 | 5.65E-05 |
| A_32_P114896 | PTGES3 | NM_006601 | -1.69 | 1.95E-05 |
| A_23_P300150 | NFATC1 | NM_172387 | -1.69 | 6.67E-05 |
| A_33_P3287028 | Sep7 | NM_001788 | -1.68 | 2.72E-05 |
| A_23_P22671 | VAMP7 | NM_005638 | -1.68 | 3.22E-04 |
| A_23_P418031 | IFFO2 | NM_001136265 | -1.68 | 2.22E-04 |
| A_23_P110473 | NAIP | NM_004536 | -1.68 | 6.98E-04 |
| A_19_P00809682 | BC014023 | BC014023 | -1.68 | 8.10E-05 |
| A_23_P23356 | RRP15 | NM_016052 | -1.68 | 5.67E-04 |
| A_23_P215406 | RAC1 | NM_018890 | -1.68 | 3.42E-05 |
| A_23_P204472 | RPLP0 | NM_053275 | -1.68 | 2.21E-05 |
| A_23_P5551 | NCL | NM_005381 | -1.68 | 1.56E-04 |
| A_23_P59637 | DOCK4 | NM_014705 | -1.68 | 9.38E-05 |
| A_24_P55465 | MTPN | NM_145808 | -1.68 | 1.56E-04 |
| A_23_P215956 | MYC | NM_002467 | -1.68 | 2.17E-05 |
| A_21_P0011712 | XLOC_l2_006815 | TCONS_l2_00012696 | -1.68 | 7.12E-06 |
| A_23_P131935 | FERMT1 | NM_017671 | -1.67 | 6.27E-04 |
| A_24_P400376 | CHCHD2 | NM_016139 | -1.67 | 3.65E-05 |
| A_21_P0000282 | SNORD56 | NR_002739 | -1.67 | 2.39E-04 |
| A_21_P0010647 | ANKRD20A3 | NM_001012419 | -1.67 | 8.16E-04 |
| A_33_P3309924 | HDAC2 | NM_001527 | -1.67 | 2.20E-04 |
| A_33_P3508822 | APP | NM_000484 | -1.67 | 8.13E-04 |
| A_24_P398810 | EIF5 | NM_001969 | -1.67 | 7.77E-04 |
| A_23_P84922 | HDAC8 | NM_018486 | -1.67 | 9.10E-04 |
| A_24_P285623 | DGUOK | NM_080916 | -1.67 | 2.19E-05 |
| A_19_P00809372 | LOC100505634 | XR_109905 | -1.67 | 5.43E-04 |
| A_32_P206698 | CKS1B | NM_001826 | -1.67 | 1.08E-04 |
| A_33_P3286254 | AP3S1 | NM_001284 | -1.67 | 9.69E-05 |
| A_33_P3331242 | G3BP1 | NM_005754 | -1.67 | 6.79E-05 |
| A_33_P3316223 | SNAPC1 | NM_003082 | -1.67 | 2.57E-04 |
| A_33_P3598466 | HSP90AB6P | AY956767 | -1.67 | 3.10E-04 |
| A_33_P3361257 | NOP16 | NM_016391 | -1.67 | 7.15E-04 |
| A_23_P35791 | RPS6KA4 | NM_003942 | -1.67 | 8.82E-05 |
| A_24_P228228 | B4GALT6 | NM_004775 | -1.67 | 5.37E-04 |
| A_21_P0012078 | LOC541471 | NR_015395 | -1.67 | 1.31E-04 |
| A_23_P41716 | GNB2L1 | NM_006098 | -1.67 | 1.81E-04 |
| A_21_P0000666 | LOC100129361 | NR_036583 | -1.67 | 3.98E-05 |
| A_24_P928052 | NRP1 | NM_003873 | -1.67 | 8.16E-05 |
| A_32_P141612 | ORC4 | NM_002552 | -1.67 | 1.35E-04 |
| A_33_P3399373 | TPRA1 | NM_001142646 | -1.67 | 2.53E-05 |
| A_23_P121095 | TOP2B | NM_001068 | -1.67 | 2.55E-05 |
| A_23_P69826 | DHX15 | NM_001358 | -1.67 | 3.42E-04 |
| A_24_P399942 | ATP11C | NM_173694 | -1.67 | 4.94E-04 |
| A_23_P215956 | MYC | NM_002467 | -1.66 | 1.31E-04 |
| A_33_P3374723 | ZEB1 | NM_001128128 | -1.66 | 3.07E-04 |
| A_33_P3416762 | LYPLA1 | NM_006330 | -1.66 | 9.49E-05 |
| A_32_P155364 | RPL7 | NM_000971 | -1.66 | 6.06E-04 |
| A_21_P0000482 | SNORD80 | NR_003940 | -1.66 | 2.66E-04 |
| A_23_P141680 | BCAS3 | NM_017679 | -1.66 | 5.90E-04 |
| A_33_P3668839 | LOC644656 | NR_036539 | -1.66 | 1.32E-05 |
| A_33_P3384825 | CTDNEP1 | NM_015343 | -1.66 | 2.26E-04 |
| A_24_P364296 | STX2 | NM_001980 | -1.66 | 1.49E-05 |
| A_32_P186027 | ANP32A | NM_006305 | -1.66 | 3.78E-04 |
| A_33_P3705884 | LOC148189 | AK094188 | -1.66 | 3.44E-04 |
| A_24_P337657 | SRF | NM_003131 | -1.66 | 1.44E-04 |
| A_23_P114466 | TBL1Y | NM_033284 | -1.66 | 9.89E-05 |
| A_23_P130020 | UTP18 | NM_016001 | -1.66 | 6.62E-05 |
| A_24_P941505 | FAM120A | NM_014612 | -1.66 | 9.07E-07 |
| A_23_P60101 | ZNF696 | NM_030895 | -1.66 | 6.46E-05 |
| A_23_P256223 | VBP1 | NM_003372 | -1.66 | 5.01E-04 |
| A_19_P00322531 | CRNDE | NR_034105 | -1.66 | 1.09E-04 |
| A_23_P215956 | MYC | NM_002467 | -1.66 | 5.90E-05 |
| A_32_P148796 | UBXN2B | NM_001077619 | -1.66 | 6.40E-04 |
| A_23_P162874 | HSP90AA1 | NM_005348 | -1.66 | 5.41E-04 |
| A_23_P125829 | PGK1 | NM_000291 | -1.66 | 1.40E-05 |
| A_23_P105664 | CCDC59 | NM_014167 | -1.66 | 7.97E-04 |
| A_23_P400465 | GTF3C6 | NM_138408 | -1.66 | 6.71E-04 |
| A_23_P82299 | PPP1R35 | NM_145030 | -1.66 | 1.48E-04 |
| A_23_P122001 | MSH3 | NM_002439 | -1.66 | 3.23E-04 |
| A_23_P105664 | CCDC59 | NM_014167 | -1.66 | 3.99E-04 |
| A_23_P309865 | ZNF449 | NM_152695 | -1.66 | 1.19E-04 |
| A_21_P0014771 | LOC100509105 | XR_113070 | -1.66 | 3.68E-04 |
| A_33_P3214129 | LOC728061 | AK025151 | -1.66 | 8.14E-04 |
| A_33_P3397399 | PPP3CB | ENST00000394822 | -1.66 | 4.47E-04 |
| A_21_P0013302 | XLOC_l2_013808 | ENST00000413875 | -1.66 | 3.17E-04 |
| A_23_P104025 | TSEN15 | NM_052965 | -1.66 | 1.43E-05 |
| A_24_P538478 | MED28 | NM_025205 | -1.66 | 6.41E-04 |
| A_23_P162874 | HSP90AA1 | NM_005348 | -1.66 | 5.58E-04 |
| A_23_P365817 | PPP1R14B | NM_138689 | -1.66 | 1.12E-04 |
| A_23_P405942 | LARP4B | NM_015155 | -1.65 | 1.31E-04 |
| A_24_P328872 | CCT5 | NM_012073 | -1.65 | 9.07E-04 |
| A_23_P125829 | PGK1 | NM_000291 | -1.65 | 1.99E-04 |
| A_23_P114466 | TBL1Y | NM_033284 | -1.65 | 5.06E-04 |
| A_23_P102122 | ARPC2 | NM_152862 | -1.65 | 7.90E-08 |
| A_23_P125829 | PGK1 | NM_000291 | -1.65 | 7.22E-05 |
| A_23_P204472 | RPLP0 | NM_053275 | -1.65 | 1.50E-05 |
| A_23_P78835 | ZNF787 | NM_001002836 | -1.65 | 6.04E-04 |
| A_33_P3223082 | MRPL19 | NM_014763 | -1.65 | 4.14E-05 |
| A_24_P359545 | RBMXL1 | NM_019610 | -1.65 | 3.60E-04 |
| A_33_P3360525 | BU535024 | BU535024 | -1.65 | 2.51E-04 |
| A_23_P379746 | DENND5B | NM_144973 | -1.65 | 4.42E-04 |
| A_24_P341677 | CR612178 | CR612178 | -1.65 | 9.26E-05 |
| A_33_P3340666 | KRTAP19-5 | NM_181611 | -1.65 | 1.19E-04 |
| A_23_P86632 | DCLRE1C | NM_001033858 | -1.65 | 8.55E-04 |
| A_23_P125829 | PGK1 | NM_000291 | -1.65 | 3.67E-05 |
| A_23_P105664 | CCDC59 | NM_014167 | -1.65 | 2.45E-04 |
| A_33_P3402570 | CCDC59 | NM_014167 | -1.65 | 1.26E-05 |
| A_33_P3355407 | RNU105A | NR_004404 | -1.65 | 3.20E-04 |
| A_23_P144384 | GALNT7 | NM_017423 | -1.65 | 6.49E-05 |
| A_32_P6172 | LOC100128822 | NR_027387 | -1.65 | 4.34E-04 |
| A_24_P325176 | KIAA1109 | NM_015312 | -1.65 | 3.45E-04 |
| A_23_P399501 | PKM2 | NM_182470 | -1.65 | 8.10E-04 |
| A_23_P363896 | C17orf76-AS1 | NR_027162 | -1.65 | 1.17E-04 |
| A_23_P102122 | ARPC2 | NM_152862 | -1.65 | 2.44E-05 |
| A_23_P165840 | ODC1 | NM_002539 | -1.65 | 4.24E-04 |
| A_23_P47208 | BANF1 | NM_003860 | -1.65 | 1.20E-05 |
| A_23_P204980 | UGGT2 | NM_020121 | -1.65 | 2.53E-05 |
| A_21_P0014018 | LOC100652736 | XM_003403487 | -1.65 | 2.71E-04 |
| A_23_P108785 | ACTR3 | NM_005721 | -1.65 | 7.81E-05 |
| A_33_P3328837 | THC2621677 | THC2621677 | -1.65 | 9.71E-06 |
| A_23_P143446 | MRPL39 | NM_017446 | -1.65 | 6.51E-05 |
| A_23_P410587 | PHF17 | NM_024900 | -1.65 | 1.33E-04 |
| A_23_P406350 | MFSD3 | NM_138431 | -1.65 | 3.71E-04 |
| A_23_P162874 | HSP90AA1 | NM_005348 | -1.64 | 8.91E-04 |
| A_23_P11025 | ZNF185 | NM_001178106 | -1.64 | 5.99E-06 |
| A_23_P132438 | MSL2 | NM_018133 | -1.64 | 7.71E-04 |
| A_23_P102122 | ARPC2 | NM_152862 | -1.64 | 1.04E-05 |
| A_23_P102122 | ARPC2 | NM_152862 | -1.64 | 2.76E-04 |
| A_23_P102122 | ARPC2 | NM_152862 | -1.64 | 1.58E-06 |
| A_23_P72840 | DPM3 | NM_018973 | -1.64 | 1.94E-04 |
| A_21_P0006566 | XLOC_008033 | THC2649084 | -1.64 | 6.96E-04 |
| A_21_P0000221 | SNORD32A | NR_000021 | -1.64 | 3.86E-04 |
| A_23_P6802 | RRP9 | NM_004704 | -1.64 | 1.70E-04 |
| A_23_P128663 | SACS | NM_014363 | -1.64 | 2.22E-04 |
| A_23_P123563 | RPS6 | NM_001010 | -1.64 | 1.08E-04 |
| A_23_P162874 | HSP90AA1 | NM_005348 | -1.64 | 9.32E-04 |
| A_33_P3283636 | THC2731370 | THC2731370 | -1.64 | 4.71E-04 |
| A_23_P122304 | HDAC2 | NM_001527 | -1.64 | 4.98E-05 |
| A_23_P102122 | ARPC2 | NM_152862 | -1.64 | 2.67E-05 |
| A_24_P234415 | STAC | NM_003149 | -1.64 | 9.67E-04 |
| A_23_P11025 | ZNF185 | NM_001178106 | -1.64 | 1.61E-05 |
| A_32_P40377 | LOC389906 | NR_034031 | -1.64 | 6.59E-05 |
| A_23_P150238 | C11orf68 | NM_031450 | -1.64 | 5.64E-04 |
| A_24_P123245 | HNRNPD | NM_031370 | -1.64 | 1.43E-04 |
| A_23_P394166 | CNOT7 | NM_013354 | -1.64 | 7.95E-04 |
| A_33_P3354176 | MYOF | ENST00000371488 | -1.64 | 9.00E-04 |
| A_23_P105664 | CCDC59 | NM_014167 | -1.64 | 1.64E-04 |
| A_24_P392690 | NP1202467 | NP1202467 | -1.64 | 4.15E-04 |
| A_24_P136182 | RPS2P32 | NR_026676 | -1.64 | 1.27E-04 |
| A_33_P3780901 | SBNO1 | NM_001167856 | -1.64 | 4.17E-05 |
| A_23_P33407 | HERC2 | NM_004667 | -1.64 | 8.23E-04 |
| A_33_P3230090 | LUZP6 | NM_001128619 | -1.64 | 9.31E-04 |
| A_23_P212639 | TRA2B | NM_004593 | -1.64 | 3.14E-04 |
| A_32_P135902 | EIF4A1 | NM_001416 | -1.64 | 1.33E-04 |
| A_23_P133365 | TCERG1 | NM_006706 | -1.64 | 5.80E-04 |
| A_19_P00320721 | MDM4 | NM_002393 | -1.64 | 1.71E-04 |
| A_23_P108785 | ACTR3 | NM_005721 | -1.64 | 3.65E-04 |
| A_19_P00322533 | CRNDE | NR_034105 | -1.64 | 3.38E-04 |
| A_32_P20367 | RPS7 | NM_001011 | -1.64 | 5.26E-05 |
| A_23_P102122 | ARPC2 | NM_152862 | -1.64 | 3.35E-06 |
| A_23_P162874 | HSP90AA1 | NM_005348 | -1.64 | 3.14E-04 |
| A_23_P215956 | MYC | NM_002467 | -1.64 | 4.79E-05 |
| A_32_P135243 | MTHFD1L | NM_015440 | -1.64 | 6.02E-04 |
| A_23_P118435 | SUMO2 | NM_006937 | -1.64 | 2.04E-05 |
| A_32_P50924 | HNRNPA1L2 | NM_001011724 | -1.64 | 8.04E-04 |
| A_33_P3298492 | PCBP2 | NM_005016 | -1.64 | 1.61E-05 |
| A_23_P162874 | HSP90AA1 | NM_005348 | -1.63 | 9.40E-04 |
| A_33_P3357580 | MRTO4 | NM_016183 | -1.63 | 7.78E-05 |
| A_24_P375849 | THC2555910 | THC2555910 | -1.63 | 1.74E-04 |
| A_24_P861009 | BRWD1 | NM_001007246 | -1.63 | 2.73E-05 |
| A_23_P352266 | BCL2 | NM_000633 | -1.63 | 5.33E-04 |
| A_21_P0000750 | MFI2-AS1 | NR_038285 | -1.63 | 7.60E-04 |
| A_23_P168229 | TXNDC5 | NM_030810 | -1.63 | 3.20E-05 |
| A_23_P120414 | YWHAB | NM_003404 | -1.63 | 5.90E-06 |
| A_23_P145089 | HSP90AB1 | NM_007355 | -1.63 | 2.10E-04 |
| A_23_P108785 | ACTR3 | NM_005721 | -1.63 | 1.50E-04 |
| A_21_P0011341 | NPM1 | NM_002520 | -1.63 | 2.85E-04 |
| A_23_P11025 | ZNF185 | NM_001178106 | -1.63 | 1.03E-05 |
| A_24_P1255 | BCCIP | NM_078469 | -1.63 | 6.09E-04 |
| A_32_P69465 | MORN2 | NM_001145450 | -1.63 | 6.11E-07 |
| A_24_P391431 | TAF9B | NM_015975 | -1.63 | 5.34E-05 |
| A_23_P28625 | WDR12 | NM_018256 | -1.63 | 4.56E-05 |
| A_23_P125829 | PGK1 | NM_000291 | -1.63 | 2.33E-05 |
| A_23_P51317 | CCDC76 | NM_019083 | -1.63 | 2.65E-04 |
| A_24_P154037 | IRS2 | NM_003749 | -1.63 | 2.39E-04 |
| A_23_P136232 | IMPAD1 | NM_017813 | -1.63 | 4.93E-04 |
| A_23_P105664 | CCDC59 | NM_014167 | -1.63 | 7.66E-04 |
| A_32_P104063 | CRNDE | NR_034105 | -1.63 | 8.55E-05 |
| A_33_P3292337 | KIF5B | NM_004521 | -1.63 | 3.28E-04 |
| A_33_P3235766 | QSER1 | NM_001076786 | -1.63 | 1.18E-04 |
| A_24_P472455 | ARF6 | NM_001663 | -1.63 | 7.01E-04 |
| A_21_P0013234 | XLOC_l2_013460 | THC2569255 | -1.63 | 4.63E-04 |
| A_32_P117313 | C8orf83 | NM_001171796 | -1.63 | 6.34E-04 |
| A_23_P108785 | ACTR3 | NM_005721 | -1.63 | 5.98E-04 |
| A_23_P214139 | REV3L | NM_002912 | -1.63 | 2.43E-04 |
| A_24_P135902 | RPS2 | NM_002952 | -1.63 | 1.54E-05 |
| A_24_P161403 | ENST00000407723 | ENST00000407723 | -1.63 | 3.70E-04 |
| A_23_P16762 | PRPF40A | NM_017892 | -1.62 | 4.81E-04 |
| A_24_P75456 | ENST00000445031 | ENST00000445031 | -1.62 | 1.20E-04 |
| A_23_P121095 | TOP2B | NM_001068 | -1.62 | 5.75E-05 |
| A_23_P151337 | DLEU1 | NR_002605 | -1.62 | 1.29E-04 |
| A_33_P3399248 | UFM1 | NM_016617 | -1.62 | 3.67E-05 |
| A_23_P162874 | HSP90AA1 | NM_005348 | -1.62 | 2.35E-04 |
| A_23_P125829 | PGK1 | NM_000291 | -1.62 | 2.36E-04 |
| A_24_P754803 | RPL10 | NM_006013 | -1.62 | 4.59E-05 |
| A_33_P3248519 | SMC4 | NM_005496 | -1.62 | 1.35E-05 |
| A_24_P192434 | TERF1 | NM_017489 | -1.62 | 1.54E-04 |
| A_21_P0013805 | LOC389906 | NR_034031 | -1.62 | 3.85E-05 |
| A_23_P156319 | LARP1 | NM_015315 | -1.62 | 1.48E-05 |
| A_33_P3270384 | PPP1R14B | NM_138689 | -1.62 | 8.52E-05 |
| A_23_P11025 | ZNF185 | NM_001178106 | -1.62 | 4.70E-06 |
| A_23_P125829 | PGK1 | NM_000291 | -1.62 | 3.32E-05 |
| A_32_P222961 | SPIN4 | NM_001012968 | -1.62 | 1.37E-04 |
| A_33_P3396951 | BNIP3L | NM_004331 | -1.62 | 4.09E-04 |
| A_33_P3223208 | CCDC41 | NM_016122 | -1.62 | 2.04E-04 |
| A_23_P122001 | MSH3 | NM_002439 | -1.62 | 3.83E-04 |
| A_23_P204158 | RNFT2 | NM_032814 | -1.62 | 6.34E-05 |
| A_23_P11025 | ZNF185 | NM_001178106 | -1.62 | 3.48E-05 |
| A_23_P99540 | ZFP36L1 | NM_004926 | -1.62 | 3.22E-04 |
| A_23_P104741 | KIRREL3 | NM_032531 | -1.62 | 2.49E-04 |
| A_23_P104025 | TSEN15 | NM_052965 | -1.62 | 6.85E-05 |
| A_23_P120414 | YWHAB | NM_003404 | -1.62 | 6.19E-04 |
| A_23_P34510 | PHC2 | NM_198040 | -1.62 | 8.32E-04 |
| A_23_P121095 | TOP2B | NM_001068 | -1.61 | 4.31E-05 |
| A_24_P330691 | SUMO2 | NM_006937 | -1.61 | 1.09E-04 |
| A_23_P18317 | SLC41A3 | NM_017836 | -1.61 | 1.17E-05 |
| A_23_P162874 | HSP90AA1 | NM_005348 | -1.61 | 8.36E-04 |
| A_23_P162874 | HSP90AA1 | NM_005348 | -1.61 | 7.27E-04 |
| A_23_P11025 | ZNF185 | NM_001178106 | -1.61 | 4.04E-06 |
| A_33_P3257714 | RPS23 | NM_001025 | -1.61 | 1.50E-04 |
| A_23_P215406 | RAC1 | NM_018890 | -1.61 | 1.24E-04 |
| A_33_P3291454 | C10orf96 | NM_198515 | -1.61 | 4.26E-04 |
| A_19_P00812587 | RPL5 | NM_000969 | -1.61 | 3.55E-04 |
| A_24_P341476 | LOC729313 | XR_132614 | -1.61 | 6.41E-04 |
| A_23_P19210 | RPF2 | NM_032194 | -1.61 | 8.29E-04 |
| A_23_P114466 | TBL1Y | NM_033284 | -1.61 | 7.81E-04 |
| A_23_P141520 | C17orf49 | NM_174893 | -1.61 | 7.08E-04 |
| A_23_P122304 | HDAC2 | NM_001527 | -1.61 | 3.10E-05 |
| A_21_P0011675 | XLOC_l2_006609 | TCONS_l2_00012321 | -1.61 | 1.75E-04 |
| A_23_P60248 | TXN | NM_003329 | -1.61 | 5.84E-06 |
| A_24_P316102 | MLLT10 | NM_001195630 | -1.61 | 6.05E-06 |
| A_21_P0000368 | SNORD5 | NR_003033 | -1.61 | 4.14E-04 |
| A_24_P188878 | RPL34 | NM_033625 | -1.61 | 8.27E-05 |
| A_24_P363005 | UBE2D3 | NM_181886 | -1.61 | 2.37E-04 |
| A_23_P120414 | YWHAB | NM_003404 | -1.61 | 1.28E-05 |
| A_24_P702813 | XPR1 | NM_004736 | -1.61 | 6.21E-04 |
| A_33_P3335022 | THC2504295 | THC2504295 | -1.61 | 3.73E-04 |
| A_33_P3392177 | CLIC5 | NM_001114086 | -1.61 | 3.72E-05 |
| A_23_P122674 | PAK1IP1 | NM_017906 | -1.61 | 1.81E-04 |
| A_23_P122001 | MSH3 | NM_002439 | -1.61 | 4.39E-04 |
| A_23_P131227 | TTC27 | NM_017735 | -1.61 | 2.27E-04 |
| A_23_P120414 | YWHAB | NM_003404 | -1.61 | 1.63E-04 |
| A_24_P24972 | ENST00000538228 | ENST00000538228 | -1.61 | 1.06E-04 |
| A_24_P152094 | NP1245239 | NP1245239 | -1.61 | 3.45E-04 |
| A_24_P791829 | NUFIP2 | NM_020772 | -1.61 | 7.08E-04 |
| A_24_P385313 | PTPRF | NM_002840 | -1.61 | 8.68E-06 |
| A_23_P210939 | EIF6 | NM_181468 | -1.61 | 6.54E-04 |
| A_23_P11025 | ZNF185 | NM_001178106 | -1.61 | 5.33E-05 |
| A_23_P122001 | MSH3 | NM_002439 | -1.61 | 1.49E-04 |
| A_23_P162106 | MRPL48 | NM_016055 | -1.61 | 8.79E-05 |
| A_23_P11025 | ZNF185 | NM_001178106 | -1.61 | 5.46E-06 |
| A_33_P3389394 | RPPH1 | NR_002312 | -1.61 | 1.25E-04 |
| A_23_P13663 | FAM60A | NM_021238 | -1.60 | 6.96E-05 |
| A_21_P0012077 | XLOC_l2_008203 | ENST00000432818 | -1.60 | 2.46E-04 |
| A_24_P127312 | THC2554858 | THC2554858 | -1.60 | 1.48E-04 |
| A_23_P111037 | HIST1H3A | NM_003529 | -1.60 | 2.73E-06 |
| A_23_P215956 | MYC | NM_002467 | -1.60 | 8.69E-04 |
| A_23_P215956 | MYC | NM_002467 | -1.60 | 2.37E-04 |
| A_19_P00315528 | XLOC_008370 | AF088007 | -1.60 | 6.52E-05 |
| A_23_P108785 | ACTR3 | NM_005721 | -1.60 | 9.79E-04 |
| A_21_P0007163 | XLOC_009567 | ENST00000457746 | -1.60 | 1.82E-04 |
| A_23_P213661 | PPIP5K2 | NM_015216 | -1.60 | 3.97E-04 |
| A_23_P122674 | PAK1IP1 | NM_017906 | -1.60 | 3.97E-04 |
| A_19_P00805291 | ENST00000441797 | ENST00000441797 | -1.60 | 2.42E-04 |
| A_33_P3882659 | HSP90AB5P | AY956766 | -1.60 | 1.62E-05 |
| A_24_P114249 | GALNT3 | NM_004482 | -1.60 | 2.68E-04 |
| A_19_P00322702 | SNHG5 | NR_003038 | -1.60 | 1.54E-04 |
| A_23_P46844 | TRIM8 | NM_030912 | -1.60 | 6.27E-04 |
| A_24_P388940 | C6orf162 | NM_001042493 | -1.60 | 1.50E-04 |
| A_23_P214091 | LYPLA1 | NM_006330 | -1.60 | 6.76E-06 |
| A_23_P122674 | PAK1IP1 | NM_017906 | -1.60 | 3.83E-04 |
| A_33_P3389188 | TFAM | NM_003201 | -1.60 | 1.43E-04 |
| A_24_P305570 | RIN2 | NM_018993 | -1.60 | 5.85E-04 |
| A_32_P87531 | DNAH14 | NM_001145154 | -1.60 | 6.88E-04 |
| A_23_P122615 | PNISR | NM_032870 | -1.60 | 1.18E-05 |
| A_21_P0003871 | XLOC_003825 | TCONS_00008969 | -1.60 | 7.83E-04 |
| A_23_P12336 | PRMT6 | NM_018137 | -1.60 | 4.94E-04 |
| A_23_P122304 | HDAC2 | NM_001527 | -1.60 | 1.50E-06 |
| A_23_P108785 | ACTR3 | NM_005721 | -1.60 | 1.99E-04 |
| A_23_P121095 | TOP2B | NM_001068 | -1.60 | 8.80E-05 |
| A_23_P110473 | NAIP | NM_004536 | -1.60 | 1.66E-04 |
| A_23_P27381 | TSHZ1 | NM_005786 | -1.60 | 5.62E-04 |
| A_23_P108785 | ACTR3 | NM_005721 | -1.60 | 2.18E-04 |
| A_33_P3421365 | ZNF169 | NM_194320 | -1.60 | 4.60E-04 |
| A_24_P38895 | H2AFX | NM_002105 | -1.60 | 1.78E-04 |
| A_23_P110473 | NAIP | NM_004536 | -1.60 | 8.22E-04 |
| A_21_P0001879 | MOB1A | AK123865 | -1.60 | 3.96E-04 |
| A_32_P123629 | TTC39C | NM_001135993 | -1.60 | 4.19E-04 |
| A_23_P211007 | NRIP1 | NM_003489 | -1.60 | 3.77E-04 |
| A_23_P11025 | ZNF185 | NM_001178106 | -1.60 | 3.73E-05 |
| A_32_P220696 | TERF1 | NM_017489 | -1.60 | 1.25E-04 |
| A_23_P74269 | SRM | NM_003132 | -1.60 | 2.61E-04 |
| A_23_P258340 | PPIA | NM_021130 | -1.60 | 8.27E-05 |
| A_33_P3335124 | RPS2 | NM_002952 | -1.60 | 3.61E-06 |
| A_23_P12336 | PRMT6 | NM_018137 | -1.60 | 5.71E-04 |
| A_23_P11025 | ZNF185 | NM_001178106 | -1.60 | 1.90E-05 |
| A_23_P121095 | TOP2B | NM_001068 | -1.60 | 1.79E-04 |
| A_23_P389919 | WHSC1 | NM_133330 | -1.60 | 6.32E-04 |
| A_19_P00808320 | BC014023 | BC014023 | -1.60 | 1.72E-04 |
| A_33_P3716128 | SMC4 | NM_005496 | -1.60 | 1.27E-05 |
| A_23_P120414 | YWHAB | NM_003404 | -1.60 | 2.63E-04 |
| A_23_P155147 | ZBED4 | NM_014838 | -1.59 | 8.08E-04 |
| A_23_P104741 | KIRREL3 | NM_032531 | -1.59 | 4.19E-04 |
| A_21_P0005250 | LOC100506507 | XR_108853 | -1.59 | 2.18E-04 |
| A_21_P0007030 | XLOC_008984 | TCONS_00018636 | -1.59 | 1.37E-04 |
| A_24_P74932 | PLP2 | NM_002668 | -1.59 | 2.70E-04 |
| A_24_P287756 | NUDT21 | NM_007006 | -1.59 | 4.01E-04 |
| A_23_P104025 | TSEN15 | NM_052965 | -1.59 | 4.51E-04 |
| A_24_P144499 | PPIAL4A | NM_178230 | -1.59 | 5.38E-04 |
| A_33_P3268567 | NCK2 | NM_003581 | -1.59 | 1.67E-04 |
| A_21_P0011271 | RPL22 | NM_000983 | -1.59 | 1.68E-05 |
| A_23_P58293 | UBE2D3 | NM_181886 | -1.59 | 3.49E-05 |
| A_23_P157196 | RPS2P32 | NR_026676 | -1.59 | 9.61E-06 |
| A_23_P58280 | GAR1 | NM_018983 | -1.59 | 4.65E-04 |
| A_23_P121095 | TOP2B | NM_001068 | -1.59 | 6.91E-05 |
| A_33_P3359306 | MTA1 | NM_004689 | -1.59 | 6.81E-05 |
| A_24_P138022 | FAM120A | NM_014612 | -1.59 | 1.58E-04 |
| A_23_P30024 | NFKB1 | NM_003998 | -1.59 | 1.89E-04 |
| A_23_P215956 | MYC | NM_002467 | -1.59 | 1.98E-04 |
| A_33_P3369286 | IPO8 | NM_006390 | -1.59 | 8.99E-04 |
| A_32_P8813 | LOC283663 | NR_024433 | -1.59 | 7.69E-04 |
| A_19_P00318645 | CRNDE | NR_034105 | -1.59 | 5.81E-05 |
| A_33_P3327479 | ZDHHC3 | NM_016598 | -1.59 | 3.96E-04 |
| A_21_P0000215 | SNORD55 | NR_000015 | -1.59 | 3.60E-05 |
| A_23_P215406 | RAC1 | NM_018890 | -1.59 | 1.32E-04 |
| A_24_P195794 | AK130930 | AK130930 | -1.59 | 9.64E-06 |
| A_19_P00322948 | SNHG6 | NR_002599 | -1.59 | 4.40E-04 |
| A_23_P106145 | ERO1L | NM_014584 | -1.59 | 6.02E-05 |
| A_23_P125829 | PGK1 | NM_000291 | -1.59 | 6.97E-05 |
| A_32_P184279 | CCDC6 | NM_005436 | -1.59 | 9.15E-05 |
| A_23_P120170 | TIGD1 | NM_145702 | -1.59 | 4.21E-04 |
| A_21_P0011945 | XLOC_l2_008195 | TCONS_l2_00014773 | -1.59 | 1.53E-04 |
| A_23_P258340 | PPIA | NM_021130 | -1.59 | 9.64E-05 |
| A_23_P40025 | DAZAP2 | NM_014764 | -1.59 | 5.28E-05 |
| A_24_P312692 | API5 | NM_006595 | -1.59 | 2.94E-04 |
| A_23_P253177 | STK24 | NM_001032296 | -1.59 | 6.05E-04 |
| A_23_P2066 | APIP | NM_015957 | -1.59 | 9.94E-05 |
| A_21_P0011150 | DHX9 | NM_001357 | -1.59 | 4.36E-04 |
| A_23_P250813 | WRN | NM_000553 | -1.59 | 8.79E-04 |
| A_23_P111381 | ATG5 | NM_004849 | -1.59 | 2.44E-04 |
| A_23_P30024 | NFKB1 | NM_003998 | -1.59 | 1.10E-05 |
| A_23_P122304 | HDAC2 | NM_001527 | -1.59 | 1.69E-04 |
| A_23_P115645 | CELF2 | NM_001025076 | -1.59 | 3.30E-04 |
| A_23_P256773 | TSSC1 | NM_003310 | -1.59 | 8.32E-05 |
| A_23_P122001 | MSH3 | NM_002439 | -1.59 | 3.32E-04 |
| A_23_P111381 | ATG5 | NM_004849 | -1.58 | 3.99E-05 |
| A_23_P109593 | TBC1D22A | NM_014346 | -1.58 | 2.20E-05 |
| A_23_P120414 | YWHAB | NM_003404 | -1.58 | 1.31E-04 |
| A_23_P125829 | PGK1 | NM_000291 | -1.58 | 3.16E-05 |
| A_33_P3360426 | WDR1 | NM_017491 | -1.58 | 5.16E-04 |
| A_23_P168898 | RPS20 | NM_001023 | -1.58 | 4.51E-05 |
| A_23_P121095 | TOP2B | NM_001068 | -1.58 | 4.15E-06 |
| A_21_P0013784 | XLOC_l2_015585 | BC078675 | -1.58 | 4.14E-05 |
| A_23_P120414 | YWHAB | NM_003404 | -1.58 | 4.36E-05 |
| A_23_P340333 | ITPRIP | NM_033397 | -1.58 | 1.31E-04 |
| A_32_P93852 | BOD1 | NM_138369 | -1.58 | 7.78E-04 |
| A_23_P104025 | TSEN15 | NM_052965 | -1.58 | 5.16E-05 |
| A_23_P215956 | MYC | NM_002467 | -1.58 | 1.53E-05 |
| A_23_P215406 | RAC1 | NM_018890 | -1.58 | 7.02E-05 |
| A_33_P3423610 | Sep15 | NM_004261 | -1.58 | 2.74E-04 |
| A_33_P3420762 | THC2577776 | THC2577776 | -1.58 | 2.71E-04 |
| A_33_P3321533 | THC2671740 | THC2671740 | -1.58 | 6.81E-04 |
| A_23_P120414 | YWHAB | NM_003404 | -1.58 | 5.47E-05 |
| A_23_P120414 | YWHAB | NM_003404 | -1.58 | 3.69E-06 |
| A_32_P170444 | SUB1 | NM_006713 | -1.58 | 6.74E-04 |
| A_23_P401098 | TTC39C | NM_153211 | -1.58 | 7.93E-04 |
| A_23_P53668 | NFYB | NM_006166 | -1.58 | 2.40E-04 |
| A_23_P54576 | KIFC3 | NM_005550 | -1.58 | 1.24E-04 |
| A_23_P115645 | CELF2 | NM_001025076 | -1.58 | 5.61E-04 |
| A_24_P95154 | TUSC3 | NM_178234 | -1.58 | 9.54E-04 |
| A_23_P55477 | ADORA2B | NM_000676 | -1.58 | 6.09E-05 |
| A_23_P29769 | WWTR1 | NM_015472 | -1.58 | 8.11E-04 |
| A_23_P215406 | RAC1 | NM_018890 | -1.58 | 3.68E-06 |
| A_23_P146187 | RRS1 | NM_015169 | -1.58 | 9.37E-05 |
| A_33_P3414669 | RLIM | NM_183353 | -1.58 | 5.91E-04 |
| A_21_P0000841 | LOC100505483 | NR_038926 | -1.58 | 4.89E-04 |
| A_24_P309360 | TM9SF3 | NM_020123 | -1.58 | 3.34E-06 |
| A_33_P3789894 | SNORA75 | AW382724 | -1.58 | 1.43E-04 |
| A_23_P102420 | CCT4 | NM_006430 | -1.58 | 9.66E-05 |
| A_24_P136211 | CR590938 | CR590938 | -1.58 | 6.44E-05 |
| A_32_P148710 | CFL1 | NM_005507 | -1.57 | 2.26E-04 |
| A_23_P215956 | MYC | NM_002467 | -1.57 | 2.13E-04 |
| A_23_P161644 | RBM14 | NM_006328 | -1.57 | 5.29E-04 |
| A_23_P122001 | MSH3 | NM_002439 | -1.57 | 2.49E-04 |
| A_23_P201002 | RNF220 | NM_018150 | -1.57 | 2.53E-04 |
| A_23_P104741 | KIRREL3 | NM_032531 | -1.57 | 7.24E-04 |
| A_23_P122304 | HDAC2 | NM_001527 | -1.57 | 3.58E-05 |
| A_24_P140608 | HBEGF | NM_001945 | -1.57 | 9.33E-05 |
| A_23_P23765 | ITGB3BP | NM_014288 | -1.57 | 1.27E-05 |
| A_33_P3269598 | C9orf102 | ENST00000479391 | -1.57 | 3.56E-04 |
| A_33_P3407945 | TMEM19 | NM_018279 | -1.57 | 4.68E-04 |
| A_32_P38467 | SNHG8 | NR_003584 | -1.57 | 1.08E-04 |
| A_21_P0000233 | SNORD38A | NR_001456 | -1.57 | 9.02E-04 |
| A_33_P3384997 | ENST00000402278 | ENST00000402278 | -1.57 | 1.84E-04 |
| A_24_P266728 | SF1 | NM_004630 | -1.57 | 9.81E-04 |
| A_23_P108785 | ACTR3 | NM_005721 | -1.57 | 5.71E-04 |
| A_23_P104025 | TSEN15 | NM_052965 | -1.57 | 4.59E-04 |
| A_24_P242820 | TSN | NM_004622 | -1.57 | 3.51E-04 |
| A_23_P406135 | IFT172 | NM_015662 | -1.57 | 4.94E-04 |
| A_23_P122674 | PAK1IP1 | NM_017906 | -1.57 | 2.68E-04 |
| A_23_P86855 | MACROD1 | NM_014067 | -1.57 | 9.45E-04 |
| A_24_P179013 | NP1083521 | NP1083521 | -1.57 | 6.09E-05 |
| A_23_P167401 | PCDHB11 | NM_018931 | -1.57 | 2.01E-04 |
| A_23_P41267 | LOC401127 | NR_026854 | -1.57 | 1.49E-04 |
| A_24_P710730 | LOC100170939 | NR_024054 | -1.57 | 1.66E-04 |
| A_33_P3278410 | MLLT4 | NM_001207008 | -1.57 | 3.02E-04 |
| A_32_P187599 | SERBP1 | NM_001018067 | -1.57 | 8.86E-05 |
| A_23_P111381 | ATG5 | NM_004849 | -1.57 | 7.30E-05 |
| A_23_P123330 | RPL30 | NM_000989 | -1.57 | 5.52E-05 |
| A_33_P3702364 | SNX24 | NM_014035 | -1.57 | 4.46E-04 |
| A_23_P20683 | KIAA0020 | NM_014878 | -1.57 | 9.87E-04 |
| A_23_P137909 | HIST3H3 | NM_003493 | -1.57 | 5.88E-06 |
| A_24_P3461 | ENST00000434007 | ENST00000434007 | -1.57 | 2.28E-04 |
| A_21_P0010889 | LOC728190 | NR_024397 | -1.57 | 2.66E-04 |
| A_23_P215406 | RAC1 | NM_018890 | -1.57 | 8.39E-05 |
| A_33_P3361513 | NLE1 | NM_001014445 | -1.57 | 3.53E-06 |
| A_23_P98085 | PTEN | NM_000314 | -1.57 | 6.66E-04 |
| A_23_P215406 | RAC1 | NM_018890 | -1.57 | 7.19E-06 |
| A_23_P122674 | PAK1IP1 | NM_017906 | -1.57 | 9.50E-04 |
| A_23_P370434 | C1QBP | NM_001212 | -1.57 | 4.64E-04 |
| A_23_P54041 | THTPA | NM_024328 | -1.57 | 9.13E-04 |
| A_23_P83463 | Sep15 | NM_004261 | -1.57 | 8.46E-05 |
| A_33_P3235454 | THC2542270 | THC2542270 | -1.57 | 7.29E-04 |
| A_23_P8763 | PTPN12 | NM_002835 | -1.57 | 7.08E-05 |
| A_23_P258340 | PPIA | NM_021130 | -1.57 | 2.49E-04 |
| A_23_P75330 | HNRNPF | NM_004966 | -1.57 | 2.80E-04 |
| A_23_P39542 | C2orf76 | NM_001017927 | -1.57 | 8.19E-04 |
| A_23_P125829 | PGK1 | NM_000291 | -1.57 | 5.15E-06 |
| A_33_P3333975 | CBWD5 | NM_001024916 | -1.57 | 1.78E-05 |
| A_33_P3319765 | FANCC | NM_001243744 | -1.57 | 6.38E-04 |
| A_21_P0000598 | FBXW4 | NM_022039 | -1.57 | 5.60E-04 |
| A_33_P3216237 | BZW2 | NM_001159767 | -1.57 | 3.84E-04 |
| A_21_P0011990 | LOC646324 | NR_037195 | -1.56 | 7.00E-05 |
| A_24_P17302 | UBE2J2 | NM_194458 | -1.56 | 9.62E-05 |
| A_23_P22352 | FRMD4A | ENST00000342409 | -1.56 | 7.39E-04 |
| A_33_P3251538 | MAPKAP1 | NM_001006618 | -1.56 | 7.66E-05 |
| A_21_P0014815 | LOC646778 | XR_132648 | -1.56 | 1.97E-04 |
| A_23_P45917 | CKS1B | NM_001826 | -1.56 | 3.05E-05 |
| A_24_P140391 | PIGY | NM_001042616 | -1.56 | 3.30E-04 |
| A_23_P12336 | PRMT6 | NM_018137 | -1.56 | 7.30E-04 |
| A_23_P163467 | C15orf52 | NM_207380 | -1.56 | 4.18E-04 |
| A_23_P121095 | TOP2B | NM_001068 | -1.56 | 3.42E-05 |
| A_23_P328206 | DNMBP | NM_015221 | -1.56 | 8.04E-05 |
| A_23_P158925 | GPR125 | NM_145290 | -1.56 | 4.81E-04 |
| A_33_P3406245 | TAF1A | NM_005681 | -1.56 | 8.39E-04 |
| A_23_P104741 | KIRREL3 | NM_032531 | -1.56 | 6.34E-04 |
| A_32_P174572 | HTR7P1 | NR_002774 | -1.56 | 2.19E-04 |
| A_19_P00316659 | SNHG5 | NR_003038 | -1.56 | 3.63E-04 |
| A_32_P54544 | CCT6A | NM_001762 | -1.56 | 9.84E-05 |
| A_23_P111381 | ATG5 | NM_004849 | -1.56 | 4.27E-05 |
| A_23_P122001 | MSH3 | NM_002439 | -1.56 | 3.30E-04 |
| A_23_P104876 | SPA17 | NM_017425 | -1.56 | 6.50E-05 |
| A_23_P7827 | FAM26F | NM_001010919 | -1.56 | 5.52E-04 |
| A_23_P122001 | MSH3 | NM_002439 | -1.56 | 4.45E-04 |
| A_33_P3419334 | GNAI3 | NM_006496 | -1.56 | 5.69E-06 |
| A_23_P98085 | PTEN | NM_000314 | -1.56 | 3.73E-04 |
| A_24_P340679 | PPIA | NM_021130 | -1.56 | 1.94E-04 |
| A_33_P3397755 | MAPKAP1 | NM_001006618 | -1.56 | 6.14E-04 |
| A_23_P105571 | CHPT1 | NM_020244 | -1.56 | 7.08E-04 |
| A_23_P259272 | WSB2 | NM_018639 | -1.56 | 4.60E-04 |
| A_23_P122304 | HDAC2 | NM_001527 | -1.56 | 2.17E-04 |
| A_23_P88740 | CENPN | NM_018455 | -1.56 | 7.05E-04 |
| A_24_P203226 | PPIAL4A | NM_178230 | -1.56 | 5.91E-04 |
| A_23_P34496 | TMEM39B | NM_018056 | -1.56 | 2.03E-06 |
| A_23_P128734 | ERH | NM_004450 | -1.56 | 1.15E-04 |
| A_23_P108785 | ACTR3 | NM_005721 | -1.56 | 4.22E-04 |
| A_23_P120414 | YWHAB | NM_003404 | -1.56 | 8.62E-05 |
| A_23_P30024 | NFKB1 | NM_003998 | -1.56 | 3.46E-05 |
| A_23_P316741 | TSPAN4 | NM_001025237 | -1.56 | 2.19E-05 |
| A_23_P305245 | C3orf80 | NM_001168214 | -1.56 | 2.21E-04 |
| A_23_P104892 | EIF4G2 | NM_001418 | -1.56 | 2.86E-04 |
| A_23_P98252 | ARL2 | NM_001667 | -1.56 | 6.85E-04 |
| A_21_P0013300 | XLOC_l2_013808 | TCONS_l2_00026546 | -1.56 | 6.95E-04 |
| A_23_P215956 | MYC | NM_002467 | -1.56 | 7.35E-04 |
| A_23_P23765 | ITGB3BP | NM_014288 | -1.56 | 2.87E-04 |
| A_23_P12336 | PRMT6 | NM_018137 | -1.56 | 8.99E-04 |
| A_23_P161918 | CCDC86 | NM_024098 | -1.56 | 1.40E-04 |
| A_33_P3374833 | PLDN | NM_012388 | -1.56 | 4.95E-04 |
| A_24_P29445 | TMEM14B | NM_030969 | -1.56 | 9.62E-05 |
| A_21_P0013757 | XLOC_l2_015478 | TCONS_l2_00030171 | -1.56 | 4.64E-04 |
| A_23_P346384 | MRPL43 | NM_176792 | -1.56 | 8.56E-05 |
| A_21_P0000190 | AFF3 | NM_002285 | -1.56 | 3.97E-04 |
| A_21_P0006274 | XLOC_007725 | TCONS_00016344 | -1.56 | 7.62E-04 |
| A_23_P104025 | TSEN15 | NM_052965 | -1.56 | 7.47E-04 |
| A_21_P0013170 | XLOC_l2_013272 | BF348138 | -1.56 | 4.41E-04 |
| A_21_P0014623 | LOC100505616 | XR_110597 | -1.56 | 6.84E-04 |
| A_33_P3314902 | BIRC6 | NM_016252 | -1.56 | 3.56E-04 |
| A_32_P143880 | MEMO1 | NM_015955 | -1.56 | 2.97E-04 |
| A_33_P3234410 | THC2587207 | THC2587207 | -1.55 | 1.37E-04 |
| A_23_P122001 | MSH3 | NM_002439 | -1.55 | 7.84E-04 |
| A_23_P102420 | CCT4 | NM_006430 | -1.55 | 4.94E-04 |
| A_23_P98085 | PTEN | NM_000314 | -1.55 | 7.88E-05 |
| A_33_P3412538 | ANKRD17 | NM_032217 | -1.55 | 4.81E-04 |
| A_23_P23765 | ITGB3BP | NM_014288 | -1.55 | 7.33E-05 |
| A_23_P134925 | BNIP3L | NM_004331 | -1.55 | 6.72E-05 |
| A_23_P98085 | PTEN | NM_000314 | -1.55 | 8.18E-04 |
| A_23_P202458 | ZNF22 | NM_006963 | -1.55 | 4.98E-04 |
| A_19_P00319019 | SNHG5 | NR_003038 | -1.55 | 8.52E-04 |
| A_23_P73721 | RRAGB | NM_016656 | -1.55 | 7.60E-04 |
| A_23_P106145 | ERO1L | NM_014584 | -1.55 | 9.42E-05 |
| A_23_P104025 | TSEN15 | NM_052965 | -1.55 | 1.12E-04 |
| A_32_P201773 | AMMECR1 | NM_015365 | -1.55 | 9.38E-04 |
| A_23_P15202 | DHODH | NM_001361 | -1.55 | 4.79E-04 |
| A_33_P3413216 | TSPAN4 | NM_001025237 | -1.55 | 8.27E-04 |
| A_23_P422851 | CABLES1 | NM_138375 | -1.55 | 3.38E-04 |
| A_33_P3324890 | HSP90AB2P | NR_003132 | -1.55 | 4.37E-04 |
| A_32_P72940 | RPL35 | NM_007209 | -1.55 | 3.76E-04 |
| A_23_P121095 | TOP2B | NM_001068 | -1.55 | 3.19E-05 |
| A_33_P3294252 | ATF1 | NM_005171 | -1.55 | 1.14E-04 |
| A_23_P43141 | EIF3E | NM_001568 | -1.55 | 2.97E-04 |
| A_23_P61398 | PIM3 | NM_001001852 | -1.55 | 6.10E-05 |
| A_23_P69791 | AP1AR | NM_018569 | -1.55 | 3.43E-05 |
| A_23_P23765 | ITGB3BP | NM_014288 | -1.55 | 2.34E-06 |
| A_23_P7221 | RPL34 | NM_033625 | -1.55 | 3.26E-04 |
| A_19_P00810361 | AF239727 | AF239727 | -1.55 | 5.05E-05 |
| A_23_P23765 | ITGB3BP | NM_014288 | -1.55 | 4.31E-04 |
| A_24_P36847 | DHX9 | NM_001357 | -1.55 | 1.68E-04 |
| A_24_P81841 | CDKN1B | NM_004064 | -1.55 | 2.34E-04 |
| A_23_P104892 | EIF4G2 | NM_001418 | -1.55 | 4.50E-04 |
| A_21_P0012845 | MRPL10 | NM_148887 | -1.55 | 1.48E-04 |
| A_32_P149536 | SUMO2 | NM_006937 | -1.55 | 1.28E-05 |
| A_23_P122304 | HDAC2 | NM_001527 | -1.55 | 3.98E-04 |
| A_23_P12336 | PRMT6 | NM_018137 | -1.55 | 5.62E-04 |
| A_23_P258340 | PPIA | NM_021130 | -1.55 | 1.55E-04 |
| A_23_P41734 | RNF130 | NM_018434 | -1.55 | 3.97E-04 |
| A_24_P30206 | BCCIP | NM_078468 | -1.55 | 4.18E-05 |
| A_24_P33444 | YWHAE | NM_006761 | -1.55 | 4.45E-04 |
| A_23_P155332 | PCNP | NM_020357 | -1.55 | 6.41E-06 |
| A_23_P258340 | PPIA | NM_021130 | -1.55 | 8.28E-05 |
| A_23_P30024 | NFKB1 | NM_003998 | -1.55 | 3.04E-05 |
| A_23_P141315 | NLE1 | NM_001014445 | -1.55 | 7.43E-04 |
| A_23_P122674 | PAK1IP1 | NM_017906 | -1.55 | 9.01E-04 |
| A_23_P122674 | PAK1IP1 | NM_017906 | -1.54 | 8.38E-04 |
| A_24_P724040 | SNRPB2 | NM_003092 | -1.54 | 2.26E-04 |
| A_23_P369899 | TMEM158 | NM_015444 | -1.54 | 6.86E-04 |
| A_23_P102508 | SLC5A6 | NM_021095 | -1.54 | 1.32E-05 |
| A_23_P104892 | EIF4G2 | NM_001418 | -1.54 | 9.65E-04 |
| A_23_P214977 | SEC63 | NM_007214 | -1.54 | 9.22E-04 |
| A_23_P111381 | ATG5 | NM_004849 | -1.54 | 2.18E-04 |
| A_24_P381555 | SAP18 | NM_005870 | -1.54 | 3.27E-05 |
| A_23_P258340 | PPIA | NM_021130 | -1.54 | 1.32E-04 |
| A_32_P110243 | RPS20P27 | BC071734 | -1.54 | 2.18E-04 |
| A_23_P388433 | C4orf3 | NM_001170330 | -1.54 | 7.94E-04 |
| A_23_P213661 | PPIP5K2 | NM_015216 | -1.54 | 5.61E-04 |
| A_23_P122304 | HDAC2 | NM_001527 | -1.54 | 6.23E-04 |
| A_23_P111228 | COQ3 | NM_017421 | -1.54 | 6.04E-04 |
| A_21_P0013060 | XLOC_l2_012870 | THC2774178 | -1.54 | 7.49E-05 |
| A_33_P3316505 | SNORA73A | NR_002907 | -1.54 | 2.07E-04 |
| A_23_P71530 | TNFRSF11B | NM_002546 | -1.54 | 9.89E-04 |
| A_23_P258340 | PPIA | NM_021130 | -1.54 | 9.36E-05 |
| A_23_P258340 | PPIA | NM_021130 | -1.54 | 1.74E-04 |
| A_23_P343411 | AGRN | NM_198576 | -1.54 | 9.92E-04 |
| A_23_P121095 | TOP2B | NM_001068 | -1.54 | 5.49E-05 |
| A_23_P129659 | ZNF689 | NM_138447 | -1.54 | 4.49E-04 |
| A_23_P144816 | VDAC1 | NM_003374 | -1.54 | 1.20E-06 |
| A_23_P217666 | RPL10 | NM_006013 | -1.54 | 3.04E-05 |
| A_19_P00315717 | FAM200B | NM_001145191 | -1.54 | 8.94E-04 |
| A_33_P3268144 | ZNF614 | NM_025040 | -1.54 | 3.36E-04 |
| A_23_P40307 | SNRPB2 | NM_003092 | -1.54 | 7.41E-05 |
| A_33_P3340404 | SCLT1 | NM_144643 | -1.54 | 9.74E-05 |
| A_23_P365060 | MDN1 | NM_014611 | -1.54 | 5.51E-04 |
| A_23_P25525 | GTF3A | NM_002097 | -1.54 | 1.09E-04 |
| A_33_P3240229 | CREBBP | NM_004380 | -1.54 | 9.93E-04 |
| A_23_P63829 | HSPA14 | NM_016299 | -1.54 | 2.36E-05 |
| A_24_P95038 | PPIA | NM_021130 | -1.54 | 8.00E-04 |
| A_23_P45726 | HNRNPR | NM_005826 | -1.54 | 3.49E-04 |
| A_23_P18292 | RPL14 | NM_001034996 | -1.54 | 2.04E-04 |
| A_23_P344451 | HDGFRP3 | NM_016073 | -1.53 | 3.42E-05 |
| A_23_P361085 | SNHG5 | NR_003038 | -1.53 | 7.06E-04 |
| A_23_P117068 | SNRPF | NM_003095 | -1.53 | 2.46E-04 |
| A_23_P120170 | TIGD1 | NM_145702 | -1.53 | 6.09E-04 |
| A_24_P57528 | SLC39A11 | NM_139177 | -1.53 | 9.43E-04 |
| A_23_P18422 | MRPL3 | NM_007208 | -1.53 | 6.76E-04 |
| A_23_P106145 | ERO1L | NM_014584 | -1.53 | 6.03E-05 |
| A_23_P102420 | CCT4 | NM_006430 | -1.53 | 2.56E-04 |
| A_23_P106145 | ERO1L | NM_014584 | -1.53 | 4.78E-05 |
| A_32_P129527 | C6orf70 | NM_018341 | -1.53 | 1.64E-04 |
| A_23_P102420 | CCT4 | NM_006430 | -1.53 | 1.58E-04 |
| A_33_P3218089 | CLDN24 | NM_001185149 | -1.53 | 6.70E-04 |
| A_23_P30024 | NFKB1 | NM_003998 | -1.53 | 2.11E-05 |
| A_23_P7684 | CCNJL | NM_024565 | -1.53 | 6.57E-04 |
| A_19_P00812215 | MRPL39 | NM_017446 | -1.53 | 1.06E-04 |
| A_23_P206532 | PHKB | NM_001031835 | -1.53 | 1.75E-04 |
| A_33_P3390335 | RSU1 | NM_012425 | -1.53 | 4.90E-04 |
| A_23_P122304 | HDAC2 | NM_001527 | -1.53 | 9.86E-05 |
| A_23_P215406 | RAC1 | NM_018890 | -1.53 | 5.16E-05 |
| A_23_P127140 | RAB11FIP2 | NM_014904 | -1.53 | 2.95E-04 |
| A_23_P108785 | ACTR3 | NM_005721 | -1.53 | 4.60E-04 |
| A_23_P67785 | SPAG16 | NM_001025436 | -1.53 | 1.75E-04 |
| A_32_P60223 | ING5 | NM_032329 | -1.53 | 6.31E-04 |
| A_33_P3325229 | LOC389906 | NR_034031 | -1.53 | 3.81E-04 |
| A_23_P28652 | C2orf28 | NM_016085 | -1.53 | 1.01E-04 |
| A_23_P215406 | RAC1 | NM_018890 | -1.53 | 5.30E-06 |
| A_23_P501887 | DHPS | NM_013406 | -1.53 | 1.97E-04 |
| A_33_P3276282 | PTPRF | ENST00000436724 | -1.53 | 1.64E-04 |
| A_23_P436353 | MLLT4 | NM_001207008 | -1.53 | 7.07E-04 |
| A_24_P263937 | CCDC23 | NM_199342 | -1.53 | 1.25E-04 |
| A_24_P409330 | MRPL52 | NM_181304 | -1.53 | 3.71E-04 |
| A_33_P3357322 | SMC2 | NM_001042550 | -1.53 | 1.49E-04 |
| A_23_P73780 | IRAK1 | NM_001569 | -1.53 | 9.57E-05 |
| A_23_P111188 | ZBTB22 | NM_005453 | -1.53 | 3.47E-04 |
| A_32_P100258 | FLJ37453 | NR_024279 | -1.53 | 4.51E-05 |
| A_23_P117068 | SNRPF | NM_003095 | -1.53 | 2.05E-06 |
| A_24_P303989 | BMI1 | NM_005180 | -1.53 | 5.05E-04 |
| A_23_P92552 | PET112 | NM_004564 | -1.53 | 1.50E-05 |
| A_23_P106145 | ERO1L | NM_014584 | -1.53 | 4.01E-04 |
| A_33_P3320943 | THC2525667 | THC2525667 | -1.53 | 2.42E-04 |
| A_33_P3268564 | NCK2 | NM_001004722 | -1.53 | 5.62E-04 |
| A_24_P409857 | HMGN1 | NM_004965 | -1.53 | 1.25E-05 |
| A_23_P115645 | CELF2 | NM_001025076 | -1.53 | 7.18E-04 |
| A_23_P102420 | CCT4 | NM_006430 | -1.53 | 1.52E-04 |
| A_23_P104025 | TSEN15 | NM_052965 | -1.53 | 2.69E-04 |
| A_23_P104876 | SPA17 | NM_017425 | -1.53 | 2.04E-05 |
| A_24_P269895 | HNRNPA3 | NM_194247 | -1.53 | 9.51E-04 |
| A_23_P103690 | FAM189B | NM_006589 | -1.53 | 8.47E-04 |
| A_32_P215113 | CCDC58 | NM_001017928 | -1.53 | 8.95E-04 |
| A_21_P0000489 | SNORD104 | NR_004380 | -1.52 | 6.57E-04 |
| A_23_P122304 | HDAC2 | NM_001527 | -1.52 | 2.47E-04 |
| A_33_P3218694 | PNN | NM_002687 | -1.52 | 3.85E-04 |
| A_23_P40847 | CHST2 | NM_004267 | -1.52 | 5.21E-04 |
| A_23_P102508 | SLC5A6 | NM_021095 | -1.52 | 1.07E-04 |
| A_23_P123330 | RPL30 | NM_000989 | -1.52 | 8.76E-06 |
| A_23_P122615 | PNISR | NM_032870 | -1.52 | 6.34E-04 |
| A_33_P3366987 | GPR125 | NM_145290 | -1.52 | 3.75E-04 |
| A_23_P47614 | PHLDA2 | NM_003311 | -1.52 | 2.14E-05 |
| A_23_P104892 | EIF4G2 | NM_001418 | -1.52 | 4.67E-04 |
| A_21_P0010912 | XLOC_l2_002176 | TCONS_l2_00003853 | -1.52 | 3.06E-04 |
| A_23_P58466 | SMN1 | NM_000344 | -1.52 | 1.17E-04 |
| A_23_P104892 | EIF4G2 | NM_001418 | -1.52 | 9.31E-04 |
| A_23_P70201 | CHD1 | NM_001270 | -1.52 | 8.42E-04 |
| A_23_P213661 | PPIP5K2 | NM_015216 | -1.52 | 4.90E-04 |
| A_23_P111381 | ATG5 | NM_004849 | -1.52 | 4.05E-05 |
| A_23_P30024 | NFKB1 | NM_003998 | -1.52 | 4.01E-04 |
| A_24_P408424 | MYH9 | NM_002473 | -1.52 | 9.18E-06 |
| A_24_P335358 | PUS1 | NM_025215 | -1.52 | 4.05E-04 |
| A_33_P3324651 | RBM39 | NM_001242600 | -1.52 | 7.17E-05 |
| A_23_P148513 | GNG5 | NM_005274 | -1.52 | 1.61E-04 |
| A_23_P111381 | ATG5 | NM_004849 | -1.52 | 6.94E-04 |
| A_23_P104876 | SPA17 | NM_017425 | -1.52 | 4.18E-05 |
| A_23_P104201 | YME1L1 | NM_139312 | -1.52 | 3.14E-04 |
| A_23_P404965 | GNL1 | NM_005275 | -1.52 | 9.18E-04 |
| A_19_P00322944 | SNHG5 | NR_003038 | -1.52 | 7.19E-04 |
| A_23_P204252 | M6PR | NM_002355 | -1.52 | 5.06E-04 |
| A_23_P104892 | EIF4G2 | NM_001418 | -1.52 | 6.63E-04 |
| A_23_P123330 | RPL30 | NM_000989 | -1.52 | 6.59E-05 |
| A_23_P117068 | SNRPF | NM_003095 | -1.52 | 1.44E-04 |
| A_21_P0012564 | XLOC_l2_010508 | ENST00000445739 | -1.52 | 6.41E-06 |
| A_33_P3258127 | LOC100132188 | AY203946 | -1.52 | 7.66E-04 |
| A_21_P0013728 | FRG1 | NM_004477 | -1.52 | 9.50E-05 |
| A_23_P213661 | PPIP5K2 | NM_015216 | -1.52 | 7.16E-04 |
| A_23_P117068 | SNRPF | NM_003095 | -1.52 | 1.14E-04 |
| A_23_P12336 | PRMT6 | NM_018137 | -1.52 | 5.29E-04 |
| A_33_P3392952 | FOXK2 | NM_004514 | -1.52 | 7.12E-04 |
| A_23_P104892 | EIF4G2 | NM_001418 | -1.52 | 7.94E-04 |
| A_24_P72750 | SPIN1 | NM_006717 | -1.52 | 3.28E-04 |
| A_23_P213661 | PPIP5K2 | NM_015216 | -1.52 | 2.89E-04 |
| A_33_P3231432 | LDHB | NM_001174097 | -1.52 | 6.37E-04 |
| A_33_P3264121 | TBCA | NM_004607 | -1.52 | 2.15E-04 |
| A_23_P64019 | MTMR2 | NM_201278 | -1.52 | 2.92E-04 |
| A_23_P213661 | PPIP5K2 | NM_015216 | -1.52 | 2.85E-05 |
| A_24_P81841 | CDKN1B | NM_004064 | -1.52 | 1.70E-04 |
| A_33_P3236868 | MT1X | NM_005952 | -1.52 | 2.29E-04 |
| A_23_P130304 | TXNL4A | NM_006701 | -1.52 | 7.51E-06 |
| A_32_P225604 | RPL5 | NM_000969 | -1.52 | 7.41E-05 |
| A_23_P104876 | SPA17 | NM_017425 | -1.52 | 1.15E-04 |
| A_33_P3328736 | CCDC23 | NM_199342 | -1.52 | 7.00E-05 |
| A_23_P12336 | PRMT6 | NM_018137 | -1.52 | 9.12E-05 |
| A_23_P82334 | SLC25A13 | NM_014251 | -1.52 | 4.82E-05 |
| A_23_P57658 | HRASLS | NM_020386 | -1.52 | 6.15E-04 |
| A_23_P123256 | PDAP1 | NM_014891 | -1.52 | 6.14E-04 |
| A_23_P106145 | ERO1L | NM_014584 | -1.52 | 2.30E-05 |
| A_23_P119102 | VASP | NM_003370 | -1.52 | 3.81E-04 |
| A_21_P0014456 | LOC100505634 | XR_109905 | -1.52 | 3.69E-04 |
| A_23_P216149 | TERF1 | NM_017489 | -1.52 | 2.39E-04 |
| A_23_P102420 | CCT4 | NM_006430 | -1.51 | 5.12E-04 |
| A_23_P213661 | PPIP5K2 | NM_015216 | -1.51 | 6.16E-04 |
| A_23_P104201 | YME1L1 | NM_139312 | -1.51 | 2.94E-05 |
| A_24_P551028 | SPOPL | NM_001001664 | -1.51 | 1.70E-04 |
| A_24_P169574 | LOC649395 | NR_029404 | -1.51 | 2.27E-04 |
| A_23_P154315 | MRPS9 | NM_182640 | -1.51 | 1.63E-04 |
| A_23_P30024 | NFKB1 | NM_003998 | -1.51 | 4.66E-06 |
| A_24_P81841 | CDKN1B | NM_004064 | -1.51 | 2.81E-04 |
| A_33_P3264505 | FCF1 | NM_015962 | -1.51 | 6.33E-04 |
| A_23_P106145 | ERO1L | NM_014584 | -1.51 | 9.39E-04 |
| A_33_P3367102 | PFN1P2 | NR_003242 | -1.51 | 2.84E-05 |
| A_23_P111811 | ING3 | NM_019071 | -1.51 | 3.59E-06 |
| A_19_P00322754 | SNHG5 | NR_003038 | -1.51 | 1.62E-04 |
| A_23_P58647 | CTNNA1 | NM_001903 | -1.51 | 3.65E-04 |
| A_23_P104876 | SPA17 | NM_017425 | -1.51 | 1.23E-04 |
| A_23_P115645 | CELF2 | NM_001025076 | -1.51 | 2.65E-04 |
| A_23_P23765 | ITGB3BP | NM_014288 | -1.51 | 1.91E-04 |
| A_33_P3362869 | DNAJC19 | NM_001190233 | -1.51 | 2.61E-05 |
| A_33_P3221489 | KIRREL | NM_018240 | -1.51 | 2.84E-05 |
| A_23_P213661 | PPIP5K2 | NM_015216 | -1.51 | 7.09E-04 |
| A_23_P104025 | TSEN15 | NM_052965 | -1.51 | 2.08E-04 |
| A_19_P00318587 | HP07349 | XM_003403534 | -1.51 | 3.85E-05 |
| A_23_P93009 | SRP19 | NM_001204199 | -1.51 | 7.44E-04 |
| A_23_P138435 | ZMIZ1 | NM_020338 | -1.51 | 9.60E-04 |
| A_33_P3319331 | PRDM2 | AK123605 | -1.51 | 5.18E-05 |
| A_24_P211151 | EXOSC5 | NM_020158 | -1.51 | 5.25E-04 |
| A_33_P3287710 | LOC100128292 | NR_024585 | -1.51 | 6.31E-04 |
| A_32_P191004 | ATAD2B | NM_017552 | -1.51 | 5.12E-04 |
| A_23_P102420 | CCT4 | NM_006430 | -1.51 | 4.54E-04 |
| A_23_P104201 | YME1L1 | NM_139312 | -1.51 | 7.25E-04 |
| A_33_P3344831 | TMEM45A | NM_018004 | -1.51 | 6.42E-04 |
| A_33_P3351092 | KRTAP20-2 | NM_181616 | -1.51 | 2.88E-04 |
| A_33_P3422812 | C7orf73 | NM_001130929 | -1.51 | 8.66E-04 |
| A_23_P213661 | PPIP5K2 | NM_015216 | -1.51 | 3.17E-05 |
| A_23_P1361 | ALDH18A1 | NM_002860 | -1.51 | 2.35E-04 |
| A_23_P30024 | NFKB1 | NM_003998 | -1.51 | 2.83E-06 |
| A_23_P334123 | ITFG1 | NM_030790 | -1.51 | 1.24E-04 |
| A_24_P50458 | TERF1 | NM_017489 | -1.51 | 3.98E-04 |
| A_23_P418413 | OXSR1 | NM_005109 | -1.51 | 6.30E-04 |
| A_23_P138881 | ACTN3 | NM_001104 | -1.51 | 7.12E-05 |
| A_33_P3249716 | ENST00000415640 | ENST00000415640 | -1.51 | 8.60E-04 |
| A_23_P98085 | PTEN | NM_000314 | -1.51 | 9.24E-04 |
| A_33_P3272165 | C8orf38 | NM_152416 | -1.51 | 5.05E-04 |
| A_23_P24997 | CDK4 | NM_000075 | -1.51 | 4.93E-04 |
| A_33_P3368328 | PHB2 | NM_007273 | -1.51 | 6.70E-04 |
| A_23_P100203 | HSBP1 | NM_001537 | -1.51 | 1.33E-05 |
| A_24_P313262 | ARPP19 | NM_006628 | -1.51 | 5.86E-05 |
| A_32_P184367 | RPL10 | NM_006013 | -1.51 | 6.33E-05 |
| A_23_P73493 | CETN2 | NM_004344 | -1.51 | 1.35E-04 |
| A_33_P3270581 | ENST00000308739 | ENST00000308739 | -1.50 | 1.08E-04 |
| A_23_P122674 | PAK1IP1 | NM_017906 | -1.50 | 8.41E-04 |
| A_23_P104942 | TMX2 | NM_015959 | -1.50 | 5.07E-04 |
| A_24_P272873 | RPL13AP3 | NR_004844 | -1.50 | 5.35E-05 |
| A_24_P106357 | WDR36 | NM_139281 | -1.50 | 1.91E-04 |
| A_23_P78410 | C18orf55 | NM_014177 | -1.50 | 3.16E-04 |
| A_23_P105571 | CHPT1 | NM_020244 | -1.50 | 1.44E-04 |
| A_24_P81841 | CDKN1B | NM_004064 | -1.50 | 3.25E-05 |
| A_23_P2474 | COPS7A | NM_016319 | -1.50 | 7.27E-04 |
| A_23_P100203 | HSBP1 | NM_001537 | -1.50 | 9.95E-04 |
| A_23_P144476 | SPRY1 | NM_199327 | -1.50 | 7.33E-04 |
| A_33_P3577142 | LOC283861 | AK098143 | -1.50 | 9.88E-04 |
| A_23_P213883 | NIPBL | NM_133433 | -1.50 | 1.18E-04 |
| A_32_P158746 | RPL17 | NM_000985 | -1.50 | 8.33E-04 |
| A_23_P336796 | GXYLT1 | NM_173601 | -1.50 | 4.07E-04 |
| A_23_P117068 | SNRPF | NM_003095 | -1.50 | 8.61E-04 |
| A_32_P49350 | THC2562448 | THC2562448 | -1.50 | 3.31E-04 |
| A_32_P184796 | RPLP0 | NM_053275 | -1.50 | 1.08E-04 |
| A_23_P215790 | EGFR | NM_005228 | -1.50 | 2.01E-04 |
| A_33_P3258782 | AP1S2 | NM_003916 | -1.50 | 2.22E-04 |
| A_33_P3290888 | CPNE1 | NM_003915 | -1.50 | 7.64E-05 |
| A_23_P374782 | SH3KBP1 | NM_001024666 | -1.50 | 1.13E-04 |
| A_33_P3252196 | EZH2 | NM_004456 | -1.50 | 1.66E-04 |
| A_33_P3246163 | RPL5 | NM_000969 | -1.50 | 2.31E-04 |
| A_32_P192545 | TCEAL6 | NM_001006938 | 1.50 | 1.57E-05 |
| A_32_P182473 | ZNF625 | NM_145233 | 1.50 | 5.77E-04 |
| A_24_P319923 | MYLK | NM_053025 | 1.50 | 2.16E-04 |
| A_23_P118406 | C17orf62 | NM_001033046 | 1.50 | 9.60E-04 |
| A_33_P3805090 | FNIP2 | NM_020840 | 1.50 | 2.20E-04 |
| A_23_P36753 | ALDH2 | NM_000690 | 1.51 | 3.60E-05 |
| A_23_P121215 | CAMK1 | NM_003656 | 1.51 | 3.43E-05 |
| A_23_P349771 | HAUS5 | NM_015302 | 1.51 | 9.49E-04 |
| A_24_P132039 | RNF14 | NM_004290 | 1.51 | 8.92E-05 |
| A_24_P151498 | PRMT2 | NM_206962 | 1.51 | 5.53E-05 |
| A_33_P3380417 | SLC25A30 | NM_001010875 | 1.51 | 1.38E-04 |
| A_23_P39402 | ALKBH6 | NM_198867 | 1.51 | 3.24E-04 |
| A_23_P15394 | CD68 | NM_001251 | 1.51 | 3.10E-04 |
| A_23_P166775 | IL17RC | NM_153461 | 1.51 | 1.02E-05 |
| A_23_P159937 | SLC6A8 | NM_005629 | 1.51 | 2.95E-04 |
| A_24_P750305 | LOC643837 | NR_015368 | 1.51 | 4.48E-04 |
| A_33_P3406899 | TRAK1 | NM_014965 | 1.51 | 3.94E-04 |
| A_21_P0011384 | UBE2Q2P1 | NR_003661 | 1.51 | 9.85E-05 |
| A_33_P3357087 | MPV17 | NM_002437 | 1.51 | 9.08E-04 |
| A_24_P143492 | BCAS4 | NM_001010974 | 1.51 | 3.68E-04 |
| A_19_P00315824 | LOC100505495 | NR_040109 | 1.51 | 1.21E-04 |
| A_19_P00322371 | LOC100506190 | BC038559 | 1.51 | 9.21E-04 |
| A_23_P36825 | GPRC5A | NM_003979 | 1.51 | 1.40E-04 |
| A_33_P3215288 | LOC284757 | AK128288 | 1.51 | 5.49E-05 |
| A_24_P38081 | FKBP5 | NM_004117 | 1.51 | 6.90E-04 |
| A_23_P108835 | YPEL5 | NM_016061 | 1.51 | 5.67E-04 |
| A_23_P315589 | KIAA0930 | NM_015264 | 1.51 | 2.07E-04 |
| A_23_P102109 | TUBA4A | NM_006000 | 1.51 | 3.91E-06 |
| A_23_P18684 | CLGN | NM_004362 | 1.51 | 4.67E-04 |
| A_24_P405621 | NISCH | NM_007184 | 1.51 | 2.29E-04 |
| A_21_P0001340 | XLOC_000301 | TCONS_00001034 | 1.52 | 4.83E-04 |
| A_24_P45005 | NPEPL1 | NM_024663 | 1.52 | 6.73E-04 |
| A_33_P3313245 | AMACR | NM_001167595 | 1.52 | 6.20E-04 |
| A_23_P106727 | RAB11FIP3 | NM_014700 | 1.52 | 4.89E-04 |
| A_33_P3266396 | GLTPD1 | NM_001029885 | 1.52 | 2.32E-04 |
| A_24_P117138 | ZNF626 | NM_145297 | 1.52 | 7.78E-04 |
| A_32_P76853 | LOC653075 | NR_033933 | 1.52 | 5.25E-04 |
| A_19_P00322977 | FLJ43663 | NR_015431 | 1.52 | 2.36E-04 |
| A_23_P157809 | PTGR1 | NM_012212 | 1.52 | 2.43E-04 |
| A_23_P140146 | IFI27L2 | NM_032036 | 1.52 | 1.96E-04 |
| A_19_P00316333 | LOC100506714 | NR_038956 | 1.52 | 3.39E-04 |
| A_23_P137543 | ZNF362 | NM_152493 | 1.52 | 6.36E-04 |
| A_33_P3230219 | TMEM54 | NM_033504 | 1.52 | 1.91E-04 |
| A_24_P120537 | SH3RF2 | NM_152550 | 1.52 | 3.50E-04 |
| A_23_P70355 | SERPINB6 | NM_004568 | 1.52 | 1.05E-05 |
| A_19_P00318494 | LINC00467 | NR_026761 | 1.52 | 1.11E-04 |
| A_21_P0007221 | XLOC_009181 | ENST00000535746 | 1.52 | 8.03E-04 |
| A_24_P177964 | NCKAP5L | NM_001037806 | 1.52 | 1.72E-05 |
| A_23_P47410 | ESAM | NM_138961 | 1.53 | 3.64E-04 |
| A_33_P3366053 | ADPRH | NM_001125 | 1.53 | 4.90E-04 |
| A_23_P114947 | RGS2 | NM_002923 | 1.53 | 4.54E-04 |
| A_24_P369232 | CCDC3 | NM_031455 | 1.53 | 3.59E-04 |
| A_23_P111273 | TBC1D7 | NM_016495 | 1.53 | 2.54E-04 |
| A_24_P148094 | LEPROT | NM_017526 | 1.53 | 1.27E-04 |
| A_23_P20832 | SPTAN1 | NM_003127 | 1.53 | 5.38E-04 |
| A_24_P318967 | PDXK | NM_003681 | 1.53 | 4.94E-04 |
| A_23_P111273 | TBC1D7 | NM_016495 | 1.53 | 3.16E-04 |
| A_23_P30655 | NFKBIE | NM_004556 | 1.53 | 7.89E-04 |
| A_23_P106727 | RAB11FIP3 | NM_014700 | 1.53 | 9.32E-05 |
| A_23_P156739 | C6orf125 | NM_032340 | 1.53 | 7.74E-04 |
| A_24_P190168 | TMEM97 | NM_014573 | 1.53 | 7.70E-04 |
| A_23_P106727 | RAB11FIP3 | NM_014700 | 1.53 | 4.34E-04 |
| A_24_P79054 | TGFB1 | NM_000660 | 1.53 | 4.36E-04 |
| A_23_P91430 | PXMP4 | NM_007238 | 1.53 | 6.59E-07 |
| A_23_P1602 | CDC42EP2 | NM_006779 | 1.54 | 1.75E-04 |
| A_23_P77430 | PRMT7 | NM_019023 | 1.54 | 8.99E-04 |
| A_24_P215804 | CKLF | NM_016951 | 1.54 | 3.13E-05 |
| A_23_P4036 | TEX2 | NM_018469 | 1.54 | 2.21E-04 |
| A_33_P3218148 | C5orf42 | NM_023073 | 1.54 | 3.23E-04 |
| A_33_P3297217 | NAAA | NM_001042402 | 1.54 | 9.61E-04 |
| A_33_P3390032 | EXOC7 | NM_001145297 | 1.54 | 1.28E-06 |
| A_33_P3224809 | IL17RA | NM_014339 | 1.54 | 6.74E-05 |
| A_33_P3240946 | DPF3 | ENST00000366353 | 1.54 | 6.43E-04 |
| A_23_P48717 | NPC2 | NM_006432 | 1.54 | 4.10E-04 |
| A_23_P108835 | YPEL5 | NM_016061 | 1.54 | 5.41E-04 |
| A_33_P3361152 | LOC100134259 | NR_024452 | 1.54 | 8.91E-04 |
| A_23_P152963 | C17orf59 | NM_017622 | 1.54 | 4.77E-04 |
| A_24_P141214 | STOM | NM_198194 | 1.54 | 1.18E-04 |
| A_33_P3271800 | SIRT2 | NM_012237 | 1.54 | 4.12E-05 |
| A_24_P360269 | RNASET2 | NM_003730 | 1.55 | 1.38E-05 |
| A_33_P3389286 | SFN | NM_006142 | 1.55 | 3.00E-04 |
| A_23_P106727 | RAB11FIP3 | NM_014700 | 1.55 | 6.70E-04 |
| A_33_P3220698 | EPS8L1 | NM_133180 | 1.55 | 2.22E-04 |
| A_23_P154962 | RIMBP3 | NM_015672 | 1.55 | 9.63E-06 |
| A_23_P121064 | PTX3 | NM_002852 | 1.55 | 3.99E-04 |
| A_23_P205336 | C14orf129 | NM_016472 | 1.55 | 1.50E-04 |
| A_32_P56525 | FAM115A | NM_014719 | 1.55 | 8.99E-05 |
| A_33_P3361457 | IFNAR2 | NM_000874 | 1.55 | 8.97E-04 |
| A_23_P148584 | DOCK11 | NM_144658 | 1.55 | 1.12E-04 |
| A_21_P0011681 | XLOC_l2_006648 | ENST00000360204 | 1.55 | 9.36E-04 |
| A_23_P123672 | TDRD7 | NM_014290 | 1.55 | 7.72E-04 |
| A_21_P0012391 | XLOC_l2_009883 | ENST00000468859 | 1.56 | 7.14E-04 |
| A_33_P3239839 | FBXL18 | NM_024963 | 1.56 | 9.75E-04 |
| A_23_P201287 | KIF1B | NM_015074 | 1.56 | 1.89E-04 |
| A_21_P0009319 | XLOC_012457 | THC2665248 | 1.56 | 2.10E-04 |
| A_33_P3390580 | CCDC144A | NM_014695 | 1.56 | 3.65E-04 |
| A_23_P108835 | YPEL5 | NM_016061 | 1.56 | 4.91E-04 |
| A_24_P22079 | FOXO1 | NM_002015 | 1.56 | 4.05E-04 |
| A_24_P16815 | ZMYM3 | NM_005096 | 1.56 | 1.23E-04 |
| A_23_P136347 | EPS8 | NM_004447 | 1.56 | 8.38E-04 |
| A_33_P3387365 | PXMP4 | NM_007238 | 1.56 | 3.14E-05 |
| A_23_P115407 | GSTM1 | NM_146421 | 1.56 | 6.09E-05 |
| A_32_P46981 | HSBP1L1 | NM_001136180 | 1.56 | 1.21E-04 |
| A_33_P3383233 | NDRG2 | NM_201535 | 1.57 | 3.15E-04 |
| A_23_P115407 | GSTM1 | NM_146421 | 1.57 | 8.75E-06 |
| A_23_P111273 | TBC1D7 | NM_016495 | 1.57 | 5.29E-04 |
| A_23_P134085 | CNKSR3 | NM_173515 | 1.57 | 5.53E-05 |
| A_23_P251717 | WDR45 | NM_007075 | 1.57 | 8.63E-04 |
| A_33_P3303136 | SERPINB6 | NM_001195291 | 1.57 | 2.24E-04 |
| A_23_P131846 | SNAI1 | NM_005985 | 1.57 | 7.21E-04 |
| A_23_P2355 | CBX5 | NM_012117 | 1.57 | 3.66E-04 |
| A_23_P100654 | ZBTB4 | NM_020899 | 1.57 | 6.86E-04 |
| A_23_P376096 | TICAM1 | NM_182919 | 1.57 | 6.75E-05 |
| A_24_P261929 | IFI27L1 | NM_206949 | 1.57 | 4.03E-05 |
| A_23_P106727 | RAB11FIP3 | NM_014700 | 1.57 | 2.37E-04 |
| A_24_P274270 | STAT1 | NM_139266 | 1.57 | 6.45E-05 |
| A_23_P1014 | LINC00467 | NR_026761 | 1.57 | 4.14E-04 |
| A_23_P162211 | MANSC1 | NM_018050 | 1.57 | 6.97E-04 |
| A_23_P12147 | C1orf74 | NM_152485 | 1.57 | 1.31E-05 |
| A_23_P108835 | YPEL5 | NM_016061 | 1.57 | 1.74E-04 |
| A_23_P500892 | TUB | NM_003320 | 1.57 | 7.86E-04 |
| A_21_P0009558 | XLOC_012847 | TCONS_00026523 | 1.57 | 8.17E-05 |
| A_33_P3223097 | LINC00467 | NR_026761 | 1.57 | 6.98E-04 |
| A_23_P108835 | YPEL5 | NM_016061 | 1.57 | 1.11E-04 |
| A_23_P106727 | RAB11FIP3 | NM_014700 | 1.58 | 2.66E-04 |
| A_23_P123916 | LRSAM1 | NM_138361 | 1.58 | 2.38E-04 |
| A_24_P206047 | SLC25A4 | NM_001151 | 1.58 | 2.03E-06 |
| A_23_P148121 | EHBP1L1 | NM_001099409 | 1.58 | 2.05E-04 |
| A_23_P73747 | ARMCX2 | NM_014782 | 1.58 | 1.66E-04 |
| A_23_P110569 | TRIM36 | NM_018700 | 1.58 | 6.48E-04 |
| A_23_P115407 | GSTM1 | NM_146421 | 1.58 | 5.55E-05 |
| A_23_P108835 | YPEL5 | NM_016061 | 1.58 | 8.54E-04 |
| A_21_P0011148 | XLOC_l2_003757 | THC2581448 | 1.58 | 4.37E-04 |
| A_23_P152115 | NME3 | NM_002513 | 1.58 | 1.10E-04 |
| A_32_P163247 | CD8A | NM_001768 | 1.58 | 6.30E-05 |
| A_24_P160104 | TUBA8 | NM_018943 | 1.58 | 9.88E-04 |
| A_24_P210829 | NME4 | NM_005009 | 1.58 | 3.03E-04 |
| A_23_P379475 | DHCR24 | NM_014762 | 1.58 | 1.95E-04 |
| A_21_P0000080 | FAM122C | NM_001170781 | 1.59 | 1.99E-04 |
| A_21_P0011143 | XLOC_l2_003732 | TCONS_l2_00006853 | 1.59 | 4.59E-04 |
| A_33_P3358243 | KIAA1161 | NM_020702 | 1.59 | 1.15E-04 |
| A_24_P274270 | STAT1 | NM_139266 | 1.59 | 2.48E-04 |
| A_24_P79054 | TGFB1 | NM_000660 | 1.59 | 9.65E-04 |
| A_33_P3305790 | NOS3 | NM_000603 | 1.59 | 1.82E-04 |
| A_23_P401106 | PDE2A | NM_002599 | 1.59 | 3.56E-04 |
| A_23_P83599 | PRKAR1B | NM_002735 | 1.59 | 1.70E-04 |
| A_23_P115407 | GSTM1 | NM_146421 | 1.59 | 4.14E-04 |
| A_23_P165937 | DSN1 | NM_024918 | 1.59 | 2.61E-04 |
| A_23_P143817 | MYLK | NM_053025 | 1.59 | 4.52E-05 |
| A_23_P115407 | GSTM1 | NM_146421 | 1.59 | 7.14E-05 |
| A_33_P3409513 | MAPK11 | NM_002751 | 1.59 | 3.73E-04 |
| A_23_P120883 | HMOX1 | NM_002133 | 1.59 | 5.46E-04 |
| A_23_P123672 | TDRD7 | NM_014290 | 1.59 | 1.76E-04 |
| A_33_P3408962 | FLJ33996 | XR_110352 | 1.59 | 9.80E-04 |
| A_23_P111888 | CTHRC1 | NM_138455 | 1.59 | 3.52E-04 |
| A_24_P152968 | AKR1C1 | NM_001353 | 1.59 | 1.52E-04 |
| A_23_P1014 | LINC00467 | NR_026761 | 1.59 | 6.87E-04 |
| A_23_P14072 | KRT8 | NM_002273 | 1.59 | 2.96E-04 |
| A_23_P415006 | RAB11FIP5 | NM_015470 | 1.59 | 3.56E-06 |
| A_33_P3397865 | TNNT1 | NM_003283 | 1.59 | 9.28E-04 |
| A_33_P3282489 | GCNT1 | NM_001097634 | 1.59 | 4.55E-04 |
| A_23_P106727 | RAB11FIP3 | NM_014700 | 1.60 | 1.35E-04 |
| A_33_P3334443 | FAM69A | NM_001252271 | 1.60 | 7.49E-04 |
| A_23_P216489 | GNE | NM_005476 | 1.60 | 1.14E-05 |
| A_23_P387471 | MICB | NM_005931 | 1.60 | 7.36E-05 |
| A_23_P106727 | RAB11FIP3 | NM_014700 | 1.60 | 6.57E-05 |
| A_23_P118061 | CKLF | NM_181641 | 1.60 | 1.35E-04 |
| A_33_P3419945 | AK130724 | AK130724 | 1.60 | 5.60E-04 |
| A_23_P1014 | LINC00467 | NR_026761 | 1.60 | 2.82E-04 |
| A_23_P204947 | GJB2 | NM_004004 | 1.60 | 6.29E-04 |
| A_23_P115407 | GSTM1 | NM_146421 | 1.60 | 2.69E-05 |
| A_23_P121064 | PTX3 | NM_002852 | 1.60 | 8.62E-04 |
| A_33_P3222788 | LOC100188947 | NR_024467 | 1.60 | 1.68E-04 |
| A_23_P44942 | CCDC15 | NM_025004 | 1.61 | 7.45E-04 |
| A_23_P127460 | SIPA1 | NM_153253 | 1.61 | 2.77E-04 |
| A_23_P12147 | C1orf74 | NM_152485 | 1.61 | 1.44E-04 |
| A_24_P274270 | STAT1 | NM_139266 | 1.61 | 3.27E-05 |
| A_23_P113005 | EFNA1 | NM_004428 | 1.61 | 5.65E-04 |
| A_23_P115792 | PLEKHA1 | NM_001001974 | 1.61 | 1.93E-04 |
| A_23_P127394 | CRY2 | NM_021117 | 1.61 | 5.67E-04 |
| A_23_P205031 | COL4A2 | NM_001846 | 1.61 | 2.56E-04 |
| A_23_P53276 | TIMELESS | NM_003920 | 1.61 | 3.61E-04 |
| A_23_P120883 | HMOX1 | NM_002133 | 1.61 | 6.93E-05 |
| A_24_P45367 | NIPAL3 | NM_020448 | 1.61 | 1.61E-05 |
| A_23_P113005 | EFNA1 | NM_004428 | 1.61 | 4.41E-05 |
| A_24_P289471 | RNASET2 | NM_003730 | 1.61 | 1.91E-04 |
| A_24_P364236 | NDUFC2 | NM_004549 | 1.62 | 5.62E-04 |
| A_24_P79054 | TGFB1 | NM_000660 | 1.62 | 9.44E-04 |
| A_23_P109269 | LAMA5 | NM_005560 | 1.62 | 1.37E-04 |
| A_33_P3235716 | SPSB3 | NM_080861 | 1.62 | 1.31E-05 |
| A_23_P121064 | PTX3 | NM_002852 | 1.62 | 2.12E-04 |
| A_23_P201264 | NMNAT1 | NM_022787 | 1.62 | 6.35E-04 |
| A_23_P1014 | LINC00467 | NR_026761 | 1.62 | 5.46E-05 |
| A_23_P120883 | HMOX1 | NM_002133 | 1.62 | 6.18E-05 |
| A_23_P128094 | ABCB9 | NM_019625 | 1.62 | 6.59E-04 |
| A_23_P121064 | PTX3 | NM_002852 | 1.62 | 5.81E-04 |
| A_33_P3240532 | RGL1 | NM_015149 | 1.62 | 6.04E-04 |
| A_23_P330616 | WIPF1 | NM_001077269 | 1.62 | 3.82E-04 |
| A_23_P399255 | RNF182 | NM_152737 | 1.62 | 1.04E-04 |
| A_33_P3772937 | KRT8P12 | AL133645 | 1.62 | 1.56E-04 |
| A_24_P212539 | GALM | NM_138801 | 1.62 | 4.00E-05 |
| A_23_P1014 | LINC00467 | NR_026761 | 1.62 | 9.65E-05 |
| A_23_P1014 | LINC00467 | NR_026761 | 1.62 | 2.74E-04 |
| A_21_P0010981 | MALAT1 | NR_002819 | 1.62 | 1.39E-04 |
| A_23_P72025 | SLC25A20 | NM_000387 | 1.62 | 3.97E-05 |
| A_23_P121064 | PTX3 | NM_002852 | 1.62 | 5.83E-05 |
| A_21_P0000138 | BTN2A2 | NM_001197238 | 1.62 | 2.43E-04 |
| A_23_P115407 | GSTM1 | NM_146421 | 1.63 | 2.27E-04 |
| A_23_P212696 | FSTL1 | NM_007085 | 1.63 | 9.28E-05 |
| A_33_P3219965 | TCIRG1 | NM_006019 | 1.63 | 3.66E-04 |
| A_23_P120883 | HMOX1 | NM_002133 | 1.63 | 4.50E-04 |
| A_33_P3231267 | LOC257396 | BC041894 | 1.63 | 8.96E-04 |
| A_23_P358195 | GOLGA6L10 | NM_001164465 | 1.63 | 5.61E-04 |
| A_23_P115407 | GSTM1 | NM_146421 | 1.63 | 1.91E-04 |
| A_23_P12147 | C1orf74 | NM_152485 | 1.63 | 6.78E-04 |
| A_24_P390928 | TRAPPC6A | NM_024108 | 1.63 | 3.17E-04 |
| A_23_P83781 | CYTH1 | NM_004762 | 1.63 | 1.16E-04 |
| A_24_P274270 | STAT1 | NM_139266 | 1.63 | 2.04E-05 |
| A_23_P105545 | VAMP1 | NM_016830 | 1.63 | 5.35E-04 |
| A_23_P67971 | GALM | NM_138801 | 1.63 | 3.40E-05 |
| A_33_P3305731 | NINL | NM_025176 | 1.63 | 4.91E-04 |
| A_33_P3234697 | LXN | NM_020169 | 1.63 | 1.01E-04 |
| A_23_P120883 | HMOX1 | NM_002133 | 1.63 | 6.29E-04 |
| A_23_P108835 | YPEL5 | NM_016061 | 1.63 | 1.62E-04 |
| A_21_P0014446 | HHIP | ENST00000296575 | 1.63 | 5.17E-04 |
| A_21_P0014132 | LOC100505787 | XR_108528 | 1.64 | 7.10E-05 |
| A_23_P45475 | GLA | NM_000169 | 1.64 | 5.41E-05 |
| A_23_P12147 | C1orf74 | NM_152485 | 1.64 | 4.67E-04 |
| A_23_P120883 | HMOX1 | NM_002133 | 1.64 | 1.72E-05 |
| A_33_P3289406 | LOC91450 | NR_026998 | 1.64 | 4.44E-05 |
| A_23_P1014 | LINC00467 | NR_026761 | 1.64 | 7.01E-04 |
| A_23_P96599 | TMSB15B | NM_194324 | 1.64 | 2.42E-04 |
| A_23_P77437 | PRMT7 | NM_019023 | 1.64 | 5.94E-04 |
| A_23_P205200 | DHRS12 | NM_024705 | 1.64 | 3.96E-04 |
| A_33_P3262833 | GOLGA8E | NR_033350 | 1.64 | 4.62E-04 |
| A_23_P113005 | EFNA1 | NM_004428 | 1.64 | 9.13E-04 |
| A_33_P3317321 | TPMT | NM_000367 | 1.64 | 1.91E-04 |
| A_24_P274270 | STAT1 | NM_139266 | 1.64 | 1.03E-06 |
| A_23_P52207 | BAMBI | NM_012342 | 1.64 | 4.97E-04 |
| A_33_P3346826 | IL32 | NM_001012633 | 1.64 | 1.78E-04 |
| A_33_P3411848 | CNFN | NM_032488 | 1.64 | 4.20E-04 |
| A_24_P295010 | SERPINB9 | NM_004155 | 1.64 | 4.89E-04 |
| A_23_P11598 | ZNF684 | NM_152373 | 1.64 | 1.82E-04 |
| A_33_P3297978 | MYO1E | NM_004998 | 1.64 | 8.99E-05 |
| A_23_P205997 | APH1B | NM_031301 | 1.64 | 1.19E-05 |
| A_24_P274270 | STAT1 | NM_139266 | 1.64 | 2.37E-06 |
| A_23_P1014 | LINC00467 | NR_026761 | 1.64 | 5.53E-04 |
| A_33_P3325723 | CHN1 | NM_001822 | 1.64 | 5.43E-05 |
| A_23_P127367 | POLD4 | NM_021173 | 1.64 | 5.94E-04 |
| A_23_P121064 | PTX3 | NM_002852 | 1.64 | 4.06E-04 |
| A_33_P3363425 | FRMD3 | NM_174938 | 1.64 | 1.56E-04 |
| A_24_P229669 | GOLGA8IP | NR_024074 | 1.65 | 2.91E-04 |
| A_24_P113264 | FBXO27 | NM_178820 | 1.65 | 3.57E-04 |
| A_23_P76364 | CD9 | NM_001769 | 1.65 | 8.91E-05 |
| A_23_P12147 | C1orf74 | NM_152485 | 1.65 | 1.34E-05 |
| A_23_P216556 | EPB41L4B | NM_018424 | 1.65 | 5.24E-04 |
| A_23_P259090 | NUDT12 | NM_031438 | 1.65 | 5.32E-04 |
| A_21_P0011129 | TPTE2P6 | NR_002815 | 1.65 | 2.14E-05 |
| A_23_P352684 | DCAF5 | NM_003861 | 1.65 | 1.85E-04 |
| A_23_P107963 | FUT1 | NM_000148 | 1.65 | 5.42E-06 |
| A_33_P3219651 | BMPER | NM_133468 | 1.65 | 2.02E-04 |
| A_23_P29855 | USO1 | NM_003715 | 1.65 | 1.98E-04 |
| A_19_P00319675 | LOC100506190 | BC038559 | 1.65 | 6.97E-04 |
| A_23_P120883 | HMOX1 | NM_002133 | 1.65 | 1.40E-04 |
| A_33_P3229953 | EEF1A2 | NM_001958 | 1.65 | 1.20E-04 |
| A_23_P300484 | OBSL1 | NM_001173408 | 1.65 | 1.48E-04 |
| A_23_P115407 | GSTM1 | NM_146421 | 1.65 | 4.25E-05 |
| A_23_P55936 | FCGRT | NM_004107 | 1.65 | 1.08E-04 |
| A_33_P3222630 | FBXO43 | NM_001029860 | 1.65 | 6.98E-05 |
| A_24_P117177 | SNX21 | NM_152897 | 1.65 | 2.21E-04 |
| A_33_P3288189 | RHOBTB3 | NM_014899 | 1.65 | 5.44E-05 |
| A_23_P120883 | HMOX1 | NM_002133 | 1.65 | 6.23E-04 |
| A_24_P167642 | GCH1 | NM_000161 | 1.65 | 6.63E-04 |
| A_23_P24586 | ACCS | NM_032592 | 1.66 | 6.16E-04 |
| A_23_P105012 | HRASLS2 | NM_017878 | 1.66 | 3.19E-04 |
| A_24_P203689 | KIF7 | NM_198525 | 1.66 | 2.03E-04 |
| A_24_P79054 | TGFB1 | NM_000660 | 1.66 | 3.04E-04 |
| A_19_P00322793 | LOC100506190 | BC038559 | 1.66 | 1.34E-04 |
| A_23_P208698 | GYS1 | NM_002103 | 1.66 | 8.68E-04 |
| A_23_P122662 | GFOD1 | NM_018988 | 1.66 | 1.68E-04 |
| A_24_P188377 | CD55 | NM_000574 | 1.66 | 3.18E-04 |
| A_33_P3269803 | CLSTN3 | NM_014718 | 1.66 | 2.45E-04 |
| A_23_P389588 | TCF7L2 | NM_030756 | 1.66 | 6.40E-04 |
| A_23_P122662 | GFOD1 | NM_018988 | 1.66 | 6.94E-04 |
| A_23_P121064 | PTX3 | NM_002852 | 1.66 | 2.63E-04 |
| A_23_P255076 | RWDD2A | NM_033411 | 1.66 | 2.47E-04 |
| A_23_P122662 | GFOD1 | NM_018988 | 1.66 | 1.76E-04 |
| A_23_P23194 | PINK1 | NM_032409 | 1.66 | 7.52E-04 |
| A_24_P79054 | TGFB1 | NM_000660 | 1.66 | 3.32E-05 |
| A_33_P3334220 | ACACB | NM_001093 | 1.66 | 4.50E-04 |
| A_33_P3241786 | ADD2 | NM_017482 | 1.66 | 2.01E-04 |
| A_23_P74359 | CSRP1 | NM_004078 | 1.66 | 7.53E-04 |
| A_33_P3230723 | KIF1B | NM_183416 | 1.66 | 6.08E-04 |
| A_23_P29067 | TMPRSS2 | NM_005656 | 1.66 | 7.65E-04 |
| A_33_P3246985 | PDE4DIP | NM_022359 | 1.66 | 2.46E-05 |
| A_23_P118633 | SPATA20 | NM_022827 | 1.66 | 1.03E-04 |
| A_24_P79054 | TGFB1 | NM_000660 | 1.66 | 8.45E-04 |
| A_23_P120883 | HMOX1 | NM_002133 | 1.66 | 6.24E-04 |
| A_24_P394533 | NEU1 | NM_000434 | 1.66 | 8.63E-04 |
| A_23_P209735 | ARMC9 | NM_025139 | 1.66 | 3.99E-05 |
| A_23_P115407 | GSTM1 | NM_146421 | 1.66 | 8.11E-06 |
| A_23_P147495 | BCORL1 | NM_021946 | 1.66 | 2.08E-04 |
| A_24_P274270 | STAT1 | NM_139266 | 1.67 | 2.69E-04 |
| A_24_P79054 | TGFB1 | NM_000660 | 1.67 | 9.21E-04 |
| A_23_P122662 | GFOD1 | NM_018988 | 1.67 | 3.85E-04 |
| A_23_P122662 | GFOD1 | NM_018988 | 1.67 | 1.32E-04 |
| A_23_P431305 | FAM69B | NM_152421 | 1.67 | 7.01E-04 |
| A_23_P29248 | TST | NM_003312 | 1.67 | 4.60E-04 |
| A_23_P149992 | PDLIM1 | NM_020992 | 1.67 | 6.22E-05 |
| A_24_P579356 | ARHGAP28 | NM_001010000 | 1.67 | 5.04E-04 |
| A_23_P122662 | GFOD1 | NM_018988 | 1.67 | 5.45E-05 |
| A_23_P254831 | MAGEB2 | NM_002364 | 1.67 | 8.05E-05 |
| A_23_P122662 | GFOD1 | NM_018988 | 1.67 | 9.91E-04 |
| A_23_P122662 | GFOD1 | NM_018988 | 1.67 | 1.87E-04 |
| A_19_P00316467 | XLOC_002736 | ENST00000473756 | 1.67 | 8.43E-04 |
| A_23_P37415 | SECISBP2L | NM_014701 | 1.67 | 1.23E-04 |
| A_24_P274270 | STAT1 | NM_139266 | 1.67 | 3.88E-04 |
| A_23_P310274 | PRSS2 | NM_002770 | 1.67 | 2.07E-04 |
| A_23_P111888 | CTHRC1 | NM_138455 | 1.67 | 5.76E-04 |
| A_24_P331704 | KRT80 | NM_182507 | 1.67 | 3.53E-05 |
| A_24_P333019 | RNF24 | NM_007219 | 1.67 | 1.34E-05 |
| A_23_P111804 | PARP12 | NM_022750 | 1.68 | 3.23E-04 |
| A_24_P79054 | TGFB1 | NM_000660 | 1.68 | 8.61E-04 |
| A_23_P121064 | PTX3 | NM_002852 | 1.68 | 3.76E-04 |
| A_21_P0005546 | XLOC_006661 | TCONS_00014014 | 1.68 | 9.05E-04 |
| A_23_P156327 | TGFBI | NM_000358 | 1.68 | 5.57E-04 |
| A_33_P3306163 | LGALS3 | NM_001177388 | 1.68 | 1.87E-04 |
| A_24_P79054 | TGFB1 | NM_000660 | 1.68 | 1.90E-04 |
| A_23_P381017 | WBSCR27 | NM_152559 | 1.68 | 4.10E-04 |
| A_21_P0009495 | XLOC_012709 | THC2659942 | 1.68 | 8.99E-04 |
| A_23_P101013 | TMC6 | NM_007267 | 1.68 | 9.17E-04 |
| A_24_P79054 | TGFB1 | NM_000660 | 1.68 | 3.76E-04 |
| A_23_P214330 | SERPINB1 | NM_030666 | 1.68 | 4.87E-04 |
| A_23_P127475 | CCS | NM_005125 | 1.68 | 7.12E-06 |
| A_21_P0007222 | XLOC_009181 | ENST00000535746 | 1.68 | 1.92E-04 |
| A_24_P49533 | ZYG11B | NM_024646 | 1.68 | 1.31E-05 |
| A_23_P120056 | RTKN | NM_033046 | 1.68 | 4.61E-04 |
| A_33_P3423830 | FAM115A | NM_014719 | 1.69 | 3.22E-04 |
| A_23_P1998 | APBB1 | NM_001164 | 1.69 | 1.18E-04 |
| A_24_P104119 | RHOF | NM_019034 | 1.69 | 3.83E-04 |
| A_23_P207400 | BRCA1 | NM_007300 | 1.69 | 3.01E-04 |
| A_23_P318904 | SERTAD4 | NM_019605 | 1.69 | 4.45E-04 |
| A_23_P111888 | CTHRC1 | NM_138455 | 1.69 | 7.65E-04 |
| A_23_P79302 | LYPD6B | NM_177964 | 1.69 | 4.19E-04 |
| A_23_P73429 | HCLS1 | NM_005335 | 1.69 | 1.66E-04 |
| A_21_P0001244 | XLOC_001265 | ENST00000451766 | 1.69 | 5.67E-04 |
| A_23_P102965 | BCL2L13 | NM_015367 | 1.69 | 2.30E-06 |
| A_23_P12199 | FAM46B | NM_052943 | 1.69 | 2.19E-04 |
| A_23_P90419 | PBX4 | NM_025245 | 1.69 | 5.21E-04 |
| A_23_P23748 | WDR47 | NM_014969 | 1.69 | 1.54E-04 |
| A_19_P00318495 | LINC00467 | NR_026761 | 1.69 | 5.06E-04 |
| A_23_P102965 | BCL2L13 | NM_015367 | 1.69 | 7.72E-05 |
| A_23_P49279 | C16orf87 | NM_001001436 | 1.69 | 1.89E-05 |
| A_23_P351215 | SKIL | NM_005414 | 1.69 | 1.55E-04 |
| A_23_P121885 | ROPN1L | NM_031916 | 1.70 | 1.63E-05 |
| A_23_P133438 | FAM105A | NM_019018 | 1.70 | 4.88E-04 |
| A_23_P316960 | GRINA | NM_000837 | 1.70 | 2.83E-04 |
| A_23_P101013 | TMC6 | NM_007267 | 1.70 | 5.90E-04 |
| A_23_P121885 | ROPN1L | NM_031916 | 1.70 | 2.11E-04 |
| A_23_P68628 | NECAB3 | NM_031232 | 1.70 | 3.53E-05 |
| A_23_P347169 | MTUS1 | NM_001001924 | 1.70 | 9.82E-04 |
| A_23_P89249 | ERBB2 | NM_001005862 | 1.70 | 2.11E-04 |
| A_24_P160969 | TP53I11 | NM_001076787 | 1.70 | 4.02E-04 |
| A_33_P3393341 | LPIN3 | NM_022896 | 1.70 | 7.57E-04 |
| A_23_P122662 | GFOD1 | NM_018988 | 1.70 | 3.69E-04 |
| A_33_P3242543 | MAOA | NM_000240 | 1.70 | 2.45E-04 |
| A_23_P106761 | CORO1A | NM_007074 | 1.70 | 8.18E-04 |
| A_21_P0011386 | LOC100505679 | NM_001243531 | 1.70 | 8.57E-05 |
| A_23_P122662 | GFOD1 | NM_018988 | 1.70 | 3.17E-04 |
| A_24_P159227 | PAK6 | NM_020168 | 1.71 | 4.72E-05 |
| A_23_P121885 | ROPN1L | NM_031916 | 1.71 | 8.13E-04 |
| A_23_P101380 | B3GNT8 | NM_198540 | 1.71 | 6.90E-04 |
| A_33_P3380086 | PXN | ENST00000323871 | 1.71 | 3.57E-04 |
| A_32_P68050 | NEK1 | NM_012224 | 1.71 | 6.36E-05 |
| A_23_P207400 | BRCA1 | NM_007300 | 1.71 | 9.86E-05 |
| A_23_P129246 | PLEKHO2 | NM_025201 | 1.71 | 2.46E-04 |
| A_24_P274270 | STAT1 | NM_139266 | 1.71 | 9.85E-05 |
| A_23_P141180 | TOM1L2 | NM_001082968 | 1.71 | 2.21E-05 |
| A_23_P39076 | RRAS | NM_006270 | 1.71 | 7.82E-04 |
| A_23_P41854 | CARD6 | NM_032587 | 1.71 | 8.38E-04 |
| A_19_P00322339 | LOC100507127 | NR_038291 | 1.71 | 1.61E-04 |
| A_33_P3373459 | CHST12 | NM_018641 | 1.71 | 1.15E-04 |
| A_24_P145629 | SERINC2 | NM_178865 | 1.71 | 1.34E-04 |
| A_19_P00803151 | LOC100506119 | ENST00000436710 | 1.71 | 2.76E-04 |
| A_33_P3378835 | SLC9A3R1 | NM_004252 | 1.71 | 3.40E-04 |
| A_23_P19291 | TUBB2A | NM_001069 | 1.71 | 5.08E-04 |
| A_33_P3326210 | ESCO2 | NM_001017420 | 1.71 | 2.34E-06 |
| A_24_P192262 | RALA | NM_005402 | 1.71 | 5.17E-04 |
| A_33_P3263232 | LRRC3 | NM_030891 | 1.72 | 1.28E-04 |
| A_24_P254949 | PGM5 | NM_021965 | 1.72 | 4.21E-04 |
| A_32_P213831 | FAM40B | NM_020704 | 1.72 | 1.01E-04 |
| A_23_P209519 | DNAJB2 | NM_001039550 | 1.72 | 2.17E-04 |
| A_33_P3231750 | ZNF738 | NR_027130 | 1.72 | 3.20E-04 |
| A_21_P0010982 | MALAT1 | NR_002819 | 1.72 | 2.23E-04 |
| A_23_P54649 | TRADD | NM_003789 | 1.72 | 4.30E-04 |
| A_33_P3415551 | GPAT2 | NM_207328 | 1.72 | 1.12E-04 |
| A_24_P348203 | LRRC8E | NM_025061 | 1.72 | 1.91E-06 |
| A_23_P202156 | NFKB2 | NM_001077493 | 1.72 | 9.15E-04 |
| A_23_P133474 | GPX3 | NM_002084 | 1.72 | 3.40E-04 |
| A_33_P3822503 | CTF1 | NM_001330 | 1.72 | 2.89E-04 |
| A_23_P101093 | COPZ2 | NM_016429 | 1.72 | 3.28E-04 |
| A_23_P68240 | GPAT2 | NM_207328 | 1.72 | 1.18E-04 |
| A_21_P0007223 | XLOC_009181 | ENST00000535746 | 1.72 | 9.36E-05 |
| A_23_P23221 | GADD45A | NM_001924 | 1.72 | 8.93E-05 |
| A_23_P27606 | IL27RA | NM_004843 | 1.72 | 5.02E-04 |
| A_23_P113005 | EFNA1 | NM_004428 | 1.73 | 4.54E-05 |
| A_23_P69497 | CLEC3B | NM_003278 | 1.73 | 6.17E-04 |
| A_24_P274270 | STAT1 | NM_139266 | 1.73 | 5.09E-04 |
| A_23_P111804 | PARP12 | NM_022750 | 1.73 | 7.99E-05 |
| A_23_P114929 | BRP44 | NM_015415 | 1.73 | 2.67E-05 |
| A_21_P0005820 | XLOC_007057 | ENST00000519764 | 1.73 | 2.60E-05 |
| A_23_P24129 | DKK1 | NM_012242 | 1.73 | 2.81E-05 |
| A_23_P89249 | ERBB2 | NM_001005862 | 1.73 | 7.63E-05 |
| A_19_P00319404 | LINC00472 | NR_026807 | 1.73 | 9.31E-04 |
| A_23_P111804 | PARP12 | NM_022750 | 1.73 | 9.85E-05 |
| A_21_P0011821 | GPAT2 | NM_207328 | 1.73 | 2.28E-04 |
| A_33_P3379967 | HLA-F | NM_001098478 | 1.73 | 8.69E-04 |
| A_33_P3387621 | RHPN2 | NM_033103 | 1.73 | 1.26E-04 |
| A_23_P102965 | BCL2L13 | NM_015367 | 1.74 | 5.81E-04 |
| A_23_P114929 | BRP44 | NM_015415 | 1.74 | 1.73E-05 |
| A_33_P3368014 | HVCN1 | NM_001040107 | 1.74 | 1.91E-04 |
| A_23_P89249 | ERBB2 | NM_001005862 | 1.74 | 3.22E-04 |
| A_33_P3394380 | AKAP5 | NM_004857 | 1.74 | 2.44E-04 |
| A_23_P69339 | ACAA1 | NM_001607 | 1.74 | 1.15E-04 |
| A_24_P189533 | ENDOD1 | NM_015036 | 1.74 | 5.65E-04 |
| A_23_P77493 | TUBB3 | NM_006086 | 1.74 | 4.25E-04 |
| A_23_P145485 | ULBP2 | NM_025217 | 1.74 | 8.65E-04 |
| A_23_P44244 | SMARCA1 | NM_003069 | 1.74 | 6.97E-05 |
| A_33_P3260575 | CERCAM | NM_016174 | 1.74 | 4.05E-04 |
| A_23_P101013 | TMC6 | NM_007267 | 1.74 | 3.00E-04 |
| A_33_P3281408 | YPEL5 | NM_001127401 | 1.74 | 3.26E-04 |
| A_23_P258221 | ABCC5 | NM_005688 | 1.74 | 3.80E-04 |
| A_23_P15146 | IL32 | NM_001012631 | 1.75 | 5.24E-05 |
| A_21_P0012240 | psiTPTE22 | NR_001591 | 1.75 | 7.33E-04 |
| A_24_P389415 | PNMA2 | NM_007257 | 1.75 | 3.34E-05 |
| A_33_P3369371 | GPX3 | NM_002084 | 1.75 | 9.31E-04 |
| A_19_P00322929 | XLOC_003870 | ENST00000513179 | 1.75 | 7.63E-04 |
| A_23_P156408 | LINC00472 | NR_026807 | 1.75 | 5.47E-04 |
| A_23_P114929 | BRP44 | NM_015415 | 1.75 | 8.21E-05 |
| A_32_P41553 | C1orf182 | NM_144627 | 1.75 | 2.46E-05 |
| A_23_P64661 | ARHGAP9 | NM_032496 | 1.75 | 1.52E-04 |
| A_33_P3367447 | ALDH3B1 | NM_001161473 | 1.75 | 9.23E-04 |
| A_23_P205228 | ATP7B | NM_000053 | 1.75 | 2.38E-04 |
| A_33_P3253832 | FV367791 | FV367791 | 1.75 | 2.44E-04 |
| A_23_P328600 | SLC24A6 | NM_024959 | 1.75 | 2.45E-04 |
| A_23_P102965 | BCL2L13 | NM_015367 | 1.75 | 4.76E-04 |
| A_21_P0010449 | XLOC_014399 | ENST00000422971 | 1.75 | 2.06E-04 |
| A_32_P184488 | PHLDB3 | NM_198850 | 1.75 | 8.73E-04 |
| A_23_P207967 | CTIF | NM_014772 | 1.75 | 4.30E-04 |
| A_23_P2967 | AP1G2 | NM_003917 | 1.75 | 2.01E-05 |
| A_24_P184799 | COCH | NM_004086 | 1.75 | 5.34E-05 |
| A_23_P59397 | RSPH3 | NM_031924 | 1.75 | 2.95E-05 |
| A_19_P00320866 | LOC100506119 | ENST00000436710 | 1.75 | 7.73E-04 |
| A_23_P40611 | TCN2 | NM_000355 | 1.75 | 4.93E-04 |
| A_23_P39465 | BST2 | NM_004335 | 1.75 | 4.88E-04 |
| A_24_P303454 | TIAM2 | NM_012454 | 1.76 | 2.17E-04 |
| A_21_P0008527 | XLOC_010971 | TCONS_00022675 | 1.76 | 3.46E-04 |
| A_23_P121885 | ROPN1L | NM_031916 | 1.76 | 1.26E-04 |
| A_23_P207400 | BRCA1 | NM_007300 | 1.76 | 3.49E-04 |
| A_21_P0001959 | XLOC_001826 | ENST00000430494 | 1.76 | 7.25E-05 |
| A_23_P14124 | RASL11A | NM_206827 | 1.76 | 3.56E-04 |
| A_23_P338519 | NKIRAS1 | NM_020345 | 1.76 | 1.12E-04 |
| A_33_P3284919 | SEMA6C | NM_001178061 | 1.76 | 8.95E-04 |
| A_23_P82503 | PEG10 | NM_001040152 | 1.76 | 2.35E-04 |
| A_23_P114929 | BRP44 | NM_015415 | 1.76 | 6.59E-05 |
| A_23_P163682 | RHBDF1 | NM_022450 | 1.76 | 2.53E-04 |
| A_23_P89249 | ERBB2 | NM_001005862 | 1.76 | 7.37E-05 |
| A_23_P121885 | ROPN1L | NM_031916 | 1.76 | 1.95E-04 |
| A_21_P0008620 | LOC100131089 | NR_040062 | 1.76 | 1.82E-04 |
| A_23_P101013 | TMC6 | NM_007267 | 1.76 | 2.75E-04 |
| A_23_P102965 | BCL2L13 | NM_015367 | 1.76 | 1.05E-04 |
| A_33_P3209491 | TNS1 | NM_022648 | 1.76 | 1.56E-04 |
| A_23_P105012 | HRASLS2 | NM_017878 | 1.76 | 6.73E-04 |
| A_24_P307854 | FBXL18 | NM_024963 | 1.76 | 1.21E-04 |
| A_23_P111804 | PARP12 | NM_022750 | 1.76 | 1.22E-05 |
| A_24_P46953 | SGK3 | NM_013257 | 1.77 | 1.90E-05 |
| A_23_P121885 | ROPN1L | NM_031916 | 1.77 | 8.38E-05 |
| A_23_P101093 | COPZ2 | NM_016429 | 1.77 | 3.27E-04 |
| A_24_P5743 | ALDH16A1 | NM_153329 | 1.77 | 6.79E-05 |
| A_23_P207400 | BRCA1 | NM_007300 | 1.77 | 4.37E-04 |
| A_33_P3376214 | FMO5 | NM_001144829 | 1.77 | 6.72E-04 |
| A_23_P102965 | BCL2L13 | NM_015367 | 1.77 | 6.42E-04 |
| A_23_P89249 | ERBB2 | NM_001005862 | 1.77 | 2.62E-05 |
| A_23_P89249 | ERBB2 | NM_001005862 | 1.77 | 1.37E-05 |
| A_23_P114929 | BRP44 | NM_015415 | 1.77 | 2.99E-05 |
| A_23_P107963 | FUT1 | NM_000148 | 1.77 | 3.96E-04 |
| A_33_P3220723 | KIAA0922 | NM_015196 | 1.77 | 7.62E-04 |
| A_33_P3329023 | FAM69A | NM_001252273 | 1.77 | 9.77E-04 |
| A_23_P210210 | EPAS1 | NM_001430 | 1.77 | 1.53E-04 |
| A_23_P134854 | CLDN23 | NM_194284 | 1.78 | 4.98E-04 |
| A_23_P120883 | HMOX1 | NM_002133 | 1.78 | 9.90E-05 |
| A_23_P154688 | SLC4A11 | NM_032034 | 1.78 | 2.59E-04 |
| A_23_P207400 | BRCA1 | NM_007300 | 1.78 | 5.13E-04 |
| A_33_P3257861 | SARDH | NM_001134707 | 1.78 | 3.35E-04 |
| A_24_P398323 | TRIM34 | NM_001003827 | 1.78 | 9.15E-05 |
| A_23_P12147 | C1orf74 | NM_152485 | 1.78 | 3.37E-04 |
| A_23_P111804 | PARP12 | NM_022750 | 1.78 | 1.31E-04 |
| A_23_P12343 | GSTM3 | NM_000849 | 1.78 | 1.35E-05 |
| A_33_P3243069 | KIAA0040 | NM_001162893 | 1.78 | 2.20E-04 |
| A_33_P3273534 | KRT81 | NM_002281 | 1.78 | 3.37E-04 |
| A_23_P62764 | CCDC28B | NM_024296 | 1.78 | 1.28E-04 |
| A_24_P102981 | DNAJB2 | NM_006736 | 1.78 | 4.13E-04 |
| A_33_P3210671 | GPAT2 | NM_207328 | 1.78 | 7.35E-05 |
| A_23_P106898 | ORAI3 | NM_152288 | 1.78 | 3.41E-04 |
| A_23_P90311 | TICAM1 | NM_182919 | 1.78 | 1.91E-04 |
| A_24_P37253 | LYPD6 | NM_194317 | 1.78 | 7.98E-06 |
| A_23_P89249 | ERBB2 | NM_001005862 | 1.78 | 1.77E-04 |
| A_24_P873659 | MALAT1 | NR_002819 | 1.79 | 6.76E-05 |
| A_23_P84202 | WDR25 | NM_024515 | 1.79 | 8.97E-04 |
| A_33_P3280531 | CRAT | NM_000755 | 1.79 | 2.14E-05 |
| A_23_P39050 | ZNF823 | NM_001080493 | 1.79 | 1.81E-04 |
| A_33_P3372332 | CYBA | NM_000101 | 1.79 | 3.79E-05 |
| A_33_P3348224 | SEC14L6 | NM_001193336 | 1.79 | 3.70E-04 |
| A_23_P121657 | HS3ST1 | NM_005114 | 1.79 | 8.70E-04 |
| A_23_P408167 | GPRASP2 | NM_001004051 | 1.79 | 6.37E-05 |
| A_23_P64567 | PPME1 | NM_016147 | 1.79 | 1.28E-04 |
| A_23_P42087 | BPHL | NM_004332 | 1.79 | 1.56E-04 |
| A_23_P119464 | RHPN2 | NM_033103 | 1.79 | 1.06E-04 |
| A_23_P385206 | STX12 | NM_177424 | 1.79 | 7.01E-04 |
| A_23_P106898 | ORAI3 | NM_152288 | 1.79 | 1.08E-04 |
| A_23_P89249 | ERBB2 | NM_001005862 | 1.80 | 9.42E-06 |
| A_23_P211504 | KDELR3 | NM_016657 | 1.80 | 1.65E-04 |
| A_23_P207400 | BRCA1 | NM_007300 | 1.80 | 8.54E-04 |
| A_23_P171366 | USP11 | NM_004651 | 1.80 | 3.45E-05 |
| A_23_P100315 | DECR2 | NM_020664 | 1.80 | 4.68E-05 |
| A_23_P114929 | BRP44 | NM_015415 | 1.80 | 6.22E-05 |
| A_23_P121885 | ROPN1L | NM_031916 | 1.80 | 1.70E-04 |
| A_24_P148750 | SH3BP5 | NM_004844 | 1.80 | 2.74E-04 |
| A_33_P3220911 | BST2 | ENST00000252593 | 1.80 | 2.36E-04 |
| A_21_P0014231 | HMGA1P4 | XR_108949 | 1.80 | 3.41E-04 |
| A_23_P89249 | ERBB2 | NM_001005862 | 1.80 | 1.26E-05 |
| A_23_P12343 | GSTM3 | NM_000849 | 1.80 | 3.77E-06 |
| A_23_P336198 | GLCCI1 | NM_138426 | 1.80 | 2.69E-04 |
| A_33_P3330549 | SLC44A2 | NM_020428 | 1.80 | 3.30E-04 |
| A_23_P120002 | SP110 | NM_004510 | 1.80 | 6.89E-05 |
| A_23_P121657 | HS3ST1 | NM_005114 | 1.80 | 5.18E-04 |
| A_21_P0011960 | XLOC_l2_008226 | ENST00000449819 | 1.80 | 1.97E-04 |
| A_33_P3410589 | FAM43A | NM_153690 | 1.80 | 6.84E-04 |
| A_33_P3375314 | ATP9A | NM_006045 | 1.80 | 5.24E-04 |
| A_23_P114929 | BRP44 | NM_015415 | 1.80 | 1.33E-05 |
| A_23_P83818 | COL5A1 | NM_000093 | 1.80 | 2.09E-04 |
| A_23_P207400 | BRCA1 | NM_007300 | 1.81 | 5.84E-04 |
| A_23_P89249 | ERBB2 | NM_001005862 | 1.81 | 5.49E-06 |
| A_23_P126212 | CLSPN | NM_022111 | 1.81 | 1.16E-04 |
| A_23_P101093 | COPZ2 | NM_016429 | 1.81 | 1.76E-04 |
| A_24_P37264 | RNF144A | NM_014746 | 1.81 | 3.67E-05 |
| A_23_P111804 | PARP12 | NM_022750 | 1.81 | 2.19E-05 |
| A_23_P107963 | FUT1 | NM_000148 | 1.81 | 1.05E-04 |
| A_23_P12343 | GSTM3 | NM_000849 | 1.81 | 4.06E-05 |
| A_23_P121657 | HS3ST1 | NM_005114 | 1.81 | 6.23E-04 |
| A_24_P734953 | TRNP1 | NM_001013642 | 1.81 | 5.39E-04 |
| A_21_P0010980 | MALAT1 | NR_002819 | 1.81 | 1.34E-04 |
| A_23_P417415 | ACOT11 | NM_147161 | 1.82 | 1.20E-04 |
| A_23_P114929 | BRP44 | NM_015415 | 1.82 | 1.37E-07 |
| A_23_P109427 | GSTT2 | NM_000854 | 1.82 | 3.78E-04 |
| A_23_P406448 | RAB9B | NM_016370 | 1.82 | 2.60E-04 |
| A_24_P44462 | TPM1 | NM_000366 | 1.82 | 2.35E-04 |
| A_23_P152782 | IFI35 | NM_005533 | 1.82 | 1.79E-04 |
| A_23_P101093 | COPZ2 | NM_016429 | 1.82 | 6.24E-05 |
| A_23_P107963 | FUT1 | NM_000148 | 1.82 | 5.57E-04 |
| A_23_P30243 | ERAP2 | NM_022350 | 1.82 | 1.97E-05 |
| A_23_P213336 | FGF1 | NM_000800 | 1.82 | 3.20E-04 |
| A_24_P261567 | GDPD5 | NM_030792 | 1.82 | 2.11E-04 |
| A_33_P3404531 | ZCWPW1 | NM_017984 | 1.82 | 2.92E-04 |
| A_23_P27649 | ZNF433 | NM_001080411 | 1.82 | 3.43E-05 |
| A_23_P111804 | PARP12 | NM_022750 | 1.82 | 6.10E-05 |
| A_23_P111804 | PARP12 | NM_022750 | 1.82 | 1.25E-04 |
| A_24_P75917 | CCDC144A | NM_014695 | 1.83 | 1.56E-04 |
| A_24_P35400 | SARDH | NM_007101 | 1.83 | 2.98E-04 |
| A_23_P151166 | HVCN1 | NM_001040107 | 1.83 | 2.44E-04 |
| A_21_P0014948 | LOC100510710 | ENST00000313929 | 1.83 | 5.19E-05 |
| A_23_P114929 | BRP44 | NM_015415 | 1.83 | 1.92E-05 |
| A_23_P76102 | GDF11 | NM_005811 | 1.83 | 4.64E-04 |
| A_24_P269814 | PLEKHA1 | NM_001001974 | 1.83 | 5.33E-04 |
| A_23_P154208 | NAGK | NM_017567 | 1.83 | 7.01E-04 |
| A_23_P107963 | FUT1 | NM_000148 | 1.83 | 8.71E-05 |
| A_23_P392222 | SWI5 | NM_001040011 | 1.83 | 1.08E-05 |
| A_32_P512061 | GBAP1 | NR_002188 | 1.83 | 8.99E-04 |
| A_23_P121885 | ROPN1L | NM_031916 | 1.83 | 7.85E-05 |
| A_33_P3266674 | ZBTB46 | NM_025224 | 1.83 | 5.18E-05 |
| A_33_P3404189 | HEATR7A | NM_032450 | 1.83 | 7.38E-04 |
| A_23_P38864 | RABAC1 | NM_006423 | 1.83 | 8.95E-06 |
| A_23_P101093 | COPZ2 | NM_016429 | 1.83 | 2.73E-06 |
| A_23_P114929 | BRP44 | NM_015415 | 1.83 | 2.85E-05 |
| A_23_P14184 | THSD1 | NM_018676 | 1.84 | 6.67E-04 |
| A_23_P111888 | CTHRC1 | NM_138455 | 1.84 | 3.25E-04 |
| A_33_P3418716 | EFHD1 | NM_025202 | 1.84 | 1.52E-04 |
| A_32_P129894 | MEGF9 | NM_001080497 | 1.84 | 7.82E-04 |
| A_21_P0000827 | LOC100506428 | NR_038894 | 1.84 | 3.59E-05 |
| A_23_P111804 | PARP12 | NM_022750 | 1.84 | 8.28E-05 |
| A_23_P164912 | LIN7B | NM_022165 | 1.84 | 4.05E-04 |
| A_21_P0006579 | XLOC_008151 | BC015977 | 1.84 | 9.90E-04 |
| A_24_P90216 | LGR4 | NM_018490 | 1.84 | 8.59E-04 |
| A_24_P247978 | ZNF589 | NM_016089 | 1.84 | 1.05E-04 |
| A_23_P107963 | FUT1 | NM_000148 | 1.85 | 7.31E-04 |
| A_24_P147407 | STRADA | NM_153335 | 1.85 | 4.21E-04 |
| A_33_P3530314 | C5orf42 | NM_023073 | 1.85 | 1.61E-04 |
| A_23_P102965 | BCL2L13 | NM_015367 | 1.85 | 5.98E-05 |
| A_19_P00324839 | MALAT1 | NR_002819 | 1.85 | 9.82E-05 |
| A_23_P211598 | PMM1 | NM_002676 | 1.85 | 5.11E-05 |
| A_23_P121885 | ROPN1L | NM_031916 | 1.85 | 5.31E-05 |
| A_23_P12343 | GSTM3 | NM_000849 | 1.85 | 3.43E-06 |
| A_23_P12343 | GSTM3 | NM_000849 | 1.85 | 3.03E-06 |
| A_23_P12343 | GSTM3 | NM_000849 | 1.85 | 3.86E-05 |
| A_23_P12343 | GSTM3 | NM_000849 | 1.86 | 5.16E-06 |
| A_23_P48997 | PSTPIP1 | NM_003978 | 1.86 | 8.92E-04 |
| A_23_P11598 | ZNF684 | NM_152373 | 1.86 | 3.40E-05 |
| A_23_P98631 | HPS5 | NM_181507 | 1.86 | 3.78E-05 |
| A_23_P251647 | RFESD | NM_173362 | 1.86 | 4.13E-04 |
| A_23_P101380 | B3GNT8 | NM_198540 | 1.86 | 7.72E-05 |
| A_32_P420009 | ALS2CL | NM_147129 | 1.86 | 1.20E-05 |
| A_23_P12343 | GSTM3 | NM_000849 | 1.86 | 2.85E-06 |
| A_23_P106103 | AKAP5 | NM_004857 | 1.86 | 5.34E-04 |
| A_21_P0011435 | RHPN2 | NM_033103 | 1.86 | 2.99E-04 |
| A_23_P121657 | HS3ST1 | NM_005114 | 1.86 | 5.28E-04 |
| A_23_P94552 | TMEM2 | NM_013390 | 1.87 | 3.74E-04 |
| A_23_P102965 | BCL2L13 | NM_015367 | 1.87 | 3.33E-04 |
| A_33_P3335966 | TPM1 | NM_001018005 | 1.87 | 8.66E-05 |
| A_23_P101093 | COPZ2 | NM_016429 | 1.87 | 7.65E-04 |
| A_24_P10657 | SLC44A2 | NM_020428 | 1.87 | 6.57E-06 |
| A_23_P145 | HMGCL | NM_000191 | 1.87 | 1.11E-04 |
| A_23_P12343 | GSTM3 | NM_000849 | 1.87 | 1.43E-06 |
| A_23_P106103 | AKAP5 | NM_004857 | 1.87 | 6.77E-04 |
| A_23_P107963 | FUT1 | NM_000148 | 1.87 | 1.43E-04 |
| A_24_P307869 | LLGL2 | NM_001015002 | 1.87 | 4.53E-04 |
| A_32_P18440 | ARID5B | NM_032199 | 1.87 | 5.11E-04 |
| A_23_P109072 | SALL4 | NM_020436 | 1.87 | 1.81E-04 |
| A_23_P121657 | HS3ST1 | NM_005114 | 1.87 | 2.41E-05 |
| A_21_P0006829 | LOC100507127 | NR_038291 | 1.88 | 8.86E-04 |
| A_24_P406060 | RNF144B | NM_182757 | 1.88 | 1.51E-04 |
| A_23_P10232 | BANK1 | NM_017935 | 1.88 | 9.28E-04 |
| A_23_P121657 | HS3ST1 | NM_005114 | 1.88 | 2.03E-04 |
| A_23_P53891 | KLF5 | NM_001730 | 1.88 | 4.09E-05 |
| A_33_P3289045 | GSTT2B | NM_001080843 | 1.88 | 6.50E-04 |
| A_21_P0013686 | XLOC_l2_015360 | ENST00000449730 | 1.88 | 4.17E-04 |
| A_33_P3414907 | NPL | NM_001200050 | 1.88 | 3.46E-04 |
| A_23_P1043 | C1orf106 | NM_018265 | 1.88 | 7.65E-04 |
| A_24_P353638 | SLAMF7 | NM_021181 | 1.88 | 4.20E-04 |
| A_23_P325924 | FAM59B | NM_001191033 | 1.88 | 3.51E-04 |
| A_23_P321511 | Mar3 | NM_178450 | 1.88 | 7.60E-05 |
| A_23_P92230 | TSC22D2 | NM_014779 | 1.88 | 3.98E-05 |
| A_24_P349039 | ARHGAP31 | NM_020754 | 1.88 | 2.08E-04 |
| A_33_P3388618 | TNK1 | NM_001251902 | 1.89 | 2.88E-04 |
| A_19_P00803606 | Q21C94 | AK002196 | 1.89 | 1.51E-04 |
| A_23_P100501 | HMOX2 | NM_002134 | 1.89 | 3.87E-04 |
| A_33_P3268555 | SP140 | NM_001005176 | 1.89 | 6.30E-04 |
| A_24_P218688 | ALDH3B1 | NM_000694 | 1.89 | 6.11E-05 |
| A_23_P101093 | COPZ2 | NM_016429 | 1.89 | 5.60E-05 |
| A_23_P101380 | B3GNT8 | NM_198540 | 1.89 | 1.65E-04 |
| A_23_P45011 | PPP1R14C | NM_030949 | 1.89 | 5.59E-04 |
| A_23_P47116 | RASSF7 | NM_003475 | 1.89 | 2.38E-05 |
| A_23_P55011 | SLC38A10 | NM_001037984 | 1.89 | 1.12E-04 |
| A_23_P10025 | NELL2 | NM_006159 | 1.89 | 3.83E-04 |
| A_23_P56228 | GMIP | NM_016573 | 1.89 | 7.76E-04 |
| A_23_P106103 | AKAP5 | NM_004857 | 1.89 | 9.87E-04 |
| A_32_P175301 | DENND3 | NM_014957 | 1.89 | 5.60E-05 |
| A_33_P3349702 | LOC400927 | NR_002821 | 1.89 | 9.31E-04 |
| A_33_P3318288 | CFH | NM_001014975 | 1.89 | 5.12E-05 |
| A_23_P133648 | FAM8A1 | NM_016255 | 1.90 | 2.10E-04 |
| A_33_P3227920 | SLC16A4 | NM_004696 | 1.90 | 8.69E-05 |
| A_23_P137470 | SIPA1L2 | NM_020808 | 1.90 | 6.12E-04 |
| A_23_P26649 | NMRAL1 | NM_020677 | 1.90 | 1.61E-05 |
| A_23_P422718 | POLH | NM_006502 | 1.90 | 1.55E-04 |
| A_24_P842006 | C16orf93 | NM_001014979 | 1.90 | 1.26E-04 |
| A_21_P0001469 | XLOC_000711 | TCONS_00001393 | 1.90 | 5.90E-04 |
| A_33_P3380944 | GPR116 | NM_001098518 | 1.90 | 2.01E-04 |
| A_23_P137097 | SLC16A2 | NM_006517 | 1.90 | 1.98E-04 |
| A_23_P9662 | IPP | NM_005897 | 1.90 | 1.57E-04 |
| A_33_P3324884 | MICAL1 | NM_022765 | 1.90 | 6.93E-04 |
| A_32_P129752 | TMEM30B | NM_001017970 | 1.90 | 6.22E-04 |
| A_32_P3572 | ENST00000449075 | ENST00000449075 | 1.90 | 2.70E-04 |
| A_23_P106898 | ORAI3 | NM_152288 | 1.90 | 9.22E-04 |
| A_23_P102965 | BCL2L13 | NM_015367 | 1.90 | 2.79E-04 |
| A_32_P70315 | TIMP4 | NM_003256 | 1.90 | 6.32E-04 |
| A_23_P121657 | HS3ST1 | NM_005114 | 1.90 | 1.32E-04 |
| A_33_P3367692 | CFH | NM_001014975 | 1.90 | 2.98E-05 |
| A_33_P3369436 | LOC100130111 | XR_109206 | 1.91 | 6.06E-04 |
| A_23_P101093 | COPZ2 | NM_016429 | 1.91 | 1.18E-04 |
| A_23_P100711 | PMP22 | NM_000304 | 1.91 | 1.89E-04 |
| A_23_P12343 | GSTM3 | NM_000849 | 1.91 | 9.84E-06 |
| A_23_P101093 | COPZ2 | NM_016429 | 1.91 | 9.25E-04 |
| A_23_P103256 | CFHR3 | NM_021023 | 1.91 | 9.32E-04 |
| A_23_P102965 | BCL2L13 | NM_015367 | 1.91 | 2.76E-04 |
| A_33_P3682006 | ENST00000425189 | ENST00000425189 | 1.91 | 8.47E-04 |
| A_23_P100711 | PMP22 | NM_000304 | 1.91 | 5.77E-04 |
| A_33_P3336925 | PDLIM5 | NM_001011516 | 1.91 | 1.66E-04 |
| A_33_P3385266 | ABCC6 | NM_001079528 | 1.91 | 3.35E-04 |
| A_33_P3344618 | TFEB | NM_007162 | 1.91 | 4.01E-04 |
| A_23_P33914 | NUP62CL | NM_017681 | 1.91 | 5.02E-04 |
| A_33_P3421243 | AFP | NM_001134 | 1.91 | 1.18E-04 |
| A_33_P3339860 | PLEKHA2 | NM_021623 | 1.91 | 4.74E-05 |
| A_23_P150281 | TP53I11 | NM_001076787 | 1.92 | 4.01E-04 |
| A_33_P3243554 | Sep6 | NM_145802 | 1.92 | 1.29E-04 |
| A_23_P205389 | MOAP1 | NM_022151 | 1.92 | 4.45E-04 |
| A_33_P3407937 | PLCXD1 | NM_018390 | 1.92 | 9.42E-04 |
| A_23_P100711 | PMP22 | NM_000304 | 1.92 | 2.44E-04 |
| A_23_P121499 | WFS1 | NM_006005 | 1.92 | 2.10E-04 |
| A_33_P3264895 | RHEBL1 | NM_144593 | 1.92 | 9.67E-04 |
| A_23_P65930 | ZFYVE19 | NM_001077268 | 1.92 | 9.25E-05 |
| A_33_P3216714 | DNAJC6 | NM_014787 | 1.92 | 1.39E-04 |
| A_33_P3363620 | TMEM91 | NM_001098824 | 1.92 | 3.95E-04 |
| A_23_P167599 | FAM134B | NM_001034850 | 1.92 | 3.47E-04 |
| A_23_P97064 | FBXO6 | NM_018438 | 1.92 | 4.91E-04 |
| A_33_P3237775 | NR1H3 | NM_005693 | 1.92 | 1.21E-04 |
| A_23_P107963 | FUT1 | NM_000148 | 1.92 | 2.61E-04 |
| A_21_P0011344 | RHPN2 | NM_033103 | 1.92 | 2.03E-04 |
| A_23_P10025 | NELL2 | NM_006159 | 1.92 | 2.72E-04 |
| A_32_P110390 | TMEM171 | NM_173490 | 1.92 | 3.29E-04 |
| A_33_P3305173 | RGAG4 | NM_001024455 | 1.93 | 3.21E-04 |
| A_24_P4705 | PPME1 | NM_016147 | 1.93 | 5.75E-04 |
| A_23_P11598 | ZNF684 | NM_152373 | 1.93 | 1.28E-04 |
| A_33_P3414683 | KIFC2 | NM_145754 | 1.93 | 1.23E-04 |
| A_33_P3333054 | SYBU | NM_001099744 | 1.93 | 1.56E-04 |
| A_23_P162589 | VDR | NM_001017535 | 1.93 | 5.79E-04 |
| A_24_P940166 | PAPSS2 | NM_001015880 | 1.93 | 4.74E-05 |
| A_23_P1043 | C1orf106 | NM_018265 | 1.93 | 2.83E-04 |
| A_23_P162171 | MCAM | NM_006500 | 1.93 | 2.18E-04 |
| A_23_P48561 | EFS | NM_005864 | 1.93 | 2.47E-04 |
| A_23_P153461 | LPPR2 | NM_022737 | 1.94 | 8.74E-04 |
| A_24_P116535 | MMP15 | NM_002428 | 1.94 | 2.13E-04 |
| A_33_P3554318 | TRIM3 | NM_006458 | 1.94 | 5.13E-05 |
| A_23_P34597 | CDA | NM_001785 | 1.94 | 2.10E-04 |
| A_21_P0011068 | LOC100506691 | XR_109143 | 1.94 | 5.09E-05 |
| A_23_P100711 | PMP22 | NM_000304 | 1.94 | 3.46E-04 |
| A_21_P0007189 | XLOC_009092 | TCONS_00019262 | 1.94 | 4.36E-06 |
| A_23_P105794 | EPSTI1 | NM_033255 | 1.94 | 4.47E-04 |
| A_23_P10025 | NELL2 | NM_006159 | 1.94 | 6.69E-04 |
| A_24_P319364 | F11R | NM_016946 | 1.95 | 8.05E-05 |
| A_23_P209944 | RETSAT | NM_017750 | 1.95 | 7.74E-05 |
| A_33_P3352019 | SCARA3 | NM_182826 | 1.95 | 1.51E-04 |
| A_23_P2814 | SMAD9 | NM_005905 | 1.95 | 3.97E-05 |
| A_23_P100711 | PMP22 | NM_000304 | 1.95 | 1.26E-04 |
| A_23_P77328 | GCHFR | NM_005258 | 1.95 | 2.34E-04 |
| A_24_P364057 | C11orf70 | NM_032930 | 1.95 | 3.71E-04 |
| A_23_P319617 | CHST7 | NM_019886 | 1.95 | 8.18E-05 |
| A_23_P106103 | AKAP5 | NM_004857 | 1.95 | 2.99E-04 |
| A_33_P3238250 | F11R | NM_016946 | 1.96 | 3.27E-04 |
| A_23_P119943 | IGFBP2 | NM_000597 | 1.96 | 3.20E-04 |
| A_23_P207399 | NBR1 | NM_031858 | 1.96 | 5.08E-05 |
| A_23_P100711 | PMP22 | NM_000304 | 1.96 | 2.19E-04 |
| A_33_P3406072 | FRMD3 | NM_001244959 | 1.96 | 3.01E-04 |
| A_33_P3365134 | PPP1R13B | NM_015316 | 1.96 | 1.40E-04 |
| A_23_P116235 | MDK | NM_001012334 | 1.96 | 2.28E-04 |
| A_23_P101380 | B3GNT8 | NM_198540 | 1.96 | 8.42E-04 |
| A_23_P106898 | ORAI3 | NM_152288 | 1.97 | 4.22E-04 |
| A_23_P100711 | PMP22 | NM_000304 | 1.97 | 3.32E-04 |
| A_23_P206018 | TPM1 | NM_001018004 | 1.97 | 3.53E-05 |
| A_23_P10025 | NELL2 | NM_006159 | 1.97 | 1.31E-04 |
| A_23_P96369 | CXorf57 | NM_018015 | 1.97 | 3.66E-04 |
| A_23_P398294 | HIP1R | NM_003959 | 1.97 | 2.05E-04 |
| A_23_P393620 | TFPI2 | NM_006528 | 1.97 | 1.72E-05 |
| A_23_P15272 | ABCC6 | NM_001079528 | 1.97 | 1.13E-04 |
| A_23_P101380 | B3GNT8 | NM_198540 | 1.98 | 3.54E-04 |
| A_23_P100711 | PMP22 | NM_000304 | 1.98 | 3.78E-04 |
| A_23_P119943 | IGFBP2 | NM_000597 | 1.98 | 2.41E-04 |
| A_23_P48747 | DHRS1 | NM_138452 | 1.98 | 1.18E-05 |
| A_23_P162589 | VDR | NM_001017535 | 1.98 | 1.59E-04 |
| A_21_P0001636 | XLOC_001230 | ENST00000433058 | 1.98 | 3.63E-04 |
| A_23_P80739 | PLCD1 | NM_006225 | 1.98 | 2.32E-05 |
| A_33_P3285565 | CLDN3 | NM_001306 | 1.98 | 5.00E-04 |
| A_33_P3728698 | FLJ45248 | AK127183 | 1.98 | 6.64E-05 |
| A_23_P417942 | FNBP1L | NM_001024948 | 1.98 | 7.71E-04 |
| A_23_P125423 | C1R | NM_001733 | 1.98 | 1.24E-04 |
| A_23_P100711 | PMP22 | NM_000304 | 1.98 | 2.30E-04 |
| A_24_P47988 | ELL3 | NM_025165 | 1.99 | 2.14E-04 |
| A_23_P106835 | BBS2 | NM_031885 | 1.99 | 7.22E-04 |
| A_23_P10025 | NELL2 | NM_006159 | 1.99 | 1.06E-04 |
| A_23_P10025 | NELL2 | NM_006159 | 1.99 | 3.85E-04 |
| A_33_P3350748 | KRT7 | NM_005556 | 1.99 | 6.95E-06 |
| A_33_P3351175 | WNK2 | NM_006648 | 1.99 | 2.12E-04 |
| A_32_P47988 | CAMK2D | NM_001221 | 1.99 | 5.74E-04 |
| A_23_P100711 | PMP22 | NM_000304 | 2.00 | 1.82E-04 |
| A_23_P119943 | IGFBP2 | NM_000597 | 2.00 | 2.19E-04 |
| A_32_P166693 | HEG1 | NM_020733 | 2.00 | 2.14E-04 |
| A_23_P119943 | IGFBP2 | NM_000597 | 2.00 | 3.34E-05 |
| A_23_P41470 | DDX60 | NM_017631 | 2.00 | 9.94E-04 |
| A_33_P3843285 | ENST00000460754 | ENST00000460754 | 2.00 | 4.85E-04 |
| A_23_P44724 | CSRP2 | NM_001321 | 2.00 | 2.45E-04 |
| A_33_P3376365 | HES2 | ENST00000377836 | 2.00 | 1.21E-04 |
| A_23_P10025 | NELL2 | NM_006159 | 2.00 | 3.94E-05 |
| A_24_P42066 | GGT3P | NR_003267 | 2.00 | 2.04E-04 |
| A_33_P3277447 | SLC26A2 | NM_000112 | 2.00 | 1.92E-05 |
| A_23_P66117 | ITFG3 | NM_032039 | 2.00 | 7.27E-05 |
| A_23_P35414 | PPP1R3C | NM_005398 | 2.00 | 1.45E-04 |
| A_33_P3213064 | STAT2 | NM_005419 | 2.00 | 5.65E-04 |
| A_23_P30315 | TRIM7 | NM_033342 | 2.00 | 4.80E-04 |
| A_23_P128817 | PCK2 | NM_004563 | 2.00 | 3.38E-04 |
| A_23_P218505 | LHB | NM_000894 | 2.00 | 8.15E-05 |
| A_32_P21255 | SLC30A4 | NM_013309 | 2.00 | 8.53E-04 |
| A_21_P0005617 | FLJ45248 | AK127183 | 2.01 | 2.28E-06 |
| A_21_P0012252 | XLOC_l2_009311 | TCONS_l2_00017628 | 2.01 | 2.24E-04 |
| A_23_P100539 | ABCC6 | NM_001171 | 2.01 | 3.02E-04 |
| A_23_P116280 | WT1 | NM_024426 | 2.01 | 8.98E-04 |
| A_23_P132910 | RBM47 | NM_019027 | 2.01 | 4.54E-05 |
| A_23_P408473 | HEATR7A | NM_032450 | 2.01 | 4.18E-04 |
| A_24_P246351 | FAM71E1 | NM_138411 | 2.01 | 1.02E-04 |
| A_32_P54274 | DRD5 | NM_000798 | 2.01 | 9.10E-05 |
| A_33_P3249072 | HOGA1 | ENST00000370642 | 2.01 | 6.20E-04 |
| A_23_P21495 | FCGBP | NM_003890 | 2.02 | 5.52E-04 |
| A_23_P119943 | IGFBP2 | NM_000597 | 2.02 | 3.19E-04 |
| A_23_P101380 | B3GNT8 | NM_198540 | 2.02 | 6.44E-04 |
| A_19_P00801752 | XLOC_014103 | THC2718861 | 2.02 | 2.30E-04 |
| A_23_P205567 | PRKCH | NM_006255 | 2.02 | 6.92E-06 |
| A_21_P0006537 | XLOC_008183 | ENST00000439926 | 2.02 | 2.88E-04 |
| A_23_P103511 | C1orf226 | NM_001085375 | 2.02 | 4.34E-04 |
| A_33_P3238335 | MCOLN3 | ENST00000370587 | 2.02 | 8.07E-04 |
| A_23_P119943 | IGFBP2 | NM_000597 | 2.02 | 2.68E-04 |
| A_23_P128598 | TUBA3C | NM_006001 | 2.02 | 2.25E-05 |
| A_32_P407245 | DNAJC22 | NM_024902 | 2.02 | 1.14E-05 |
| A_21_P0006404 | XLOC_007966 | AK127697 | 2.02 | 5.15E-05 |
| A_23_P430658 | HEYL | NM_014571 | 2.03 | 9.70E-05 |
| A_24_P655849 | SMAD9 | NM_001127217 | 2.03 | 4.27E-04 |
| A_23_P162579 | HSPB8 | NM_014365 | 2.03 | 5.51E-04 |
| A_33_P3404651 | TTC7A | NM_020458 | 2.03 | 6.82E-05 |
| A_24_P787889 | TMEM191B | NM_001242313 | 2.03 | 1.19E-04 |
| A_21_P0012456 | XLOC_l2_010330 | TCONS_l2_00019488 | 2.03 | 5.43E-05 |
| A_21_P0006725 | LOC100652988 | XR_132573 | 2.03 | 6.34E-04 |
| A_33_P3398862 | RHOB | NM_004040 | 2.03 | 3.64E-04 |
| A_24_P119545 | ITPKB | ENST00000366784 | 2.03 | 1.78E-04 |
| A_23_P110005 | NICN1 | NM_032316 | 2.03 | 3.62E-04 |
| A_33_P3265749 | PTGER3 | NM_198717 | 2.03 | 4.11E-04 |
| A_23_P119943 | IGFBP2 | NM_000597 | 2.03 | 3.14E-04 |
| A_23_P14515 | ACOT4 | NM_152331 | 2.03 | 2.16E-04 |
| A_23_P101380 | B3GNT8 | NM_198540 | 2.03 | 4.70E-04 |
| A_21_P0000996 | LOC100505940 | XR_110477 | 2.04 | 4.56E-04 |
| A_23_P103256 | CFHR3 | NM_021023 | 2.04 | 7.86E-04 |
| A_33_P3303464 | C19orf39 | NM_175871 | 2.04 | 9.39E-04 |
| A_33_P3283669 | ATP1A3 | NM_152296 | 2.04 | 1.01E-05 |
| A_33_P3420466 | MATN3 | NM_002381 | 2.04 | 1.41E-04 |
| A_23_P129209 | IDH2 | NM_002168 | 2.04 | 6.20E-05 |
| A_23_P119943 | IGFBP2 | NM_000597 | 2.04 | 1.18E-04 |
| A_24_P126139 | RAB9B | NM_016370 | 2.04 | 6.48E-04 |
| A_23_P75786 | SLC15A3 | NM_016582 | 2.04 | 8.82E-05 |
| A_23_P103256 | CFHR3 | NM_021023 | 2.04 | 4.48E-04 |
| A_23_P426472 | ZNF45 | NM_003425 | 2.04 | 2.51E-04 |
| A_21_P0014465 | LOC100507286 | XR_109951 | 2.05 | 4.38E-04 |
| A_23_P106835 | BBS2 | NM_031885 | 2.05 | 5.40E-04 |
| A_33_P3223592 | APOE | NM_000041 | 2.05 | 3.42E-04 |
| A_21_P0010536 | GBP3 | NM_018284 | 2.05 | 4.90E-04 |
| A_33_P3362088 | P2RX4 | NM_002560 | 2.05 | 5.16E-04 |
| A_23_P61180 | PLCXD1 | NM_018390 | 2.05 | 4.10E-04 |
| A_33_P3382629 | ENST00000477589 | ENST00000477589 | 2.05 | 3.11E-04 |
| A_33_P3423365 | GSN | NM_001127663 | 2.05 | 7.54E-04 |
| A_23_P103511 | C1orf226 | NM_001085375 | 2.05 | 8.63E-04 |
| A_23_P142447 | MYO1F | NM_012335 | 2.05 | 8.02E-05 |
| A_19_P00315506 | KIAA0040 | NM_001162893 | 2.05 | 5.81E-04 |
| A_21_P0013563 | XLOC_l2_014771 | DN914066 | 2.05 | 1.78E-04 |
| A_23_P27983 | APLP1 | NM_005166 | 2.06 | 1.66E-04 |
| A_21_P0011305 | LOC100130111 | XR_109206 | 2.06 | 4.06E-05 |
| A_33_P3227472 | SDSL | NM_138432 | 2.06 | 5.98E-05 |
| A_33_P3287825 | CCDC136 | NM_022742 | 2.06 | 7.69E-04 |
| A_23_P106835 | BBS2 | NM_031885 | 2.06 | 2.40E-04 |
| A_19_P00811178 | MGC16121 | NR_024607 | 2.06 | 1.36E-04 |
| A_33_P3405424 | IL4I1 | NM_172374 | 2.06 | 3.06E-04 |
| A_23_P133133 | ALPK1 | NM_025144 | 2.06 | 2.76E-04 |
| A_23_P150741 | C2CD3 | ENST00000442398 | 2.06 | 7.33E-06 |
| A_21_P0006970 | SFTA1P | NR_027082 | 2.07 | 5.02E-05 |
| A_23_P329890 | TMEM136 | NM_174926 | 2.07 | 1.17E-04 |
| A_24_P152649 | LOC644189 | NR_033748 | 2.07 | 9.37E-04 |
| A_24_P345209 | DYRK3 | NM_001004023 | 2.07 | 2.54E-05 |
| A_23_P12405 | ESPN | NM_031475 | 2.07 | 1.72E-04 |
| A_23_P214766 | HIVEP2 | NM_006734 | 2.07 | 1.12E-04 |
| A_23_P106898 | ORAI3 | NM_152288 | 2.07 | 5.24E-04 |
| A_33_P3531979 | LOC283352 | AK097496 | 2.07 | 8.53E-05 |
| A_24_P160466 | GPRIN1 | NM_052899 | 2.07 | 1.14E-04 |
| A_21_P0006639 | XLOC_008348 | ENST00000436340 | 2.07 | 8.11E-04 |
| A_33_P3236392 | PVRL4 | NM_030916 | 2.07 | 9.96E-04 |
| A_23_P10025 | NELL2 | NM_006159 | 2.08 | 9.87E-05 |
| A_33_P3377760 | DA946325 | DA946325 | 2.08 | 1.43E-05 |
| A_23_P105409 | MAP3K12 | NM_006301 | 2.08 | 5.81E-04 |
| A_21_P0001341 | XLOC_000301 | TCONS_00001035 | 2.08 | 1.20E-04 |
| A_23_P119943 | IGFBP2 | NM_000597 | 2.08 | 2.39E-04 |
| A_23_P22614 | Sep6 | NM_145802 | 2.08 | 2.62E-04 |
| A_23_P35617 | PLCE1 | NM_016341 | 2.08 | 6.22E-04 |
| A_23_P103256 | CFHR3 | NM_021023 | 2.09 | 2.32E-04 |
| A_23_P106835 | BBS2 | NM_031885 | 2.09 | 2.66E-04 |
| A_23_P116037 | TM7SF2 | NM_003273 | 2.09 | 3.15E-04 |
| A_23_P103256 | CFHR3 | NM_021023 | 2.09 | 5.04E-04 |
| A_23_P154338 | EFHD1 | NM_025202 | 2.09 | 1.42E-04 |
| A_23_P12405 | ESPN | NM_031475 | 2.09 | 1.28E-04 |
| A_23_P121253 | TNFSF10 | NM_003810 | 2.09 | 3.83E-04 |
| A_23_P393099 | TFF3 | NM_003226 | 2.09 | 1.13E-04 |
| A_23_P92042 | ITPR1 | NM_002222 | 2.09 | 4.42E-06 |
| A_21_P0011934 | LOC100506123 | NR_040097 | 2.09 | 2.02E-04 |
| A_23_P392384 | AIF1L | NM_001185095 | 2.10 | 4.86E-05 |
| A_24_P135748 | GRTP1 | NM_024719 | 2.10 | 2.15E-05 |
| A_23_P9523 | RBKS | NM_022128 | 2.10 | 6.64E-04 |
| A_23_P106835 | BBS2 | NM_031885 | 2.10 | 6.25E-04 |
| A_23_P1043 | C1orf106 | NM_018265 | 2.10 | 3.76E-06 |
| A_33_P3351536 | PTK2B | NM_173174 | 2.10 | 8.45E-04 |
| A_23_P74112 | IL28RA | NM_170743 | 2.10 | 1.63E-05 |
| A_23_P67278 | ZNF443 | NM_005815 | 2.11 | 3.29E-05 |
| A_23_P114689 | ASAP3 | NM_017707 | 2.11 | 5.71E-04 |
| A_21_P0010997 | LOC100506870 | XR_110520 | 2.11 | 1.89E-05 |
| A_33_P3415092 | CLCN5 | NM_001127899 | 2.11 | 7.46E-04 |
| A_23_P84448 | TUBA4A | NM_006000 | 2.11 | 1.41E-04 |
| A_23_P160618 | SH2D2A | NM_003975 | 2.11 | 4.35E-04 |
| A_23_P67529 | KCNN4 | NM_002250 | 2.11 | 8.37E-05 |
| A_24_P152188 | PRICKLE2 | NM_198859 | 2.11 | 6.69E-04 |
| A_23_P147822 | EPS8L2 | NM_022772 | 2.11 | 4.73E-05 |
| A_23_P106835 | BBS2 | NM_031885 | 2.11 | 1.91E-04 |
| A_23_P104438 | MYPN | NM_032578 | 2.11 | 6.11E-05 |
| A_32_P60459 | OTUD1 | NM_001145373 | 2.11 | 6.75E-06 |
| A_23_P136978 | SRPX2 | NM_014467 | 2.11 | 1.64E-04 |
| A_23_P425332 | PPP4R4 | NM_058237 | 2.12 | 2.25E-04 |
| A_32_P215938 | GPSM1 | NM_001145638 | 2.12 | 5.78E-04 |
| A_23_P1043 | C1orf106 | NM_018265 | 2.12 | 1.20E-04 |
| A_21_P0010328 | XLOC_014161 | ENST00000444039 | 2.12 | 8.12E-04 |
| A_21_P0002604 | XLOC_001576 | ENST00000480208 | 2.12 | 2.70E-04 |
| A_32_P104478 | FGD6 | NM_018351 | 2.12 | 2.03E-04 |
| A_23_P259442 | CPE | NM_001873 | 2.12 | 8.41E-05 |
| A_23_P204087 | OAS2 | NM_016817 | 2.12 | 6.93E-04 |
| A_33_P3293266 | TMEM175 | NM_032326 | 2.12 | 2.60E-05 |
| A_33_P3355014 | TMEM229B | NM_182526 | 2.12 | 5.68E-04 |
| A_23_P110712 | DUSP1 | NM_004417 | 2.12 | 1.63E-04 |
| A_23_P8571 | SRCRB4D | NM_080744 | 2.13 | 3.29E-04 |
| A_32_P209230 | CITED4 | NM_133467 | 2.13 | 2.21E-04 |
| A_23_P128956 | ZFYVE1 | NM_021260 | 2.13 | 2.50E-05 |
| A_23_P106675 | PLCG2 | NM_002661 | 2.13 | 3.61E-05 |
| A_23_P10025 | NELL2 | NM_006159 | 2.14 | 5.53E-04 |
| A_23_P116037 | TM7SF2 | NM_003273 | 2.14 | 3.25E-04 |
| A_33_P3300975 | HOXC4 | NM_014620 | 2.14 | 1.60E-04 |
| A_21_P0014517 | LOC100507613 | ENST00000434985 | 2.14 | 9.55E-05 |
| A_23_P110712 | DUSP1 | NM_004417 | 2.14 | 1.32E-04 |
| A_23_P51487 | GBP3 | NM_018284 | 2.14 | 3.95E-04 |
| A_23_P110712 | DUSP1 | NM_004417 | 2.15 | 3.12E-05 |
| A_23_P103256 | CFHR3 | NM_021023 | 2.15 | 9.42E-05 |
| A_23_P110712 | DUSP1 | NM_004417 | 2.15 | 1.70E-04 |
| A_32_P24585 | SH3PXD2B | NM_001017995 | 2.15 | 5.55E-04 |
| A_23_P115064 | CRABP2 | NM_001878 | 2.15 | 1.76E-04 |
| A_23_P119943 | IGFBP2 | NM_000597 | 2.15 | 4.60E-04 |
| A_23_P110712 | DUSP1 | NM_004417 | 2.15 | 1.09E-04 |
| A_21_P0011339 | XLOC_l2_004772 | TCONS_l2_00008799 | 2.15 | 1.02E-04 |
| A_19_P00321333 | NEAT1 | NR_028272 | 2.15 | 8.01E-04 |
| A_24_P55496 | OSR2 | NM_053001 | 2.16 | 1.23E-04 |
| A_23_P164179 | TOB1 | NM_005749 | 2.16 | 1.87E-04 |
| A_23_P85693 | GBP2 | NM_004120 | 2.16 | 8.31E-05 |
| A_23_P123086 | KIAA1908 | NR_027329 | 2.16 | 5.30E-04 |
| A_19_P00321332 | NEAT1 | NR_028272 | 2.16 | 7.99E-04 |
| A_33_P3410449 | SCARF2 | NM_153334 | 2.16 | 6.35E-04 |
| A_23_P31873 | RAB11FIP1 | NM_001002814 | 2.17 | 4.27E-05 |
| A_23_P106898 | ORAI3 | NM_152288 | 2.17 | 7.19E-04 |
| A_23_P116037 | TM7SF2 | NM_003273 | 2.17 | 9.88E-04 |
| A_24_P316019 | ENST00000333156 | ENST00000333156 | 2.17 | 9.00E-04 |
| A_24_P289178 | C16orf74 | NM_206967 | 2.17 | 1.41E-04 |
| A_21_P0006971 | XLOC_008730 | TCONS_00018438 | 2.17 | 6.55E-04 |
| A_23_P250212 | SGK223 | NM_001080826 | 2.17 | 5.02E-06 |
| A_23_P10025 | NELL2 | NM_006159 | 2.17 | 9.71E-05 |
| A_23_P75741 | UBE2L6 | NM_198183 | 2.17 | 1.32E-05 |
| A_23_P110712 | DUSP1 | NM_004417 | 2.18 | 6.56E-05 |
| A_24_P941166 | ZNF425 | NM_001001661 | 2.18 | 2.15E-04 |
| A_33_P3228558 | ARHGAP27 | NM_199282 | 2.18 | 4.20E-06 |
| A_24_P365975 | COL8A2 | NM_005202 | 2.18 | 1.33E-04 |
| A_23_P3963 | CDR2L | NM_014603 | 2.19 | 3.58E-04 |
| A_23_P100539 | ABCC6 | NM_001171 | 2.19 | 2.04E-04 |
| A_23_P93988 | ARHGEF5 | NM_005435 | 2.19 | 1.44E-05 |
| A_23_P154065 | TUBA4A | NM_006000 | 2.19 | 2.52E-05 |
| A_23_P132763 | VGLL3 | NM_016206 | 2.19 | 1.74E-04 |
| A_21_P0013462 | XLOC_l2_014077 | ENST00000418309 | 2.19 | 6.24E-04 |
| A_23_P27285 | MPPE1 | NM_023075 | 2.19 | 1.11E-05 |
| A_23_P34827 | HCN3 | NM_020897 | 2.19 | 4.53E-04 |
| A_23_P128919 | LGALS3 | NM_002306 | 2.19 | 1.28E-05 |
| A_23_P100539 | ABCC6 | NM_001171 | 2.19 | 7.99E-04 |
| A_23_P103256 | CFHR3 | NM_021023 | 2.20 | 1.12E-04 |
| A_23_P106675 | PLCG2 | NM_002661 | 2.20 | 3.48E-05 |
| A_23_P32404 | ISG20 | NM_002201 | 2.20 | 8.77E-04 |
| A_33_P3346669 | PLCE1 | NM_016341 | 2.21 | 2.71E-04 |
| A_23_P255126 | GAB3 | NM_080612 | 2.21 | 8.13E-04 |
| A_23_P104438 | MYPN | NM_032578 | 2.21 | 1.96E-04 |
| A_23_P110712 | DUSP1 | NM_004417 | 2.21 | 1.78E-05 |
| A_24_P335305 | OAS3 | NM_006187 | 2.21 | 3.65E-04 |
| A_23_P5983 | PLTP | NM_006227 | 2.21 | 5.45E-04 |
| A_23_P119143 | ICAM5 | NM_003259 | 2.21 | 3.64E-04 |
| A_23_P25994 | LGMN | NM_001008530 | 2.21 | 4.14E-05 |
| A_24_P379353 | CCDC24 | NM_152499 | 2.21 | 2.61E-04 |
| A_23_P106675 | PLCG2 | NM_002661 | 2.21 | 2.77E-04 |
| A_23_P119143 | ICAM5 | NM_003259 | 2.21 | 7.44E-04 |
| A_23_P127584 | NNMT | NM_006169 | 2.22 | 6.80E-04 |
| A_24_P64653 | METTL7B | NM_152637 | 2.22 | 9.77E-04 |
| A_32_P104432 | LINC00087 | NR_024493 | 2.22 | 1.80E-04 |
| A_32_P141238 | ANO2 | NM_020373 | 2.22 | 4.72E-04 |
| A_23_P34915 | ATF3 | NM_001040619 | 2.22 | 6.49E-05 |
| A_23_P103256 | CFHR3 | NM_021023 | 2.22 | 3.00E-04 |
| A_32_P157945 | DSP | NM_004415 | 2.22 | 5.01E-05 |
| A_23_P168882 | TP53INP1 | NM_033285 | 2.22 | 8.99E-04 |
| A_23_P99853 | KIAA1370 | NM_019600 | 2.23 | 8.68E-05 |
| A_33_P3402329 | MGC16121 | NR_024607 | 2.23 | 6.25E-04 |
| A_23_P101476 | ZNF442 | NM_030824 | 2.23 | 2.56E-04 |
| A_23_P101476 | ZNF442 | NM_030824 | 2.23 | 5.01E-04 |
| A_21_P0004632 | XLOC_005167 | ENST00000366312 | 2.23 | 5.45E-04 |
| A_23_P327361 | DMXL2 | NM_015263 | 2.23 | 4.75E-04 |
| A_23_P166336 | TMEM191A | NR_026815 | 2.23 | 1.45E-05 |
| A_33_P3387796 | PHLDB1 | NM_015157 | 2.23 | 4.35E-05 |
| A_24_P117323 | KLHL22 | NM_032775 | 2.24 | 1.88E-04 |
| A_21_P0005891 | XLOC_007189 | TCONS_00015146 | 2.24 | 7.76E-04 |
| A_33_P3352148 | AGAP2 | NM_001122772 | 2.24 | 5.64E-05 |
| A_23_P110712 | DUSP1 | NM_004417 | 2.24 | 3.07E-05 |
| A_23_P110712 | DUSP1 | NM_004417 | 2.24 | 1.66E-05 |
| A_24_P215765 | ATP10A | NM_024490 | 2.24 | 5.19E-04 |
| A_23_P1043 | C1orf106 | NM_018265 | 2.24 | 1.49E-04 |
| A_23_P104438 | MYPN | NM_032578 | 2.24 | 7.33E-06 |
| A_23_P21560 | FAM49A | NM_030797 | 2.24 | 1.50E-04 |
| A_23_P38154 | FDXR | NM_004110 | 2.24 | 8.66E-05 |
| A_23_P119143 | ICAM5 | NM_003259 | 2.25 | 8.96E-04 |
| A_23_P110712 | DUSP1 | NM_004417 | 2.25 | 1.39E-04 |
| A_24_P85775 | C1orf38 | NM_001039477 | 2.25 | 7.40E-05 |
| A_23_P106675 | PLCG2 | NM_002661 | 2.25 | 3.18E-05 |
| A_33_P3402565 | DSP | NM_004415 | 2.25 | 9.68E-05 |
| A_23_P348264 | LETM2 | NM_144652 | 2.25 | 1.65E-04 |
| A_23_P119143 | ICAM5 | NM_003259 | 2.26 | 9.59E-04 |
| A_33_P3322519 | VWA5B2 | NM_138345 | 2.26 | 9.64E-04 |
| A_33_P3357591 | ATHL1 | NM_025092 | 2.26 | 5.48E-04 |
| A_23_P106675 | PLCG2 | NM_002661 | 2.26 | 3.16E-04 |
| A_21_P0002205 | XLOC_001448 | TCONS_00003661 | 2.26 | 9.59E-06 |
| A_23_P24555 | PHLDB1 | NM_015157 | 2.26 | 1.90E-05 |
| A_23_P154855 | KCNE1 | NM_000219 | 2.26 | 1.10E-05 |
| A_33_P3360728 | BLVRB | NM_000713 | 2.26 | 2.93E-05 |
| A_19_P00318409 | NEAT1 | NR_028272 | 2.26 | 5.45E-04 |
| A_23_P69310 | CCRL2 | NM_003965 | 2.26 | 1.05E-04 |
| A_33_P3259135 | D4S234E | NM_014392 | 2.26 | 6.31E-04 |
| A_23_P1043 | C1orf106 | NM_018265 | 2.27 | 6.56E-04 |
| A_23_P104438 | MYPN | NM_032578 | 2.27 | 2.86E-06 |
| A_33_P3719083 | CHRFAM7A | NM_139320 | 2.27 | 8.65E-04 |
| A_23_P90079 | ZNF799 | NM_001080821 | 2.27 | 1.20E-04 |
| A_23_P101476 | ZNF442 | NM_030824 | 2.27 | 5.06E-04 |
| A_24_P273253 | AHNAK2 | NM_138420 | 2.27 | 3.15E-05 |
| A_23_P101476 | ZNF442 | NM_030824 | 2.28 | 2.23E-04 |
| A_21_P0003478 | XLOC_003986 | ENST00000505930 | 2.28 | 6.77E-04 |
| A_21_P0000132 | C16orf93 | NM_001195620 | 2.28 | 1.71E-04 |
| A_24_P33895 | ATF3 | NM_001040619 | 2.28 | 7.27E-04 |
| A_23_P101476 | ZNF442 | NM_030824 | 2.28 | 3.33E-04 |
| A_24_P201171 | STXBP1 | NM_003165 | 2.28 | 2.92E-04 |
| A_23_P819 | ISG15 | NM_005101 | 2.28 | 4.10E-05 |
| A_23_P118203 | ZG16B | NM_145252 | 2.28 | 2.71E-04 |
| A_23_P71480 | DEFB1 | NM_005218 | 2.28 | 1.53E-04 |
| A_23_P215549 | PON3 | NM_000940 | 2.28 | 1.04E-05 |
| A_23_P119143 | ICAM5 | NM_003259 | 2.29 | 7.60E-04 |
| A_33_P3369760 | GLIPR2 | NM_022343 | 2.29 | 2.51E-04 |
| A_33_P3314594 | RAB37 | NM_175738 | 2.29 | 2.90E-05 |
| A_33_P3358208 | PADI1 | NM_013358 | 2.29 | 5.15E-04 |
| A_23_P78053 | FAM117A | NM_030802 | 2.30 | 6.85E-04 |
| A_23_P133543 | KLHL3 | NM_017415 | 2.30 | 7.87E-04 |
| A_24_P410797 | KALRN | AK125979 | 2.30 | 9.80E-04 |
| A_23_P114689 | ASAP3 | NM_017707 | 2.30 | 1.89E-04 |
| A_23_P114689 | ASAP3 | NM_017707 | 2.30 | 9.61E-04 |
| A_19_P00318172 | ENST00000485364 | ENST00000485364 | 2.30 | 1.93E-04 |
| A_23_P116037 | TM7SF2 | NM_003273 | 2.30 | 5.61E-04 |
| A_23_P153320 | ICAM1 | NM_000201 | 2.30 | 1.52E-04 |
| A_23_P106675 | PLCG2 | NM_002661 | 2.30 | 3.76E-05 |
| A_19_P00320579 | FLJ43663 | NR_015431 | 2.30 | 1.65E-04 |
| A_23_P51002 | SULT1C2 | NM_176825 | 2.31 | 1.44E-04 |
| A_23_P1043 | C1orf106 | NM_018265 | 2.31 | 4.02E-05 |
| A_21_P0008655 | XLOC_011307 | TCONS_00023462 | 2.31 | 1.32E-06 |
| A_23_P116280 | WT1 | NM_024426 | 2.31 | 7.82E-05 |
| A_23_P62901 | BTG2 | NM_006763 | 2.31 | 2.25E-04 |
| A_23_P12405 | ESPN | NM_031475 | 2.32 | 1.38E-04 |
| A_23_P143143 | ID2 | NM_002166 | 2.32 | 5.24E-04 |
| A_23_P78980 | B3GNT3 | NM_014256 | 2.32 | 3.68E-04 |
| A_23_P25674 | CKB | NM_001823 | 2.32 | 6.82E-06 |
| A_23_P114689 | ASAP3 | NM_017707 | 2.32 | 2.78E-04 |
| A_23_P104438 | MYPN | NM_032578 | 2.32 | 1.09E-04 |
| A_23_P1043 | C1orf106 | NM_018265 | 2.32 | 3.36E-04 |
| A_33_P3825869 | CACNA1C | NM_199460 | 2.32 | 1.77E-04 |
| A_23_P404667 | BIK | NM_001197 | 2.32 | 2.13E-05 |
| A_23_P101476 | ZNF442 | NM_030824 | 2.33 | 1.27E-04 |
| A_21_P0012289 | GGT1 | NM_005265 | 2.33 | 7.19E-04 |
| A_21_P0004229 | XLOC_004297 | TCONS_00009876 | 2.33 | 1.18E-04 |
| A_23_P104438 | MYPN | NM_032578 | 2.33 | 1.06E-04 |
| A_19_P00813077 | LOC375190 | NM_001145710 | 2.33 | 2.88E-05 |
| A_32_P115050 | LOC646576 | NR_037595 | 2.33 | 2.63E-05 |
| A_23_P106675 | PLCG2 | NM_002661 | 2.33 | 9.07E-04 |
| A_23_P117298 | F7 | NM_000131 | 2.33 | 8.78E-04 |
| A_21_P0004242 | XLOC_004350 | BG207267 | 2.34 | 3.64E-04 |
| A_33_P3295098 | ESYT3 | ENST00000289135 | 2.34 | 7.88E-04 |
| A_23_P13713 | PRPH | NM_006262 | 2.34 | 1.45E-05 |
| A_24_P101800 | ENST00000485364 | ENST00000485364 | 2.34 | 8.91E-04 |
| A_23_P421175 | FAM83H | NM_198488 | 2.34 | 3.75E-05 |
| A_33_P3417626 | ENHO | NM_198573 | 2.34 | 7.81E-04 |
| A_33_P3415633 | TMEM136 | NM_001198670 | 2.35 | 8.05E-04 |
| A_21_P0014697 | LOC100509315 | XR_111795 | 2.35 | 7.27E-04 |
| A_23_P422911 | HS6ST3 | NM_153456 | 2.35 | 3.49E-04 |
| A_23_P1492 | AVPI1 | NM_021732 | 2.35 | 5.97E-04 |
| A_24_P40594 | HOMEZ | NM_020834 | 2.36 | 2.41E-04 |
| A_23_P386942 | DIRAS1 | NM_145173 | 2.36 | 5.03E-05 |
| A_21_P0008125 | LOC100506394 | NR_044993 | 2.36 | 4.25E-04 |
| A_33_P3258346 | XAF1 | NM_017523 | 2.36 | 5.42E-04 |
| A_21_P0013346 | XLOC_l2_013963 | ENST00000451962 | 2.36 | 9.37E-04 |
| A_33_P3409159 | SLC22A23 | NM_015482 | 2.37 | 3.57E-04 |
| A_23_P114689 | ASAP3 | NM_017707 | 2.37 | 2.88E-05 |
| A_23_P10873 | TLR1 | NM_003263 | 2.37 | 5.76E-05 |
| A_24_P390833 | MPPE1 | NM_023075 | 2.37 | 2.22E-05 |
| A_23_P10873 | TLR1 | NM_003263 | 2.38 | 4.85E-04 |
| A_23_P153320 | ICAM1 | NM_000201 | 2.38 | 9.64E-04 |
| A_23_P333498 | EEPD1 | NM_030636 | 2.38 | 1.47E-04 |
| A_33_P3377187 | BK250D10.8 | NR_024355 | 2.38 | 3.35E-04 |
| A_23_P12241 | MCOLN3 | NM_018298 | 2.38 | 4.89E-06 |
| A_24_P109214 | APOC1 | NM_001645 | 2.38 | 1.63E-05 |
| A_23_P143885 | ARHGEF3 | NM_019555 | 2.38 | 5.56E-05 |
| A_32_P107493 | C16orf55 | NM_153025 | 2.38 | 6.20E-05 |
| A_23_P12405 | ESPN | NM_031475 | 2.39 | 1.59E-04 |
| A_23_P385126 | DEPDC7 | NM_139160 | 2.39 | 9.47E-05 |
| A_23_P30603 | DDO | NM_003649 | 2.39 | 9.65E-04 |
| A_33_P3380236 | ENST00000423408 | ENST00000423408 | 2.39 | 5.03E-04 |
| A_32_P69368 | ID2 | NM_002166 | 2.39 | 4.80E-04 |
| A_33_P3795524 | LOC100506485 | XR_110000 | 2.39 | 2.76E-04 |
| A_23_P153320 | ICAM1 | NM_000201 | 2.39 | 5.98E-04 |
| A_23_P17695 | SLC37A1 | NM_018964 | 2.39 | 6.01E-05 |
| A_21_P0014166 | LOC100506379 | XR_108672 | 2.39 | 9.04E-05 |
| A_23_P49499 | ST6GALNAC2 | NM_006456 | 2.39 | 8.71E-04 |
| A_24_P6370 | C1orf110 | NM_178550 | 2.40 | 4.94E-04 |
| A_23_P12405 | ESPN | NM_031475 | 2.40 | 3.09E-04 |
| A_23_P384748 | PLEKHH2 | NM_172069 | 2.40 | 1.74E-04 |
| A_23_P153320 | ICAM1 | NM_000201 | 2.40 | 8.40E-04 |
| A_21_P0006166 | XLOC_007433 | BI046002 | 2.40 | 2.35E-04 |
| A_33_P3298024 | ABCC3 | NM_001144070 | 2.40 | 2.20E-05 |
| A_23_P101476 | ZNF442 | NM_030824 | 2.40 | 6.33E-04 |
| A_24_P62530 | RHOU | NM_021205 | 2.41 | 4.92E-04 |
| A_32_P202759 | FAM171B | NM_177454 | 2.41 | 8.08E-05 |
| A_23_P162486 | PTPN6 | NM_002831 | 2.41 | 3.17E-04 |
| A_23_P104464 | ALOX5 | NM_000698 | 2.41 | 3.01E-04 |
| A_23_P6413 | SELM | NM_080430 | 2.41 | 1.54E-04 |
| A_33_P3233580 | KIAA1217 | AK125675 | 2.41 | 1.60E-04 |
| A_23_P14673 | IGDCC4 | NM_020962 | 2.41 | 3.11E-04 |
| A_24_P229164 | HIP1R | NM_003959 | 2.41 | 6.47E-05 |
| A_24_P49383 | C11orf67 | NM_024684 | 2.42 | 4.57E-06 |
| A_33_P3261408 | TMIE | NM_147196 | 2.42 | 7.19E-04 |
| A_23_P259292 | C1QTNF5 | NM_015645 | 2.42 | 5.82E-04 |
| A_24_P296587 | DLX3 | NM_005220 | 2.42 | 5.90E-04 |
| A_19_P00331853 | LOC100131564 | NR_034089 | 2.42 | 4.33E-04 |
| A_23_P10873 | TLR1 | NM_003263 | 2.42 | 1.81E-04 |
| A_23_P66948 | FAM59A | NM_022751 | 2.42 | 1.94E-04 |
| A_23_P42784 | STK31 | NM_032944 | 2.43 | 9.72E-04 |
| A_23_P22350 | GRAMD3 | NM_023927 | 2.43 | 1.91E-04 |
| A_23_P104438 | MYPN | NM_032578 | 2.43 | 4.40E-04 |
| A_33_P3338928 | DAB2 | NM_001343 | 2.43 | 8.22E-05 |
| A_33_P3358183 | PTGFR | NM_000959 | 2.43 | 9.52E-05 |
| A_33_P3423270 | TMEM40 | NM_018306 | 2.43 | 9.76E-05 |
| A_23_P88303 | HSPA2 | NM_021979 | 2.44 | 1.27E-04 |
| A_23_P114689 | ASAP3 | NM_017707 | 2.44 | 5.45E-05 |
| A_33_P3225522 | OAS2 | NM_001032731 | 2.44 | 3.79E-04 |
| A_21_P0000749 | LOC100506994 | NR_038281 | 2.44 | 6.84E-04 |
| A_21_P0005869 | XLOC_007149 | TCONS_00015084 | 2.44 | 4.04E-05 |
| A_23_P105862 | FRY | NM_023037 | 2.45 | 1.98E-04 |
| A_21_P0008404 | XLOC_010952 | BM562684 | 2.45 | 8.56E-05 |
| A_33_P3342628 | HES4 | NM_021170 | 2.45 | 4.67E-05 |
| A_23_P79251 | EHD3 | NM_014600 | 2.45 | 9.77E-04 |
| A_23_P53137 | HBG1 | NM_000559 | 2.46 | 5.27E-04 |
| A_23_P104464 | ALOX5 | NM_000698 | 2.46 | 7.25E-04 |
| A_23_P73571 | MUM1L1 | NM_152423 | 2.46 | 4.16E-04 |
| A_23_P119040 | GREB1L | NM_001142966 | 2.46 | 4.17E-04 |
| A_33_P3410599 | FAM46A | NM_017633 | 2.46 | 2.70E-04 |
| A_23_P80040 | PROCR | NM_006404 | 2.46 | 2.41E-05 |
| A_23_P12405 | ESPN | NM_031475 | 2.46 | 8.99E-04 |
| A_23_P10873 | TLR1 | NM_003263 | 2.46 | 3.42E-04 |
| A_23_P116037 | TM7SF2 | NM_003273 | 2.46 | 2.42E-04 |
| A_23_P87310 | LMO1 | NM_002315 | 2.47 | 2.58E-05 |
| A_21_P0008779 | XLOC_011577 | TCONS_00023782 | 2.47 | 8.27E-04 |
| A_23_P79803 | VSTM2L | NM_080607 | 2.47 | 2.50E-05 |
| A_33_P3367850 | CHRM4 | NM_000741 | 2.47 | 6.33E-04 |
| A_23_P114689 | ASAP3 | NM_017707 | 2.48 | 4.59E-05 |
| A_23_P38346 | DHX58 | NM_024119 | 2.48 | 2.78E-04 |
| A_23_P76823 | ADSSL1 | NM_199165 | 2.48 | 3.92E-04 |
| A_33_P3293362 | Mar1 | NM_022746 | 2.48 | 6.70E-04 |
| A_23_P10873 | TLR1 | NM_003263 | 2.48 | 2.25E-04 |
| A_23_P162486 | PTPN6 | NM_002831 | 2.48 | 6.20E-04 |
| A_23_P254741 | SOD3 | NM_003102 | 2.48 | 2.32E-05 |
| A_21_P0001471 | XLOC_000721 | TCONS_00001406 | 2.49 | 6.62E-04 |
| A_23_P422071 | B3GALT4 | NM_003782 | 2.49 | 8.34E-05 |
| A_24_P403417 | PTGES | NM_004878 | 2.49 | 7.38E-05 |
| A_23_P114689 | ASAP3 | NM_017707 | 2.50 | 4.67E-04 |
| A_24_P247902 | PCLO | NM_014510 | 2.50 | 3.13E-04 |
| A_23_P103672 | NES | NM_006617 | 2.50 | 6.31E-04 |
| A_23_P62634 | RHCE | NM_020485 | 2.51 | 2.06E-04 |
| A_23_P63032 | GUCA2B | NM_007102 | 2.51 | 9.42E-05 |
| A_33_P3383866 | TREX1 | NM_016381 | 2.51 | 4.28E-05 |
| A_21_P0012781 | XLOC_l2_011204 | ENST00000512915 | 2.51 | 9.71E-05 |
| A_23_P85164 | DNASE1L1 | NM_006730 | 2.52 | 1.98E-05 |
| A_19_P00320275 | XLOC_008079 | ENST00000498732 | 2.52 | 2.13E-05 |
| A_33_P3318796 | FSTL3 | NM_005860 | 2.52 | 5.27E-05 |
| A_33_P3345812 | GPER | NM_001039966 | 2.52 | 7.33E-04 |
| A_32_P181527 | C8orf85 | NM_001025357 | 2.52 | 3.35E-04 |
| A_23_P12241 | MCOLN3 | NM_018298 | 2.52 | 6.95E-04 |
| A_23_P151805 | FBLN5 | NM_006329 | 2.52 | 1.49E-04 |
| A_23_P114689 | ASAP3 | NM_017707 | 2.52 | 3.91E-04 |
| A_33_P3352712 | CDR2L | NM_014603 | 2.52 | 3.36E-04 |
| A_23_P26629 | PYCARD | NM_013258 | 2.53 | 7.65E-07 |
| A_33_P3338733 | MITF | NM_198159 | 2.53 | 7.77E-05 |
| A_23_P416178 | CCDC136 | NM_022742 | 2.53 | 2.33E-04 |
| A_21_P0008224 | LOC440149 | AK057085 | 2.53 | 9.73E-04 |
| A_23_P104464 | ALOX5 | NM_000698 | 2.53 | 2.11E-04 |
| A_23_P119353 | RASIP1 | NM_017805 | 2.53 | 4.05E-04 |
| A_23_P426663 | MITF | NM_198159 | 2.53 | 2.04E-04 |
| A_23_P48596 | RNASE1 | NM_198232 | 2.53 | 9.44E-05 |
| A_24_P97405 | CCRL2 | NM_003965 | 2.54 | 1.62E-04 |
| A_23_P12405 | ESPN | NM_031475 | 2.54 | 3.90E-04 |
| A_33_P3222380 | AHNAK2 | NM_138420 | 2.54 | 1.87E-04 |
| A_23_P117582 | JDP2 | NM_130469 | 2.54 | 1.37E-05 |
| A_21_P0008398 | XLOC_010945 | TCONS_00022642 | 2.54 | 1.81E-05 |
| A_23_P404494 | IL7R | NM_002185 | 2.54 | 2.79E-04 |
| A_23_P422831 | FAM189A2 | NM_004816 | 2.54 | 5.61E-05 |
| A_23_P100539 | ABCC6 | NM_001171 | 2.54 | 1.89E-04 |
| A_23_P12241 | MCOLN3 | NM_018298 | 2.54 | 5.57E-04 |
| A_33_P3333317 | OPTN | NM_001008211 | 2.55 | 1.06E-04 |
| A_23_P12241 | MCOLN3 | NM_018298 | 2.55 | 1.16E-04 |
| A_33_P3353552 | SLC48A1 | NM_017842 | 2.55 | 9.76E-04 |
| A_23_P148990 | HMCN1 | NM_031935 | 2.55 | 8.36E-04 |
| A_33_P3378514 | PDE5A | NM_001083 | 2.55 | 4.54E-05 |
| A_23_P162486 | PTPN6 | NM_002831 | 2.55 | 7.10E-04 |
| A_23_P53193 | SYTL2 | NM_206927 | 2.56 | 1.15E-04 |
| A_24_P410408 | KRT83 | NM_002282 | 2.56 | 2.35E-04 |
| A_23_P53137 | HBG1 | NM_000559 | 2.56 | 6.62E-04 |
| A_23_P119040 | GREB1L | NM_001142966 | 2.56 | 6.24E-04 |
| A_23_P65240 | COL4A1 | NM_001845 | 2.57 | 2.51E-05 |
| A_23_P37127 | FOXA1 | NM_004496 | 2.57 | 5.80E-04 |
| A_33_P3377750 | KLC3 | NM_177417 | 2.58 | 1.42E-05 |
| A_23_P321501 | DHRS2 | NM_182908 | 2.58 | 2.92E-04 |
| A_21_P0011280 | XLOC_l2_004212 | ENST00000229465 | 2.59 | 1.21E-05 |
| A_21_P0013788 | FRMPD3 | ENST00000276185 | 2.59 | 5.69E-05 |
| A_32_P218355 | C6orf132 | NM_001164446 | 2.59 | 4.23E-04 |
| A_23_P16252 | KLK1 | NM_002257 | 2.59 | 3.31E-04 |
| A_24_P252364 | NRCAM | NM_001037132 | 2.59 | 1.88E-04 |
| A_21_P0012674 | XLOC_l2_011204 | ENST00000512915 | 2.60 | 7.61E-05 |
| A_23_P12241 | MCOLN3 | NM_018298 | 2.60 | 7.45E-05 |
| A_23_P28953 | DNMT3B | NM_175850 | 2.60 | 2.19E-05 |
| A_23_P119040 | GREB1L | NM_001142966 | 2.60 | 7.43E-04 |
| A_23_P114983 | TRIM63 | NM_032588 | 2.60 | 2.69E-04 |
| A_24_P67988 | FRMD8 | NM_031904 | 2.61 | 1.51E-04 |
| A_21_P0013073 | XLOC_l2_012929 | TCONS_l2_00024680 | 2.61 | 8.68E-04 |
| A_23_P117582 | JDP2 | NM_130469 | 2.61 | 2.33E-05 |
| A_33_P3369844 | CD24 | NM_013230 | 2.61 | 4.57E-06 |
| A_23_P349566 | CCDC85A | NM_001080433 | 2.61 | 2.05E-05 |
| A_24_P296508 | SLC43A2 | NM_152346 | 2.61 | 6.25E-05 |
| A_23_P168610 | TSPAN13 | NM_014399 | 2.61 | 2.97E-05 |
| A_33_P3380063 | ADAMTSL4 | NM_025008 | 2.62 | 2.28E-05 |
| A_23_P103486 | CYP2J2 | NM_000775 | 2.62 | 1.27E-05 |
| A_23_P354908 | NMNAT2 | NM_015039 | 2.62 | 6.07E-04 |
| A_33_P3273552 | KRT83 | NM_002282 | 2.62 | 6.92E-05 |
| A_23_P73345 | MITF | NM_198159 | 2.63 | 4.75E-04 |
| A_21_P0012978 | XLOC_l2_011983 | ENST00000505339 | 2.63 | 3.85E-05 |
| A_33_P3268304 | LIMS2 | NM_001161404 | 2.63 | 2.55E-04 |
| A_33_P3414912 | NPL | NM_001200056 | 2.64 | 2.34E-04 |
| A_23_P117582 | JDP2 | NM_130469 | 2.64 | 1.29E-05 |
| A_23_P122924 | INHBA | NM_002192 | 2.64 | 4.30E-04 |
| A_23_P201181 | PTPN22 | NM_012411 | 2.64 | 3.38E-05 |
| A_23_P215900 | SCARA3 | NM_016240 | 2.64 | 3.69E-05 |
| A_33_P3323842 | BDNF-AS1 | NR_002832 | 2.64 | 2.09E-04 |
| A_33_P3397658 | SYNPO | ENST00000394243 | 2.64 | 3.99E-05 |
| A_23_P117582 | JDP2 | NM_130469 | 2.64 | 4.30E-05 |
| A_33_P3262789 | REEP6 | NM_138393 | 2.64 | 1.88E-06 |
| A_33_P3230818 | RCAN2 | NM_001251973 | 2.65 | 2.13E-04 |
| A_24_P133584 | MFGE8 | NM_005928 | 2.65 | 4.34E-05 |
| A_23_P425925 | KRT222 | NM_152349 | 2.65 | 1.85E-05 |
| A_33_P3317613 | SYN2 | NM_133625 | 2.65 | 1.34E-04 |
| A_23_P117582 | JDP2 | NM_130469 | 2.65 | 5.10E-06 |
| A_23_P133902 | PSORS1C1 | NM_014068 | 2.66 | 1.25E-06 |
| A_21_P0012585 | XLOC_l2_010679 | TCONS_l2_00020535 | 2.66 | 4.81E-05 |
| A_33_P3295358 | ANGPTL4 | NM_139314 | 2.66 | 4.24E-05 |
| A_33_P3403254 | ZNF878 | NM_001080404 | 2.67 | 3.39E-04 |
| A_23_P501822 | JUP | NM_002230 | 2.67 | 1.50E-04 |
| A_23_P122924 | INHBA | NM_002192 | 2.67 | 8.38E-04 |
| A_23_P12241 | MCOLN3 | NM_018298 | 2.67 | 2.51E-04 |
| A_23_P34537 | EPHX1 | NM_000120 | 2.67 | 3.13E-05 |
| A_21_P0000036 | RDM1 | NM_145654 | 2.67 | 8.05E-04 |
| A_21_P0011250 | FLJ22447 | NR_039985 | 2.67 | 6.33E-05 |
| A_23_P122924 | INHBA | NM_002192 | 2.67 | 5.01E-04 |
| A_23_P119040 | GREB1L | NM_001142966 | 2.67 | 4.73E-04 |
| A_23_P99642 | SLC7A7 | NM_001126106 | 2.68 | 6.71E-04 |
| A_23_P112634 | C4orf34 | NM_174921 | 2.68 | 3.52E-04 |
| A_23_P10873 | TLR1 | NM_003263 | 2.68 | 1.62E-04 |
| A_23_P28507 | MGAT4A | NM_012214 | 2.68 | 1.01E-04 |
| A_33_P3219942 | EML1 | NM_001008707 | 2.68 | 4.33E-04 |
| A_23_P120863 | GAL3ST1 | NM_004861 | 2.68 | 3.31E-04 |
| A_33_P3234580 | ASS1 | NM_000050 | 2.69 | 5.35E-06 |
| A_23_P1292 | ERCC6 | NM_000124 | 2.69 | 3.45E-04 |
| A_23_P372946 | TM4SF19 | NM_138461 | 2.69 | 2.98E-04 |
| A_33_P3306654 | LOC100131551 | NR_024480 | 2.69 | 8.41E-04 |
| A_23_P117582 | JDP2 | NM_130469 | 2.69 | 3.27E-05 |
| A_23_P392126 | C17orf108 | NM_001076680 | 2.69 | 5.03E-06 |
| A_23_P104464 | ALOX5 | NM_000698 | 2.70 | 1.04E-04 |
| A_23_P97860 | LIPA | NM_000235 | 2.70 | 1.00E-05 |
| A_23_P103486 | CYP2J2 | NM_000775 | 2.70 | 2.15E-05 |
| A_24_P393958 | DNAJB4 | NM_007034 | 2.70 | 1.53E-04 |
| A_23_P204016 | CACNB3 | NM_000725 | 2.70 | 1.09E-04 |
| A_23_P360804 | CPNE5 | NM_020939 | 2.70 | 1.96E-04 |
| A_23_P359214 | LOC643650 | NR_033957 | 2.70 | 3.64E-04 |
| A_23_P12241 | MCOLN3 | NM_018298 | 2.70 | 1.61E-04 |
| A_21_P0009003 | XLOC_011865 | THC2684660 | 2.71 | 9.67E-04 |
| A_33_P3213029 | RNF43 | NM_017763 | 2.71 | 1.09E-04 |
| A_23_P76245 | SCN8A | NM_014191 | 2.71 | 4.59E-04 |
| A_23_P110412 | TMEM150C | NM_001080506 | 2.71 | 9.41E-05 |
| A_33_P3369153 | KIF3C | NM_002254 | 2.72 | 3.66E-05 |
| A_24_P342632 | AK5 | NM_174858 | 2.72 | 6.95E-04 |
| A_33_P3275615 | THC2786689 | THC2786689 | 2.72 | 8.39E-04 |
| A_23_P103486 | CYP2J2 | NM_000775 | 2.72 | 8.36E-08 |
| A_23_P122924 | INHBA | NM_002192 | 2.72 | 2.87E-04 |
| A_23_P104464 | ALOX5 | NM_000698 | 2.72 | 3.51E-04 |
| A_33_P3259393 | HAPLN3 | NM_178232 | 2.72 | 1.67E-04 |
| A_23_P423427 | FAM171B | NM_177454 | 2.72 | 2.06E-04 |
| A_23_P406341 | AFAP1L2 | NM_001001936 | 2.72 | 3.40E-06 |
| A_23_P212042 | MFI2 | NM_005929 | 2.72 | 8.28E-04 |
| A_33_P3391603 | LAMA4 | NM_001105209 | 2.73 | 6.48E-04 |
| A_33_P3268181 | LIMS2 | NM_001161404 | 2.73 | 4.35E-04 |
| A_23_P206310 | KIAA0513 | NM_014732 | 2.73 | 8.73E-04 |
| A_33_P3252834 | PHLDA3 | NM_012396 | 2.73 | 9.34E-06 |
| A_23_P163227 | CKMT1A | NM_001015001 | 2.73 | 7.24E-06 |
| A_21_P0008267 | LOC440149 | AK057085 | 2.73 | 8.95E-04 |
| A_23_P12241 | MCOLN3 | NM_018298 | 2.73 | 2.17E-04 |
| A_23_P201459 | IFI6 | NM_022873 | 2.74 | 3.02E-05 |
| A_32_P351968 | HLA-DMB | NM_002118 | 2.74 | 2.13E-06 |
| A_23_P122924 | INHBA | NM_002192 | 2.74 | 2.54E-04 |
| A_32_P41065 | TMCC1 | NM_001017395 | 2.74 | 5.17E-04 |
| A_23_P103486 | CYP2J2 | NM_000775 | 2.74 | 1.29E-05 |
| A_23_P1523 | RHOD | NM_014578 | 2.74 | 2.86E-05 |
| A_23_P15876 | ALPK2 | NM_052947 | 2.74 | 6.51E-04 |
| A_23_P122924 | INHBA | NM_002192 | 2.74 | 3.88E-04 |
| A_23_P371824 | TUFT1 | NM_020127 | 2.74 | 3.16E-04 |
| A_23_P31921 | ASS1 | NM_000050 | 2.74 | 9.76E-05 |
| A_24_P10137 | C13orf15 | NM_014059 | 2.74 | 8.63E-06 |
| A_23_P102391 | SLC40A1 | NM_014585 | 2.74 | 7.47E-04 |
| A_23_P104464 | ALOX5 | NM_000698 | 2.75 | 2.97E-04 |
| A_24_P450285 | CCDC153 | NM_001145018 | 2.75 | 6.67E-04 |
| A_23_P116512 | PRR5L | NM_024841 | 2.75 | 1.76E-04 |
| A_24_P133253 | KITLG | NM_000899 | 2.75 | 3.86E-04 |
| A_33_P3258607 | LOC100506422 | NM_001004352 | 2.75 | 5.14E-04 |
| A_24_P48898 | APOL2 | NM_145637 | 2.75 | 2.76E-04 |
| A_33_P3392077 | TP53I3 | NM_004881 | 2.76 | 6.63E-06 |
| A_33_P3405204 | LOC729911 | NR_038997 | 2.76 | 4.11E-04 |
| A_33_P3238280 | ESYT3 | NM_031913 | 2.76 | 8.68E-05 |
| A_23_P122924 | INHBA | NM_002192 | 2.76 | 4.62E-04 |
| A_23_P105862 | FRY | NM_023037 | 2.77 | 6.75E-05 |
| A_19_P00316341 | LOC100506860 | XR_108813 | 2.77 | 1.55E-04 |
| A_23_P162486 | PTPN6 | NM_002831 | 2.77 | 1.11E-04 |
| A_23_P112482 | AQP3 | NM_004925 | 2.77 | 6.53E-04 |
| A_23_P117582 | JDP2 | NM_130469 | 2.78 | 5.64E-05 |
| A_23_P29118 | DGCR5 | NR_002733 | 2.78 | 4.80E-05 |
| A_23_P82929 | NOV | NM_002514 | 2.78 | 6.92E-06 |
| A_23_P117582 | JDP2 | NM_130469 | 2.78 | 8.96E-05 |
| A_23_P4190 | ACSF2 | NM_025149 | 2.78 | 2.69E-04 |
| A_33_P3209960 | RASGRP2 | NM_153819 | 2.78 | 8.52E-06 |
| A_23_P103486 | CYP2J2 | NM_000775 | 2.78 | 1.11E-05 |
| A_33_P3409210 | ENST00000502514 | ENST00000502514 | 2.78 | 1.96E-04 |
| A_23_P122924 | INHBA | NM_002192 | 2.78 | 5.89E-04 |
| A_23_P112482 | AQP3 | NM_004925 | 2.78 | 8.53E-05 |
| A_33_P3329013 | SSTR2 | NM_001050 | 2.78 | 5.47E-04 |
| A_23_P122924 | INHBA | NM_002192 | 2.79 | 7.18E-04 |
| A_23_P421032 | SEC14L4 | NM_174977 | 2.79 | 6.47E-06 |
| A_23_P120863 | GAL3ST1 | NM_004861 | 2.79 | 1.79E-04 |
| A_23_P102391 | SLC40A1 | NM_014585 | 2.79 | 7.32E-04 |
| A_24_P557479 | XAF1 | NM_017523 | 2.79 | 1.21E-04 |
| A_21_P0013098 | XLOC_l2_013125 | ENST00000418837 | 2.80 | 5.65E-04 |
| A_23_P37127 | FOXA1 | NM_004496 | 2.80 | 9.02E-04 |
| A_21_P0012441 | TRANK1 | ENST00000463764 | 2.80 | 8.29E-04 |
| A_23_P414978 | NUDT14 | NM_177533 | 2.80 | 6.89E-05 |
| A_23_P117582 | JDP2 | NM_130469 | 2.80 | 1.59E-05 |
| A_23_P69383 | PARP9 | NM_031458 | 2.81 | 3.53E-04 |
| A_21_P0000065 | SLC1A3 | NM_001166696 | 2.81 | 2.31E-04 |
| A_33_P3336700 | SHROOM3 | NM_020859 | 2.81 | 4.18E-05 |
| A_23_P501933 | CACNG6 | NM_145814 | 2.81 | 6.62E-04 |
| A_23_P37127 | FOXA1 | NM_004496 | 2.82 | 4.61E-04 |
| A_23_P416774 | CLIC5 | NM_016929 | 2.82 | 4.32E-06 |
| A_21_P0002168 | XLOC_001342 | THC2561405 | 2.83 | 6.93E-04 |
| A_23_P103486 | CYP2J2 | NM_000775 | 2.83 | 3.98E-06 |
| A_33_P3345782 | CCDC88B | ENST00000494080 | 2.83 | 6.83E-04 |
| A_23_P12241 | MCOLN3 | NM_018298 | 2.83 | 2.25E-04 |
| A_33_P3257518 | FLJ22447 | NR_039985 | 2.84 | 1.53E-05 |
| A_32_P200238 | UCA1 | NR_015379 | 2.84 | 1.71E-04 |
| A_23_P103486 | CYP2J2 | NM_000775 | 2.84 | 1.73E-05 |
| A_21_P0003115 | XLOC_003111 | TCONS_00006505 | 2.84 | 5.06E-04 |
| A_33_P3254320 | SH2D3A | NM_005490 | 2.84 | 4.91E-05 |
| A_23_P63178 | TAF12 | NM_005644 | 2.84 | 5.87E-06 |
| A_23_P107247 | CACNA1G | NM_018896 | 2.85 | 2.83E-05 |
| A_23_P131676 | CXCR7 | NM_020311 | 2.85 | 2.72E-04 |
| A_33_P3209962 | RASGRP2 | NM_153819 | 2.85 | 5.66E-06 |
| A_33_P3254708 | ARHGAP40 | NM_001164431 | 2.85 | 2.78E-04 |
| A_21_P0000160 | TM4SF19 | NM_138461 | 2.86 | 7.56E-04 |
| A_23_P160881 | SMPDL3B | NM_001009568 | 2.86 | 1.30E-06 |
| A_21_P0006968 | SFTA1P | NR_027082 | 2.86 | 5.47E-06 |
| A_23_P114353 | NXF5 | NM_032946 | 2.86 | 9.62E-04 |
| A_23_P152655 | ICAM2 | NM_000873 | 2.86 | 6.25E-05 |
| A_23_P103486 | CYP2J2 | NM_000775 | 2.86 | 1.60E-06 |
| A_23_P103486 | CYP2J2 | NM_000775 | 2.86 | 1.39E-05 |
| A_23_P88278 | RPGRIP1 | NM_020366 | 2.87 | 1.13E-04 |
| A_23_P112220 | INSL4 | NM_002195 | 2.87 | 3.08E-04 |
| A_23_P166280 | ICOSLG | ENST00000407780 | 2.87 | 4.99E-04 |
| A_24_P125283 | HDAC5 | NM_001015053 | 2.87 | 9.52E-04 |
| A_23_P376060 | IKZF3 | NM_012481 | 2.87 | 7.90E-04 |
| A_23_P39931 | DYSF | NM_003494 | 2.88 | 2.57E-04 |
| A_21_P0012780 | XLOC_l2_011204 | ENST00000505709 | 2.88 | 1.67E-05 |
| A_23_P201863 | CDK18 | NM_212503 | 2.88 | 4.00E-04 |
| A_33_P3266744 | SYTL1 | NM_032872 | 2.88 | 8.52E-05 |
| A_33_P3340199 | ENST00000506386 | ENST00000506386 | 2.88 | 3.75E-05 |
| A_23_P117582 | JDP2 | NM_130469 | 2.88 | 8.54E-06 |
| A_24_P475349 | RAB6B | NM_016577 | 2.89 | 9.54E-04 |
| A_23_P116512 | PRR5L | NM_024841 | 2.89 | 8.04E-04 |
| A_23_P434919 | RAB42 | NM_152304 | 2.89 | 3.01E-04 |
| A_23_P129169 | CYP11A1 | NM_000781 | 2.89 | 6.56E-05 |
| A_21_P0010998 | XLOC_l2_002469 | THC2636507 | 2.89 | 1.08E-05 |
| A_23_P215484 | CCL26 | NM_006072 | 2.90 | 1.32E-04 |
| A_23_P122924 | INHBA | NM_002192 | 2.91 | 4.03E-04 |
| A_21_P0011843 | LOC729468 | BU567215 | 2.91 | 2.53E-04 |
| A_33_P3247205 | Mar1 | NM_022746 | 2.91 | 4.99E-04 |
| A_23_P57199 | GGTLC1 | NM_178311 | 2.91 | 3.34E-04 |
| A_21_P0011939 | XLOC_l2_008151 | ENST00000431244 | 2.91 | 7.84E-05 |
| A_23_P3221 | SQRDL | NM_021199 | 2.92 | 5.24E-05 |
| A_24_P329795 | C10orf10 | NM_007021 | 2.92 | 7.74E-04 |
| A_23_P46315 | DENND2C | ENST00000369540 | 2.92 | 2.77E-04 |
| A_21_P0012868 | XLOC_l2_011873 | ENST00000502514 | 2.92 | 8.14E-04 |
| A_21_P0007876 | XLOC_010061 | TCONS_00021307 | 2.92 | 3.97E-04 |
| A_23_P112482 | AQP3 | NM_004925 | 2.93 | 5.23E-04 |
| A_23_P64792 | KCNMB4 | NM_014505 | 2.93 | 5.39E-04 |
| A_33_P3290239 | DUOXA1 | EU927394 | 2.94 | 2.79E-04 |
| A_33_P3398448 | PARP10 | NM_032789 | 2.94 | 4.41E-04 |
| A_32_P33083 | VCX2 | NM_016378 | 2.94 | 9.03E-04 |
| A_23_P205746 | EML1 | NM_001008707 | 2.94 | 3.60E-05 |
| A_23_P162486 | PTPN6 | NM_002831 | 2.94 | 1.37E-04 |
| A_23_P112634 | C4orf34 | NM_174921 | 2.94 | 3.14E-04 |
| A_21_P0012872 | XLOC_l2_011873 | TCONS_l2_00022667 | 2.94 | 2.05E-04 |
| A_24_P50245 | HLA-DMA | NM_006120 | 2.94 | 4.15E-05 |
| A_23_P146134 | DUSP26 | NM_024025 | 2.95 | 2.45E-04 |
| A_23_P10873 | TLR1 | NM_003263 | 2.95 | 9.53E-05 |
| A_24_P54174 | TNFRSF1B | NM_001066 | 2.96 | 7.60E-04 |
| A_33_P3350858 | LOC100652857 | XM_003403456 | 2.96 | 7.43E-04 |
| A_23_P29975 | C4orf19 | NM_018302 | 2.97 | 4.58E-04 |
| A_21_P0004119 | XLOC_004772 | ENST00000511840 | 2.97 | 3.65E-04 |
| A_21_P0009265 | LOC100507002 | XR_109421 | 2.97 | 6.18E-07 |
| A_23_P87742 | IFFO1 | NM_001039670 | 2.98 | 9.69E-05 |
| A_33_P3376971 | CHAC1 | NM_024111 | 2.98 | 7.96E-07 |
| A_33_P3220470 | SMAD6 | NM_005585 | 2.98 | 9.97E-06 |
| A_33_P3290573 | FAM46A | NM_017633 | 2.98 | 1.90E-04 |
| A_24_P901986 | BC029255 | BC029255 | 2.98 | 5.48E-04 |
| A_24_P48069 | DOK4 | NM_018110 | 2.99 | 3.62E-04 |
| A_33_P3262635 | CECR1 | NM_177405 | 2.99 | 4.30E-06 |
| A_23_P42306 | HLA-DMA | NM_006120 | 2.99 | 5.63E-06 |
| A_23_P171336 | NXF3 | NM_022052 | 3.00 | 3.56E-04 |
| A_21_P0012437 | TRANK1 | NM_014831 | 3.00 | 8.08E-04 |
| A_21_P0000182 | LOC100507462 | NM_001242740 | 3.00 | 2.35E-04 |
| A_21_P0006969 | XLOC_008730 | TCONS_00018437 | 3.00 | 1.84E-05 |
| A_33_P3408953 | GGTLC2 | NM_199127 | 3.00 | 1.68E-04 |
| A_23_P148609 | PLAC1 | NM_021796 | 3.00 | 3.20E-04 |
| A_23_P129695 | VASN | NM_138440 | 3.01 | 4.91E-04 |
| A_23_P110412 | TMEM150C | NM_001080506 | 3.01 | 2.99E-05 |
| A_23_P142574 | MOGAT1 | NM_058165 | 3.02 | 2.94E-04 |
| A_21_P0013156 | XLOC_l2_013125 | ENST00000418837 | 3.02 | 3.48E-04 |
| A_23_P403488 | NLRP10 | NM_176821 | 3.02 | 2.49E-04 |
| A_23_P201687 | HES2 | ENST00000377836 | 3.03 | 2.91E-04 |
| A_33_P3400843 | C1QL4 | NM_001008223 | 3.03 | 6.52E-04 |
| A_33_P3244122 | HAAO | NM_012205 | 3.04 | 9.03E-05 |
| A_33_P3376965 | CHAC1 | NM_024111 | 3.05 | 1.34E-05 |
| A_23_P105862 | FRY | NM_023037 | 3.05 | 6.18E-05 |
| A_33_P3396214 | KREMEN2 | NM_172229 | 3.05 | 9.11E-05 |
| A_33_P3399363 | GPR155 | NM_001033045 | 3.05 | 4.28E-04 |
| A_23_P259071 | AREG | NM_001657 | 3.06 | 3.46E-05 |
| A_33_P3419190 | AREG | NM_001657 | 3.06 | 3.21E-05 |
| A_23_P103617 | ANXA9 | NM_003568 | 3.07 | 1.88E-04 |
| A_23_P102391 | SLC40A1 | NM_014585 | 3.07 | 3.21E-04 |
| A_21_P0001232 | XLOC_001230 | ENST00000428642 | 3.08 | 9.42E-04 |
| A_24_P158946 | FGD4 | NM_139241 | 3.08 | 3.87E-05 |
| A_23_P19020 | SNCAIP | NM_005460 | 3.08 | 9.06E-04 |
| A_21_P0001418 | XLOC_000555 | TCONS_00001268 | 3.09 | 3.14E-04 |
| A_33_P3345344 | DRGX | ENST00000374139 | 3.09 | 9.87E-04 |
| A_24_P153840 | FGD3 | NM_033086 | 3.09 | 3.68E-04 |
| A_24_P261417 | DKK3 | NM_015881 | 3.09 | 6.48E-04 |
| A_23_P301521 | KIAA1462 | NM_020848 | 3.09 | 9.77E-05 |
| A_19_P00330814 | HOTAIR | NR_003716 | 3.10 | 4.80E-04 |
| A_33_P3321657 | HSPG2 | NM_005529 | 3.10 | 4.91E-04 |
| A_23_P116512 | PRR5L | NM_024841 | 3.11 | 1.81E-04 |
| A_23_P5392 | TP53I3 | NM_004881 | 3.11 | 5.57E-06 |
| A_23_P112482 | AQP3 | NM_004925 | 3.11 | 7.89E-05 |
| A_23_P162486 | PTPN6 | NM_002831 | 3.11 | 5.32E-05 |
| A_32_P140030 | FOXN4 | NM_213596 | 3.11 | 5.37E-04 |
| A_23_P80817 | TAGLN3 | NM_013259 | 3.11 | 3.08E-06 |
| A_24_P270460 | IFI27 | NM_005532 | 3.11 | 4.60E-06 |
| A_21_P0014597 | LOC100507539 | XR_110467 | 3.11 | 2.65E-04 |
| A_33_P3323692 | ENST00000452785 | ENST00000452785 | 3.11 | 2.82E-04 |
| A_23_P116512 | PRR5L | NM_024841 | 3.12 | 2.46E-04 |
| A_21_P0008538 | XLOC_011047 | BX648502 | 3.12 | 3.37E-07 |
| A_23_P112220 | INSL4 | NM_002195 | 3.12 | 5.52E-05 |
| A_23_P103486 | CYP2J2 | NM_000775 | 3.13 | 6.47E-06 |
| A_23_P431939 | MR1 | NM_001531 | 3.13 | 2.24E-04 |
| A_21_P0001233 | XLOC_001230 | ENST00000433058 | 3.13 | 4.09E-04 |
| A_33_P3281283 | S1PR3 | NM_005226 | 3.14 | 6.16E-05 |
| A_21_P0014535 | LOC100506718 | XR_110230 | 3.14 | 6.67E-05 |
| A_32_P125771 | RGS22 | NM_015668 | 3.15 | 9.00E-04 |
| A_23_P86021 | SELENBP1 | NM_003944 | 3.15 | 3.44E-05 |
| A_23_P380857 | APOL4 | NM_030643 | 3.15 | 2.47E-04 |
| A_23_P46871 | SLC29A3 | NM_018344 | 3.16 | 9.80E-06 |
| A_21_P0008352 | XLOC_010859 | ENST00000553944 | 3.16 | 2.50E-05 |
| A_23_P333640 | PAPLN | NM_173462 | 3.16 | 1.59E-05 |
| A_23_P110412 | TMEM150C | NM_001080506 | 3.17 | 9.13E-06 |
| A_23_P68121 | PSD4 | NM_012455 | 3.17 | 3.16E-04 |
| A_23_P54116 | DAAM1 | NM_014992 | 3.17 | 1.19E-04 |
| A_21_P0008446 | XLOC_011047 | THC2533854 | 3.17 | 9.13E-04 |
| A_23_P122724 | VNN2 | NM_004665 | 3.17 | 5.33E-06 |
| A_23_P424561 | RHOV | NM_133639 | 3.17 | 6.85E-06 |
| A_23_P112220 | INSL4 | NM_002195 | 3.17 | 3.75E-04 |
| A_23_P112220 | INSL4 | NM_002195 | 3.17 | 3.96E-05 |
| A_33_P3407895 | RINL | NM_001195833 | 3.18 | 7.32E-05 |
| A_23_P8913 | CA2 | NM_000067 | 3.18 | 4.27E-04 |
| A_24_P697685 | ESYT3 | NM_031913 | 3.18 | 4.90E-04 |
| A_23_P110412 | TMEM150C | NM_001080506 | 3.18 | 8.27E-05 |
| A_23_P433855 | RGS4 | NM_005613 | 3.19 | 4.41E-05 |
| A_33_P3288839 | C14orf37 | NM_001001872 | 3.20 | 5.70E-06 |
| A_23_P153529 | TRPM4 | NM_017636 | 3.20 | 1.71E-04 |
| A_23_P17663 | MX1 | NM_002462 | 3.20 | 3.66E-06 |
| A_23_P25194 | HRK | NM_003806 | 3.20 | 1.13E-04 |
| A_23_P54291 | DUOX1 | NM_017434 | 3.20 | 8.88E-05 |
| A_21_P0012871 | XLOC_l2_011873 | ENST00000502514 | 3.20 | 1.77E-05 |
| A_33_P3268863 | C11orf44 | XR_110537 | 3.22 | 3.11E-05 |
| A_24_P317907 | SORBS1 | NM_001034954 | 3.22 | 4.12E-05 |
| A_23_P153745 | IFI30 | NM_006332 | 3.22 | 3.36E-06 |
| A_33_P3364038 | IRGM | NM_001145805 | 3.23 | 4.49E-04 |
| A_23_P314101 | SUSD2 | NM_019601 | 3.23 | 1.80E-04 |
| A_23_P112482 | AQP3 | NM_004925 | 3.23 | 2.92E-04 |
| A_33_P3408757 | ENST00000372591 | ENST00000372591 | 3.23 | 4.11E-05 |
| A_23_P63896 | FAS | NM_000043 | 3.24 | 5.08E-04 |
| A_23_P102391 | SLC40A1 | NM_014585 | 3.24 | 4.03E-04 |
| A_23_P110412 | TMEM150C | NM_001080506 | 3.24 | 7.51E-04 |
| A_33_P3235321 | SYTL1 | NM_032872 | 3.25 | 9.04E-04 |
| A_23_P106602 | CRISPLD2 | NM_031476 | 3.25 | 3.19E-04 |
| A_32_P156851 | RCAN2 | NM_005822 | 3.25 | 4.48E-05 |
| A_23_P215913 | CLU | NM_001831 | 3.26 | 4.77E-05 |
| A_23_P131683 | MAPRE3 | NM_012326 | 3.26 | 8.70E-04 |
| A_23_P112220 | INSL4 | NM_002195 | 3.26 | 6.41E-06 |
| A_23_P119040 | GREB1L | NM_001142966 | 3.27 | 2.47E-05 |
| A_21_P0013105 | XLOC_l2_013149 | ENST00000454588 | 3.27 | 9.90E-04 |
| A_23_P211680 | MLC1 | NM_015166 | 3.27 | 9.23E-04 |
| A_23_P105307 | DGKA | NM_201444 | 3.27 | 9.89E-06 |
| A_23_P121926 | SEPP1 | NM_005410 | 3.27 | 1.51E-04 |
| A_23_P380298 | ProSAPiP1 | NM_014731 | 3.27 | 3.22E-04 |
| A_33_P3232688 | ENST00000381524 | ENST00000381524 | 3.27 | 6.95E-06 |
| A_23_P215111 | ATP6V0A4 | NM_020632 | 3.28 | 4.24E-05 |
| A_23_P112482 | AQP3 | NM_004925 | 3.28 | 3.12E-05 |
| A_23_P117387 | MIA2 | NM_054024 | 3.29 | 4.60E-04 |
| A_23_P102391 | SLC40A1 | NM_014585 | 3.29 | 3.28E-04 |
| A_23_P63896 | FAS | NM_000043 | 3.30 | 3.40E-04 |
| A_23_P252052 | FILIP1L | NM_182909 | 3.30 | 1.69E-06 |
| A_23_P110412 | TMEM150C | NM_001080506 | 3.31 | 9.88E-06 |
| A_32_P107876 | FRAS1 | NM_025074 | 3.31 | 4.93E-04 |
| A_21_P0001245 | XLOC_001265 | ENST00000451766 | 3.32 | 1.40E-04 |
| A_23_P112220 | INSL4 | NM_002195 | 3.32 | 1.61E-04 |
| A_23_P304716 | HES2 | NM_019089 | 3.32 | 2.10E-04 |
| A_33_P3278362 | ANKRD2 | NM_020349 | 3.33 | 3.27E-05 |
| A_23_P124642 | RASGRP1 | NM_005739 | 3.33 | 3.30E-04 |
| A_33_P3327642 | AIM1L | NM_001039775 | 3.33 | 3.74E-04 |
| A_23_P213137 | LNX1 | NM_032622 | 3.33 | 4.27E-04 |
| A_24_P181295 | C14orf37 | NM_001001872 | 3.34 | 6.64E-05 |
| A_23_P132159 | USP18 | NM_017414 | 3.34 | 2.40E-04 |
| A_21_P0002046 | XLOC_002076 | ENST00000456467 | 3.35 | 1.23E-04 |
| A_33_P3247165 | CT62 | NM_001102658 | 3.35 | 1.22E-04 |
| A_24_P378019 | IRF7 | NM_004031 | 3.35 | 1.04E-04 |
| A_33_P3397763 | TNFSF9 | NM_003811 | 3.35 | 4.38E-05 |
| A_23_P162486 | PTPN6 | NM_002831 | 3.35 | 1.95E-05 |
| A_32_P132317 | GPR155 | NM_001033045 | 3.35 | 2.69E-04 |
| A_23_P124837 | LRP1 | NM_002332 | 3.35 | 1.72E-04 |
| A_23_P116512 | PRR5L | NM_024841 | 3.36 | 7.07E-04 |
| A_23_P121926 | SEPP1 | NM_005410 | 3.36 | 6.43E-06 |
| A_23_P116512 | PRR5L | NM_024841 | 3.36 | 2.77E-04 |
| A_33_P3422968 | DNAH6 | NM_001370 | 3.36 | 6.00E-04 |
| A_23_P63896 | FAS | NM_000043 | 3.36 | 1.10E-04 |
| A_23_P85800 | CD52 | NM_001803 | 3.36 | 2.00E-04 |
| A_23_P121926 | SEPP1 | NM_005410 | 3.37 | 1.09E-05 |
| A_23_P91910 | PLSCR4 | NM_020353 | 3.37 | 1.30E-04 |
| A_23_P361448 | SESN3 | NM_144665 | 3.38 | 4.86E-05 |
| A_23_P63896 | FAS | NM_000043 | 3.38 | 4.34E-04 |
| A_23_P78795 | MEIS3 | NM_001009813 | 3.38 | 1.16E-04 |
| A_23_P121926 | SEPP1 | NM_005410 | 3.38 | 2.98E-05 |
| A_24_P4816 | GABARAPL1 | NM_031412 | 3.38 | 1.13E-05 |
| A_23_P129005 | NYNRIN | NM_025081 | 3.38 | 9.88E-06 |
| A_33_P3358745 | SEPP1 | NM_001093726 | 3.39 | 1.17E-05 |
| A_21_P0012309 | DGCR5 | NR_002733 | 3.39 | 1.20E-04 |
| A_23_P110412 | TMEM150C | NM_001080506 | 3.39 | 1.73E-04 |
| A_23_P344531 | SYNPO | NM_007286 | 3.40 | 6.62E-04 |
| A_23_P63896 | FAS | NM_000043 | 3.40 | 6.89E-05 |
| A_23_P65518 | DACT1 | NM_016651 | 3.40 | 1.89E-05 |
| A_33_P3349299 | ENST00000377879 | ENST00000377879 | 3.40 | 3.61E-05 |
| A_21_P0013865 | XLOC_l2_015752 | ENST00000422194 | 3.40 | 1.43E-04 |
| A_23_P124837 | LRP1 | NM_002332 | 3.41 | 4.74E-04 |
| A_24_P408736 | GALNT5 | NM_014568 | 3.41 | 1.19E-04 |
| A_23_P120863 | GAL3ST1 | NM_004861 | 3.41 | 3.01E-04 |
| A_33_P3269636 | SBSN | NM_001166034 | 3.42 | 9.82E-06 |
| A_23_P74928 | MR1 | NM_001531 | 3.42 | 8.79E-04 |
| A_21_P0012336 | GGTLC1 | NM_178311 | 3.42 | 4.14E-04 |
| A_33_P3354374 | LOC100507410 | NR_040018 | 3.42 | 4.90E-04 |
| A_23_P102391 | SLC40A1 | NM_014585 | 3.43 | 1.80E-04 |
| A_33_P3381127 | FAS | NM_000043 | 3.43 | 8.68E-04 |
| A_23_P112220 | INSL4 | NM_002195 | 3.43 | 1.98E-04 |
| A_33_P3319791 | NR4A1 | NM_002135 | 3.44 | 2.93E-04 |
| A_32_P34138 | FAM25A | NM_001146157 | 3.44 | 1.10E-05 |
| A_23_P63896 | FAS | NM_000043 | 3.44 | 2.08E-04 |
| A_23_P112220 | INSL4 | NM_002195 | 3.44 | 3.92E-07 |
| A_33_P3375790 | RFPL4A | NM_001145014 | 3.45 | 6.23E-06 |
| A_23_P150609 | IGF2 | NM_000612 | 3.46 | 5.43E-06 |
| A_23_P64173 | CARD16 | NM_001017534 | 3.46 | 3.75E-04 |
| A_23_P121926 | SEPP1 | NM_005410 | 3.46 | 3.08E-05 |
| A_33_P3332112 | FAS | NM_000043 | 3.46 | 4.56E-04 |
| A_23_P4052 | ENST00000428928 | ENST00000428928 | 3.46 | 5.87E-05 |
| A_23_P112482 | AQP3 | NM_004925 | 3.47 | 1.69E-04 |
| A_23_P63896 | FAS | NM_000043 | 3.47 | 6.40E-04 |
| A_23_P52121 | PDZK1 | NM_002614 | 3.47 | 3.05E-04 |
| A_21_P0000121 | C19orf81 | NM_001195076 | 3.48 | 5.20E-04 |
| A_23_P32165 | LHX2 | NM_004789 | 3.48 | 1.08E-04 |
| A_23_P114934 | MAEL | NM_032858 | 3.48 | 6.06E-04 |
| A_23_P114934 | MAEL | NM_032858 | 3.49 | 7.17E-04 |
| A_23_P112482 | AQP3 | NM_004925 | 3.50 | 2.11E-04 |
| A_21_P0005327 | XLOC_006580 | ENST00000431071 | 3.50 | 6.13E-04 |
| A_33_P3380597 | PRR14L | NM_173566 | 3.50 | 7.67E-04 |
| A_23_P110412 | TMEM150C | NM_001080506 | 3.50 | 1.32E-05 |
| A_33_P3281710 | ENST00000399093 | ENST00000399093 | 3.51 | 3.33E-04 |
| A_23_P116512 | PRR5L | NM_024841 | 3.52 | 4.91E-04 |
| A_33_P3255929 | ENST00000432125 | ENST00000432125 | 3.52 | 2.32E-04 |
| A_21_P0010502 | HOTAIR | NR_003716 | 3.53 | 1.70E-04 |
| A_23_P114934 | MAEL | NM_032858 | 3.53 | 2.30E-04 |
| A_33_P3288832 | GPRIN1 | NM_052899 | 3.53 | 2.91E-04 |
| A_33_P3404588 | FGD4 | NM_139241 | 3.53 | 3.99E-04 |
| A_21_P0005049 | XLOC_005803 | TCONS_00012247 | 3.53 | 4.77E-04 |
| A_23_P119040 | GREB1L | NM_001142966 | 3.54 | 1.39E-04 |
| A_23_P37127 | FOXA1 | NM_004496 | 3.54 | 3.91E-05 |
| A_32_P452655 | LGALS9C | NM_001040078 | 3.54 | 1.47E-05 |
| A_23_P315451 | KIRREL2 | NM_199180 | 3.54 | 1.65E-05 |
| A_23_P118042 | LRRC36 | NM_018296 | 3.54 | 4.19E-04 |
| A_23_P6818 | SEMA3G | NM_020163 | 3.54 | 2.76E-05 |
| A_33_P3417339 | SHROOM3 | NM_020859 | 3.54 | 7.22E-06 |
| A_23_P63209 | HSD11B1 | NM_181755 | 3.54 | 6.49E-06 |
| A_33_P3265314 | FLJ44054 | NR_024609 | 3.55 | 1.10E-04 |
| A_23_P102391 | SLC40A1 | NM_014585 | 3.55 | 3.85E-04 |
| A_23_P16523 | GDF15 | NM_004864 | 3.55 | 3.79E-04 |
| A_33_P3252286 | CRLF1 | NM_004750 | 3.55 | 2.40E-06 |
| A_23_P124837 | LRP1 | NM_002332 | 3.55 | 7.71E-05 |
| A_23_P124837 | LRP1 | NM_002332 | 3.56 | 2.32E-04 |
| A_23_P121926 | SEPP1 | NM_005410 | 3.56 | 9.79E-05 |
| A_21_P0007352 | XLOC_009437 | TCONS_00019635 | 3.57 | 7.69E-04 |
| A_23_P87238 | SAA4 | NM_006512 | 3.58 | 9.02E-04 |
| A_33_P3398331 | MMP24 | NM_006690 | 3.58 | 6.02E-04 |
| A_21_P0001616 | XLOC_001188 | TCONS_00001799 | 3.58 | 2.06E-04 |
| A_23_P112220 | INSL4 | NM_002195 | 3.58 | 1.93E-06 |
| A_23_P311895 | CLIC5 | NM_016929 | 3.58 | 9.71E-05 |
| A_23_P160886 | TAS1R1 | NM_138697 | 3.59 | 7.91E-04 |
| A_32_P339640 | C20orf197 | NM_173644 | 3.59 | 8.09E-05 |
| A_23_P49060 | SPINT1 | NM_181642 | 3.60 | 1.40E-04 |
| A_23_P122724 | VNN2 | NM_004665 | 3.60 | 5.74E-05 |
| A_23_P63896 | FAS | NM_000043 | 3.60 | 4.26E-04 |
| A_23_P122724 | VNN2 | NM_004665 | 3.60 | 2.05E-05 |
| A_33_P3363260 | PGM2L1 | NM_173582 | 3.60 | 6.47E-05 |
| A_19_P00316340 | LOC100506860 | XR_108813 | 3.60 | 4.59E-05 |
| A_23_P53176 | FOLR1 | NM_016725 | 3.60 | 2.84E-05 |
| A_23_P81590 | PDE6A | NM_000440 | 3.61 | 2.23E-04 |
| A_33_P3306823 | ZNF846 | NM_001077624 | 3.61 | 7.26E-04 |
| A_23_P112220 | INSL4 | NM_002195 | 3.61 | 4.65E-06 |
| A_23_P122724 | VNN2 | NM_004665 | 3.61 | 1.53E-04 |
| A_23_P112482 | AQP3 | NM_004925 | 3.61 | 7.51E-04 |
| A_23_P139585 | PDE1B | NM_000924 | 3.61 | 5.88E-04 |
| A_33_P3268487 | ABAT | NM_000663 | 3.61 | 1.21E-04 |
| A_33_P3290040 | CPT1C | NM_001199752 | 3.62 | 6.91E-05 |
| A_33_P3393170 | CAPN5 | NM_004055 | 3.62 | 1.06E-04 |
| A_23_P106602 | CRISPLD2 | NM_031476 | 3.63 | 5.05E-04 |
| A_21_P0011751 | CD177 | NM_020406 | 3.64 | 8.88E-04 |
| A_23_P250102 | CAND2 | NM_012298 | 3.65 | 5.60E-05 |
| A_33_P3812669 | GABARAPL1 | NM_031412 | 3.65 | 1.69E-05 |
| A_23_P124837 | LRP1 | NM_002332 | 3.65 | 3.15E-04 |
| A_21_P0005727 | XLOC_006850 | ENST00000518732 | 3.65 | 3.70E-04 |
| A_23_P34233 | QPRT | NM_014298 | 3.66 | 6.52E-04 |
| A_23_P118042 | LRRC36 | NM_018296 | 3.67 | 9.79E-05 |
| A_23_P45999 | FBXO2 | NM_012168 | 3.67 | 1.29E-04 |
| A_23_P120863 | GAL3ST1 | NM_004861 | 3.67 | 4.93E-04 |
| A_33_P3299220 | ADAMTSL4 | NM_025008 | 3.67 | 7.48E-06 |
| A_23_P115011 | ADAMTSL4 | NM_019032 | 3.67 | 8.11E-05 |
| A_23_P154526 | GRB14 | NM_004490 | 3.69 | 2.30E-04 |
| A_23_P15450 | TMEM100 | NM_018286 | 3.69 | 7.75E-04 |
| A_33_P3235262 | PIP5KL1 | NM_001135219 | 3.71 | 8.73E-05 |
| A_21_P0006028 | XLOC_007458 | ENST00000436942 | 3.71 | 2.97E-05 |
| A_21_P0014384 | LOC100505701 | XR_109542 | 3.72 | 6.14E-04 |
| A_23_P303833 | SCN4B | NM_174934 | 3.72 | 9.23E-04 |
| A_23_P118042 | LRRC36 | NM_018296 | 3.72 | 4.09E-04 |
| A_23_P121926 | SEPP1 | NM_005410 | 3.73 | 7.56E-05 |
| A_21_P0002356 | XLOC_001870 | TCONS_00004032 | 3.73 | 5.63E-04 |
| A_23_P121926 | SEPP1 | NM_005410 | 3.73 | 3.10E-05 |
| A_23_P61371 | TMEM173 | NM_198282 | 3.73 | 3.43E-06 |
| A_23_P23346 | MLLT11 | NM_006818 | 3.73 | 1.66E-05 |
| A_32_P74409 | C11orf96 | NM_001145033 | 3.73 | 1.39E-06 |
| A_23_P118042 | LRRC36 | NM_018296 | 3.74 | 3.92E-04 |
| A_23_P360797 | NTF3 | NM_002527 | 3.75 | 1.01E-05 |
| A_23_P54144 | BMP4 | NM_001202 | 3.75 | 3.49E-04 |
| A_23_P122724 | VNN2 | NM_004665 | 3.75 | 1.59E-04 |
| A_23_P124837 | LRP1 | NM_002332 | 3.75 | 1.68E-04 |
| A_23_P74309 | NOS1AP | NM_014697 | 3.75 | 7.43E-04 |
| A_24_P390060 | IQCD | NM_138451 | 3.76 | 4.13E-04 |
| A_23_P435636 | DAND5 | NM_152654 | 3.76 | 1.15E-04 |
| A_23_P343398 | CCR7 | NM_001838 | 3.77 | 1.95E-04 |
| A_33_P3368695 | HES7 | NM_001165967 | 3.77 | 9.17E-04 |
| A_33_P3366127 | RFPL4A | NM_001145014 | 3.77 | 1.06E-05 |
| A_21_P0012373 | XLOC_l2_009658 | ENST00000450746 | 3.78 | 2.82E-05 |
| A_23_P118042 | LRRC36 | NM_018296 | 3.78 | 3.69E-04 |
| A_33_P3349637 | PCDH1 | NM_002587 | 3.78 | 6.75E-04 |
| A_23_P63896 | FAS | NM_000043 | 3.79 | 1.50E-04 |
| A_23_P121926 | SEPP1 | NM_005410 | 3.80 | 1.72E-05 |
| A_33_P3843873 | ESRG | NR_027122 | 3.81 | 1.95E-04 |
| A_23_P324327 | GPRC5B | NM_016235 | 3.82 | 2.43E-05 |
| A_33_P3371999 | TPPP | NM_007030 | 3.82 | 8.02E-04 |
| A_23_P122724 | VNN2 | NM_004665 | 3.83 | 1.68E-06 |
| A_33_P3307337 | MAPK4 | NM_002747 | 3.83 | 4.22E-04 |
| A_23_P121926 | SEPP1 | NM_005410 | 3.84 | 1.24E-05 |
| A_23_P122724 | VNN2 | NM_004665 | 3.85 | 5.88E-04 |
| A_24_P59667 | JAK3 | NM_000215 | 3.86 | 2.19E-04 |
| A_23_P120863 | GAL3ST1 | NM_004861 | 3.87 | 3.37E-05 |
| A_23_P33984 | TMEM27 | NM_020665 | 3.87 | 2.90E-05 |
| A_23_P124837 | LRP1 | NM_002332 | 3.88 | 9.02E-05 |
| A_32_P78681 | GLP2R | ENST00000262441 | 3.88 | 2.88E-04 |
| A_23_P118042 | LRRC36 | NM_018296 | 3.90 | 2.01E-04 |
| A_23_P325690 | ANKRD35 | NM_144698 | 3.90 | 9.00E-04 |
| A_23_P130027 | EPN3 | NM_017957 | 3.91 | 1.42E-04 |
| A_33_P3253792 | ENST00000342995 | ENST00000342995 | 3.91 | 1.16E-04 |
| A_23_P94338 | ENPP2 | NM_006209 | 3.92 | 4.95E-05 |
| A_23_P122724 | VNN2 | NM_004665 | 3.92 | 9.09E-05 |
| A_23_P160559 | ECM1 | NM_004425 | 3.93 | 3.38E-06 |
| A_23_P256312 | MST1R | NM_002447 | 3.93 | 1.23E-04 |
| A_23_P212830 | FGFR3 | NM_000142 | 3.94 | 4.20E-05 |
| A_23_P122724 | VNN2 | NM_004665 | 3.94 | 1.82E-05 |
| A_23_P350005 | TRIML2 | NM_173553 | 3.96 | 1.75E-05 |
| A_21_P0014087 | LOC100506405 | ENST00000455754 | 3.96 | 3.99E-04 |
| A_33_P3396139 | CTLA4 | NM_005214 | 3.96 | 7.17E-04 |
| A_21_P0011007 | LOC283116 | NM_001206626 | 3.96 | 1.82E-04 |
| A_33_P3293446 | KIAA1462 | NM_020848 | 3.97 | 2.25E-05 |
| A_23_P360964 | DACT3 | NM_145056 | 3.97 | 5.01E-07 |
| A_33_P3262729 | MYH8 | NM_002472 | 3.97 | 3.68E-04 |
| A_23_P106024 | JAG2 | NM_002226 | 3.97 | 2.64E-04 |
| A_23_P88351 | ATL1 | NM_181598 | 3.97 | 1.13E-05 |
| A_23_P414654 | RAB37 | NM_175738 | 3.98 | 8.94E-05 |
| A_23_P215720 | CFTR | NM_000492 | 3.98 | 3.63E-04 |
| A_23_P8640 | GPER | NM_001039966 | 4.00 | 3.48E-04 |
| A_23_P143559 | CLTCL1 | NM_007098 | 4.00 | 9.02E-05 |
| A_23_P502464 | NOS2 | NM_000625 | 4.00 | 6.94E-05 |
| A_23_P501985 | CSF2RA | NM_172249 | 4.00 | 3.37E-05 |
| A_21_P0011069 | LOC100506691 | XR_109143 | 4.01 | 2.12E-05 |
| A_21_P0007586 | XLOC_009810 | ENST00000553247 | 4.01 | 9.24E-06 |
| A_23_P119478 | EBI3 | NM_005755 | 4.03 | 4.22E-05 |
| A_23_P2492 | C1S | NM_001734 | 4.03 | 9.88E-04 |
| A_23_P106024 | JAG2 | NM_002226 | 4.03 | 7.06E-04 |
| A_23_P106024 | JAG2 | NM_002226 | 4.03 | 2.64E-04 |
| A_23_P23947 | MAP3K8 | NM_005204 | 4.03 | 1.17E-05 |
| A_33_P3263867 | P2RX7 | NM_002562 | 4.04 | 3.56E-04 |
| A_23_P342000 | RBM11 | NM_144770 | 4.05 | 1.17E-04 |
| A_33_P3392391 | CPT1C | NM_001199752 | 4.05 | 7.60E-04 |
| A_23_P145874 | SAMD9L | NM_152703 | 4.05 | 1.99E-04 |
| A_23_P103617 | ANXA9 | NM_003568 | 4.07 | 5.75E-04 |
| A_23_P126836 | TNFSF4 | NM_003326 | 4.08 | 1.49E-04 |
| A_33_P3361636 | MGP | NM_001190839 | 4.08 | 7.90E-04 |
| A_32_P52785 | DAAM2 | NM_015345 | 4.09 | 2.47E-05 |
| A_24_P787947 | YPEL2 | NM_001005404 | 4.10 | 2.34E-04 |
| A_23_P258088 | PACSIN1 | NM_020804 | 4.10 | 2.74E-04 |
| A_23_P88559 | LIPC | NM_000236 | 4.11 | 2.52E-04 |
| A_21_P0009771 | XLOC_013364 | TCONS_00027380 | 4.12 | 1.35E-04 |
| A_33_P3709327 | LOC100144602 | NR_034138 | 4.13 | 2.96E-06 |
| A_33_P3215883 | FLT1 | NM_002019 | 4.13 | 9.84E-04 |
| A_23_P37088 | RDH12 | NM_152443 | 4.15 | 7.83E-04 |
| A_23_P163455 | MAP1A | NM_002373 | 4.17 | 3.26E-05 |
| A_23_P112289 | TMOD1 | NM_003275 | 4.17 | 1.83E-04 |
| A_23_P112554 | COL15A1 | NM_001855 | 4.18 | 3.12E-04 |
| A_33_P3271711 | ENST00000449914 | ENST00000449914 | 4.19 | 1.60E-04 |
| A_23_P21976 | CSPG4 | NM_001897 | 4.20 | 5.46E-06 |
| A_23_P344884 | CARNS1 | NM_001166222 | 4.20 | 2.89E-04 |
| A_33_P3345816 | GPER | NM_001039966 | 4.20 | 3.79E-05 |
| A_23_P112554 | COL15A1 | NM_001855 | 4.21 | 9.14E-05 |
| A_23_P319598 | C4BPB | NM_000716 | 4.22 | 3.05E-05 |
| A_23_P323761 | TRAF3IP3 | NM_025228 | 4.22 | 7.13E-04 |
| A_33_P3210762 | ADAMTS7 | NM_014272 | 4.23 | 9.83E-06 |
| A_23_P106024 | JAG2 | NM_002226 | 4.23 | 8.92E-04 |
| A_19_P00331623 | XIST | NR_001564 | 4.26 | 4.24E-06 |
| A_24_P89457 | CDKN1A | NM_078467 | 4.26 | 2.67E-06 |
| A_21_P0011723 | XLOC_l2_006937 | TCONS_l2_00012888 | 4.28 | 6.05E-04 |
| A_23_P133408 | CSF2 | NM_000758 | 4.29 | 4.05E-04 |
| A_23_P150053 | ACTA2 | NM_001613 | 4.29 | 1.78E-04 |
| A_33_P3330911 | BCAS1 | NM_003657 | 4.31 | 3.25E-04 |
| A_33_P3367860 | CHRM1 | NM_000738 | 4.31 | 7.48E-05 |
| A_23_P422933 | ARHGAP20 | NM_020809 | 4.33 | 3.39E-04 |
| A_21_P0014218 | LOC100507186 | XR_108901 | 4.33 | 3.32E-04 |
| A_21_P0003917 | XLOC_004207 | CR936814 | 4.34 | 9.81E-04 |
| A_23_P106024 | JAG2 | NM_002226 | 4.34 | 1.58E-04 |
| A_23_P15101 | TMC5 | NM_024780 | 4.34 | 2.04E-04 |
| A_33_P3386671 | RORC | NM_005060 | 4.34 | 4.33E-04 |
| A_23_P106024 | JAG2 | NM_002226 | 4.34 | 2.62E-04 |
| A_23_P119478 | EBI3 | NM_005755 | 4.37 | 6.82E-05 |
| A_23_P118042 | LRRC36 | NM_018296 | 4.37 | 1.06E-04 |
| A_23_P112554 | COL15A1 | NM_001855 | 4.40 | 1.24E-04 |
| A_33_P3327041 | LOC100128054 | NR_033969 | 4.42 | 2.22E-04 |
| A_33_P3268838 | CPEB1 | NM_030594 | 4.42 | 1.31E-04 |
| A_32_P337442 | C10orf67 | NM_153714 | 4.43 | 4.24E-04 |
| A_21_P0009460 | XLOC_012632 | TCONS_00026290 | 4.43 | 4.97E-04 |
| A_21_P0008517 | LOC100506476 | XR_110221 | 4.44 | 7.81E-05 |
| A_23_P119478 | EBI3 | NM_005755 | 4.44 | 6.67E-06 |
| A_23_P106024 | JAG2 | NM_002226 | 4.45 | 3.26E-04 |
| A_23_P1575 | TRIM49 | NM_020358 | 4.45 | 5.18E-06 |
| A_21_P0014610 | LOC100506941 | XR_110521 | 4.46 | 1.41E-04 |
| A_33_P3269924 | HIP1R | ENST00000535831 | 4.48 | 1.54E-04 |
| A_23_P349416 | ERBB3 | NM_001982 | 4.48 | 5.10E-04 |
| A_32_P234145 | SHC4 | NM_203349 | 4.52 | 3.05E-07 |
| A_21_P0005616 | XLOC_006863 | ENST00000522365 | 4.53 | 1.39E-04 |
| A_23_P87879 | CD69 | NM_001781 | 4.55 | 5.98E-04 |
| A_23_P112798 | CRIP2 | NM_001312 | 4.55 | 4.11E-05 |
| A_23_P118042 | LRRC36 | NM_018296 | 4.56 | 4.51E-04 |
| A_33_P3384287 | PALM | NM_002579 | 4.57 | 8.73E-05 |
| A_23_P161218 | ANKRD1 | NM_014391 | 4.57 | 3.27E-04 |
| A_24_P3005 | SCN9A | NM_002977 | 4.57 | 3.58E-04 |
| A_23_P112554 | COL15A1 | NM_001855 | 4.58 | 6.40E-04 |
| A_23_P122724 | VNN2 | NM_004665 | 4.58 | 4.74E-05 |
| A_23_P119478 | EBI3 | NM_005755 | 4.60 | 1.79E-05 |
| A_23_P121795 | SORBS2 | NM_021069 | 4.60 | 2.83E-05 |
| A_23_P121795 | SORBS2 | NM_021069 | 4.61 | 1.40E-05 |
| A_33_P3406661 | TMEM63C | NM_020431 | 4.64 | 1.13E-04 |
| A_23_P254165 | RAI2 | NM_021785 | 4.64 | 5.96E-05 |
| A_23_P135990 | SLCO2A1 | NM_005630 | 4.65 | 9.56E-05 |
| A_23_P118042 | LRRC36 | NM_018296 | 4.67 | 2.67E-05 |
| A_23_P121795 | SORBS2 | NM_021069 | 4.67 | 1.37E-04 |
| A_23_P119478 | EBI3 | NM_005755 | 4.67 | 8.41E-06 |
| A_23_P310460 | MDGA1 | NM_153487 | 4.68 | 1.63E-05 |
| A_23_P121795 | SORBS2 | NM_021069 | 4.68 | 2.89E-05 |
| A_23_P42909 | TMEM139 | NM_153345 | 4.70 | 1.83E-05 |
| A_23_P112554 | COL15A1 | NM_001855 | 4.70 | 4.03E-04 |
| A_33_P3248163 | TRIM43 | NM_138800 | 4.70 | 5.44E-04 |
| A_23_P119478 | EBI3 | NM_005755 | 4.71 | 8.27E-06 |
| A_21_P0011401 | XLOC_l2_004611 | BC037839 | 4.73 | 1.57E-04 |
| A_23_P120227 | LBH | NM_030915 | 4.73 | 1.71E-05 |
| A_23_P72668 | SDPR | NM_004657 | 4.75 | 3.92E-05 |
| A_23_P121795 | SORBS2 | NM_021069 | 4.76 | 8.49E-05 |
| A_23_P121795 | SORBS2 | NM_021069 | 4.77 | 1.63E-04 |
| A_23_P170050 | RIT2 | NM_002930 | 4.78 | 2.07E-04 |
| A_24_P595717 | FAM179A | NM_199280 | 4.78 | 2.79E-04 |
| A_23_P119478 | EBI3 | NM_005755 | 4.79 | 4.35E-05 |
| A_23_P121795 | SORBS2 | NM_021069 | 4.79 | 3.11E-05 |
| A_21_P0013062 | XLOC_l2_012871 | ENST00000431401 | 4.82 | 5.92E-05 |
| A_23_P119478 | EBI3 | NM_005755 | 4.82 | 6.16E-05 |
| A_32_P222695 | ARHGEF37 | NM_001001669 | 4.83 | 9.39E-05 |
| A_23_P94230 | LY96 | NM_015364 | 4.83 | 4.25E-04 |
| A_23_P309837 | STON2 | NM_033104 | 4.83 | 3.12E-04 |
| A_32_P46571 | RHBDL2 | NM_017821 | 4.85 | 4.34E-04 |
| A_23_P169017 | DEFB103B | NM_018661 | 4.86 | 1.49E-04 |
| A_23_P121795 | SORBS2 | NM_021069 | 4.86 | 9.54E-05 |
| A_23_P122216 | LOX | NM_002317 | 4.88 | 7.32E-05 |
| A_23_P121795 | SORBS2 | NM_021069 | 4.89 | 1.70E-04 |
| A_21_P0011719 | XLOC_l2_006902 | TCONS_l2_00012839 | 4.90 | 1.99E-04 |
| A_23_P119478 | EBI3 | NM_005755 | 4.90 | 1.79E-05 |
| A_23_P121795 | SORBS2 | NM_021069 | 4.91 | 1.40E-04 |
| A_24_P315056 | C10orf122 | NM_001128202 | 4.91 | 2.84E-04 |
| A_23_P386268 | C4orf26 | NM_178497 | 4.92 | 3.55E-05 |
| A_33_P3338698 | IHH | NM_002181 | 4.93 | 3.73E-05 |
| A_23_P122216 | LOX | NM_002317 | 4.93 | 5.57E-05 |
| A_24_P140475 | SORBS2 | NM_021069 | 4.93 | 5.91E-04 |
| A_21_P0004659 | XLOC_005341 | ENST00000418403 | 4.93 | 9.18E-04 |
| A_21_P0001366 | XLOC_000358 | TCONS_00001113 | 4.94 | 4.35E-04 |
| A_23_P59452 | ABP1 | NM_001091 | 4.94 | 9.83E-05 |
| A_23_P502464 | NOS2 | NM_000625 | 4.95 | 1.01E-04 |
| A_23_P500501 | FGFR3 | NM_000142 | 4.95 | 1.65E-05 |
| A_24_P203000 | IL2RB | NM_000878 | 4.99 | 1.40E-04 |
| A_33_P3329974 | CGN | NM_020770 | 4.99 | 1.92E-05 |
| A_23_P122216 | LOX | NM_002317 | 5.02 | 1.62E-04 |
| A_19_P00320722 | LOC100131089 | NR_040059 | 5.05 | 7.45E-04 |
| A_23_P215720 | CFTR | NM_000492 | 5.08 | 1.82E-04 |
| A_23_P215720 | CFTR | NM_000492 | 5.09 | 1.80E-04 |
| A_23_P119478 | EBI3 | NM_005755 | 5.09 | 1.93E-05 |
| A_23_P23074 | IFI44 | NM_006417 | 5.12 | 8.86E-05 |
| A_23_P121120 | GPR87 | NM_023915 | 5.12 | 1.11E-04 |
| A_33_P3348239 | FBN1 | NM_000138 | 5.12 | 2.51E-06 |
| A_33_P3364268 | LBH | NM_030915 | 5.12 | 4.21E-04 |
| A_23_P33326 | ADRA1B | NM_000679 | 5.13 | 3.31E-05 |
| A_23_P122216 | LOX | NM_002317 | 5.14 | 2.45E-04 |
| A_21_P0014111 | LOC100506708 | ENST00000490351 | 5.15 | 2.04E-04 |
| A_23_P57784 | CLDN1 | NM_021101 | 5.16 | 6.45E-06 |
| A_23_P122216 | LOX | NM_002317 | 5.18 | 7.08E-05 |
| A_23_P122216 | LOX | NM_002317 | 5.18 | 7.09E-05 |
| A_19_P00329511 | XIST | NR_001564 | 5.19 | 8.99E-05 |
| A_24_P17870 | HCP5 | NR_040662 | 5.19 | 3.31E-05 |
| A_23_P421401 | PDGFRB | NM_002609 | 5.21 | 6.64E-04 |
| A_24_P349196 | CCDC30 | NM_001080850 | 5.21 | 9.18E-04 |
| A_23_P122216 | LOX | NM_002317 | 5.27 | 8.28E-05 |
| A_23_P29773 | LAMP3 | NM_014398 | 5.29 | 1.82E-04 |
| A_21_P0011070 | XLOC_l2_003188 | BI114595 | 5.30 | 6.52E-05 |
| A_23_P104798 | IL18 | NM_001562 | 5.30 | 3.09E-05 |
| A_23_P87238 | SAA4 | NM_006512 | 5.33 | 3.37E-04 |
| A_33_P3358943 | GRM2 | NM_000839 | 5.33 | 7.84E-04 |
| A_23_P45560 | GPR143 | NM_000273 | 5.35 | 8.42E-04 |
| A_24_P190472 | SLPI | NM_003064 | 5.38 | 3.31E-04 |
| A_23_P167030 | PTH1R | NM_000316 | 5.39 | 1.06E-04 |
| A_21_P0008692 | XLOC_011379 | AL109706 | 5.39 | 7.95E-04 |
| A_23_P122216 | LOX | NM_002317 | 5.43 | 8.69E-05 |
| A_23_P122216 | LOX | NM_002317 | 5.47 | 3.45E-05 |
| A_21_P0008293 | XLOC_011028 | ENST00000418927 | 5.49 | 4.18E-04 |
| A_23_P104798 | IL18 | NM_001562 | 5.51 | 4.80E-06 |
| A_23_P120227 | LBH | NM_030915 | 5.54 | 1.09E-05 |
| A_23_P51213 | MYOM3 | NM_152372 | 5.55 | 2.85E-04 |
| A_23_P62607 | IL22RA1 | NM_021258 | 5.57 | 9.59E-04 |
| A_23_P502464 | NOS2 | NM_000625 | 5.59 | 7.53E-04 |
| A_23_P120227 | LBH | NM_030915 | 5.59 | 1.03E-04 |
| A_23_P104798 | IL18 | NM_001562 | 5.59 | 7.31E-06 |
| A_23_P122216 | LOX | NM_002317 | 5.59 | 4.11E-05 |
| A_21_P0011781 | PSG8 | NM_001130167 | 5.60 | 3.09E-04 |
| A_23_P80048 | FER1L4 | NR_024377 | 5.64 | 9.09E-05 |
| A_23_P104798 | IL18 | NM_001562 | 5.65 | 8.24E-05 |
| A_23_P139786 | OASL | NM_003733 | 5.67 | 9.49E-04 |
| A_23_P215720 | CFTR | NM_000492 | 5.68 | 9.18E-05 |
| A_23_P104798 | IL18 | NM_001562 | 5.68 | 1.81E-05 |
| A_23_P104798 | IL18 | NM_001562 | 5.69 | 1.58E-06 |
| A_23_P104798 | IL18 | NM_001562 | 5.69 | 2.99E-05 |
| A_33_P3283611 | IFIT3 | NM_001549 | 5.70 | 1.31E-04 |
| A_23_P329962 | SUN3 | NM_001030019 | 5.72 | 5.42E-05 |
| A_23_P426021 | SEL1L3 | NM_015187 | 5.72 | 2.86E-04 |
| A_23_P114883 | FMOD | NM_002023 | 5.72 | 4.45E-05 |
| A_23_P150198 | LOC440040 | NR_027044 | 5.74 | 1.24E-04 |
| A_33_P3373960 | RD3 | NM_183059 | 5.74 | 2.19E-04 |
| A_23_P206359 | CDH1 | NM_004360 | 5.76 | 7.74E-04 |
| A_23_P120227 | LBH | NM_030915 | 5.76 | 1.16E-05 |
| A_32_P25357 | CDH15 | NM_004933 | 5.77 | 7.95E-05 |
| A_23_P2283 | TAC3 | NM_013251 | 5.79 | 4.85E-05 |
| A_23_P104798 | IL18 | NM_001562 | 5.80 | 1.13E-05 |
| A_23_P120227 | LBH | NM_030915 | 5.83 | 5.13E-05 |
| A_24_P139665 | HPCAL4 | NM_016257 | 5.86 | 5.71E-04 |
| A_24_P286951 | C10orf81 | NM_024889 | 5.86 | 3.28E-04 |
| A_23_P344555 | NEDD9 | NM_006403 | 5.86 | 1.44E-05 |
| A_21_P0002043 | XLOC_002075 | ENST00000437916 | 5.89 | 3.69E-05 |
| A_23_P120227 | LBH | NM_030915 | 5.89 | 6.43E-05 |
| A_23_P104798 | IL18 | NM_001562 | 5.89 | 3.19E-05 |
| A_21_P0006934 | XLOC_008659 | TCONS_00018381 | 5.90 | 1.45E-04 |
| A_23_P351148 | SH2D1B | NM_053282 | 5.91 | 2.44E-04 |
| A_23_P202427 | HKDC1 | NM_025130 | 5.92 | 5.20E-05 |
| A_23_P120227 | LBH | NM_030915 | 5.96 | 1.28E-05 |
| A_33_P3341686 | XIST | NR_001564 | 5.96 | 3.43E-04 |
| A_24_P270728 | NUPR1 | NM_001042483 | 5.96 | 1.28E-04 |
| A_23_P215720 | CFTR | NM_000492 | 5.97 | 4.66E-04 |
| A_33_P3264846 | SAMD9L | NM_152703 | 5.99 | 1.96E-04 |
| A_33_P3319542 | SLC8A2 | NM_015063 | 6.02 | 2.72E-05 |
| A_23_P120227 | LBH | NM_030915 | 6.08 | 1.26E-05 |
| A_23_P120227 | LBH | NM_030915 | 6.08 | 2.51E-05 |
| A_21_P0012598 | XLOC_l2_010724 | TCONS_l2_00020599 | 6.23 | 5.36E-04 |
| A_21_P0014889 | LOC100507431 | ENST00000533434 | 6.24 | 1.01E-04 |
| A_23_P436369 | FILIP1 | NM_015687 | 6.25 | 4.54E-04 |
| A_23_P217379 | COL4A6 | NM_033641 | 6.26 | 1.60E-04 |
| A_24_P318656 | ITGB3 | NM_000212 | 6.26 | 2.60E-06 |
| A_21_P0014441 | LOC100507233 | XR_109824 | 6.31 | 3.03E-06 |
| A_23_P206359 | CDH1 | NM_004360 | 6.33 | 4.89E-05 |
| A_24_P392110 | PSG8 | NM_182707 | 6.34 | 9.01E-06 |
| A_23_P500000 | SCEL | NM_144777 | 6.36 | 2.06E-04 |
| A_33_P3312119 | C6orf99 | NM_001195032 | 6.37 | 2.04E-05 |
| A_21_P0008768 | XLOC_011559 | BG208552 | 6.38 | 5.19E-04 |
| A_23_P104798 | IL18 | NM_001562 | 6.42 | 2.85E-06 |
| A_23_P114883 | FMOD | NM_002023 | 6.43 | 6.50E-05 |
| A_23_P215720 | CFTR | NM_000492 | 6.44 | 5.56E-04 |
| A_33_P3227400 | COL4A4 | NM_000092 | 6.53 | 7.89E-05 |
| A_33_P3313145 | ITIH3 | NM_002217 | 6.56 | 1.57E-04 |
| A_23_P36531 | TSPAN8 | NM_004616 | 6.58 | 4.77E-04 |
| A_23_P143935 | PIGZ | NM_025163 | 6.59 | 4.94E-05 |
| A_23_P120227 | LBH | NM_030915 | 6.60 | 9.04E-07 |
| A_23_P52266 | IFIT1 | NM_001548 | 6.62 | 4.21E-06 |
| A_23_P26994 | GNGT2 | NM_031498 | 6.65 | 1.69E-06 |
| A_23_P212469 | ENTPD3 | NM_001248 | 6.68 | 2.40E-05 |
| A_21_P0007497 | LOC100506465 | XR_109133 | 6.69 | 5.84E-06 |
| A_23_P84219 | LIPH | NM_139248 | 6.72 | 2.80E-06 |
| A_23_P114883 | FMOD | NM_002023 | 6.81 | 1.02E-04 |
| A_33_P3374970 | LOC399939 | NM_001206627 | 6.87 | 2.10E-04 |
| A_23_P4899 | NTF4 | NM_006179 | 6.87 | 7.90E-05 |
| A_23_P398854 | DOK7 | NM_173660 | 6.92 | 3.08E-04 |
| A_21_P0014570 | LOC100505814 | ENST00000547027 | 6.98 | 8.66E-04 |
| A_23_P371729 | GJA5 | NM_005266 | 7.00 | 1.17E-04 |
| A_23_P66881 | RGS9 | NM_003835 | 7.08 | 6.28E-04 |
| A_21_P0002781 | XLOC_002871 | ENST00000498005 | 7.12 | 4.15E-04 |
| A_33_P3292769 | NFAM1 | NM_145912 | 7.13 | 9.25E-04 |
| A_24_P120907 | PGM5 | NM_021965 | 7.22 | 4.33E-04 |
| A_23_P148088 | FGG | NM_000509 | 7.30 | 9.15E-05 |
| A_33_P3286273 | EBLN1 | NM_001199938 | 7.31 | 3.04E-04 |
| A_21_P0009341 | LOC645638 | NR_030732 | 7.37 | 1.15E-05 |
| A_33_P3449097 | TSPAN10 | NM_031945 | 7.37 | 3.49E-04 |
| A_23_P340848 | PTGIR | NM_000960 | 7.39 | 3.66E-05 |
| A_33_P3243702 | KLHL30 | NM_198582 | 7.42 | 2.18E-04 |
| A_24_P395814 | CGB | NM_000737 | 7.47 | 1.33E-05 |
| A_32_P83049 | EFR3B | NM_014971 | 7.56 | 6.01E-05 |
| A_33_P3234472 | LOC284751 | NR_034124 | 7.61 | 3.08E-05 |
| A_23_P47924 | PTPRR | NM_002849 | 7.62 | 7.57E-04 |
| A_33_P3222762 | HULC | NR_004855 | 7.65 | 7.49E-04 |
| A_33_P3384133 | C17orf99 | NM_001163075 | 7.65 | 3.83E-04 |
| A_24_P937325 | FGD4 | ENST00000395742 | 7.70 | 6.11E-04 |
| A_21_P0001429 | XLOC_000587 | ENST00000448264 | 7.70 | 8.61E-04 |
| A_32_P105549 | ANXA8L2 | NM_001630 | 7.72 | 1.79E-05 |
| A_23_P88678 | C15orf27 | NM_152335 | 7.79 | 5.96E-04 |
| A_32_P473302 | FLJ35024 | NR_015375 | 7.86 | 6.25E-04 |
| A_33_P3232273 | THC2600547 | THC2600547 | 7.93 | 2.10E-04 |
| A_21_P0000624 | SLC6A13 | NM_001243392 | 8.07 | 1.78E-07 |
| A_33_P3268129 | ENST00000367356 | ENST00000367356 | 8.13 | 1.01E-04 |
| A_33_P3241269 | CES1 | NM_001025195 | 8.17 | 3.89E-04 |
| A_23_P157736 | PPAPDC3 | NM_032728 | 8.18 | 3.35E-04 |
| A_23_P2674 | KRT4 | NM_002272 | 8.18 | 6.18E-06 |
| A_33_P3782529 | LOC553103 | NR_037898 | 8.22 | 6.30E-05 |
| A_33_P3332999 | THC2600547 | THC2600547 | 8.26 | 3.37E-04 |
| A_23_P87879 | CD69 | NM_001781 | 8.34 | 3.58E-04 |
| A_23_P19754 | CPA4 | NM_016352 | 8.43 | 1.40E-06 |
| A_33_P3293918 | SH2D3C | NM_170600 | 8.50 | 5.35E-04 |
| A_21_P0013061 | XLOC_l2_012871 | TCONS_l2_00024609 | 8.55 | 2.77E-04 |
| A_24_P251734 | FSTL5 | NM_020116 | 8.65 | 8.86E-05 |
| A_33_P3308347 | ADAMTS8 | NM_007037 | 8.71 | 7.39E-05 |
| A_32_P224525 | COL6A6 | NM_001102608 | 8.74 | 5.62E-04 |
| A_23_P166929 | SERPINI1 | NM_005025 | 8.79 | 1.21E-05 |
| A_23_P71328 | MATN2 | NM_030583 | 8.95 | 7.20E-06 |
| A_23_P58706 | SPINK13 | NM_001040129 | 9.00 | 1.99E-05 |
| A_21_P0007774 | XLOC_010219 | TCONS_00020953 | 9.13 | 1.39E-04 |
| A_23_P52067 | GRHL3 | NM_198173 | 9.16 | 1.97E-04 |
| A_23_P206359 | CDH1 | NM_004360 | 9.18 | 1.05E-04 |
| A_23_P390068 | C19orf21 | NM_173481 | 9.18 | 1.82E-05 |
| A_23_P207632 | ATP2A3 | NM_174953 | 9.20 | 1.36E-04 |
| A_24_P453497 | RBM20 | NM_001134363 | 9.31 | 7.99E-05 |
| A_19_P00808586 | LOC152742 | NR_033931 | 9.32 | 6.81E-05 |
| A_23_P355517 | SYNPO2L | NM_024875 | 9.33 | 2.44E-04 |
| A_21_P0000747 | LOC100506895 | NR_038276 | 9.89 | 4.44E-05 |
| A_23_P68031 | STAT4 | NM_003151 | 9.96 | 7.97E-05 |
| A_21_P0012988 | XLOC_l2_012054 | ENST00000511794 | 10.21 | 3.25E-04 |
| A_23_P207850 | TNS4 | NM_032865 | 10.26 | 4.35E-06 |
| A_23_P125233 | CNN1 | NM_001299 | 10.39 | 3.52E-06 |
| A_33_P3388391 | GJB4 | NM_153212 | 10.49 | 3.65E-04 |
| A_33_P3229301 | DKFZp434J0226 | NR_027003 | 10.76 | 9.63E-04 |
| A_23_P300033 | PDGFRA | NM_006206 | 10.78 | 2.62E-04 |
| A_21_P0001288 | XLOC_000160 | TCONS_00000918 | 10.86 | 2.82E-04 |
| A_33_P3346483 | SYNPO2L | ENST00000394810 | 10.91 | 1.38E-04 |
| A_23_P83098 | ALDH1A1 | NM_000689 | 11.21 | 3.93E-05 |
| A_23_P24004 | IFIT2 | NM_001547 | 11.41 | 9.09E-05 |
| A_23_P138194 | NCF2 | NM_000433 | 11.56 | 1.78E-05 |
| A_24_P691826 | THC2682885 | THC2682885 | 11.58 | 9.80E-06 |
| A_23_P128744 | BDKRB1 | NM_000710 | 11.59 | 3.06E-05 |
| A_23_P42397 | PRSS35 | NM_153362 | 11.66 | 9.37E-05 |
| A_24_P306594 | LOC100506310 | ENST00000433342 | 11.78 | 7.03E-05 |
| A_23_P204375 | LPAR5 | NM_020400 | 12.44 | 1.31E-04 |
| A_24_P304071 | IFIT2 | NM_001547 | 12.61 | 1.66E-04 |
| A_23_P312150 | EDN2 | NM_001956 | 12.83 | 1.32E-04 |
| A_21_P0009342 | LOC645638 | NR_030732 | 13.05 | 1.19E-06 |
| A_21_P0014296 | LOC100506580 | XR_109204 | 13.08 | 5.86E-05 |
| A_21_P0012715 | LOC152742 | NR_033931 | 13.20 | 2.35E-05 |
| A_24_P389916 | LRRC32 | NM_005512 | 13.87 | 7.15E-05 |
| A_24_P124624 | OLR1 | NM_002543 | 14.48 | 2.10E-04 |
| A_23_P58266 | S100P | NM_005980 | 14.87 | 1.54E-05 |
| A_21_P0013024 | XLOC_l2_012552 | THC2642537 | 15.24 | 2.68E-06 |
| A_33_P3329088 | PRSS8 | NM_002773 | 15.52 | 9.67E-04 |
| A_21_P0002113 | XLOC_002352 | THC2653455 | 16.18 | 1.05E-05 |
| A_23_P166823 | TNNC1 | NM_003280 | 16.36 | 3.94E-05 |
| A_33_P3336686 | CLIC3 | NM_004669 | 16.74 | 1.10E-06 |
| A_33_P3418833 | FLRT3 | NM_198391 | 16.81 | 1.89E-04 |
| A_33_P3708413 | MFAP5 | NM_003480 | 17.35 | 1.66E-05 |
| A_21_P0014895 | LOC100652793 | XR_132879 | 17.75 | 3.28E-04 |
| A_33_P3215640 | PI16 | NM_153370 | 17.98 | 4.44E-04 |
| A_23_P64828 | OAS1 | NM_002534 | 19.06 | 2.41E-06 |
| A_23_P383915 | BTBD16 | NM_144587 | 22.31 | 1.95E-04 |
| A_24_P827037 | LRRC15 | NM_130830 | 28.22 | 1.32E-05 |
| A_32_P189781 | C14orf34 | NR_026796 | 35.20 | 1.11E-04 |
